# Biosynthesis of cannabigerol and cannabigerolic acid: the gateways to further cannabinoid production

**DOI:** 10.1093/synbio/ysad010

**Published:** 2023-05-27

**Authors:** Lewis J Kearsey, Cunyu Yan, Nicole Prandi, Helen S Toogood, Eriko Takano, Nigel S Scrutton

**Affiliations:** Manchester Institute of Biotechnology and School of Chemistry, University of Manchester, Manchester M1 7DN, UK; BBSRC/EPSRC Synthetic Biology Research Centre SYNBIOCHEM, University of Manchester, Manchester M1 7DN, UK; Manchester Institute of Biotechnology and School of Chemistry, University of Manchester; Manchester Institute of Biotechnology and School of Chemistry, University of Manchester, Manchester M1 7DN, UK; Manchester Institute of Biotechnology and School of Chemistry, University of Manchester, Manchester M1 7DN, UK; BBSRC/EPSRC Synthetic Biology Research Centre SYNBIOCHEM, University of Manchester, Manchester M1 7DN, UK; EPSRC/BBSRC Future Biomanufacturing Research Hub, The University of Manchester, Manchester M1 7DN, UK; Manchester Institute of Biotechnology and School of Chemistry, University of Manchester, Manchester M1 7DN, UK; BBSRC/EPSRC Synthetic Biology Research Centre SYNBIOCHEM, University of Manchester, Manchester M1 7DN, UK; EPSRC/BBSRC Future Biomanufacturing Research Hub, The University of Manchester, Manchester M1 7DN, UK

**Keywords:** Cannabigerol, cannabigerolic acid, synthetic biology, *Escherichia coli*, aromatic prenyltransferase

## Abstract

Cannabinoids are a therapeutically valuable class of secondary metabolites with a vast number of substituents. The native cannabinoid biosynthetic pathway of *Cannabis sativa* generates cannabigerolic acid (CBGA), the common substrate to multiple cannabinoid synthases. The bioactive decarboxylated analog of this compound, cannabigerol (CBG), represents an alternate gateway into the cannabinoid space as a substrate either to non-canonical cannabinoid synthase homologs or to synthetic chemical reactions. Herein, we describe the identification and repurposing of aromatic prenyltransferase (AtaPT), which when coupled with native enzymes of *C. sativa* can form an *Escherichia coli* production system for CBGA in cell lysates and CBG in whole cells. Engineering of AtaPT, guided by structural analysis, was performed to enhance its kinetics toward CBGA production for subsequent use in a proof-of-concept lysate system. For the first time, we show a synthetic biology platform for CBG biosynthesis in *E. coli* cells by employing AtaPT under an optimized microbial system. Our results have therefore set the foundation for sustainable production of well-researched and rarer cannabinoids in an *E. coli* chassis.

**Graphical Abstract** 
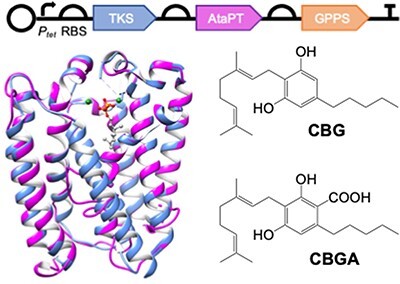

## 1. Introduction

Cannabinoids are a unique class of secondary metabolites that are found exclusively within the plant species *Cannabis sativa* (1). Over 100 compounds have been identified, and a number have been investigated for their therapeutic potential (2). The two most prominent targets are tetrahydrocannabinol (THC) and cannabidiol (CBD) due to their abundance in the plant and well-established therapeutic uses. They have been proven to be effective in treating the side effects associated with chemotherapy, anorexia in HIV patients and reducing spasticity in multiple sclerosis (3, 4).

There is now an interest in investigating the potential therapeutic use(s) of less abundant cannabinoids. One example is cannabigerolic acid (CBGA), the common intermediate to cannabinoid production pathways (5), including THC and CBD. The decarboxylated analog cannabigerol (CBG) also represents an entry point to the synthesis of an array of cannabinoids (6). It has proven efficacy in the treatment of glioblastoma brain tumors (7), reducing neuroinflammation in neurodegenerative diseases (8) and as a treatment for chemotherapy side effects without psychoactive side effects (9).

**Figure 1.**
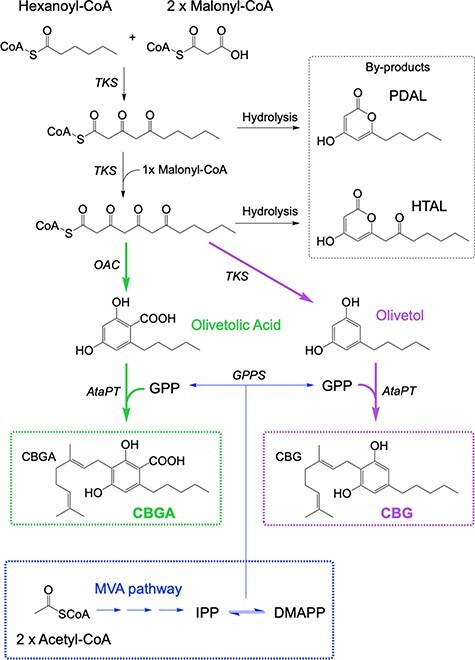
Enzymatic pathways to CBGA and CBG from starting substrates hexanoyl-CoA, malonyl-CoA and GPP. (A) In green is the route to CBGA using TKS, OAC and AtaPT. In purple is the route to CBG using the genes *TKS* and *AtaPT* via the ‘by-product’ olivetol. (B) MVA pathway from 2 acetyl-CoA molecules to IPP and DMAPP encoded on the pMVA NR2 plasmid (Supplementary Information Figure S1). Enzymes: GPPS = geranyl diphosphate synthase and OAC = olivetolic acid cyclase. Chemicals: HTAL = hexanoyl triacetic acid lactone and PDAL = pentyl diacetic lactone.

There is a growing interest to displace existing routes to a variety of natural products currently extracted from plants (or chemically synthesized) with sustainable and renewable biological routes. This is because *C. sativa* extracts contain only small quantities of the target compounds, which are heavily contaminated with a variety of other organic compounds. Therefore, synthetic biology routes to microbially sourced cannabinoids are an attractive proposition. The *C. sativa* metabolic pathway from central metabolites hexanoyl-CoA and malonyl-CoA to cannabinoids has been well characterized (Figure 1 (2, 3, 10–13)). Synthetic biology routes to THC and CBD in *Saccharomyces cerevisiae* were successful by the incorporation of native *C. sativa* pathway enzymes (12). An alternative cell-free system was designed that produced ∼0.5 g/l CBGA or cannabigerovarinic acid from low-cost materials, which was nearly two orders of magnitude higher than yeast-based production (14, 15).

We previously demonstrated the production of THC/CBD precursors olivetolic acid (OA) and by-product olivetol in *Escherichia coli* by expressing the native tetraketide synthase (*TKS*) and OA cyclase (*OAC)* from *C. sativa* (10, 11, 13) (Figure 1). The addition of a promiscuous aromatic prenyltransferase (APT; (16)) to this pathway could enable biosynthetic routes to CBGA and CBG, respectively (Figure 1). This enzyme transfers the prenyl donor geranyl pyrophosphate (GPP) to olivetol and OA, with the former originating from the methylerythritol phosphate pathway of *C. sativa* (17). As the *C. sativa* APTs CsPT1 and four are membrane-embedded, a bacterial homolog would be preferable to maximize the solubility in *E. coli*.

In this study, we describe the proof-of-principle demonstration of a synthetic biology pathway for CBGA and CBG production in *E. coli*, based on the earlier route to OA and olivetol (11). We identified an aromatic prenyltransferase homolog from *Aspergillus terreus* (AtaPT) (18) that uses GPP to prenylate OA and olivetol to CBGA and CBG, respectively. To optimize cannabinoid production, AtaPT variants were generated to improve substrate binding. We performed both *in vitro* and *in vivo* studies to optimize CBGA and/or CBG production, including upregulating GPP precursor availability (19). Overall, this proof-of-principle study demonstrates the potential of microbial routes toward functionalized cannabinoids.

## 2. Materials and Methods

### 2.1 Plasmids and strains

All chemicals and reagents were commercially sourced and were of analytical grade or better. The genes encoding the aromatic prenyltransferases from *C. sativa CsPT1* and *Aspergillus terreus*, *AtaPT* (UniProtKB: A0A455ZIK6 and A0A1B0UHJ4) were synthesized by GeneArt (Thermo Fisher), incorporating codon optimization for improved expression in *E. coli*. Plasmid pBbB2c-OA not only is based on the pBbB2c-TKS-OAC plasmid described previously for the production of OA (11) but also contains the gene encoding GPP synthase (*GPPS*). The mevalonate (MVA) pathway construct (pMVA) was constructed as described previously (19). Plasmid pMVA is a derivative of the limonene-producing construct pJBEI6410 (20) but lacks the genes encoding *GPPS* and limonene synthase (21). It contains operons for the upper and lower MVA pathway (MevT and MevB), controlled by the *lac*UV5 and *trc* promoters, respectively (Supplementary Figure S1). Plasmid pBbB2a-*GPPS-LinS* contains the downstream enzymes catalyzing linalool biosynthesis from isopentenyl pyrophosphate (IPP) and dimethylallyl pyrophosphate (DMAPP) (21). A list of all plasmids used in this study is shown in Table 1.

**Table 1.** Plasmids used in this study

Plasmid	Description^1^	Source/Ref
pBbB2c-OA	pBbB2c; *Cam^R^*; *P_tet_*-*TKS*-*OAC-GPPS*	Dr Nicole Prandi^2^
pMVA	p15A, *Kan^R^*, *P_lacUV5_*-*MevT-TrrnBT1-TT7TE,* *P_trc_-MevB*-TrrnBT1-TT7TE	(19, 21)
pET-M11-*AtaPT*^3^	pET-M11; *Kan^R^*; P_T7_-His_6_-*AtaPT*	Gene art
pBbB2c-CBGA	pBbB2c; *Cam^R^*; *P_tet_*-*TKS-OAC-AtaPT-GPPS*	This study
pBbB2c-CBG	pBbB2c; *Cam^R^*; *P_tet_*-*TKS-AtaPT-GPPS*	This study
pBbB2c-CBGA	pBbB2c; *Cam^R^*; *P_tet_*-*TKS-OAC*-*P_BAD_*-*AtaPT-GPPS*	This study
pBbB2c-CBGA_P_BAD_*	pBbB2c; *Cam^R^*; *P_tet_*-*TKS-OAC*-*P_BAD_*-*AtaPT-GPPS*	This study
pBbB2c-CBGA_P_J23100_*	pBbB2c; *Cam^R^*; *P_tet_*-*TKS-OAC*-*P_J23100_*-*AtaPT-GPPS*	This study
pBbB2c-CBGA_P_J23105_*	pBbB2c; *Cam^R^; P_tet_*-*TKS-OAC*-*P_J23105_*-*AtaPT-GPPS*	This study
pBbB2c-CBGA_P_J23116_*	pBbB2c; *Cam^R^*; *P_tet_*-*TKS-OAC*-*P_J23116_*-*AtaPT-GPPS*	This study
pBbB2c-CBGA_P_J23119_*	pBbB2c; *Cam^R^*; *P_tet_*-*TKS-OAC*-*P_J23119_*-*AtaPT-GPPS*	This study
pBbB2c-CBGA_P_J23150_*	pBbB2c; *Cam^R^*; *P_tet_*-*TKS-OAC*-*P_J23150_*-*AtaPT-GPPS*	This study
pBbB1c-CBGA*	pBbB1c; *Cam^R^*; *P_trc_*-*TKS-OAC*-*AtaPT-GPPS*	This study
pBbB1c-CBGA_P_trc_*	pBbB1c; *Cam^R^*; *P_trc_*-*TKS-OAC*-*P_trc_*-*AtaPT-GPPS*	This study
pBbB1c-CBGA_P_lacUV5_*	pBbB1c; *Cam^R^*; *P_trc_*-*TKS-OAC*-*P_lacUV5_*-*AtaPT-GPPS*	This study
pBbB5c-CBGA*	pBbB1c; *Cam^R^*; *P_lacUV5_*-*TKS-OAC-AtaPT-GPPS*	This study
pBbB5c-CBGA_P_trc_*	pBbB1c; *Cam^R^*; *P_lacUV5_*-*TKS-OAC*-*P_trc_*-*AtaPT-GPPS*	This study
pBbB5c-CBGA_P_lacUV5_*	pBbB1c; *Cam^R^*; *P_lacUV5_*-*TKS-OAC*-*P_lacUV5_*-*AtaPT-GPPS*	This study

1 Plasmid backbone, antibiotic marker and promoter-operon.

2 University of Manchester, UK.

3 Additional clones with mutations in AtaPT are described in the Supplementary Results S3 section. Genes: *OAC* = olivetolic acid cyclase; *TKS *= tetraketide synthase. Clones indicated with an asterisk are discussed in the Supplementary Results S3 section. The Genbank accession numbers for each plasmid generated in this study are in the Supporting Information document.

Coding sequences for assembly of the OA-producing construct (pBbB2c-OA) were codon optimized for the expression in *E. coli* and ordered from Twist Bioscience. The parts were amplified via PCR using Phusion Hotstart Flex New England Biolabs (NEB) and assembled on a pBbB2c backbone using the ligase cyclic reaction method as described previously (22). The correct assembly was confirmed via restriction analysis and DNA sequencing (GATC Biotech).

Recombinant protein expression was performed using *E. coli* strains ArcticExpress (DE3) (Agilent Technologies), BL21 (DE3) and NEB5α (New England Biolabs). Olivetol and CBG production were also tested using the *E. coli* DH5α Δ*fabF* variant (23), designed to reduce malonyl-CoA consumption in fatty acid biosynthesis by knocking out the 3-oxoacyl-(acyl carrier protein) synthase II (*fabF*) gene.

### 2.2 Subcloning and mutagenesis

All oligonucleotides used for cloning and mutagenesis are listed in Supplementary Tables S1 and S2. Both the *CsPT1* and *AtaPT* genes were subcloned into pETM11 by restriction cloning at the *Xho*I and *Nco*I sites, which incorporated an N-terminal His_6_-tag. The *AtaPT* variants E91A, E91D and E91Q were generated using the Q5 site-directed mutagenesis kit (New England Biolabs), according to the manufacturer’s instructions.

The following modified plasmids were generated in this study: The OA plasmid pBbB2c-OA was modified to replace the *OAC* gene with *AtaPT*. This plasmid was modified to insert the variant *AtaPT_E91Q_* gene to generate pBbB2c-CBGA (pBbB2c-*TKS-OAC-AtaPT_E91Q_-GPPS*). The GPPS precursor pathway plasmid pMVA (21) was modified to incorporate a constitutive J23116 promoter in place of the existing *trc* promoter upstream of the MVA kinase (*ScMK)* gene (Supplementary Figure S1). The correct assembly of each construct was confirmed by gene sequencing (Eurofins Genomics). Further details of the cloning and assembly of each construct are found in Supplementary Method S1.

### 2.3 Enzyme kinetics and biotransformations of purified proteins

The expression and purification of individual enzymes TKS and OAC were performed in *E. coli* (DE3) ArcticExpress using the protocols described previously (11). Both wild-type (WT) *AtaPT* and variant *AtaPT* were expressed in *E. coli* (DE3) ArcticExpress, and details of purification protocol are provided in Supplementary Method S2.

Steady state kinetics of purified WT and variant AtaPT was performed using an endpoint assay. Reaction mixtures (200 μl) were composed of buffer (25 mM Tris pH 8, 150 mM NaCl and 5% glycerol) containing GPP (1 mM), AtaPT (1 μM; 10 μM for WT) and OA (0–10 mM). Each reaction mixture was incubated for 20 min at 25°C. Biotransformations with purified enzymes were performed in a similar manner, except for the reaction volume (250 μl), incubation time (overnight), substrate concentration (10 μM each of hexanoyl-CoA, malonyl-CoA, OA and/or GPP) and enzyme concentrations (10 μM; TKS, OAC and/or WT or variant AtaPT).

Each reaction was stopped by the addition of an equal volume of ethyl acetate. The samples were vortexed to extract the substrate and product into the organic phase and then clarified by centrifugation for 10 min at 17 900 *g*. The organic phase (150 μl) was removed, dried in a vacuum centrifuge and then resuspended in an equal volume of 50% methanol for quantitative liquid chromatography–mass spectrometry (LC–MS) analysis. All assays were performed in triplicate, with the errors representing one standard deviation of the data.

### 2.4 *In vitro* biotransformations for CBGA production

*Escherichia coli* strain BL21 (DE3) was co-transformed with plasmids pBbB2c-OA and pETM11-*AtaPT_E91Q_* according to the manufacturer’s protocol. Biological triplicate cultures (50 ml) were cultivated in Luria-Bertani media (LB: 10 g/l tryptone, 5 g/l yeast extract and 10 g/l NaCl) containing 40 μg/ml kanamycin and 35 μg/ml chloramphenicol. The cultures were incubated at 37°C until an optical density at 600 nm of 0.6–0.8 was reached. The incubation temperature was reduced to 16°C, and recombinant protein expression was induced with 0.1 mM isopropyl-β-d-thiogalactopyranoside (IPTG) and 200 nM anhydrotetracycline (aTet) for plasmids pETM11-*AtaPT_E91Q_* and pBbB2c-OA, respectively. Cultures were incubated overnight at 16°C and then harvested by centrifugation, and cell lysates were generated according to the method in Supplementary Method S3.

Reaction mixtures (∼1 ml) were composed of 1 ml of lysate containing 10 μM each of hexanoyl-CoA, malonyl-CoA and GPP. Mixtures were incubated overnight at 25°C followed by the addition of 900 μl of ethyl acetate to 900 μl of the reaction mixture. Product(s) were extracted into the organic layer by vortexing, and emulsion clarification was performed by centrifugation for 10 min at 17 900 *g*. The organic layer (750 μl) was dried using a vacuum centrifuge and resuspended in 150 μl of 50% methanol for MS analysis. Error bars represent one standard deviation of the data from biological triplicate reactions.

### 2.5 *In vivo* biotransformations

#### pMVA NR2 in vivo testing.

The functional expression of pMVA_NR2 to produce GPP precursors IPP and DMAPP was tested by co-expressing it with plasmid pBbB2a-*GPPS-LinS* containing the remaining biosynthetic genes to the monoterpenoid linalool (21). These plasmids were co-transformed into *E. coli* NEB5α, and individual colonies were inoculated into 3 ml of phosphate-buffered Terrific broth (TB) media (Formedium; 12 g/l tryptone, 24 g/l yeast extract, 9.4 g/l KH_2_PO_4_ and 2.2 g/l K_2_HPO_4_) containing 40 μg/ml kanamycin and 100 μg/ml ampicillin. The cultures were grown at 37°C until growth was visible and then induced with 50 μM IPTG and 200 nM aTet for plasmids pMVA_NR2 and pBbB2a-*GPPS-LinS*, respectively. Cultures were incubated at 30°C for 72 h, and linalool was extracted by vortexing with 1 ml of ethyl acetate. The emulsion was clarified by centrifugation for 10 min at 17900 *g*, and the organic solvent was dried with anhydrous MgSO_4_. Linalool content was quantified by Gas Chromatography Mass Spectrometry (GCMS), with the error bars representing the standard deviation of data from five biological triplicates.

#### In vivo *production of cannabinoids.*

The constructs pBbB2c-CBG or pBbB2c-CBGA were co-transformed with pMVA_NR2 into *E. coli* NEB5α and the *E. coli* DH5α Δ*fabF* variant using manufacturer-specified chemical transformation and electroporation protocols, respectively. Electroporation was performed using a MicroPulser (Bio-Rad) with the bacterial protocol *E. coli* 2 (0.2 cm cuvette, 200 Ω current, 25 μF capacitance and 2.5 kV). Single colonies from the transformations (biological triplicates) were inoculated into phosphate-buffered TB media containing 0.4% glycerol, 40 μg/ml kanamycin and 35 μg/ml chloramphenicol. Cultures (3 and 25 ml for olivetol and CBG production, respectively) were cultivated at 37°C for 8 h and then induced with 50 μM IPTG and 200 nM aTet. The cultures were incubated for 72 h at 20°C, 25°C and 30°C, where specified. Aliquots (2 ml) of each culture were extracted with 2 ml of ethyl acetate, as described earlier. The organic layer (1.5 ml) was dried using a vacuum centrifuge and resuspended in 150 μl of 50% methanol for LC–MS analysis.

For monitoring the distribution of olivetol and CBG between culture medium and cells, the same protocol as earlier was used with four biological replicate cultures (25 ml) cultivated after induction at 30°C. Culture samples (25 ml) were centrifuged at 8000 *g*, and 2 ml of the clarified supernatant was extracted with ethyl acetate and processed for LC–MS analysis as earlier. The pelleted cells were resuspended in 2.5 ml of lysis buffer and lysed as described in Supplementary Method S3. The lysate (2 ml) was extracted with an equal volume of ethyl acetate and processed for LC–MS analysis as described earlier.

### 2.6 Analytical procedures

The product profile of AtaPT was identified and quantified using LC–MS using a 1290 Infinity II UHPLC coupled to a 6560 Ion Mobility Q-TOF (Agilent). The quantitative analysis of product formation was performed by using a Xevo TQ-S triple quadrupole tandem mass spectrometer (Waters MS Technologies) connected to an Acquity UPLC system (H-Class; Waters). Olivetol and CBG separation was performed by LCMS on a BEH C8 column (1.7 µm, 2.1 × 50 mm, Waters). Linalool quantitation was performed by GCMS using an Agilent Technologies 7890B GC equipped with a 5977A MSD detector. Product concentrations were determined by comparing peak areas to a standard curve generated from authentic standards run under identical conditions. Further details of the running conditions for compound identification and quantitation are found in Supplementary Methods S4.

## 3. Results and Discussion

### 3.1 Identification and engineering of prenyltransferase homolog AtaPT

We sought to identify potential candidates of non-membrane-bound soluble aromatic prenyltransferases as the expression of the *C. sativa* prenyltransferase *CsPT1* did not yield soluble protein (Supplementary Results S1 and Figure S2). To identify potential candidates, a SWISS-MODEL (24–29) of CsPT1 (GenBank: BK010678.1) was generated, based on the crystal structure of the membrane-embedded prenyltransferase (UBiA) from *Archaeoglobus fulgidus* (30) (Protein Data Bank: 4TQ3; https://www.rcsb.org/), to identify the likely sequence regions encoding membrane anchors (Supplementary Figure S3). The related UBiA superfamily member *C. sativa* homolog CsPT4 was predicted to contain eight transmembrane helices (12), which may account why these enzymes are difficult to solubly express in *E. coli*. To bypass this problem, alternative enzymes that do not contain the transmembrane domains were sought. The aromatic prenyltransferase from *Aspergillus terreus* (AtaPT) was identified, which was previously shown in the literature to express in soluble form in *E. coli* and has exceptional promiscuity toward diverse aromatic acceptors and prenyl donors (18). The crystal structure of AtaPT is known (18), which confirms the absence of the transmembrane helices seen with UBiA homologs.

**Figure 2.**
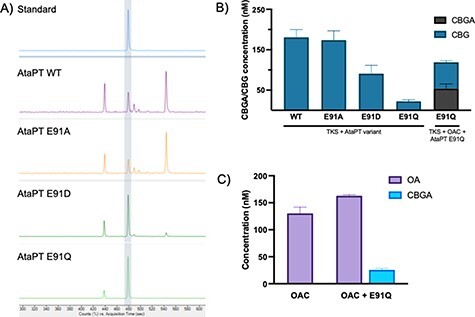
*In vitro* activity of WT and variants of AtaPT. (A) Product profiles of purified WT AtaPT and variants E91A, E91D and E91Q AtaPT variants from reactions with OA and GPP. Reaction mixtures (250 μl) were composed of 10 μM each of OA and GPP and 10 μM of AtaPT in buffer (25 mM Tris pH 8, 150 mM NaCl and 5% glycerol). After incubating at 25°C for 20 min, the products were extracted with ethyl acetate and dried. Each sample was redissolved in 50% methanol and analyzed using a 6560 Ion Mobility Q-TOF LC–MS. The full spectra are shown in Supplementary Figure S5. The peaks representing CBGA are highlighted as a vertical shaded bar. (B) *In vitro* screening of purified WT AtaPT and variants for CBG production in the presence or absence of OAC. Reactions were performed as described in (A). Error bars represent one standard deviation of biological triplicates. (C) Lysate assay of *E. coli* BL21 (DE3) expressing pBbB2c-OA ± pETM11-*AtaPT_E91Q_*. Reaction mixtures were composed of 1 ml of cell lysate with 10 μM each of hexanoyl-CoA, malonyl-CoA and GPP. Samples were extracted and analyzed as earlier. 1 nM CBGA and CBG are equivalent to 0.361 and 0.316 μg/ml, respectively.

After successful expression in *E. coli* (Supplementary Figure S4), biotransformations of purified AtaPT showed that CBGA was produced with four major side products (Figure 2a; Supplementary Figure S5). These by-products may be isomers of CBGA, where the highly promiscuous AtaPT has prenylated OA at multiple positions on its ring. Prior studies showed that the AtaPT product profile distribution could be altered by mutating residue Glu 91 to alanine, aspartate or glutamine without adversely affecting the overall catalytic activity (18). This is so because residue 91 appears to discriminate between the orientation of the aromatic substrates as it enters the active site. We presumed that a similar discrimination between substrate orientations could be generated by making the equivalent Glu91 variants in AtaPT. We found that both AtaPT_E91D_ and AtaPT_E91Q_ produced primarily CBGA with a reduction in by-product formation (Figure 2a), while AtaPT_E91A_ showed a WT-like product profile. There appears to be an interplay between changes in the residue R-group charge and/or steric bulk, which affects the prioritization of OA binding in an orientation favoring CBGA production.

### 3.2 Kinetics of WT and variant AtaPT

The apparent kinetic constants of purified WT and AtaPT variants with OA were determined to further investigate the effect of the mutations (Table 2; Supplementary Figure S6). True Michaelis–Menten kinetics constants were not determined due to the low specific activity of each enzyme (i.e. the condition that [E] <<< [S] was not possible) (31). Variants AtaPT_E91Q_ and AtaPT_E91A_ showed an increase in OA binding specificity (94 ± 32 μM and 332 ± 96 μM, respectively; versus WT 1083 ± 126 μM). As these two variants display quite different product profiles, the common increase in OA binding is likely due to different factors, such as changes in the residue charge and/or the removal of steric bulk to allow OA to bind more favorably.

**Table 2.** Apparent Michaelis-Menten kinetics of wild-type AtaPT and variants

AtaPT Variant	Apparent *K*_m_ (μM)	Apparent *k*_cat_ (min^−1^)
Wild type	1083 ± 126	0.0088 ± 0.0003
E91A	332 ± 96	0.0149 ± 0.0013
E91D	266 ± 44	0.0417 ± 0.0019
E91Q	94 ± 32	0.131 ± 0.0094

Apparent Michaelis-Menten plots can be found in Supplementary Figure S6. These kinetic constants are only approximations as the low steady state activity of the enzymes required relatively high enzyme concentrations to be used (31).

The apparent *k*_cat_ of WT and AtaPT_E91A_ showed similar turnover numbers (Table 2), while AtaPT_E91Q_ showed an almost 15-fold increase in apparent *k*_cat_ (0.131 ± 0.0094 min^−1^). Additional variants were generated based on studies with the homologous aromatic prenyltransferase NphB from *Streptomyces* sp. strain CL190 (32), but they did not improve the activity and/or specificity of AtaPT (Supplementary Results S2 and Figures S7 and S8). Therefore, AtaPT_E91Q_ was taken forward for *in vivo* CBGA production due to its high specificity and lower side product formation.

### 3.3 *In vitro* multienzyme production of CBG and CBGA

Olivetol is the decarboxylated analog of OA generated by the action of TKS alone in the absence of OAC (Figure 1). Using olivetol in place of OA, AtaPT could potentially generate CBG (Figure 1). This may be an alternate entry point into the lucrative, therapeutically active, decarboxylated cannabinoid chemical space. Biotransformations of purified TKS with WT and variant AtaPT all generated CBG (Figure 2b), with WT AtaPT and AtaPT_E91A_ showing the highest titers (180.6 ± 19.0 nM and 173.7 ± 23.0 nM, respectively). As expected, the addition of enzyme OAC showed that both CBGA and CBG were produced, with similar titers of 53.7 ± 11.8 nM and 65.3 ± 4.3 nM, respectively (Figure 2b). This decrease in CBG production in the presence of enzyme OAC is likely due to carbon being split between the two biosynthetic routes (dual products). Surprisingly, there are lower titers of CBG with the AtaPT_E91Q_/TKS construct (22.1 ± 3.9 nM) compared to the same construct with OAC being present (65.3 ± 4.3 nM). This may be due to the general observation that the addition of extra genes to an operon can lead to changes in the level of expression of the other genes already present. Therefore, both WT and AtaPT_E91A_ were taken forward to investigate *in vivo* CBG production studies.

In the next stage, *in vitro* multienzyme biotransformations for CBGA production were performed using *E. coli* cell lysates of co-expressed TKS, OAC and AtaPT_E91Q_ as the enzyme source (Figure 2c; OAC-AtaPT route in Figure 1). Both the OA precursor and CBGA were generated (163. ± 1.8 nM and 25.8 ± 2.5 nM, respectively), showing the active expression of each enzyme. The accumulation of OA suggests that further optimization is needed to upregulate *AtaPT_E91Q_* expression and/or activity to shift the equilibrium toward CBGA production. This proof-of-principle lysate system shows the potential of using TKS, OAC and AtaPT_E91Q_ in a more cost-effective *in vivo* microbial CBGA production process, which will eliminate the need for supplying costly and unstable coenzyme A derivatives.

### 3.4 *In vivo* biotransformations in *E. coli* for cannabinoid production

*In vivo* biotransformations were performed in *E. coli* expressing *TKS, OAC* and *AtaPT_E91Q_* (pBbB2c-CBGA) to generate CBGA (and CBG; Supplementary Results S3). The only cannabinoid product detected was OA (5–10 μg/ml; Supplementary Figure S9), so a variety of approaches were used to identify and overcome bottlenecks in the system and perform optimization studies to allow metabolic flux through to CBGA (or CBG). These approaches were based on (i) incorporating the gene GPPS to increase substrate GPP titers for AtaPT activity (Supplementary Results S3 and Figure S9a), (ii) generating a library of twelve genetic circuits with a selection of inducible (33) and constitutive (34) promoters positioned at different points throughout the pathway (Supplementary Figure S9a and b), (iii) increasing GPP supply by incorporating a heterologous MVA pathway (Supplementary Results S4 and Figure S10) and (iv) upregulating intracellular malonyl-CoA levels by using the *E. coli* strain DH5α Δ*fabF* knock-out (Supplementary Results S5 and Figure S10).

**Figure 3.**
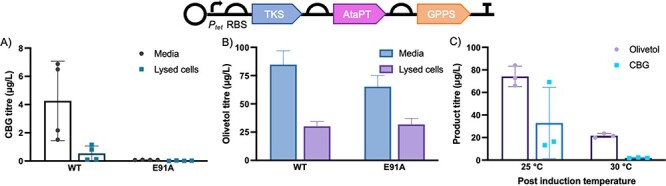
Olivetol and CBG production from cells or cell lysate expressing the pBbB2c-CBG plasmid. Inset: Schematic of genes used in CBG producing constructs. (A) CBG production and localization within the cell or culture supernatant in whole cell biotransformations of constructs containing AtaPT WT and E91A variant. CBG concentration in lysed cells is normalized to the total volume of fermentation. (B) Olivetol localization within the cell or culture supernatant in whole cell biotransformations. Olivetol concentration in lysed cells is normalized to the total volume of fermentation. (C) Production of CBG and olivetol in whole cell biotransformations at different post-induction temperatures. In each part, cultures (25 ml) in TB media containing 0.4% glycerol were grown at 37°C for 8 h then induced and incubated at 30°C for 72 h.

The culmination of these studies led to a redesign of biotransformations for CBG and CBGA production by incorporating the gene for GPPS, co-expressing the MVA pathway on a second plasmid (pMVA) and switching to the *E. coli* strain DH5α Δ*fabF*. This led to the production of low levels of CBG (4.3 ± 2.8 μg/l and 0.1 ± 0.01 μg/l, respectively; Figure 3a) using the pBb-*CBG-GPPS* construct with a WT AtaPT and E91A variant, respectively. In contrast, when using the pBbB2c-CBGA plasmid, a four-fold increase in OA production was seen (Supplementary Table S3), but no CBGA was detected.

This is the first time that CBG has been produced in a bacterial whole cell system. Unlike the lysate production system, higher titers were seen with WT AtaPT instead of the E91A variant. This may be due to different recombinant protein expression conditions between the *in vitro* and *in vivo* assays. Almost all the CBG was found to be residing in the culture supernatant, with only 11% within the cells (Figure 3a). Higher levels of olivetol were also detected with WT AtaPT, with 84.6 ± 12.2 μg/l found in the supernatant and 30.2 ± 4.1 μg/l in the lysate (Figure 3b). This suggests that there is a secretion system in *E. coli* capable of exporting some cannabinoids.

The highest titer of CBG (32.9 ± 31.5 μg/l) was obtained, albeit with high variability, after the post-induction temperature was maintained at 25°C (Figure 3c). High variability in biological replicates (individual colonies) may arise from different levels of plasmid incorporation and/or accumulation of mutation/recombination events, impacting the function of the incorporated pathway. Olivetol was also present at over double the titers, suggesting that GPP supply and/or AtaPT expression/activity is still a bottleneck in this system. While these titers are low, this proof-of-principle demonstration of *in vivo* bacterial cannabinoid production shows the potential of this ‘natural’ biological route.

Despite the demonstration of cannabinoid biosynthesis in yeast, there is still a desire to establish a production platform in an *E. coli* chassis due to its preference for industrial application owing to its 3- to 4-fold faster growth rate than *S. cerevisiae*. Alongside this, a well-characterized genome and established genetic toolbox would allow an *E. coli* system to diverge from the native enzymes of *C. sativa*, which the current *S. cerevisiae* platform relies on. This enables the exploration of homologous enzymes and novel routes to rarer cannabinoids, such as those derived from CBG.

## 4. Conclusion

This study has demonstrated a proof-of-principle demonstration of complete *in vivo* bacterial CBG production. Previous studies into cannabinoid production in an *E. coli* host only progressed as far as demonstrating OA production in a whole cell system (11). Other approaches include the construction of an *E. coli* prenylation system into which OA is fed to produce CBGA (35) or utilizing a cell-free system for cannabinoid production (14). Here, the use of a non-native prenyltransferase coupled with two substrate upregulation (GPP and malonyl-CoA) led to the development of a fully *in vivo* system for CBG production in *E. coli*. Further studies are required to increase intermediate and final product titers to commercially relevant g/l quantities.

Our construction of a CBG producing strain of *E. coli* demonstrates that by coupling enzymes of native biosynthesis pathways with homologs that show promiscuity toward their substrate, novel routes to therapeutically valuable compounds can be constructed. Here, we employed the native enzymes of *C. sativa* coupled with a promiscuous aromatic prenyltransferase to open two biosynthetic gateways into the cannabinoid chemical space, via the intermediates CBGA and CBG. Further delving into non-native enzymes capable of modifying these products, or other intermediates of cannabinoid synthesis, may well represent non-canonical routes to bioactive compounds, which until now have not been possible to study due to their lack of abundance in the *C. sativa* plant.

## Supplementary Material

ysad010_Supp

## Material availability statement

Materials used in this study are available on request to the corresponding author.
