# Supplementary material for: Biosynthesis of cannabigerol and cannabigerolic acid: the gateways to further cannabinoid production
Source: Synth Biol (Oxf). 2023 May 27;8(1):ysad010. doi: 10.1093/synbio/ysad010 (PMC10263468; doi:10.1093/synbio/ysad010)
Supplement: ysad010_Supp [file ysad010_supp.zip › suppl_data/SI_Scrutton_SYNBIO-2023-004_180523.docx]

# Supplementary Information

# Biosynthesis of cannabigerol and cannabigerolic acid, the gateways to further cannabinoid production.

**Lewis J. Kearsey,^1^ Cunyu Yan,^2^ Helen S. Toogood,^1^ David Leys,^1^ Eriko Takano^1,2, 3^ and Nigel S. Scrutton^1,2,3*^**

^1^Manchester Institute of Biotechnology and School of Chemistry, ^2^BBSRC/EPSRC Synthetic Biology Research Centre SYNBIOCHEM, and ^3^EPSRC/BBSRC Future Biomanufacturing Research Hub, The University of Manchester, 131 Princess Street, Manchester, M1 7DN, United Kingdom. ^4^Current address; Colorifix Ltd, Norwich Research Park Innovation Centre, Colney Ln, Norwich NR4 7GJ, United Kingdom.

*Corresponding author: nigel.scrutton@manchester.ac.uk; Tel: +44 (0) 161 306 5152.

**Contents**

| **Supplementary Materials and Methods** | | |  |
| --- | --- | --- | --- |
| Method S1 | | Cloning and assembly of plasmids | 3 |
| Method S2 | | Protein expression and purification | 3 |
| Method S3 | | Production of cell lysates for CBGA in vitro biotransformations | 5 |
| Method S4 | | Analytical procedures | 5 |
| **Supplementary Results and Discussion** | | |  |
| Results S1 | | Expression of *C. sativa* prenyl transferase CsPT1 | 6 |
| Results S2 | | Generation of additional variants of AtaPT | 6 |
| Results S3 | | *In vivo* biotransformations for CBGA production | 7 |
| Results S4 | | *In* vivo screen incorporating an MVA pathway for CBGA production | 7 |
| Results S5 | | Upregulating intracellular malonyl-CoA levels | 8 |
| **Supplementary Figures** | | |  |
| Fig. S1 | Plasmid map of the pMVANR3 construct. | | 9 |
| Fig. S2 | SDS PAGE and Western blot analysis of the expression of native aromatic prenyl transferase (APT) from *C. sativa* in *Escherichia coli*. | | 10 |
| 9Fig. S3 | SWISS-MODEL structure of CsPT1 modelled on the crystal structure of UBiA. | | 11 |
| Fig. S4 | SDS-PAGE analysis of IMAC purifications of WT *Aspergillus terreus* aromatic prenyl transferase (AtaPT) and variants E91A, E91D and E91Q. | | 12 |
| Fig. S5 | Product profiles of biotransformations with wild-type AtaPT and variants E91A, E91D and E91Q. | | 13 |
| Fig. S6 | Apparent Michaelis Menten plots of A) wild-type and AtaPT variants B) E91A, C) E91D and D) E91Q with OA. | | 14 |
| Fig. S7 | SDS-PAGE analysis of IMAC purifications of variants of AtaPT. | | 15 |
| Fig. S8 | Product profiles of biotransformations with wild-type AtaPT and variants Y344S, E91A/Y344S and Y344S/Y413S. | | 16 |
| Fig. S9 | Pathways to produce OA and CBGA in *E. coli*. | | 17 |
| Fig. S10 | Screening of multiple recombinant constructs and reaction conditions for GPP precursor upregulation and CBG production | | 18 |
| **Supplementary Tables** | | |  |
| Table S1 | Primers used for cloning and mutagenesis. | | 19 |
| Table S2 | Primers used for cloning and mutagenesis of libraries of clones. | | 20 |
| Table S3 | In vivo production of cannabinoids in *E. coli* pBb-CBGA / pMVA NR2. | | 21 |
| **Supplementary Plasmid Sequences** | | | 22 |
| **References** | | | 63 |

**Supplementary Materials and Methods**

**Method S1. Cloning and assembly of plasmids**

The olivetolic acid plasmid pBbB2c-OA was modified to replace the *OAC* gene with *AtaPT*. PCR linearisation of the pBbB2c-OA was performed with OAC excision and amplification of the AtaPT gene with 15 bp complementary overhangs and hexahistidine tag removal. This was followed by In-Fusion cloning (Takara Bio) to insert the *AtaPT* gene, which generated plasmid pBbB2c-CBG (pBbB2c-TKS-AtaPT-GPPS). A second PCR linearisation of pBbB2c-OA was performed without OAC elimination and amplification of AtaPT_E91Q_ with 15 bp complementary overhangs. Subsequent In-Fusion cloning was performed to insert the AtaPT_E91Q_ gene, generating pBbB2c-CBGA (pBbB2c-TKS-OAC-AtaPT_E91Q_-GPPS). The GPPS precursor pathway plasmid pMVA (1) was modified to incorporate a constitutive J23116 promoter in place of the existing *trc* promoter upstream of the mevalonate kinase (ScMK) gene. This was performed by PCR opening of pMVA to excise the *trc* promoter while simultaneously introducing the J23116 constitutive promoter. The PCR oligonucleotides contained complementary 15 bp that facilitated recirculation by In-Fusion cloning to generate plasmid pMVA NR2 (**Supplementary Figure S1**).

A library of variants of plasmid pBbB2c-CBGA were generated as it was thought that the lack of CBGA production was due to insufficient AtaPT_E91Q_ expression or suboptimal metabolic flux. A library of promoters were introduced to pBbB2c-CBGA upstream of TKS and AtaPT_E91Q_. These promoters included the L-arabinose inducible *P_BAD_*, the IPTG inducible promoters *P_Trc_* and *P_lacUV5_*, and a series of constitutive promoters from Registry of Standard Biological Parts (http://parts.igem.org/Main_Page). The library of gene cassettes constructed is displayed schematically in **Supplementary Figure S9a**.

To introduce the promoter *P_BAD_*, both the *araBAD* promoter and L-arabinose regulatory protein (*araC*) were cloned upstream of AtaPT_E91Q_. This meant that a bi-directional terminator also needed to be introduced to stop mis-transcription of *araC* or OAC due to both being orientated in different directions in the plasmid. The bi-directional terminator LuxICDABEG (+/-) was selected and cloned into position between OAC and araC. The plasmid map shown in **Supplementary Figure S9b** shows this design.

To insert the *P_BAD_* promoter, *P_BAD_* was amplified from pBbB8c-GFP and pBbB2c-CBGA was linearised through PCR using CloneAMP HiFi PCR premix. The amplified fragment was ligated into the linearised vector using In-Fusion cloning. The terminator LuxICDABEG (+/-) was introduced via the insertion capabilities of the Q5-mutagenisis kit (New England Biolabs) following the manufactures protocol.

To construct the IPTG inducible constructs, the desired promoter (*P_trc_* or *P_lacUV5_*) was inserted upstream of AtaPT_E91Q_. CloneAMP HiFi PCRs amplified the respective promoter and linearise the plasmid construct. Ligation and recirculation of the new plasmids was performed using the kinase, ligase and DpnI reaction from the Q5-mutagenisis kit. The second stage involved cloning the whole gene cassette into a backbone containing the *lac* repressor. Both pBbB1c and pBbB5c were chosen as backbone vectors as this would enable the testing of both *P_trc_* and *P_lacUV5_* upstream of the pathway, to allow a greater degree of insight into metabolic flux. This was performed by PCR amplification of each multi-gene cassette with promoter. The backbone vectors (pBbB1c and pBbB5c) were linearised via restriction enzyme digestion using NdeI and BamHI. Assembly of the new library plasmids (**Table 1**) was performed via the Q5-mutagenisis kit.

A set of constitutive promoters upstream of AtaPT_E91Q_ were designed using the online software SelProm ((2); <http://selprom.synbiochem.co.uk>). The parameters used were A) reporter = RFP; B) Hhost = BL21(DE3); C) media = TB; D) resistance = chloramphenicol; E) induction = 100 μM IPTG; F) induction point = 20% OD; G) promoter = PT7 and H) Origin = BBR1. The three constitutive promoters from the Registry of Standard Biological Parts; J23105, J23116 and J23150, were predicted by SelProm to have the most suitable relative strength. When compared to other constitutive promoters from the Registry of Standard Biological Parts, such as those of the Anderson collection, these promoters are relatively weak. The two strongest promoters of the Anderson collection were also selected (J23100 and J23119).

PCR amplification with CloneAMP HiFi PCR was used to generate the constitutive promoters and linearise the constructs. The kinase, ligase and DpnI reaction from the Q5-mutagenisis kit was employed to ligate and re-circularise the new constructs.

In each case, PCR products were analysed by agarose gel electrophoresis, extracted and purified before use. Plasmids were transformed into *E. coli* strain NEB5α and cultivated overnight in LB agar containing chloramphenicol (35 μg/ml) or kanamycin (40 μg/ml), where appropriate. Individual colonies were cultivated overnight in LB medium containing chloramphenicol (35 μg/ml) or kanamycin (40 μg/ml), where appropriate, followed by plasmid recovery and purification. The correct assembly of each construct was confirmed by gene sequencing (Eurofins Genomics). All PCR primers are listed in **Supplementary Tables S1-S2**. All gene sequences of the plasmids and their Genbank accession numbers are listed in the Supplementary Plasmid Sequences at the end of this document.

**Method S2. Protein expression and purification**

Expression of wild type (WT) and variant AtaPT was performed in *E. coli* (DE3) ArcticExpress. In this method, cultures (1 L) were grown in 2xYT broth (Formedium; 16 g/L tryptone, 10 g/L yeast extract and 5 g/L NaCl) containing 40 μg/mL kanamycin and 20 μg/mL gentamycin. Cultures were incubated at 37 °C and 200 rpm until an OD at 600 nm of 0.6-0.8 was reached. The incubation temperature was reduced to 16 °C, followed by IPTG induction (0.1 mM) and an overnight incubation. Cells were harvested by centrifugation at 8,000 *g* for 10 minutes at 4 °C.

The cell pellets were resuspended in lysis buffer (10 mL per 6 g pellet) consisting of 25 mM Tris pH 8, 150 mM NaCl and 5 % glycerol, followed by DNAse I (10 ng/mL) and a cOmplete mini EDTA-free Protease Inhibitor Cocktail (Roche) after a uniform slurry was achieved. The slurry was sonicated for 15 minutes (Bandelin sonopuls) at an amplitude of 35 %, pulsing 10 s on and 25 s off. The lysate was centrifuged at 40,000 *g* for 30 minutes at 4 °C, and the supernatant was filtered to remove intact cells (0.45 micron; Sartorius Stedim Biotech). Each AtaPT variant was purified by passage through a His Trap FF column (5 mL; GE Healthcare), pre-equilibrated in lysis buffer. The column was washed with a stepwise gradient of lysis buffer containing increasing imidazole concentrations (0-50 mM; 2 column volumes per step). AtaPT was eluted in a stepwise gradient of lysis buffer containing 200 then 500 mM imidazole (12 column volumes each). Eluates were analysed for AtaPT content and purity by SDS PAGE analysis using 12 % Mini-PROTEAN TGX Stain-Free precast gels, using the manufacturer’s protocol. Bands were visualised using the BioRad Gel Doc EZ imager. Western blots were performed on the SDS PAGE analytical gels by transferring the protein bands onto a Midi PVDF 0.2 µm membrane using the Trans-Blot Transfer system (BioRad). Subsequent antibody incubations with His Tag monoclonal antibody (mouse; Novagen), washings and signal development was performed using the iBind Western device (Life Technologies), according to the manufacturer’s protocols.

**Method S3. Production of cell lysates for CBGA in vitro biotransformations**

*E. coli* strain BL21 (DE3) was co-transformed with plasmids pBbB2c-OA and pETM11-AtaPT_E91Q_ according to the manufacturer’s protocol. Biological triplicate cultures (50 mL) were cultivated in LB media (10 g/L tryptone, 5 g/L yeast extract and 10 g/L NaCl) containing 40 μg/mL of kanamycin and 35 μg/mL chloramphenicol. The cultures were incubated at 37 °C until an OD 600 nm of 0.6-0.8 was reached. The incubation temperature was reduced to 16 °C and recombinant protein expression was induced with 0.1 mM IPTG and 200 nM anhydrotetracycline (aTet) for plasmids pETM11-AtaPT_E91Q_ and pBbB2c-OA, respectively. Cultures were incubated overnight at 16 °C then harvested by centrifugation at 8,000 *g* for 10 minutes at 4 °C. The cell pellets were resuspended in lysis buffer containing 10 ng/mL DNAse I, 100 ng/mL lysozyme, 100 ng/mL ampicillin and a cOmplete mini EDTA-free protease inhibitor cocktail tablet. The cell slurry was incubated for 30 minutes at 4 °C then lysed with four freeze thaw cycles using liquid nitrogen. The lysate was clarified by centrifugation at 17,900 *g* for 30 minutes at 4 °C, and the supernatant was used as the enzyme source for in vitro biotransformations.

**Method S4. Analytical procedures**

The product profile of AtaPT was identified and quantified using LC-MS using a 1290 Infinity II UHPLC coupled to a 6560 Ion Mobility Q-TOF (Agilent). In this method, 5 μL of samples in methanol were injected and separated using a Acquity UPLC BEH C18 column (1.7 µm, 2.1 x 50 mm, Waters) with a mobile phase gradient of solvent A (0.05 % formic acid in water) to solvent B (0.05 % formic acid in acetonitrile). The flow rate was 0.6 mL/min and the column temperature was 50 °C. This method began with a 1–3 min equilibration in 5 % solvent B followed by a subsequent 20 min gradient to 95 % solvent B. Full MS mode was used over the scan range of 100–1200 m/z and in individual negative acquisitions to acquire the data. CBGA and its isomers were detected at m/z 359.223 in negative mode.

The quantitative analysis of product formation was performed by using a Xevo TQ-S triple quadrupole tandem mass spectrometer (Waters MS Technologies) connected to an Acquity UPLC system (H-Class; Waters). OA and CBGA separation were performed on a BEH C18 (1.7µm, 2.1 x 50 mm, Waters) column using a mobile phase of A (0.05 % formic acid in water) and B (0.05 % formic acid in methanol). The gradient started from 70% B increased incrementally to 98% B in 0.7 minute, with a hold at 98%B for another 0.7 minute. This was followed by a 0.1 min gradient back to 70% B, with a final equilibration of 0.5 minute for the next injection.

Olivetol and CBG separation was performed on a BEH C8 column (1.7µm, 2.1 x 50mm, Waters) using mobile phase A (water, 0.1% NH_4_OH) and B (methanol, 0.1% NH_4_OH). The initial gradient program is 40% B, which then increased to 98% B over 3.5 minutes and held at 98% B for 0.5 minute. This was followed by an 0.2 min gradient to 40% B and a final 0.8 min equilibration at 40% B. The flow rate was 0.6 mL/min and the column temperature was 45 °C. The multiple reaction monitoring (MRM) transition used for quantification of the compounds are as follows: OA 223.01 > 179.12; CBGA 359.20 > 341.20; olivetol 181.09 > 111.14 and CBG 317.33 > 193.23.

Linalool quantitation was performed by GCMS using an Agilent Technologies 7890B GC equipped with a 5977A MSD detector. Product(s) (1 μL) were separated using a DB-WAX column (30 m x 0.32 mm i.d., 0.25 µM film thickness, Agilent Technologies) using the running conditions described previously (1). Compound identification was performed using the reference spectra found in the NIST library of MS spectra and fragmentation patterns. Linalool concentrations were determined by comparing peak areas to a standard curve generated from authentic standards run under identical conditions.

**Supplementary Results and Discussion**

**Results S1. Expression of *C. sativa* prenyl transferase CsPT1**

The N-terminally His_6_-tagged recombinant aromatic prenyl transferase (CsPT1) from *C. sativa* was transformed into aromatic prenyl transferase (APT) from *C. sativa*. Expression trials were performed to determine if significant soluble active protein could be generated. Unfortunately, a visible drop in culture optical density post induction was seen, suggesting expression of CsPT1 was toxic to the cells. Cultures were screened by monitoring the cell pellet (insoluble inclusion bodies), supernatant (soluble protein) and supernatant fractions after ultracentrifugation to separate out the membrane fraction. Both SDS PAGE and an anti-His Western blot were performed to look for the presence of an obvious 44.5 kDa CsPT1 band (**Supplementary Figure S2**). The SDS PAGE gel showed no obvious overexpression band around 44 kDa, and there was no evidence of a full length CsPT1 protein, only a smaller band around 25 kDa. This suggests there may be some truncated protein present, possibly due to proteolytic activity within *E. coli*. There was no evidence of a membrane-associated band, even though CsPT1 is known to contain membrane anchors. Therefore, gene homologues from bacteria were sought to try to find a suitable soluble prenyl transfer that expresses well in *E. coli*.

**Results S2. Generation of additional variants of AtaPT**

A homologous bacterial aromatic prenyltransferase NphB from *Streptomyces* sp. strain CL190 (3) is also known to prenylate OA to produce CBGA (4). Prior engineering studies demonstrated a significant reduction in *K_m_* towards OA with double variants Y288A/G286S, Y288V/A232S and Y288A/A232S. A variety of single and double variants of AtaPT were generated at the equivalent residues (Y344S, Y413S and E91Q) to see if further improvements in *K_m_* for OA could be achieved. However, none of these variants proved useful for CBGA production due to issues with soluble protein expression (**Supplementary Figure S7**) and product specificity (**Supplementary Figure S8**). AtaPT variants Y413S, E91Q/Y413S and E91Q/344S/Y413S showed only inclusion body formation on SDS PAGE, with the latter two proteins also having undergone proteolysis (15 kDa band). Soluble protein was detected for variants Y344S, Y344S/Y413S and E91Q/Y344S, but at a lower concentration than WT enzyme extracts. LC-MS analysis of reactions with purified enzymes showed a shift in product specificity towards a different isomer of CBGA at low titres with all variants containing Y344S and Y413S (**Supplementary Figure S8**). Therefore, AtaPT variant E91Q was taken forward for in vivo CBGA production studies due to a reduction in the production of by-products.

**Results S3. *In vivo* biotransformations for CBGA production**

Whole cell biotransformations of *E. coli* to make CBGA (and CBG) were trialled by co-expressing a plasmid containing TKS, OAC and AtaPT E91Q (pBbB2c-CBGA). Unfortunately, the only product detected was OA (5-10 mg/mL; **Supplementary Figure S9**), even though prior cell lysate assays (**Section 3.3**; **Figure 2b**) showed each of the three recombinant enzymes were being functionally expressed. Therefore, there was a need to identify the bottlenecks in the system and perform optimisation studies to allow metabolic flux through to CBGA (or CBG).

The accumulation of OA suggests a bottleneck exists in the final AtaPT catalysed step to CBGA, such as the availability of the GPP substrate. We added GPPS to the pBbB2c-CBGA construct to upregulate GPP production and generated a library of twelve genetic circuits with a selection of inducible (5) and constitutive (6) promoters positioned at different points throughout the pathway (**Supplementary Figure S9a**). This refactoring of regulatory genetic elements is often performed to achieve maximal titres of the desired product as imbalances in gene expression can cause the accumulation of potentially toxic intermediates and excessive metabolic burden, resulting in bottlenecks of metabolite production. Altering promoters gives transcriptional control of gene expression, allowing fine tuning of metabolic flux through a recombinant pathway (7).

An *in vivo* screen of this library of twelve regulatory variants generated OA at up to double the titre of the original pBbB2c-CBGA construct (**Supplementary Figure S9b**), however no CBGA was detected. Given the persistence of OA accumulation within the library screen, this suggests the overexpression of GPPS alone is not sufficient to upregulate GPP production to allow significant CBGA production to occur.

**Results S4. *In* vivo screen incorporating an MVA pathway for CBGA production**

GPP is generated in *E. coli* by the native MEP pathway and acts as a precursor to terpene production. Within *C. sativa*, both the MEP and MVA pathways generate the precursors for GPP production. Numerous studies have shown that incorporating a heterologous MVA pathway into *E. coli* engineered for GPP upregulation in terpenoid production significantly increases the titres of the target compound (1; 7-9).

To ensure sufficient GPP supply for prenylation of OA (and olivetol) in whole cells, we generated a modified heterologous MVA pathway plasmid (pMVA_NR2) containing a constitutive promoter J23116 based on the more genetically stable variant described previously (9). Unfortunately, GPP quantitation in cell lysates is notoriously difficult to achieve as it breaks down rapidly. The usual way to determine whether strategies to increase GPP titres have been successful is to couple it with a terpene cyclase and determine the titres of the resultant monoterpenoid. In our study we tested this construct for its ability to increase GPP production by co-expressing it with a linalool production plasmid (pBbB2a-GPPS-LinS). Whole cell linalool production studies showed that linalool titres were between 2.4-3.5-fold higher, dependent on the carbon source, when incorporating the modified pMVA NR2 plasmid (8.2 mg/L with glycerol) over the original pMVA plasmid (**Supplementary Figure S10a**). Further statistical analyses (unequal variance unpaired *t*-test) showed the differences were significant with a 99% confidence when using glycerol as a carbon source (P=0.0011), but only 95% confidence when using glucose (P = 0.0176). These titres are low compared to other studies with pMVA plasmids containing the J23116 promoter (150-250 mg/L; (9)), however linalool production from a two-plasmid system has been shown previously to have lower titres than the optimised one-plasmid system (1). This may be due to the metabolic burden imposed on the cells due to cultivation in the presence of two antibiotics to maintain both plasmids.

After successfully demonstrating linalool production, the pMVA_NR2 plasmid, was co-expressed with pBbB2c-CBGA. Unfortunately, no CBGA production was detected. An average titre of 20.2 ± 1.8 μg/L of OA was observed for this expression system. It was believed that this modest concentration of OA may have been the cause of lack of turnover into detectable CBGA, therefore routes to increase this titre were investigated.

**Results S5. Upregulating intracellular malonyl-CoA levels**

Another substrate that could be limiting CBGA production is the starting substrate malonyl-CoA. This is similar to the situation described for *E. coli* engineered for OA production that required supplemental feeding of hexanoate, the precursor to hexanoyl-CoA, to increase titres to 80 mg/L (10). One way to increase intracellular malonyl-CoA levels is to supplement cultures with the antibiotic cerulenin (11), which inhibits the 3-oxoacyl-acyl carrier protein synthases FabB and FabF (12). This in turn reduces the consumption of malonyl-CoA by central metabolism, enabling more to be available to recombinant pathways. However, cerulenin addition can also lead to *E. coli* growth inhibition, which would impact on final secondary product titres (13; 14). An alternative route to cerulenin supplementation is to utilise a knockout *E. coli* strain that is deficient in one or both genes. We tested both the wild-type *E. coli* NEB 5α and the DH5α Δ*fabF* knockout variant for olivetol production by incorporating the TKS gene, with the latter strain having a reduced rate of malonyl-CoA consumption by competing pathways (15; 16). The highest titres of olivetol were seen with the *E. coli* Δ*fabF* knockout expressing TKS when the post induction temperature was 25-30 °C (104-106 μg/L; **Supplementary** **Figure S10b**). These titres were around 4.4-fold higher than olivetol production in NEB 5α (23-246 μg/L). As no direct comparative quantitation of malonyl-CoA in wild-type and the DH5α Δ*fabF* knockout was performed, we cannot conclude decisively that the increased olivetol titres was due to an increase in malonyl-CoA production in the Δ*fabF* knockout. However, as this strain led to increased olivetol titres, we took this forward for use in subsequent experiments.

**Supplementary Figures**


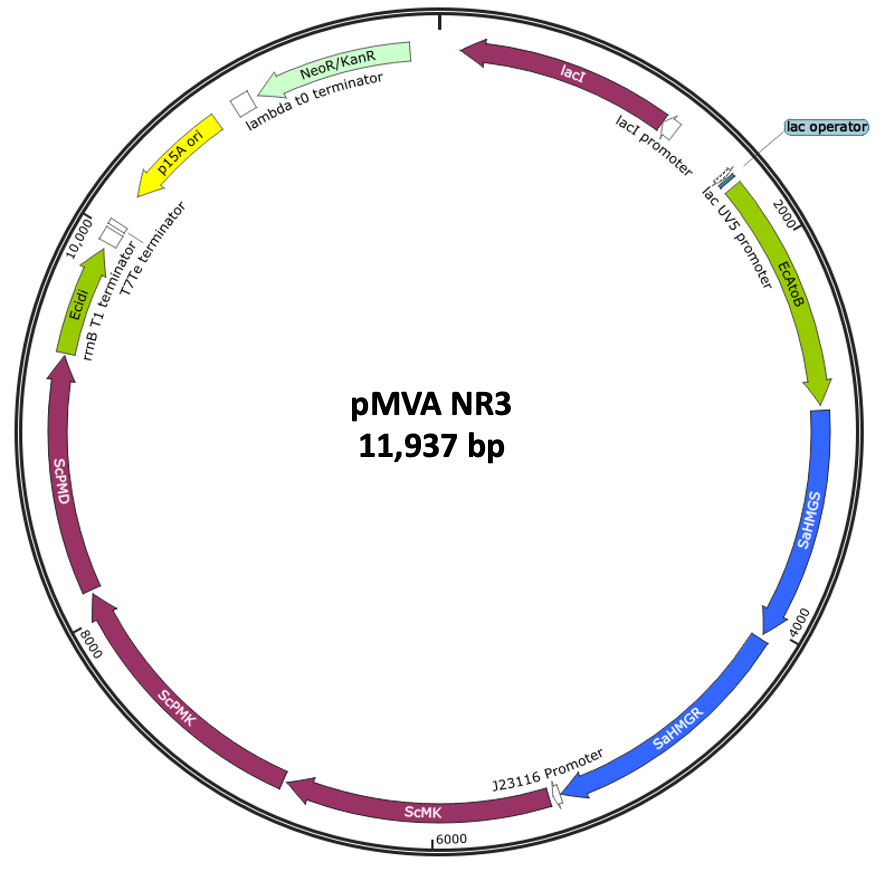


**Figure S1. Plasmid map of the pMVANR2 construct.** Genes: AgGPPS = geranyl pyrophosphate synthase; EcAtoB = acetoacetyl-CoA thiolase; EcIDI = isopentenyl diphosphate isomerase; SaHMGR = HMG-CoA reductase; SaHMGS = HMG-CoA synthase; ScMK= mevalonate kinase; ScPMD = phosphomevalonate decarboxylase and ScPMK = phosphomevalonate kinase.


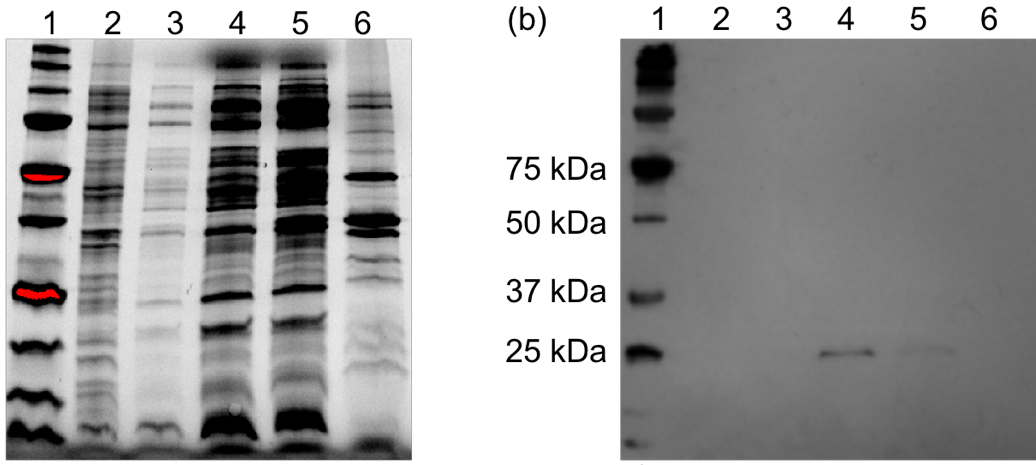


B

A)

B)


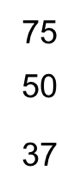

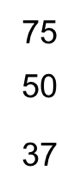


kDa

kDa

Figure S2. SDS PAGE and Western blot analysis of the expression of native aromatic prenyl transferase (APT) from *C. sativa* in *Escherichia coli*. (A) SDS-PAGE and (B) anti-His tag monoclonal antibody (mouse) Western blot analysis of culture supernatants for the presence of APT. Cultures (1 L) of *E. coli* BL21(DE3) containing recombinant N-His_6_-APT were harvested, lysed and the supernatant recovered by centrifugation 40,000 *g*. Further clarification was performed by ultracentrifugation 144794 *g*. Western blots were performed using the iBind western system according to the manufacturer’s instructions. For each gel, the lanes are: 1 = molecular mass ladder; 2 = lysate; 3 = high speed supernatant; 4 = ultra-speed supernatant; 5 = ultra-speed lower supernatant and 6 = ultra-speed lipid pellet.


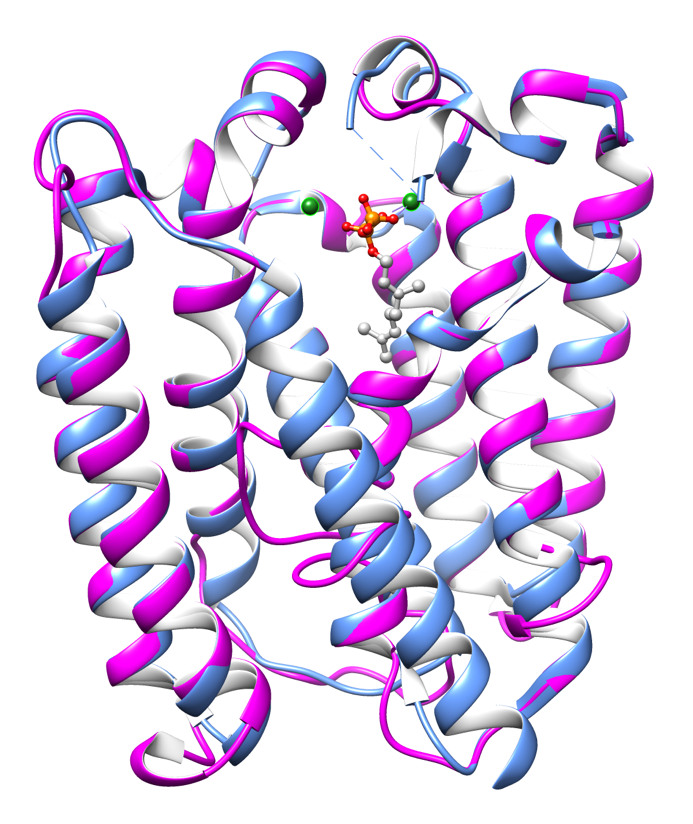


Figure S3. SWISS-MODEL structure of CsPT1 superimposed on the crystal structure of UBiA. The *Cannabis sativa* prenyl transferase 1 (CsPT1) SWISS-MODEL was generated using the crystal structure of UBiA from *Archaeoglobus fulgidus* (17) as the template (PDB: 4TQ3). The model of CsPT1 (magenta) and crystal structure of UBiA (blue) are shown in a cartoon representation. The bound GPP, Mg^2+^ ions and nearby residues of UBiA are shown as atom ball and sticks. The Figure was generated in Chimera (18).


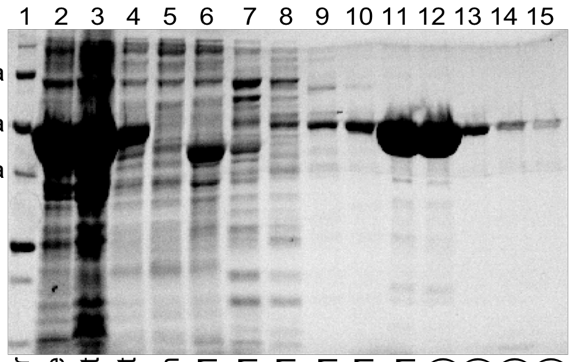

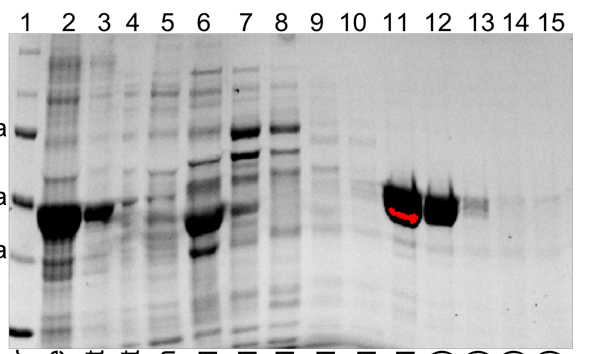

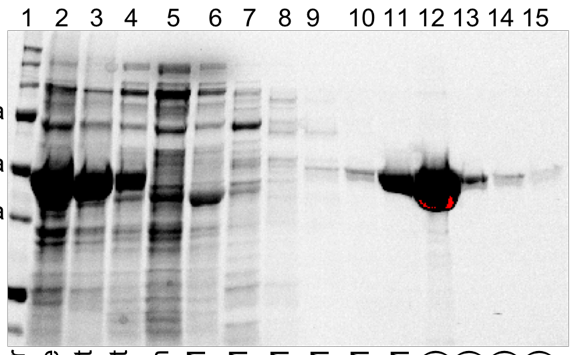

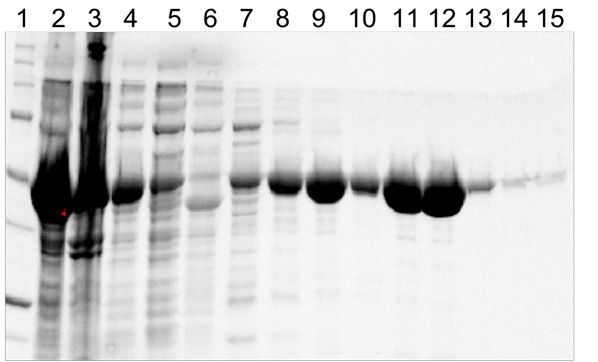


A

C

D


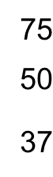


kDa


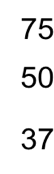


kDa


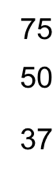


kDa

B)

A)

D)

C)


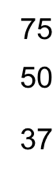


kDa

Figure S4. SDS-PAGE analysis of IMAC purifications of WT *Aspergillus terreus* aromatic prenyl transferase (AtaPT) and variants E91A, E91D and E91Q. AtaPT variants (47.5 kDa) were expressed in ArcticExpress (DE3) cells (1 L). Clarified cell extracts (20 ml) were run through a 5 mL His Trap FF column, washed and eluted with a step gradient of increasing imidazole concentration. SDS PAGE analysis of eluent fractions of AtaPT A) wild-type and variants B) E91A, C) E91D and D) E91Q. For each gel, the lanes were: 1 = molecular mass marker; 2 = lysate; 3 = cell pellet; 4 = clarified supernatant; 5 = unbound fraction; 6 = wash (no imidazole); 7 = 10 mM imidazole eluate; 8 = 20 mM imidazole eluate; 9 = 30 mM imidazole eluate; 10 = 400 mM imidazole eluate; 11 = 50 mM imidazole eluate; 12-13 = 200 mM imidazole eluate and 14-15 = 500 mM imidazole eluate.


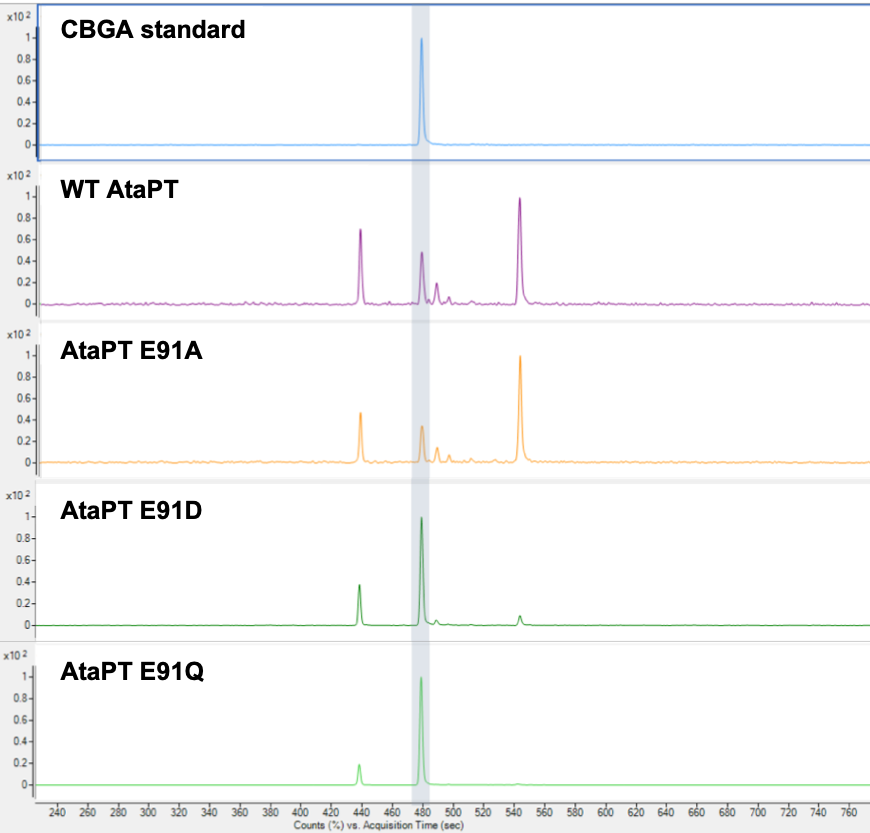


**Figure S5.** **Product profiles of biotransformations with wild-type AtaPT and variants E91A, E91D and E91Q.** Reactions were performed in the presence of olivetolic acid (OA) and geranyl pyrophosphate (GPP). The extracted samples along with a cannabegerolic acid (CBGA) standard were ran on a 6560 Ion Mobility Q-TOF LC-MS.


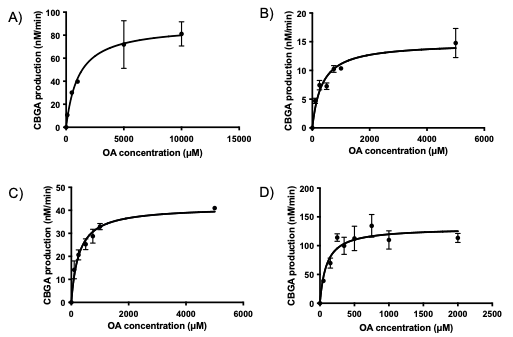


**Figure S6. Apparent Michaelis Menten plots of A) wild-type and AtaPT variants B) E91A, C) E91D and D) E91Q with OA.** Reactions (200 μL) were composed of AtaPT (1-10 μM), GPP (1 mM) and varying OA concentrations (0-10 mM) in 25 mM Tris-HCl pH 8.0, 150 mM NaCl and 5% glycerol. The reactions were incubated for 20 min at 25 °C and extracted with ethyl acetate. Analysis was performed using a Xevo TQ-S triple quadrupole tandem mass spectrometer. These data yield only approximate kinetic constant values as the low specific activity of the enzymes require relatively high enzyme concentrations to be used, which does not satisfy the Michaelis Menten equation assumption that the enzyme concentration is insignificant compared to the substrate(s) concentration ([E] <<< [S]) (19).


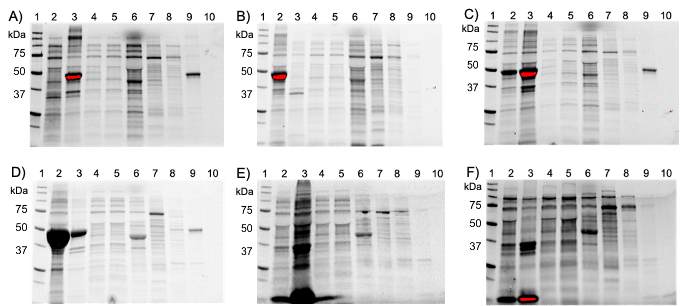


**Figure S7. SDS-PAGE analysis of IMAC purifications of variants of AtaPT.** AtaPT variants (47.5 kDa) were expressed in ArcticExpress (DE3) cells (1 L). Clarified cell extracts (20 ml) were run through a 5 mL His Trap FF column, washed and eluted with a step gradient of increasing imidazole concentration. SDS PAGE analysis of eluent fractions of AtaPT variants A) Y344S, B) Y413S, C) Y344S/Y413S, D) E91Q/Y344S, E) E91Q/Y413S and F) E91Q/Y344S/Y413S. For each gel, the lanes are as follows: lane 1 = molecular mass marker; 2 = lysate; 3 = pellet; 4 = supernatant; 5 = unbound fraction; 6 = wash (no imidazole); 7 = 20 mM imidazole eluate; 8 = 50 mM imidazole eluate; 9 = 200 mM imidazole eluate and 10 = 500 mM imidazole eluate.


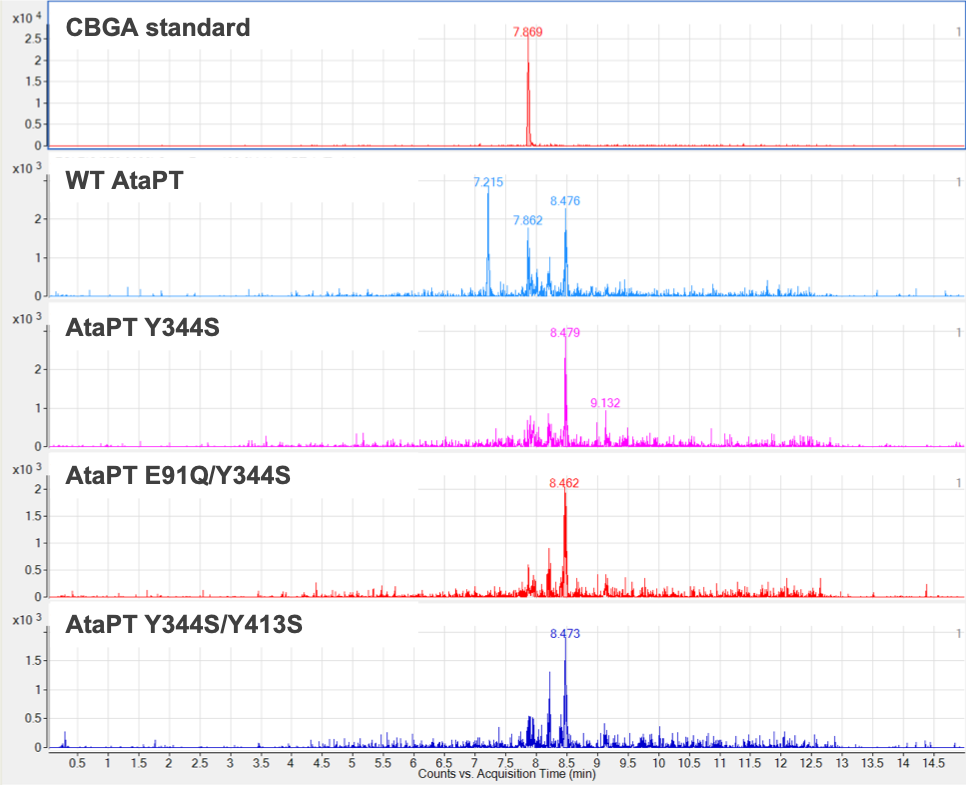


**Figure S8. Product profiles of biotransformations with wild-type AtaPT and variants Y344S, E91A/Y344S and Y344S/Y413S.** Reactions were performed in the presence of OA and GPP. The extracted samples along with a CBGA standard were run on a 6560 Ion Mobility Q-TOF LC-MS. The retention times of CBGA was 7.86 minutes, while the others are of unknown CBGA isomers.


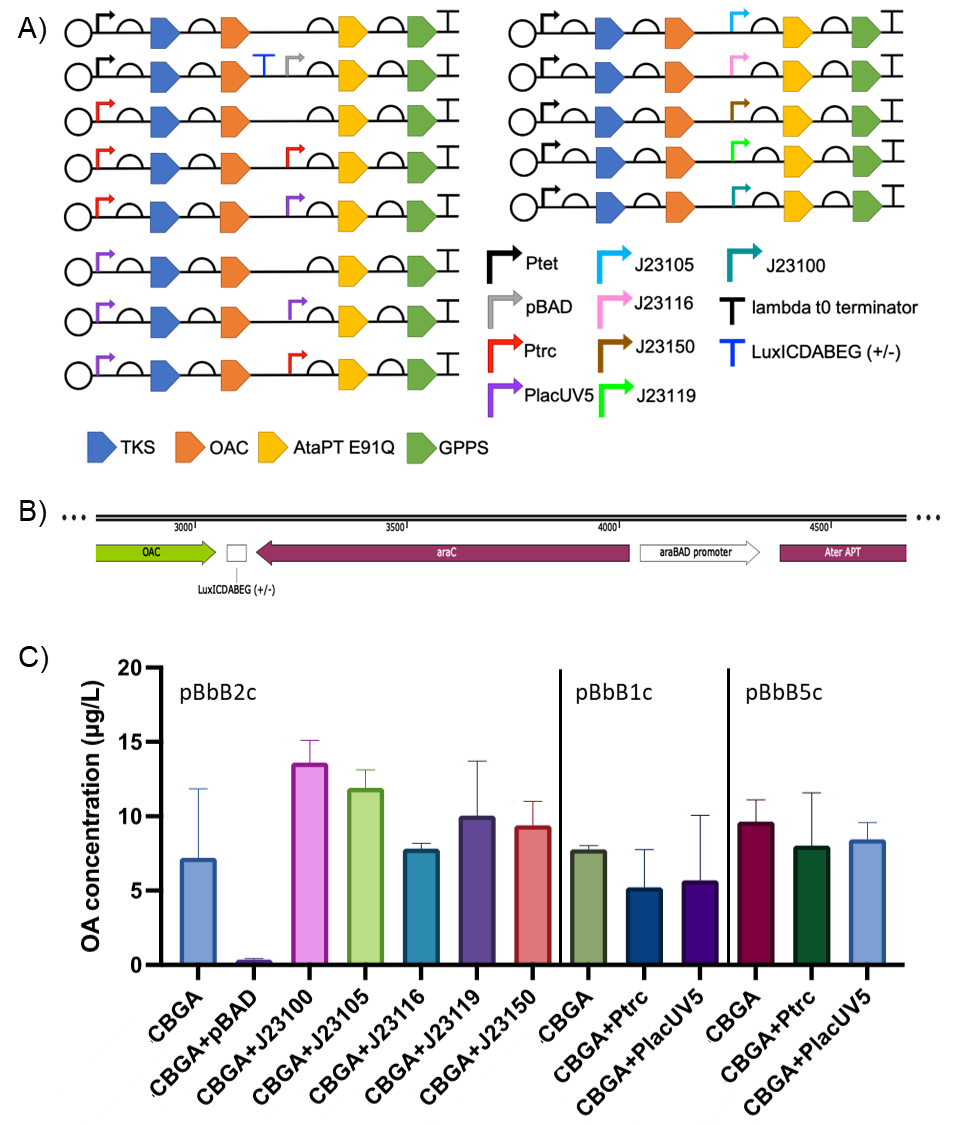


**Figure S9. Pathways to produce OA and CBGA in *E. coli***. A) Schematic of the OA/CBGA production cassettes varying the promoter and plasmid backbone. The library of promoters is denoted by the coloured arrows. For constructs where the promoter upstream of TKS is *P_tet_*, *P_trc_* and *P_lacUV5_* the backbone vector was pBbB2c, pBbB1c and pBbB5c, respectively (20). B) Plasmid map of LuxICDABEG (+/-) location in pBbB2c-CBGA. This figure was created using SnapGene. C) OA production in *E. coli* using the promoter screen panel of TKS-OAC-AtaPT E91Q-GPPS constructs. 3 mL of TB media + 0.4 % glycerol containing appropriate antibiotic were inoculated with 1 colony. For each construct this was performed in biological triplicate. The cultures were incubated at 37 °C until turbidity was visible and expression was induced with appropriate inducer. The cultures were incubated for a further 48 hours at 30 °C


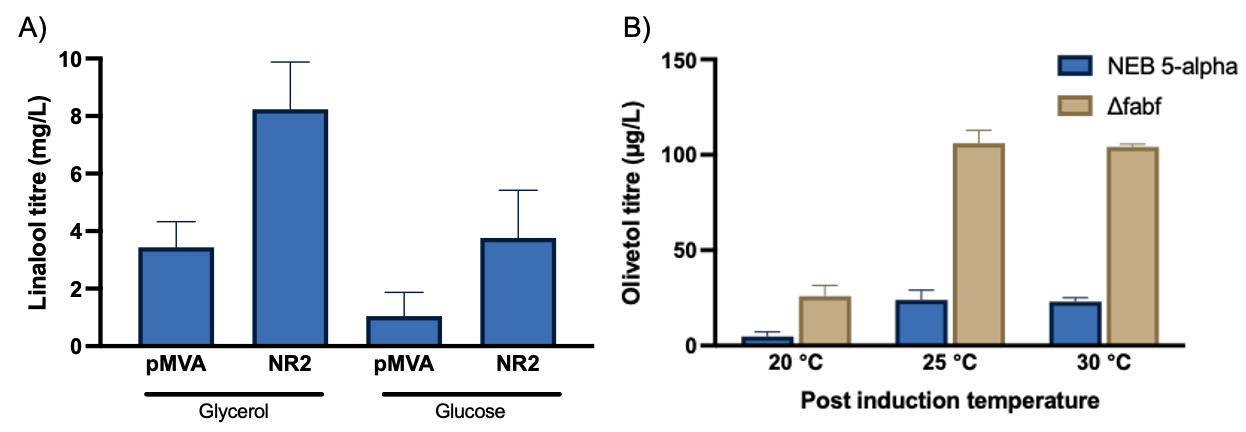


**Figure S10. Screening of multiple recombinant constructs and reaction conditions for GPP precursor upregulation and CBG production.** (A) Linalool production in *E. coli* containing pMVA or pMVA_NR2 as an indicator of GPP upregulation. 3 ml TB media cultures were grown at 37 °C until turbidity was visible and then induced and incubated at 30 °C for 72 hours. (B) The screening of the *E. coli* strains and post-induction temperatures for optimal olivetol production. 3 ml TB media + 0.4% glycerol cultures were grown at 37 °C for 8 hours and then induced and incubated at the specified temperature for 72 hours.

Supplementary Tables

| **Table S1.** Primers used for cloning and mutagenesis. | | |
| --- | --- | --- |
| **Primer** | **Sequence (5’-3’)** | **Use** |
| E91 complementary | CAAGACCATTTGTCGTCTGGGTCTGCAGG | E91A mutagenesis |
| E91A primer | CTTTTATCAATTTTCACGCTAATTGCCAGCGGATGACCACC |  |
| E91 complementary | CAAGACCATTTGTCGTCTGGGTCTGCAGG | E91D mutagenesis |
| E91D primer | CTTTTATCAATTTTCACGCTAATATCCAGCGGATGACCACC |  |
| E91 complementary | CAAGACCATTTGTCGTCTGGGTCTGCAGG | E91Q mutagenesis |
| E91Q primer | CTTTTATCAATTTTCACGCTAATTTGCAGCGGATGACCACC |  |
| PBbB2c-CBGA linearising FWD  PBbB2c-Linearising REV | ACGGAAGGAGCATGGCGATG  GAATTCTTTTCTTTATTTACGCGGGGTATAGTC | pBbB2c-OA plasmid linearisation |
| pBbB2c-CBG linearising FWD | ATGTATATCTCCTTCTTAAAGTTAAACAAAATTATTTCTAG | pBbB2c-OA plasmid linearisation |
| pBbB2c-CBG linearising REV | AGAAAAGAATTCACGATCTTAAGTAGG |  |
| AterAPT in-fusion FWD | GAAGGAGATATACATATGCTGCCTCCGAGCGATAGC | Generation of AterAPT fragment for insertion into pBbB2c-CBG |
| AterAPT in-fusion REV | CGTGAATTCTTTTCTTTAAACGCGTGCCACATTACCTGC |  |
| pMVA NR3 FWD | CTAGCTCAGTCCTAGGGACTATGCTAGCGGATCTAGGAGGAAATAACCATGTCTCTGCCATTCC | Insertion of promoter J23116 to make pMVA NR2 by InFusion cloning |
| pMVA NR3 REV | TAGTCCCTAGGACTGAGCTAGCTGTCAAGATCCTTATTGTTGTCTAATTTCTTGTAAAATATGTTCCGC |  |

| **Table S2.** Primers used for cloning and mutagenesis of libraries of clones. | | |
| --- | --- | --- |
| **Primer** | **Sequence (5’-3’)** | **Use** |
| pBAD FWD | TAAAGAAAAGAATTCGACGTCTTATGACAACTTGACG | pBbB2c-CBGA-pBAD cloning primers |
| pBAD REV | CCATGCTCCTTCCGTGATCTTTTGAATTCCCAAAAAAACGGGTATGG |  |
| pBbB2c-CBGA linearising FWD | ACGGAAGGAGCATGGCGATG |  |
| pBbB2c-CBGA linearising REV | GAATTCTTTTCTTTATTTACGCGGGGTATAGTC |  |
| LuxICDABEG (+/-) FWD | TTAATCCGGCTTTTTTATTATTTAGGAAGCTGAGTTGGTTCGACGTCTTATGACAACTTGACG |  |
| LuxICDABEG (+/-) REV | TAATCTGGCTTTTTATATTCTCTTTCGGGCTTTGTTAGTTCTTTTCTTTATTTACGCGGGGTATAGTC |  |
| Ptrc FWD | GTATAATGTGTGGAATTGTGAGCGGATAACAAGAAGCTGAGTTGGAAAAGAATTCACGGAAGGAGCATGG | Primers used to introduce *P_trc_* and *P_lacUV5_* promoters in the CBGA gene cassette |
| Ptrc REV | GAGCCGGATGATTAATTGTCAAAGTCAACAAGCTGCGCTTTATTTACGCGGGGTATAGTCAAAAATCAGG |  |
| PlacUV5 FWD | CGTATAATGTGTGGAATTGTGAGCGGATAACAAGAAGCTGAGTTGGAAAAGAATTCACGGAAGGAGCATGG |  |
| PlacUV5 REV | AGCCGGAAGCATAAAGTGTAAAAGTCAACAAGCTGCGCTTTATTTACGCGGGGTATAGTCAAAAATCAGG |  |
| CBGA cassette amplify FWD | AAGGAGATATACATATGAACCACCTAAGAGCAGAAGG ACTCGAGTTTGGATCCCTTAATTCTG |  |
| CBGA cassette amplify REV | ACTCGAGTTTGGATCCCTTAATTCTG |  |
| J23100 FWD | GTCCTAGGTACAGTGCTAGCCGCAGAGAATTCACGGAAGGAGCATGG | Cloning of constitutive primers into the library |
| J23100 REV | TGAGCTAGCCGTCAAAGTCAACAAGTTTCTTTATTTACGCGGGGTATAGTC |  |
| J23105 FWD | GTCCTAGGTACTATGCTAGCCGCAGAGAATTCACGGAAGGAGCATGG |  |
| J23105 REV | TGAGCTAGCCGTAAAAGTCAACAAGTTTCTTTATTTACGCGGGGTATAGTC |  |
| J23116 FWD | GTCCTAGGGACTATGCTAGCCGCAGAGAATTCACGGAAGGAGCATGG |  |
| J23116 REV | TGAGCTAGCTGTCAAAGTCAACAAGTTTCTTTATTTACGCGGGGTATAGTC |  |
| J23119 FWD | GTCCTAGGTATAATGCTAGCCGCAGAGAATTCACGGAAGGAGCATGG |  |
| J23119 REV | TGAGCTAGCTGTCAAAGTCAACAAGTTTCTTTATTTACGCGGGGTATAGTC |  |
| J23150 FWD | GTCCTAGGTATTATGCTAGCCGCAGAGAATTCACGGAAGGAGCATGG |  |
| J23150 REV | TGAGCTAGCCGTAAAAGTCAACAAGTTTCTTTATTTACGCGGGGTATAGTC |  |

| **Table S3.** In vivo production of cannabinoids in *E. coli* expressing pBb-CBGA and pMVA NR2. | |
| --- | --- |
| Clone / Replicates | OA (μg/L) |
| *pBb-CBGA-GPPS + pMVA NR2 in E. coli NEB5α* | |
| Colony 1 | 19.5 |
| Colony 2  Colony 3 | 19.1  19.3 |
| Colony 4 | 22.8 |
| *pBb-CBGA-GPPS + pMVA NR2 in E. coli* DH5α Δ*fabF* | |
| Colony 1  Colony 2 | 93.0  95.1 |
| Colony 3 | 71.7 |
| Colony 4 | 61.1 |
| 250 μL of the starter cultures were inoculated into 25 mL TB media + 0.4 % glycerol containing appropriate antibiotic. Cultures were incubated at 37 °C until an OD at 600 nm of 0.6-0.8 was reached. The incubation temperature was reduced to 25 °C and expression of both pathways was induced by adding 0.1 mM IPTG and 200 nM aTet. No olivetol, CBG or CBGA was detected in any sample. | |

**Supplementary Plasmid Sequences**

>pBbB2a-GPPS-LinS (Genbank: OQ725931)

AAATAATTCGATAGCTTGTCGTAATAATGGCGGCATACTATCAGTAGTAGGTGTTTCCCTTTCTTCTTTAGCGACTTGATGCTCTTGATCTTCCAATACGCAACCTAAAGTAAAATGCCCCACAGCGCTGAGTGCATATAATGCATTCTCTAGTGAAAAACCTTGTTGGCATAAAAAGGCTAATTGATTTTCGAGAGTTTCATACTGTTTTTCTGTAGGCCGTGTACCTAAATGTACTTTTGCTCCATCGCGATGACTTAGTAAAGCACATCTAAAACTTTTAGCGTTATTACGTAAAAAATCTTGCCAGCTTTCCCCTTCTAAAGGGCAAAAGTGAGTATGGTGCCTATCTAACATCTCAATGGCTAAGGCGTCGAGCAAAGCCCGCTTATTTTTTACATGCCAATACAATGTAGGCTGCTCTACACCTAGCTTCTGGGCGAGTTTACGGGTTGTTAAACCTTCGATTCCGACCTCATTAAGCAGCTCTAATGCGCTGTTAATCACTTTACTTTTATCTAATCTAGACATCATTAATTCCTAATTTTTGTTGACACTCTATCGTTGATAGAGTTATTTTACCACTCCCTATCAGTGATAGAGAAAAGAATTCACGATCTTAAGTTCAAGAGGGTAATAATGGAGTTCGACTTCAACAAATACATGGACAGCAAAGCCATGACCGTTAATGAAGCACTGAATAAAGCAATTCCGCTGCGTTATCCGCAGAAAATCTATGAAAGCATGCGTTATAGCCTGCTGGCAGGCGGTAAACGTGTTCGTCCGGTTCTGTGTATTGCAGCATGTGAACTGGTTGGTGGCACCGAAGAACTGGCAATTCCGACCGCATGTGCAATTGAAATGATTCATACCATGAGCCTGATGCATGATGATCTGCCGTGTATTGATAATGATGACCTGCGTCGTGGTAAACCGACCAATCATAAAATCTTTGGTGAAGATACCGCAGTGACCGCAGGTAATGCACTGCATAGTTATGCATTTGAACATATTGCAGTGAGCACCAGCAAAACCGTTGGTGCAGATCGTATTCTGCGTATGGTTAGCGAACTGGGTCGTGCAACCGGTAGCGAAGGTGTTATGGGTGGTCAGATGGTTGATATTGCAAGTGAAGGTGATCCGAGCATTGATCTGCAGACCCTGGAATGGATTCATATTCATAAAACCGCAATGCTGCTGGAATGTAGCGTTGTTTGTGGTGCAATTATTGGTGGTGCAAGCGAAATTGTTATTGAACGTGCCCGTCGTTATGCACGTTGTGTTGGTCTGCTGTTTCAGGTTGTTGATGATATTCTGGATGTGACCAAAAGCAGTGATGAACTGGGCAAAACCGCAGGCAAAGATCTGATTAGCGATAAAGCAACCTATCCGAAACTGATGGGTCTGGAAAAAGCCAAAGAATTTTCAGATGAACTGCTGAATCGTGCCAAAGGTGAACTGAGCTGTTTTGATCCGGTTAAAGCAGCACCGCTGCTGGGTCTGGCAGATTATGTTGCATTTCGTCAGAATTAAGCTTAATAATTTTGATTTAACTTTAAGAAGGAGATATACCATGAAACATCACCATCACCATCACCCCATGAGCGATTACGACATCCCCACTACTGAGAATCTTTATTTTCAGGGCATGCAAGAATTTGAATTTGCAGTTCCGGCACCGAGCCGTGTTAGTCCGGATCTGGCACGTGCGCGTGCACGTCATCTGGATTGGGTTCATGCAATGGATCTGGTTCGTGGTGAAGAGGCACGTCGTCGTTATGAATTTAGCTGTGTTGCAGATATTGGTGCCTATGGTTATCCGCATGCAACCGGTGCAGATCTGGATCTGTGTGTTGATGTTCTGGGTTGGACCTTTCTGTTTGATGATCAGTTTGATGCCGGTGATGGTCGTGAACGTGATGCACTGGCAGTTTGTGCAGAACTGACCGATCTGCTGTGGAAAGGTACAGCAGCAACCGCAGCAAGCCCTCCGATTGTTGTTGCATTTAGCGATTGTTGGGAACGTATGCGTGCAGGTATGAGTGATGCATGGCGTCGTCGTACCGTTCATGAATGGGTTGATTATCTGGCAGGTTGGCCGACCAAACTGGCAGATCGTGCACATGGTGCCGTTCTGGATCCGGCAGCACATCTGCGTGCTCGCCATCGTACCATTTGTTGTCGTCCGCTGTTTGCACTGGCCGAACGTGTTGGTGGTTATGAAGTTCCGCGTCGTGCATGGCATAGCAGCCGTCTGGATGGTATGCGTTTTACCACCAGTGATGCAGTTATTGGTATGAATGAACTGCACAGCTTTGAAAAAGATCGTGCCCAGGGTCATGCAAATCTGGTTCTGAGCCTGGTTCATCATGGTGGTCTGACCGGTCCGGAAGCAGTTACCCGTGTTTGTGATCTGGTGCAGGGTAGCATTGAAAGTTTTCTGCGTCTGCGTAGCGGTCTGCCTGAACTGGGTCGTGCACTGGGTGTTGAAGGTGCAGTGCTGGATCGTTATGCAGATGCACTGAGCGCATTTTGTCGTGGTTATCATGATTGGGGTCGTGGTGCAAGCCGTTATACCACACGTGATCATCCGGGTGATCTGGGTCTGGAAAATCTGGTGGCACGTAGCAGCGGTTAATAACTCGAGCACCACCACCACCACCACTGAGATCCGGCTGCTAACAAAGGGATCCTAAGGATCTCCAGGCATCAAATAAAACGAAAGGCTCAGTCGAAAGACTGGGCCTTTCGTTTTATCTGTTGTTTGTCGGTGAACGCTCTCTACTAGAGTCACACTGGCTCACCTTCGGGTGGGCCTTTCTGCGTTTATACCTAGGCTACAGCCGATAGTCTGGAACAGCGCACTTACGGGTTGCTGCGCAACCCAAGTGCTACCGGCGCGGCAGCGTGACCCGTGTCGGCGGCTCCAACGGCTCGCCATCGTCCAGAAAACACGGCTCATCGGGCATCGGCAGGCGCTGCTGCCCGCGCCGTTCCCATTCCTCCGTTTCGGTCAAGGCTGGCAGGTCTGGTTCCATGCCCGGAATGCCGGGCTGGCTGGGCGGCTCCTCGCCGGGGCCGGTCGGTAGTTGCTGCTCGCCCGGATACAGGGTCGGGATGCGGCGCAGGTCGCCATGCCCCAACAGCGATTCGTCCTGGTCGTCGTGATCAACCACCACGGCGGCACTGAACACCGACAGGCGCAACTGGTCGCGGGGCTGGCCCCACGCCACGCGGTCATTGACCACGTAGGCCAACACGGTGCCGGGGCCGTTGAGCTTCACGACGGAGATCCAGCGCTCGGCCACCAAGTCCTTGACTGCGTATTGGACCGTCCGCAAAGAACGTCCGATGAGCTTGGAAAGTGTCTTCTGGCTGACCACCACGGCGTTCTGGTGGCCCATCTGCGCCACGAGGTGATGCAGCAGCATTGCCGCCGTGGGTTTCCTCGCAATAAGCCCGGCCCACGCCTCATGCGCTTTGCGTTCCGTTTGCACCCAGTGACCGGGCTTGTTCTTGGCTTGAATGCCGATTTCTCTGGACTGCGTGGCCATGCTTATCTCCATGCGGTAGGGGTGCCGCACGGTTGCGGCACCATGCGCAATCAGCTGCAACTTTTCGGCAGCGCGACAACAATTATGCGTTGCGTAAAAGTGGCAGTCAATTACAGATTTTCTTTAACCTACGCAATGAGCTATTGCGGGGGGTGCCGCAATGAGCTGTTGCGTACCCCCCTTTTTTAAGTTGTTGATTTTTAAGTCTTTCGCATTTCGCCCTATATCTAGTTCTTTGGTGCCCAAAGAAGGGCACCCCTGCGGGGTTCCCCCACGCCTTCGGCGCGGCTCCCCCTCCGGCAAAAAGTGGCCCCTCCGGGGCTTGTTGATCGACTGCGCGGCCTTCGGCCTTGCCCAAGGTGGCGCTGCCCCCTTGGAACCCCCGCACTCGCCGCCGTGAGGCTCGGGGGGCAGGCGGGCGGGCTTCGCCCTTCGACTGCCCCCACTCGCATAGGCTTGGGTCGTTCCAGGCGCGTCAAGGCCAAGCCGCTGCGCGGTCGCTGCGCGAGCCTTGACCCGCCTTCCACTTGGTGTCCAACCGGCAAGCGAAGCGCGCAGGCCGCAGGCCGGAGGCACTAGTGCTTGGATTCTCACCAATAAAAAACGCCCGGCGGCAACCGAGCGTTCTGAACAAATCCAGATGGAGTTCTGAGGTCATTACTGGATCTATCAACAGGAGTCCAAGCGAGCTCGTAAACTTGGTCTGACAGTTACCAATGCTTAATCAGTGAGGCACCTATCTCAGCGATCTGTCTATTTCGTTCATCCATAGTTGCCTGACTCCCCGTCGTGTAGATAACTACGATACGGGAGGGCTTACCATCTGGCCCCAGTGCTGCAATGATACCGCGAGACCCACGCTCACCGGCTCCAGATTTATCAGCAATAAACCAGCCAGCCGGAAGGGCCGAGCGCAGAAGTGGTCCTGCAACTTTATCCGCCTCCATCCAGTCTATTAATTGTTGCCGGGAAGCTAGAGTAAGTAGTTCGCCAGTTAATAGTTTGCGCAACGTTGTTGCCATTGCTACAGGCATCGTGGTGTCACGCTCGTCGTTTGGTATGGCTTCATTCAGCTCCGGTTCCCAACGATCAAGGCGAGTTACATGATCCCCCATGTTGTGCAAAAAAGCGGTTAGCTCCTTCGGTCCTCCGATCGTTGTCAGAAGTAAGTTGGCCGCAGTGTTATCACTCATGGTTATGGCAGCACTGCATAATTCTCTTACTGTCATGCCATCCGTAAGATGCTTTTCTGTGACTGGTGAGTACTCAACCAAGTCATTCTGAGAATAGTGTATGCGGCGACCGAGTTGCTCTTGCCCGGCGTCAATACGGGATAATACCGCGCCACATAGCAGAACTTTAAAAGTGCTCATCATTGGAAAACGTTCTTCGGGGCGAAAACTCTCAAGGATCTTACCGCTGTTGAGATCCAGTTCGATGTAACCCACTCGTGCACCCAACTGATCTTCAGCATCTTTTACTTTCACCAGCGTTTCTGGGTGAGCAAAAACAGGAAGGCAAAATGCCGCAAAAAAGGGAATAAGGGCGACACGGAAATGTTGAATACTCATACTCTTCCTTTTTCAATATTATTGAAGCATTTATCAGGGTTATTGTCTCATGAGCGGATACATATTTGAATGTATTTAGAAAAATAAACAAATAGGGGTTCCGCGCACATTTCCCCGAAAAGTGCCACCTGACGTCTTAAGACCCACTTTCACATTTAAGTTGTTTTTCTAATCCGCATATGATCAATTCAAGGCCGAATAAGAAGGCTGGCTCTGCACCTTGGTGATC

>pBbB2c-OA (Genbank: OQ725932)

GACGTCTTAAGACCCACTTTCACATTTAAGTTGTTTTTCTAATCCGCATATGATCAATTCAAGGCCGAATAAGAAGGCTG

GCTCTGCACCTTGGTGATCAAATAATTCGATAGCTTGTCGTAATAATGGCGGCATACTATCAGTAGTAGGTGTTTCCCTT

TCTTCTTTAGCGACTTGATGCTCTTGATCTTCCAATACGCAACCTAAAGTAAAATGCCCCACAGCGCTGAGTGCATATAA

TGCATTCTCTAGTGAAAAACCTTGTTGGCATAAAAAGGCTAATTGATTTTCGAGAGTTTCATACTGTTTTTCTGTAGGCC

GTGTACCTAAATGTACTTTTGCTCCATCGCGATGACTTAGTAAAGCACATCTAAAACTTTTAGCGTTATTACGTAAAAAA

TCTTGCCAGCTTTCCCCTTCTAAAGGGCAAAAGTGAGTATGGTGCCTATCTAACATCTCAATGGCTAAGGCGTCGAGCAA

AGCCCGCTTATTTTTTACATGCCAATACAATGTAGGCTGCTCTACACCTAGCTTCTGGGCGAGTTTACGGGTTGTTAAAC

CTTCGATTCCGACCTCATTAAGCAGCTCTAATGCGCTGTTAATCACTTTACTTTTATCTAATCTAGACATCATTAATTCC

TAATTTTTGTTGACACTCTATCGTTGATAGAGTTATTTTACCACTCCCTATCAGTGATAGAGAAAAGAATTCAAAAGATC

TTTTAAGAAGCCTTAGGTCAATCCACAAAAGCGGCGCATAAGGAAAACTCCCTAGATTCGAGGACCAACAATGAACCACC

TAAGAGCAGAAGGCCCAGCCTCCGTGCTGGCCATTGGTACCGCCAATCCGGAAAACATTTTATTGCAGGACGAATTTCCT

GATTATTATTTTCGTGTTACCAAATCGGAACACATGACCCAGCTGAAAGAAAAATTTCGCAAAATTTGTGACAAAAGTAT

GATACGGAAACGAAATTGTTTTCTGAATGAGGAACACTTGAAGCAAAACCCTCGCTTAGTCGAACATGAGATGCAGACAC

TGGATGCCCGCCAGGACATGCTCGTGGTTGAAGTGCCGAAATTAGGTAAAGACGCGTGCGCGAAGGCGATTAAAGAGTGG

GGTCAGCCGAAATCCAAGATTACACACTTAATTTTTACCTCAGCGTCGACTACCGATATGCCTGGTGCCGACTATCATTG

TGCCAAGTTGTTGGGCTTAAGCCCCTCGGTTAAGCGTGTGATGATGTACCAACTGGGTTGTTATGGCGGTGGCACGGTGC

TGAGAATTGCGAAAGATATTGCCGAGAATAATAAAGGCGCGCGTGTTCTGGCCGTTTGCTGTGATATCATGGCATGCCTG

TTTCGTGGTCCTTCTGAGAGCGACTTAGAACTGCTGGTCGGCCAGGCTATATTCGGGGATGGTGCGGCGGCAGTCATTGT

TGGCGCGGAACCCGATGAAAGCGTTGGCGAGCGCCCAATCTTCGAATTAGTAAGCACAGGTCAGACGATATTACCAAATA

GCGAAGGCACTATCGGGGGCCACATCCGCGAAGCTGGACTGATTTTTGACCTGCATAAAGATGTCCCGATGCTGATTAGT

AATAATATCGAGAAATGTCTGATTGAGGCATTTACCCCAATTGGAATTAGCGACTGGAACAGCATTTTCTGGATAACGCA

TCCGGGCGGCAAAGCTATCCTGGATAAAGTTGAGGAAAAGCTCCACCTGAAATCTGATAAGTTTGTAGATTCCCGCCACG

TGTTGAGCGAACATGGTAATATGAGCTCCTCAACAGTCCTATTCGTTATGGATGAACTGCGCAAACGCTCGCTCGAAGAA

GGTAAAAGTACCACCGGCGATGGCTTTGAATGGGGCGTGTTATTCGGTTTTGGCCCAGGCTTAACCGTTGAGCGTGTGGT

GGTACGATCGGTTCCTATAAAATATTGACTCTAGAAATAATTTTGTTTAACTTTAAGAAGGAGATATACATATGTCCCCT

ATACTAGGTTATTGGAAAATTAAGGGCCTTGTGCAACCCACTCGACTTCTTTTGGAATATCTTGAAGAAAAATATGAAGA

GCATTTGTATGAGCGCGATGAAGGTGATAAATGGCGAAACAAAAAGTTTGAATTGGGTTTGGAGTTTCCCAATCTTCCTT

ATTATATTGATGGTGATGTTAAATTAACACAGTCTATGGCCATCATACGTTATATAGCTGACAAGCACAACATGTTGGGT

GGTTGTCCAAAAGAGCGTGCAGAGATTTCAATGCTTGAAGGAGCGGTTTTGGATATTAGATACGGTGTTTCGAGAATTGC

ATATAGTAAAGACTTTGAAACTCTCAAAGTTGATTTTCTTAGCAAGCTACCTGAAATGCTGAAAATGTTCGAAGATCGTT

TATGTCATAAAACATATTTAAATGGTGATCATGTAACCCATCCTGACTTCATGTTGTATGACGCTCTTGATGTTGTTTTA

TACATGGACCCAATGTGCCTGGATGCGTTCCCAAAATTAGTTTGTTTTAAAAAACGTATTGAAGCTATCCCACAAATTGA

TAAGTACTTGAAATCCAGCAAGTATATAGCATGGCCTTTGCAGGGCTGGCAAGCCACGTTTGGTGGTGGCGACCATCCTC

CAAAATCGGATGGTTCAACTAGTGGTTCTGGTCATCACCATCACCATCACTCCATGAGCGATTACGACATCCCCACTACT

GAGAATCTTTATTTTCAGGGCGCCATGGCAGTAAAACATTTAATTGTGCTCAAATTTAAAGACGAAATTACTGAAGCACA

AAAAGAAGAGTTTTTCAAAACATACGTCAATCTGGTGAACATCATCCCGGCCATGAAAGACGTCTATTGGGGCAAAGACG

TAACGCAGAAAAACAAGGAAGAAGGTTATACCCACATCGTGGAGGTCACTTTCGAATCAGTCGAAACGATCCAGGATTAC

ATTATTCATCCGGCACATGTAGGGTTCGGCGATGTATATCGTAGCTTCTGGGAAAAACTCCTGATTTTTGACTATACCCC

GCGTAAATAAAGAAAAGAATTCACGATCTTAAGTAGGCGCGGAAAATAATGGAGTTCGACTTCAACAAATACATGGACAG

CAAAGCCATGACCGTTAATGAAGCACTGAATAAAGCAATTCCGCTGCGTTATCCGCAGAAAATCTATGAAAGCATGCGTT

ATAGCCTGCTGGCAGGCGGTAAACGTGTTCGTCCGGTTCTGTGTATTGCAGCATGTGAACTGGTTGGTGGCACCGAAGAA

CTGGCAATTCCGACCGCATGTGCAATTGAAATGATTCATACCATGAGCCTGATGCATGATGATCTGCCGTGTATTGATAA

TGATGACCTGCGTCGTGGTAAACCGACCAATCATAAAATCTTTGGTGAAGATACCGCAGTGACCGCAGGTAATGCACTGC

ATAGTTATGCATTTGAACATATTGCAGTGAGCACCAGCAAAACCGTTGGTGCAGATCGTATTCTGCGTATGGTTAGCGAA

CTGGGTCGTGCAACCGGTAGCGAAGGTGTTATGGGTGGTCAGATGGTTGATATTGCAAGTGAAGGTGATCCGAGCATTGA

TCTGCAGACCCTGGAATGGATTCATATTCATAAAACCGCAATGCTGCTGGAATGTAGCGTTGTTTGTGGTGCAATTATTG

GTGGTGCAAGCGAAATTGTTATTGAACGTGCCCGTCGTTATGCACGTTGTGTTGGTCTGCTGTTTCAGGTTGTTGATGAT

ATTCTGGATGTGACCAAAAGCAGTGATGAACTGGGCAAAACCGCAGGCAAAGATCTGATTAGCGATAAAGCAACCTATCC

GAAACTGATGGGTCTGGAAAAAGCCAAAGAATTTTCAGATGAACTGCTGAATCGTGCCAAAGGTGAACTGAGCTGTTTTG

ATCCGGTTAAAGCAGCACCGCTGCTGGGTCTGGCAGATTATGTTGCATTTCGTCAGAATTAAGGGATCCAAACTCGAGTA

AGGATCTCCAGGCATCAAATAAAACGAAAGGCTCAGTCGAAAGACTGGGCCTTTCGTTTTATCTGTTGTTTGTCGGTGAA

CGCTCTCTACTAGAGTCACACTGGCTCACCTTCGGGTGGGCCTTTCTGCGTTTATACCTAGGCTACAGCCGATAGTCTGG

AACAGCGCACTTACGGGTTGCTGCGCAACCCAAGTGCTACCGGCGCGGCAGCGTGACCCGTGTCGGCGGCTCCAACGGCT

CGCCATCGTCCAGAAAACACGGCTCATCGGGCATCGGCAGGCGCTGCTGCCCGCGCCGTTCCCATTCCTCCGTTTCGGTC

AAGGCTGGCAGGTCTGGTTCCATGCCCGGAATGCCGGGCTGGCTGGGCGGCTCCTCGCCGGGGCCGGTCGGTAGTTGCTG

CTCGCCCGGATACAGGGTCGGGATGCGGCGCAGGTCGCCATGCCCCAACAGCGATTCGTCCTGGTCGTCGTGATCAACCA

CCACGGCGGCACTGAACACCGACAGGCGCAACTGGTCGCGGGGCTGGCCCCACGCCACGCGGTCATTGACCACGTAGGCC

AACACGGTGCCGGGGCCGTTGAGCTTCACGACGGAGATCCAGCGCTCGGCCACCAAGTCCTTGACTGCGTATTGGACCGT

CCGCAAAGAACGTCCGATGAGCTTGGAAAGTGTCTTCTGGCTGACCACCACGGCGTTCTGGTGGCCCATCTGCGCCACGA

GGTGATGCAGCAGCATTGCCGCCGTGGGTTTCCTCGCAATAAGCCCGGCCCACGCCTCATGCGCTTTGCGTTCCGTTTGC

ACCCAGTGACCGGGCTTGTTCTTGGCTTGAATGCCGATTTCTCTGGACTGCGTGGCCATGCTTATCTCCATGCGGTAGGG

GTGCCGCACGGTTGCGGCACCATGCGCAATCAGCTGCAACTTTTCGGCAGCGCGACAACAATTATGCGTTGCGTAAAAGT

GGCAGTCAATTACAGATTTTCTTTAACCTACGCAATGAGCTATTGCGGGGGGTGCCGCAATGAGCTGTTGCGTACCCCCC

TTTTTTAAGTTGTTGATTTTTAAGTCTTTCGCATTTCGCCCTATATCTAGTTCTTTGGTGCCCAAAGAAGGGCACCCCTG

CGGGGTTCCCCCACGCCTTCGGCGCGGCTCCCCCTCCGGCAAAAAGTGGCCCCTCCGGGGCTTGTTGATCGACTGCGCGG

CCTTCGGCCTTGCCCAAGGTGGCGCTGCCCCCTTGGAACCCCCGCACTCGCCGCCGTGAGGCTCGGGGGGCAGGCGGGCG

GGCTTCGCCCTTCGACTGCCCCCACTCGCATAGGCTTGGGTCGTTCCAGGCGCGTCAAGGCCAAGCCGCTGCGCGGTCGC

TGCGCGAGCCTTGACCCGCCTTCCACTTGGTGTCCAACCGGCAAGCGAAGCGCGCAGGCCGCAGGCCGGAGGCACTAGTG

CTTGGATTCTCACCAATAAAAAACGCCCGGCGGCAACCGAGCGTTCTGAACAAATCCAGATGGAGTTCTGAGGTCATTAC

TGGATCTATCAACAGGAGTCCAAGCGAGCTCGATATCAAATTACGCCCCGCCCTGCCACTCATCGCAGTACTGTTGTAAT

TCATTAAGCATTCTGCCGACATGGAAGCCATCACAAACGGCATGATGAACCTGAATCGCCAGCGGCATCAGCACCTTGTC

GCCTTGCGTATAATATTTGCCCATGGTGAAAACGGGGGCGAAGAAGTTGTCCATATTGGCCACGTTTAAATCAAAACTGG

TGAAACTCACCCAGGGATTGGCTGAGACGAAAAACATATTCTCAATAAACCCTTTAGGGAAATAGGCCAGGTTTTCACCG

TAACACGCCACATCTTGCGAATATATGTGTAGAAACTGCCGGAAATCGTCGTGGTATTCACTCCAGAGCGATGAAAACGT

TTCAGTTTGCTCATGGAAAACGGTGTAACAAGGGTGAACACTATCCCATATCACCAGCTCACCGTCTTTCATTGCCATAC

GAAATTCCGGATGAGCATTCATCAGGCGGGCAAGAATGTGAATAAAGGCCGGATAAAACTTGTGCTTATTTTTCTTTACG

GTCTTTAAAAAGGCCGTAATATCCAGCTGAACGGTCTGGTTATAGGTACATTGAGCAACTGACTGAAATGCCTCAAAATG

TTCTTTACGATGCCATTGGGATATATCAACGGTGGTATATCCAGTGATTTTTTTCTCCATTTTAGCTTCCTTAGCTCCTG

AAAATCTCGATAACTCAAAAAATACGCCCGGTAGTGATCTTATTTCATTATGGTGAAAGTTGGAACCTCTTACGTGCCGA

TCAACGTCTCATTTTCGCCAGATATC

>pET-M11-AtaPT (Genbank: OQ725933)

TGGCGAATGGGACGCGCCCTGTAGCGGCGCATTAAGCGCGGCGGGTGTGGTGGTTACGCGCAGCGTGACCGCTACACTTG

CCAGCGCCCTAGCGCCCGCTCCTTTCGCTTTCTTCCCTTCCTTTCTCGCCACGTTCGCCGGCTTTCCCCGTCAAGCTCTA

AATCGGGGGCTCCCTTTAGGGTTCCGATTTAGTGCTTTACGGCACCTCGACCCCAAAAAACTTGATTAGGGTGATGGTTC

ACGTAGTGGGCCATCGCCCTGATAGACGGTTTTTCGCCCTTTGACGTTGGAGTCCACGTTCTTTAATAGTGGACTCTTGT

TCCAAACTGGAACAACACTCAACCCTATCTCGGTCTATTCTTTTGATTTATAAGGGATTTTGCCGATTTCGGCCTATTGG

TTAAAAAATGAGCTGATTTAACAAAAATTTAACGCGAATTTTAACAAAATATTAACGTTTACAATTTCAGGTGGCACTTT

TCGGGGAAATGTGCGCGGAACCCCTATTTGTTTATTTTTCTAAATACATTCAAATATGTATCCGCTCATGAATTAATTCT

TAGAAAAACTCATCGAGCATCAAATGAAACTGCAATTTATTCATATCAGGATTATCAATACCATATTTTTGAAAAAGCCG

TTTCTGTAATGAAGGAGAAAACTCACCGAGGCAGTTCCATAGGATGGCAAGATCCTGGTATCGGTCTGCGATTCCGACTC

GTCCAACATCAATACAACCTATTAATTTCCCCTCGTCAAAAATAAGGTTATCAAGTGAGAAATCACCATGAGTGACGACT

GAATCCGGTGAGAATGGCAAAAGTTTATGCATTTCTTTCCAGACTTGTTCAACAGGCCAGCCATTACGCTCGTCATCAAA

ATCACTCGCATCAACCAAACCGTTATTCATTCGTGATTGCGCCTGAGCGAGACGAAATACGCGATCGCTGTTAAAAGGAC

AATTACAAACAGGAATCGAATGCAACCGGCGCAGGAACACTGCCAGCGCATCAACAATATTTTCACCTGAATCAGGATAT

TCTTCTAATACCTGGAATGCTGTTTTCCCGGGGATCGCAGTGGTGAGTAACCATGCATCATCAGGAGTACGGATAAAATG

CTTGATGGTCGGAAGAGGCATAAATTCCGTCAGCCAGTTTAGTCTGACCATCTCATCTGTAACATCATTGGCAACGCTAC

CTTTGCCATGTTTCAGAAACAACTCTGGCGCATCGGGCTTCCCATACAATCGATAGATTGTCGCACCTGATTGCCCGACA

TTATCGCGAGCCCATTTATACCCATATAAATCAGCATCCATGTTGGAATTTAATCGCGGCCTAGAGCAAGACGTTTCCCG

TTGAATATGGCTCATAACACCCCTTGTATTACTGTTTATGTAAGCAGACAGTTTTATTGTTCATGACCAAAATCCCTTAA

CGTGAGTTTTCGTTCCACTGAGCGTCAGACCCCGTAGAAAAGATCAAAGGATCTTCTTGAGATCCTTTTTTTCTGCGCGT

AATCTGCTGCTTGCAAACAAAAAAACCACCGCTACCAGCGGTGGTTTGTTTGCCGGATCAAGAGCTACCAACTCTTTTTC

CGAAGGTAACTGGCTTCAGCAGAGCGCAGATACCAAATACTGTCCTTCTAGTGTAGCCGTAGTTAGGCCACCACTTCAAG

AACTCTGTAGCACCGCCTACATACCTCGCTCTGCTAATCCTGTTACCAGTGGCTGCTGCCAGTGGCGATAAGTCGTGTCT

TACCGGGTTGGACTCAAGACGATAGTTACCGGATAAGGCGCAGCGGTCGGGCTGAACGGGGGGTTCGTGCACACAGCCCA

GCTTGGAGCGAACGACCTACACCGAACTGAGATACCTACAGCGTGAGCTATGAGAAAGCGCCACGCTTCCCGAAGGGAGA

AAGGCGGACAGGTATCCGGTAAGCGGCAGGGTCGGAACAGGAGAGCGCACGAGGGAGCTTCCAGGGGGAAACGCCTGGTA

TCTTTATAGTCCTGTCGGGTTTCGCCACCTCTGACTTGAGCGTCGATTTTTGTGATGCTCGTCAGGGGGGCGGAGCCTAT

GGAAAAACGCCAGCAACGCGGCCTTTTTACGGTTCCTGGCCTTTTGCTGGCCTTTTGCTCACATGTTCTTTCCTGCGTTA

TCCCCTGATTCTGTGGATAACCGTATTACCGCCTTTGAGTGAGCTGATACCGCTCGCCGCAGCCGAACGACCGAGCGCAG

CGAGTCAGTGAGCGAGGAAGCGGAAGAGCGCCTGATGCGGTATTTTCTCCTTACGCATCTGTGCGGTATTTCACACCGCA

TATATGGTGCACTCTCAGTACAATCTGCTCTGATGCCGCATAGTTAAGCCAGTATACACTCCGCTATCGCTACGTGACTG

GGTCATGGCTGCGCCCCGACACCCGCCAACACCCGCTGACGCGCCCTGACGGGCTTGTCTGCTCCCGGCATCCGCTTACA

GACAAGCTGTGACCGTCTCCGGGAGCTGCATGTGTCAGAGGTTTTCACCGTCATCACCGAAACGCGCGAGGCAGCTGCGG

TAAAGCTCATCAGCGTGGTCGTGAAGCGATTCACAGATGTCTGCCTGTTCATCCGCGTCCAGCTCGTTGAGTTTCTCCAG

AAGCGTTAATGTCTGGCTTCTGATAAAGCGGGCCATGTTAAGGGCGGTTTTTTCCTGTTTGGTCACTGATGCCTCCGTGT

AAGGGGGATTTCTGTTCATGGGGGTAATGATACCGATGAAACGAGAGAGGATGCTCACGATACGGGTTACTGATGATGAA

CATGCCCGGTTACTGGAACGTTGTGAGGGTAAACAACTGGCGGTATGGATGCGGCGGGACCAGAGAAAAATCACTCAGGG

TCAATGCCAGCGCTTCGTTAATACAGATGTAGGTGTTCCACAGGGTAGCCAGCAGCATCCTGCGATGCAGATCCGGAACA

TAATGGTGCAGGGCGCTGACTTCCGCGTTTCCAGACTTTACGAAACACGGAAACCGAAGACCATTCATGTTGTTGCTCAG

GTCGCAGACGTTTTGCAGCAGCAGTCGCTTCACGTTCGCTCGCGTATCGGTGATTCATTCTGCTAACCAGTAAGGCAACC

CCGCCAGCCTAGCCGGGTCCTCAACGACAGGAGCACGATCATGCGCACCCGTGGGGCCGCCATGCCGGCGATAATGGCCT

GCTTCTCGCCGAAACGTTTGGTGGCGGGACCAGTGACGAAGGCTTGAGCGAGGGCGTGCAAGATTCCGAATACCGCAAGC

GACAGGCCGATCATCGTCGCGCTCCAGCGAAAGCGGTCCTCGCCGAAAATGACCCAGAGCGCTGCCGGCACCTGTCCTAC

GAGTTGCATGATAAAGAAGACAGTCATAAGTGCGGCGACGATAGTCATGCCCCGCGCCCACCGGAAGGAGCTGACTGGGT

TGAAGGCTCTCAAGGGCATCGGTCGAGATCCCGGTGCCTAATGAGTGAGCTAACTTACATTAATTGCGTTGCGCTCACTG

CCCGCTTTCCAGTCGGGAAACCTGTCGTGCCAGCTGCATTAATGAATCGGCCAACGCGCGGGGAGAGGCGGTTTGCGTAT

TGGGCGCCAGGGTGGTTTTTCTTTTCACCAGTGAGACGGGCAACAGCTGATTGCCCTTCACCGCCTGGCCCTGAGAGAGT

TGCAGCAAGCGGTCCACGCTGGTTTGCCCCAGCAGGCGAAAATCCTGTTTGATGGTGGTTAACGGCGGGATATAACATGA

GCTGTCTTCGGTATCGTCGTATCCCACTACCGAGATATCCGCACCAACGCGCAGCCCGGACTCGGTAATGGCGCGCATTG

CGCCCAGCGCCATCTGATCGTTGGCAACCAGCATCGCAGTGGGAACGATGCCCTCATTCAGCATTTGCATGGTTTGTTGA

AAACCGGACATGGCACTCCAGTCGCCTTCCCGTTCCGCTATCGGCTGAATTTGATTGCGAGTGAGATATTTATGCCAGCC

AGCCAGACGCAGACGCGCCGAGACAGAACTTAATGGGCCCGCTAACAGCGCGATTTGCTGGTGACCCAATGCGACCAGAT

GCTCCACGCCCAGTCGCGTACCGTCTTCATGGGAGAAAATAATACTGTTGATGGGTGTCTGGTCAGAGACATCAAGAAAT

AACGCCGGAACATTAGTGCAGGCAGCTTCCACAGCAATGGCATCCTGGTCATCCAGCGGATAGTTAATGATCAGCCCACT

GACGCGTTGCGCGAGAAGATTGTGCACCGCCGCTTTACAGGCTTCGACGCCGCTTCGTTCTACCATCGACACCACCACGC

TGGCACCCAGTTGATCGGCGCGAGATTTAATCGCCGCGACAATTTGCGACGGCGCGTGCAGGGCCAGACTGGAGGTGGCA

ACGCCAATCAGCAACGACTGTTTGCCCGCCAGTTGTTGTGCCACGCGGTTGGGAATGTAATTCAGCTCCGCCATCGCCGC

TTCCACTTTTTCCCGCGTTTTCGCAGAAACGTGGCTGGCCTGGTTCACCACGCGGGAAACGGTCTGATAAGAGACACCGG

CATACTCTGCGACATCGTATAACGTTACTGGTTTCACATTCACCACCCTGAATTGACTCTCTTCCGGGCGCTATCATGCC

ATACCGCGAAAGGTTTTGCGCCATTCGATGGTGTCCGGGATCTCGACGCTCTCCCTTATGCGACTCCTGCATTAGGAAGC

AGCCCAGTAGTAGGTTGAGGCCGTTGAGCACCGCCGCCGCAAGGAATGGTGCATGCAAGGAGATGGCGCCCAACAGTCCC

CCGGCCACGGGGCCTGCCACCATACCCACGCCGAAACAAGCGCTCATGAGCCCGAAGTGGCGAGCCCGATCTTCCCCATC

GGTGATGTCGGCGATATAGGCGCCAGCAACCGCACCTGTGGCGCCGGTGATGCCGGCCACGATGCGTCCGGCGTAGAGGA

TCGAGATCTCGATCCCGCGAAATTAATACGACTCACTATAGGGGAATTGTGAGCGGATAACAATTCCCCTCTAGAAATAA

TTTTGATTTAACTTTAAGAAGGAGATATACCATGAAACATCACCATCACCATCACCCCATGAGCGATTACGACATCCCCA

CTACTGAGAATCTTTATTTTCAGGGCGCCATGGCGATGCTGCCTCCGAGCGATAGCAAAGATCCGCGTCCGTGGCAGATT

CTGAGCCAGGCACTGGGTTTTCCGAATTATGATCAAGAACTGTGGTGGCAGAATACCGCAGAAACCCTGAATCGTGTTCT

GGAACAGTGTGATTATAGCGTTCATCTGCAGTATAAATACCTGGCCTTCTACCACAAATATATCCTGCCGAGCCTGGGTC

CGTTTCGTCGTCCGGGTGTTGAACCGGAATATATCAGCGGTCTGAGTCATGGTGGTCATCCGCTGGAAATTAGCGTGAAA

ATTGATAAAAGCAAGACCATTTGTCGTCTGGGTCTGCAGGCAATTGGTCCGCTGGCAGGCACCGCACGTGATCCGCTGAA

TAGCTTTGGTGATCGTGAACTGCTGAAAAATCTGGCAACCCTGCTGCCGCATGTTGATCTGCGTCTGTTTGATCATTTTA

ATGCACAGGTTGGTCTGGATCGTGCACAGTGTGCAGTTGCAACCACCAAACTGATTAAAGAAAGCCATAATATTGTGTGC

ACCAGCCTGGATCTGAAAGATGGTGAAGTTATTCCGAAAGTGTACTTTAGCACCATTCCTAAAGGTCTGGTTACCGAAAC

ACCGCTGTTCGATCTGACCTTTGCAGCAATTGAGCAGATGGAAGTGTATCATAAAGATGCACCGCTGCGTACCGCACTGA

GCAGCCTGAAAGATTTTCTGCGTCCGCGTGTTCCGACCGATGCAAGCATTACCCCTCCGCTGACCGGTCTGATTGGTGTT

GATTGTATTGATCCGATGCTGAGCCGTCTGAAAGTTTATCTGGCCACCTTTCGTATGGATCTGAGCCTGATTCGTGATTA

TTGGACCTTAGGTGGTCTGCTGACCGATGCCGGTACAATGAAAGGCCTGGAAATGGTTGAAACCCTGGCAAAAACACTGA

AACTGGGTGATGAAGCATGTGAAACACTGGATGCAGAACGTCTGCCGTTTGGTATTAACTATGCAATGAAACCGGGTACA

GCAGAACTGGCACCGCCTCAGATCTATTTTCCGCTGCTGGGTATTAATGATGGCTTTATTGCAGATGCCCTGGTGGAATT

TTTTCAGTATATGGGTTGGGAAGATCAGGCCAATCGTTATAAAGATGAACTGAAAGCCAAATTTCCGAACGTGGATATTA

GCCAGACCAAAAATGTTCATCGTTGGCTGGGTGTTGCATATAGCGAAACCAAAGGTCCGAGCATGAACATCTATTATGAT

GTTGTTGCAGGTAATGTGGCACGCGTTTAACTCGAGCACCACCACCACCACCACTGAGATCCGGCTGCTAACAAAGCCCG

AAAGGAAGCTGAGTTGGCTGCTGCCACCGCTGAGCAATAACTAGCATAACCCCTTGGGGCCTCTAAACGGGTCTTGAGGG

GTTTTTTGCTGAAAGGAGGAACTATATCCGGAT

>pET-M11-AtaPTE91A (Genbank: OQ725934)

TGGCGAATGGGACGCGCCCTGTAGCGGCGCATTAAGCGCGGCGGGTGTGGTGGTTACGCGCAGCGTGACCGCTACACTTG

CCAGCGCCCTAGCGCCCGCTCCTTTCGCTTTCTTCCCTTCCTTTCTCGCCACGTTCGCCGGCTTTCCCCGTCAAGCTCTA

AATCGGGGGCTCCCTTTAGGGTTCCGATTTAGTGCTTTACGGCACCTCGACCCCAAAAAACTTGATTAGGGTGATGGTTC

ACGTAGTGGGCCATCGCCCTGATAGACGGTTTTTCGCCCTTTGACGTTGGAGTCCACGTTCTTTAATAGTGGACTCTTGT

TCCAAACTGGAACAACACTCAACCCTATCTCGGTCTATTCTTTTGATTTATAAGGGATTTTGCCGATTTCGGCCTATTGG

TTAAAAAATGAGCTGATTTAACAAAAATTTAACGCGAATTTTAACAAAATATTAACGTTTACAATTTCAGGTGGCACTTT

TCGGGGAAATGTGCGCGGAACCCCTATTTGTTTATTTTTCTAAATACATTCAAATATGTATCCGCTCATGAATTAATTCT

TAGAAAAACTCATCGAGCATCAAATGAAACTGCAATTTATTCATATCAGGATTATCAATACCATATTTTTGAAAAAGCCG

TTTCTGTAATGAAGGAGAAAACTCACCGAGGCAGTTCCATAGGATGGCAAGATCCTGGTATCGGTCTGCGATTCCGACTC

GTCCAACATCAATACAACCTATTAATTTCCCCTCGTCAAAAATAAGGTTATCAAGTGAGAAATCACCATGAGTGACGACT

GAATCCGGTGAGAATGGCAAAAGTTTATGCATTTCTTTCCAGACTTGTTCAACAGGCCAGCCATTACGCTCGTCATCAAA

ATCACTCGCATCAACCAAACCGTTATTCATTCGTGATTGCGCCTGAGCGAGACGAAATACGCGATCGCTGTTAAAAGGAC

AATTACAAACAGGAATCGAATGCAACCGGCGCAGGAACACTGCCAGCGCATCAACAATATTTTCACCTGAATCAGGATAT

TCTTCTAATACCTGGAATGCTGTTTTCCCGGGGATCGCAGTGGTGAGTAACCATGCATCATCAGGAGTACGGATAAAATG

CTTGATGGTCGGAAGAGGCATAAATTCCGTCAGCCAGTTTAGTCTGACCATCTCATCTGTAACATCATTGGCAACGCTAC

CTTTGCCATGTTTCAGAAACAACTCTGGCGCATCGGGCTTCCCATACAATCGATAGATTGTCGCACCTGATTGCCCGACA

TTATCGCGAGCCCATTTATACCCATATAAATCAGCATCCATGTTGGAATTTAATCGCGGCCTAGAGCAAGACGTTTCCCG

TTGAATATGGCTCATAACACCCCTTGTATTACTGTTTATGTAAGCAGACAGTTTTATTGTTCATGACCAAAATCCCTTAA

CGTGAGTTTTCGTTCCACTGAGCGTCAGACCCCGTAGAAAAGATCAAAGGATCTTCTTGAGATCCTTTTTTTCTGCGCGT

AATCTGCTGCTTGCAAACAAAAAAACCACCGCTACCAGCGGTGGTTTGTTTGCCGGATCAAGAGCTACCAACTCTTTTTC

CGAAGGTAACTGGCTTCAGCAGAGCGCAGATACCAAATACTGTCCTTCTAGTGTAGCCGTAGTTAGGCCACCACTTCAAG

AACTCTGTAGCACCGCCTACATACCTCGCTCTGCTAATCCTGTTACCAGTGGCTGCTGCCAGTGGCGATAAGTCGTGTCT

TACCGGGTTGGACTCAAGACGATAGTTACCGGATAAGGCGCAGCGGTCGGGCTGAACGGGGGGTTCGTGCACACAGCCCA

GCTTGGAGCGAACGACCTACACCGAACTGAGATACCTACAGCGTGAGCTATGAGAAAGCGCCACGCTTCCCGAAGGGAGA

AAGGCGGACAGGTATCCGGTAAGCGGCAGGGTCGGAACAGGAGAGCGCACGAGGGAGCTTCCAGGGGGAAACGCCTGGTA

TCTTTATAGTCCTGTCGGGTTTCGCCACCTCTGACTTGAGCGTCGATTTTTGTGATGCTCGTCAGGGGGGCGGAGCCTAT

GGAAAAACGCCAGCAACGCGGCCTTTTTACGGTTCCTGGCCTTTTGCTGGCCTTTTGCTCACATGTTCTTTCCTGCGTTA

TCCCCTGATTCTGTGGATAACCGTATTACCGCCTTTGAGTGAGCTGATACCGCTCGCCGCAGCCGAACGACCGAGCGCAG

CGAGTCAGTGAGCGAGGAAGCGGAAGAGCGCCTGATGCGGTATTTTCTCCTTACGCATCTGTGCGGTATTTCACACCGCA

TATATGGTGCACTCTCAGTACAATCTGCTCTGATGCCGCATAGTTAAGCCAGTATACACTCCGCTATCGCTACGTGACTG

GGTCATGGCTGCGCCCCGACACCCGCCAACACCCGCTGACGCGCCCTGACGGGCTTGTCTGCTCCCGGCATCCGCTTACA

GACAAGCTGTGACCGTCTCCGGGAGCTGCATGTGTCAGAGGTTTTCACCGTCATCACCGAAACGCGCGAGGCAGCTGCGG

TAAAGCTCATCAGCGTGGTCGTGAAGCGATTCACAGATGTCTGCCTGTTCATCCGCGTCCAGCTCGTTGAGTTTCTCCAG

AAGCGTTAATGTCTGGCTTCTGATAAAGCGGGCCATGTTAAGGGCGGTTTTTTCCTGTTTGGTCACTGATGCCTCCGTGT

AAGGGGGATTTCTGTTCATGGGGGTAATGATACCGATGAAACGAGAGAGGATGCTCACGATACGGGTTACTGATGATGAA

CATGCCCGGTTACTGGAACGTTGTGAGGGTAAACAACTGGCGGTATGGATGCGGCGGGACCAGAGAAAAATCACTCAGGG

TCAATGCCAGCGCTTCGTTAATACAGATGTAGGTGTTCCACAGGGTAGCCAGCAGCATCCTGCGATGCAGATCCGGAACA

TAATGGTGCAGGGCGCTGACTTCCGCGTTTCCAGACTTTACGAAACACGGAAACCGAAGACCATTCATGTTGTTGCTCAG

GTCGCAGACGTTTTGCAGCAGCAGTCGCTTCACGTTCGCTCGCGTATCGGTGATTCATTCTGCTAACCAGTAAGGCAACC

CCGCCAGCCTAGCCGGGTCCTCAACGACAGGAGCACGATCATGCGCACCCGTGGGGCCGCCATGCCGGCGATAATGGCCT

GCTTCTCGCCGAAACGTTTGGTGGCGGGACCAGTGACGAAGGCTTGAGCGAGGGCGTGCAAGATTCCGAATACCGCAAGC

GACAGGCCGATCATCGTCGCGCTCCAGCGAAAGCGGTCCTCGCCGAAAATGACCCAGAGCGCTGCCGGCACCTGTCCTAC

GAGTTGCATGATAAAGAAGACAGTCATAAGTGCGGCGACGATAGTCATGCCCCGCGCCCACCGGAAGGAGCTGACTGGGT

TGAAGGCTCTCAAGGGCATCGGTCGAGATCCCGGTGCCTAATGAGTGAGCTAACTTACATTAATTGCGTTGCGCTCACTG

CCCGCTTTCCAGTCGGGAAACCTGTCGTGCCAGCTGCATTAATGAATCGGCCAACGCGCGGGGAGAGGCGGTTTGCGTAT

TGGGCGCCAGGGTGGTTTTTCTTTTCACCAGTGAGACGGGCAACAGCTGATTGCCCTTCACCGCCTGGCCCTGAGAGAGT

TGCAGCAAGCGGTCCACGCTGGTTTGCCCCAGCAGGCGAAAATCCTGTTTGATGGTGGTTAACGGCGGGATATAACATGA

GCTGTCTTCGGTATCGTCGTATCCCACTACCGAGATATCCGCACCAACGCGCAGCCCGGACTCGGTAATGGCGCGCATTG

CGCCCAGCGCCATCTGATCGTTGGCAACCAGCATCGCAGTGGGAACGATGCCCTCATTCAGCATTTGCATGGTTTGTTGA

AAACCGGACATGGCACTCCAGTCGCCTTCCCGTTCCGCTATCGGCTGAATTTGATTGCGAGTGAGATATTTATGCCAGCC

AGCCAGACGCAGACGCGCCGAGACAGAACTTAATGGGCCCGCTAACAGCGCGATTTGCTGGTGACCCAATGCGACCAGAT

GCTCCACGCCCAGTCGCGTACCGTCTTCATGGGAGAAAATAATACTGTTGATGGGTGTCTGGTCAGAGACATCAAGAAAT

AACGCCGGAACATTAGTGCAGGCAGCTTCCACAGCAATGGCATCCTGGTCATCCAGCGGATAGTTAATGATCAGCCCACT

GACGCGTTGCGCGAGAAGATTGTGCACCGCCGCTTTACAGGCTTCGACGCCGCTTCGTTCTACCATCGACACCACCACGC

TGGCACCCAGTTGATCGGCGCGAGATTTAATCGCCGCGACAATTTGCGACGGCGCGTGCAGGGCCAGACTGGAGGTGGCA

ACGCCAATCAGCAACGACTGTTTGCCCGCCAGTTGTTGTGCCACGCGGTTGGGAATGTAATTCAGCTCCGCCATCGCCGC

TTCCACTTTTTCCCGCGTTTTCGCAGAAACGTGGCTGGCCTGGTTCACCACGCGGGAAACGGTCTGATAAGAGACACCGG

CATACTCTGCGACATCGTATAACGTTACTGGTTTCACATTCACCACCCTGAATTGACTCTCTTCCGGGCGCTATCATGCC

ATACCGCGAAAGGTTTTGCGCCATTCGATGGTGTCCGGGATCTCGACGCTCTCCCTTATGCGACTCCTGCATTAGGAAGC

AGCCCAGTAGTAGGTTGAGGCCGTTGAGCACCGCCGCCGCAAGGAATGGTGCATGCAAGGAGATGGCGCCCAACAGTCCC

CCGGCCACGGGGCCTGCCACCATACCCACGCCGAAACAAGCGCTCATGAGCCCGAAGTGGCGAGCCCGATCTTCCCCATC

GGTGATGTCGGCGATATAGGCGCCAGCAACCGCACCTGTGGCGCCGGTGATGCCGGCCACGATGCGTCCGGCGTAGAGGA

TCGAGATCTCGATCCCGCGAAATTAATACGACTCACTATAGGGGAATTGTGAGCGGATAACAATTCCCCTCTAGAAATAA

TTTTGATTTAACTTTAAGAAGGAGATATACCATGAAACATCACCATCACCATCACCCCATGAGCGATTACGACATCCCCA

CTACTGAGAATCTTTATTTTCAGGGCGCCATGGCGATGCTGCCTCCGAGCGATAGCAAAGATCCGCGTCCGTGGCAGATT

CTGAGCCAGGCACTGGGTTTTCCGAATTATGATCAAGAACTGTGGTGGCAGAATACCGCAGAAACCCTGAATCGTGTTCT

GGAACAGTGTGATTATAGCGTTCATCTGCAGTATAAATACCTGGCCTTCTACCACAAATATATCCTGCCGAGCCTGGGTC

CGTTTCGTCGTCCGGGTGTTGAACCGGAATATATCAGCGGTCTGAGTCATGGTGGTCATCCGCTGGCAATTAGCGTGAAA

ATTGATAAAAGCAAGACCATTTGTCGTCTGGGTCTGCAGGCAATTGGTCCGCTGGCAGGCACCGCACGTGATCCGCTGAA

TAGCTTTGGTGATCGTGAACTGCTGAAAAATCTGGCAACCCTGCTGCCGCATGTTGATCTGCGTCTGTTTGATCATTTTA

ATGCACAGGTTGGTCTGGATCGTGCACAGTGTGCAGTTGCAACCACCAAACTGATTAAAGAAAGCCATAATATTGTGTGC

ACCAGCCTGGATCTGAAAGATGGTGAAGTTATTCCGAAAGTGTACTTTAGCACCATTCCTAAAGGTCTGGTTACCGAAAC

ACCGCTGTTCGATCTGACCTTTGCAGCAATTGAGCAGATGGAAGTGTATCATAAAGATGCACCGCTGCGTACCGCACTGA

GCAGCCTGAAAGATTTTCTGCGTCCGCGTGTTCCGACCGATGCAAGCATTACCCCTCCGCTGACCGGTCTGATTGGTGTT

GATTGTATTGATCCGATGCTGAGCCGTCTGAAAGTTTATCTGGCCACCTTTCGTATGGATCTGAGCCTGATTCGTGATTA

TTGGACCTTAGGTGGTCTGCTGACCGATGCCGGTACAATGAAAGGCCTGGAAATGGTTGAAACCCTGGCAAAAACACTGA

AACTGGGTGATGAAGCATGTGAAACACTGGATGCAGAACGTCTGCCGTTTGGTATTAACTATGCAATGAAACCGGGTACA

GCAGAACTGGCACCGCCTCAGATCTATTTTCCGCTGCTGGGTATTAATGATGGCTTTATTGCAGATGCCCTGGTGGAATT

TTTTCAGTATATGGGTTGGGAAGATCAGGCCAATCGTTATAAAGATGAACTGAAAGCCAAATTTCCGAACGTGGATATTA

GCCAGACCAAAAATGTTCATCGTTGGCTGGGTGTTGCATATAGCGAAACCAAAGGTCCGAGCATGAACATCTATTATGAT

GTTGTTGCAGGTAATGTGGCACGCGTTTAACTCGAGCACCACCACCACCACCACTGAGATCCGGCTGCTAACAAAGCCCG

AAAGGAAGCTGAGTTGGCTGCTGCCACCGCTGAGCAATAACTAGCATAACCCCTTGGGGCCTCTAAACGGGTCTTGAGGG

GTTTTTTGCTGAAAGGAGGAACTATATCCGGAT

>pET-M11-AtaPT E91D (Genbank: OQ725935)

TGGCGAATGGGACGCGCCCTGTAGCGGCGCATTAAGCGCGGCGGGTGTGGTGGTTACGCGCAGCGTGACCGCTACACTTG

CCAGCGCCCTAGCGCCCGCTCCTTTCGCTTTCTTCCCTTCCTTTCTCGCCACGTTCGCCGGCTTTCCCCGTCAAGCTCTA

AATCGGGGGCTCCCTTTAGGGTTCCGATTTAGTGCTTTACGGCACCTCGACCCCAAAAAACTTGATTAGGGTGATGGTTC

ACGTAGTGGGCCATCGCCCTGATAGACGGTTTTTCGCCCTTTGACGTTGGAGTCCACGTTCTTTAATAGTGGACTCTTGT

TCCAAACTGGAACAACACTCAACCCTATCTCGGTCTATTCTTTTGATTTATAAGGGATTTTGCCGATTTCGGCCTATTGG

TTAAAAAATGAGCTGATTTAACAAAAATTTAACGCGAATTTTAACAAAATATTAACGTTTACAATTTCAGGTGGCACTTT

TCGGGGAAATGTGCGCGGAACCCCTATTTGTTTATTTTTCTAAATACATTCAAATATGTATCCGCTCATGAATTAATTCT

TAGAAAAACTCATCGAGCATCAAATGAAACTGCAATTTATTCATATCAGGATTATCAATACCATATTTTTGAAAAAGCCG

TTTCTGTAATGAAGGAGAAAACTCACCGAGGCAGTTCCATAGGATGGCAAGATCCTGGTATCGGTCTGCGATTCCGACTC

GTCCAACATCAATACAACCTATTAATTTCCCCTCGTCAAAAATAAGGTTATCAAGTGAGAAATCACCATGAGTGACGACT

GAATCCGGTGAGAATGGCAAAAGTTTATGCATTTCTTTCCAGACTTGTTCAACAGGCCAGCCATTACGCTCGTCATCAAA

ATCACTCGCATCAACCAAACCGTTATTCATTCGTGATTGCGCCTGAGCGAGACGAAATACGCGATCGCTGTTAAAAGGAC

AATTACAAACAGGAATCGAATGCAACCGGCGCAGGAACACTGCCAGCGCATCAACAATATTTTCACCTGAATCAGGATAT

TCTTCTAATACCTGGAATGCTGTTTTCCCGGGGATCGCAGTGGTGAGTAACCATGCATCATCAGGAGTACGGATAAAATG

CTTGATGGTCGGAAGAGGCATAAATTCCGTCAGCCAGTTTAGTCTGACCATCTCATCTGTAACATCATTGGCAACGCTAC

CTTTGCCATGTTTCAGAAACAACTCTGGCGCATCGGGCTTCCCATACAATCGATAGATTGTCGCACCTGATTGCCCGACA

TTATCGCGAGCCCATTTATACCCATATAAATCAGCATCCATGTTGGAATTTAATCGCGGCCTAGAGCAAGACGTTTCCCG

TTGAATATGGCTCATAACACCCCTTGTATTACTGTTTATGTAAGCAGACAGTTTTATTGTTCATGACCAAAATCCCTTAA

CGTGAGTTTTCGTTCCACTGAGCGTCAGACCCCGTAGAAAAGATCAAAGGATCTTCTTGAGATCCTTTTTTTCTGCGCGT

AATCTGCTGCTTGCAAACAAAAAAACCACCGCTACCAGCGGTGGTTTGTTTGCCGGATCAAGAGCTACCAACTCTTTTTC

CGAAGGTAACTGGCTTCAGCAGAGCGCAGATACCAAATACTGTCCTTCTAGTGTAGCCGTAGTTAGGCCACCACTTCAAG

AACTCTGTAGCACCGCCTACATACCTCGCTCTGCTAATCCTGTTACCAGTGGCTGCTGCCAGTGGCGATAAGTCGTGTCT

TACCGGGTTGGACTCAAGACGATAGTTACCGGATAAGGCGCAGCGGTCGGGCTGAACGGGGGGTTCGTGCACACAGCCCA

GCTTGGAGCGAACGACCTACACCGAACTGAGATACCTACAGCGTGAGCTATGAGAAAGCGCCACGCTTCCCGAAGGGAGA

AAGGCGGACAGGTATCCGGTAAGCGGCAGGGTCGGAACAGGAGAGCGCACGAGGGAGCTTCCAGGGGGAAACGCCTGGTA

TCTTTATAGTCCTGTCGGGTTTCGCCACCTCTGACTTGAGCGTCGATTTTTGTGATGCTCGTCAGGGGGGCGGAGCCTAT

GGAAAAACGCCAGCAACGCGGCCTTTTTACGGTTCCTGGCCTTTTGCTGGCCTTTTGCTCACATGTTCTTTCCTGCGTTA

TCCCCTGATTCTGTGGATAACCGTATTACCGCCTTTGAGTGAGCTGATACCGCTCGCCGCAGCCGAACGACCGAGCGCAG

CGAGTCAGTGAGCGAGGAAGCGGAAGAGCGCCTGATGCGGTATTTTCTCCTTACGCATCTGTGCGGTATTTCACACCGCA

TATATGGTGCACTCTCAGTACAATCTGCTCTGATGCCGCATAGTTAAGCCAGTATACACTCCGCTATCGCTACGTGACTG

GGTCATGGCTGCGCCCCGACACCCGCCAACACCCGCTGACGCGCCCTGACGGGCTTGTCTGCTCCCGGCATCCGCTTACA

GACAAGCTGTGACCGTCTCCGGGAGCTGCATGTGTCAGAGGTTTTCACCGTCATCACCGAAACGCGCGAGGCAGCTGCGG

TAAAGCTCATCAGCGTGGTCGTGAAGCGATTCACAGATGTCTGCCTGTTCATCCGCGTCCAGCTCGTTGAGTTTCTCCAG

AAGCGTTAATGTCTGGCTTCTGATAAAGCGGGCCATGTTAAGGGCGGTTTTTTCCTGTTTGGTCACTGATGCCTCCGTGT

AAGGGGGATTTCTGTTCATGGGGGTAATGATACCGATGAAACGAGAGAGGATGCTCACGATACGGGTTACTGATGATGAA

CATGCCCGGTTACTGGAACGTTGTGAGGGTAAACAACTGGCGGTATGGATGCGGCGGGACCAGAGAAAAATCACTCAGGG

TCAATGCCAGCGCTTCGTTAATACAGATGTAGGTGTTCCACAGGGTAGCCAGCAGCATCCTGCGATGCAGATCCGGAACA

TAATGGTGCAGGGCGCTGACTTCCGCGTTTCCAGACTTTACGAAACACGGAAACCGAAGACCATTCATGTTGTTGCTCAG

GTCGCAGACGTTTTGCAGCAGCAGTCGCTTCACGTTCGCTCGCGTATCGGTGATTCATTCTGCTAACCAGTAAGGCAACC

CCGCCAGCCTAGCCGGGTCCTCAACGACAGGAGCACGATCATGCGCACCCGTGGGGCCGCCATGCCGGCGATAATGGCCT

GCTTCTCGCCGAAACGTTTGGTGGCGGGACCAGTGACGAAGGCTTGAGCGAGGGCGTGCAAGATTCCGAATACCGCAAGC

GACAGGCCGATCATCGTCGCGCTCCAGCGAAAGCGGTCCTCGCCGAAAATGACCCAGAGCGCTGCCGGCACCTGTCCTAC

GAGTTGCATGATAAAGAAGACAGTCATAAGTGCGGCGACGATAGTCATGCCCCGCGCCCACCGGAAGGAGCTGACTGGGT

TGAAGGCTCTCAAGGGCATCGGTCGAGATCCCGGTGCCTAATGAGTGAGCTAACTTACATTAATTGCGTTGCGCTCACTG

CCCGCTTTCCAGTCGGGAAACCTGTCGTGCCAGCTGCATTAATGAATCGGCCAACGCGCGGGGAGAGGCGGTTTGCGTAT

TGGGCGCCAGGGTGGTTTTTCTTTTCACCAGTGAGACGGGCAACAGCTGATTGCCCTTCACCGCCTGGCCCTGAGAGAGT

TGCAGCAAGCGGTCCACGCTGGTTTGCCCCAGCAGGCGAAAATCCTGTTTGATGGTGGTTAACGGCGGGATATAACATGA

GCTGTCTTCGGTATCGTCGTATCCCACTACCGAGATATCCGCACCAACGCGCAGCCCGGACTCGGTAATGGCGCGCATTG

CGCCCAGCGCCATCTGATCGTTGGCAACCAGCATCGCAGTGGGAACGATGCCCTCATTCAGCATTTGCATGGTTTGTTGA

AAACCGGACATGGCACTCCAGTCGCCTTCCCGTTCCGCTATCGGCTGAATTTGATTGCGAGTGAGATATTTATGCCAGCC

AGCCAGACGCAGACGCGCCGAGACAGAACTTAATGGGCCCGCTAACAGCGCGATTTGCTGGTGACCCAATGCGACCAGAT

GCTCCACGCCCAGTCGCGTACCGTCTTCATGGGAGAAAATAATACTGTTGATGGGTGTCTGGTCAGAGACATCAAGAAAT

AACGCCGGAACATTAGTGCAGGCAGCTTCCACAGCAATGGCATCCTGGTCATCCAGCGGATAGTTAATGATCAGCCCACT

GACGCGTTGCGCGAGAAGATTGTGCACCGCCGCTTTACAGGCTTCGACGCCGCTTCGTTCTACCATCGACACCACCACGC

TGGCACCCAGTTGATCGGCGCGAGATTTAATCGCCGCGACAATTTGCGACGGCGCGTGCAGGGCCAGACTGGAGGTGGCA

ACGCCAATCAGCAACGACTGTTTGCCCGCCAGTTGTTGTGCCACGCGGTTGGGAATGTAATTCAGCTCCGCCATCGCCGC

TTCCACTTTTTCCCGCGTTTTCGCAGAAACGTGGCTGGCCTGGTTCACCACGCGGGAAACGGTCTGATAAGAGACACCGG

CATACTCTGCGACATCGTATAACGTTACTGGTTTCACATTCACCACCCTGAATTGACTCTCTTCCGGGCGCTATCATGCC

ATACCGCGAAAGGTTTTGCGCCATTCGATGGTGTCCGGGATCTCGACGCTCTCCCTTATGCGACTCCTGCATTAGGAAGC

AGCCCAGTAGTAGGTTGAGGCCGTTGAGCACCGCCGCCGCAAGGAATGGTGCATGCAAGGAGATGGCGCCCAACAGTCCC

CCGGCCACGGGGCCTGCCACCATACCCACGCCGAAACAAGCGCTCATGAGCCCGAAGTGGCGAGCCCGATCTTCCCCATC

GGTGATGTCGGCGATATAGGCGCCAGCAACCGCACCTGTGGCGCCGGTGATGCCGGCCACGATGCGTCCGGCGTAGAGGA

TCGAGATCTCGATCCCGCGAAATTAATACGACTCACTATAGGGGAATTGTGAGCGGATAACAATTCCCCTCTAGAAATAA

TTTTGATTTAACTTTAAGAAGGAGATATACCATGAAACATCACCATCACCATCACCCCATGAGCGATTACGACATCCCCA

CTACTGAGAATCTTTATTTTCAGGGCGCCATGGCGATGCTGCCTCCGAGCGATAGCAAAGATCCGCGTCCGTGGCAGATT

CTGAGCCAGGCACTGGGTTTTCCGAATTATGATCAAGAACTGTGGTGGCAGAATACCGCAGAAACCCTGAATCGTGTTCT

GGAACAGTGTGATTATAGCGTTCATCTGCAGTATAAATACCTGGCCTTCTACCACAAATATATCCTGCCGAGCCTGGGTC

CGTTTCGTCGTCCGGGTGTTGAACCGGAATATATCAGCGGTCTGAGTCATGGTGGTCATCCGCTGGATATTAGCGTGAAA

ATTGATAAAAGCAAGACCATTTGTCGTCTGGGTCTGCAGGCAATTGGTCCGCTGGCAGGCACCGCACGTGATCCGCTGAA

TAGCTTTGGTGATCGTGAACTGCTGAAAAATCTGGCAACCCTGCTGCCGCATGTTGATCTGCGTCTGTTTGATCATTTTA

ATGCACAGGTTGGTCTGGATCGTGCACAGTGTGCAGTTGCAACCACCAAACTGATTAAAGAAAGCCATAATATTGTGTGC

ACCAGCCTGGATCTGAAAGATGGTGAAGTTATTCCGAAAGTGTACTTTAGCACCATTCCTAAAGGTCTGGTTACCGAAAC

ACCGCTGTTCGATCTGACCTTTGCAGCAATTGAGCAGATGGAAGTGTATCATAAAGATGCACCGCTGCGTACCGCACTGA

GCAGCCTGAAAGATTTTCTGCGTCCGCGTGTTCCGACCGATGCAAGCATTACCCCTCCGCTGACCGGTCTGATTGGTGTT

GATTGTATTGATCCGATGCTGAGCCGTCTGAAAGTTTATCTGGCCACCTTTCGTATGGATCTGAGCCTGATTCGTGATTA

TTGGACCTTAGGTGGTCTGCTGACCGATGCCGGTACAATGAAAGGCCTGGAAATGGTTGAAACCCTGGCAAAAACACTGA

AACTGGGTGATGAAGCATGTGAAACACTGGATGCAGAACGTCTGCCGTTTGGTATTAACTATGCAATGAAACCGGGTACA

GCAGAACTGGCACCGCCTCAGATCTATTTTCCGCTGCTGGGTATTAATGATGGCTTTATTGCAGATGCCCTGGTGGAATT

TTTTCAGTATATGGGTTGGGAAGATCAGGCCAATCGTTATAAAGATGAACTGAAAGCCAAATTTCCGAACGTGGATATTA

GCCAGACCAAAAATGTTCATCGTTGGCTGGGTGTTGCATATAGCGAAACCAAAGGTCCGAGCATGAACATCTATTATGAT

GTTGTTGCAGGTAATGTGGCACGCGTTTAACTCGAGCACCACCACCACCACCACTGAGATCCGGCTGCTAACAAAGCCCG

AAAGGAAGCTGAGTTGGCTGCTGCCACCGCTGAGCAATAACTAGCATAACCCCTTGGGGCCTCTAAACGGGTCTTGAGGG

GTTTTTTGCTGAAAGGAGGAACTATATCCGGAT

>pET-M11-AtaPT E91Q (Genbank: OQ725936)

TGGCGAATGGGACGCGCCCTGTAGCGGCGCATTAAGCGCGGCGGGTGTGGTGGTTACGCGCAGCGTGACCGCTACACTTG

CCAGCGCCCTAGCGCCCGCTCCTTTCGCTTTCTTCCCTTCCTTTCTCGCCACGTTCGCCGGCTTTCCCCGTCAAGCTCTA

AATCGGGGGCTCCCTTTAGGGTTCCGATTTAGTGCTTTACGGCACCTCGACCCCAAAAAACTTGATTAGGGTGATGGTTC

ACGTAGTGGGCCATCGCCCTGATAGACGGTTTTTCGCCCTTTGACGTTGGAGTCCACGTTCTTTAATAGTGGACTCTTGT

TCCAAACTGGAACAACACTCAACCCTATCTCGGTCTATTCTTTTGATTTATAAGGGATTTTGCCGATTTCGGCCTATTGG

TTAAAAAATGAGCTGATTTAACAAAAATTTAACGCGAATTTTAACAAAATATTAACGTTTACAATTTCAGGTGGCACTTT

TCGGGGAAATGTGCGCGGAACCCCTATTTGTTTATTTTTCTAAATACATTCAAATATGTATCCGCTCATGAATTAATTCT

TAGAAAAACTCATCGAGCATCAAATGAAACTGCAATTTATTCATATCAGGATTATCAATACCATATTTTTGAAAAAGCCG

TTTCTGTAATGAAGGAGAAAACTCACCGAGGCAGTTCCATAGGATGGCAAGATCCTGGTATCGGTCTGCGATTCCGACTC

GTCCAACATCAATACAACCTATTAATTTCCCCTCGTCAAAAATAAGGTTATCAAGTGAGAAATCACCATGAGTGACGACT

GAATCCGGTGAGAATGGCAAAAGTTTATGCATTTCTTTCCAGACTTGTTCAACAGGCCAGCCATTACGCTCGTCATCAAA

ATCACTCGCATCAACCAAACCGTTATTCATTCGTGATTGCGCCTGAGCGAGACGAAATACGCGATCGCTGTTAAAAGGAC

AATTACAAACAGGAATCGAATGCAACCGGCGCAGGAACACTGCCAGCGCATCAACAATATTTTCACCTGAATCAGGATAT

TCTTCTAATACCTGGAATGCTGTTTTCCCGGGGATCGCAGTGGTGAGTAACCATGCATCATCAGGAGTACGGATAAAATG

CTTGATGGTCGGAAGAGGCATAAATTCCGTCAGCCAGTTTAGTCTGACCATCTCATCTGTAACATCATTGGCAACGCTAC

CTTTGCCATGTTTCAGAAACAACTCTGGCGCATCGGGCTTCCCATACAATCGATAGATTGTCGCACCTGATTGCCCGACA

TTATCGCGAGCCCATTTATACCCATATAAATCAGCATCCATGTTGGAATTTAATCGCGGCCTAGAGCAAGACGTTTCCCG

TTGAATATGGCTCATAACACCCCTTGTATTACTGTTTATGTAAGCAGACAGTTTTATTGTTCATGACCAAAATCCCTTAA

CGTGAGTTTTCGTTCCACTGAGCGTCAGACCCCGTAGAAAAGATCAAAGGATCTTCTTGAGATCCTTTTTTTCTGCGCGT

AATCTGCTGCTTGCAAACAAAAAAACCACCGCTACCAGCGGTGGTTTGTTTGCCGGATCAAGAGCTACCAACTCTTTTTC

CGAAGGTAACTGGCTTCAGCAGAGCGCAGATACCAAATACTGTCCTTCTAGTGTAGCCGTAGTTAGGCCACCACTTCAAG

AACTCTGTAGCACCGCCTACATACCTCGCTCTGCTAATCCTGTTACCAGTGGCTGCTGCCAGTGGCGATAAGTCGTGTCT

TACCGGGTTGGACTCAAGACGATAGTTACCGGATAAGGCGCAGCGGTCGGGCTGAACGGGGGGTTCGTGCACACAGCCCA

GCTTGGAGCGAACGACCTACACCGAACTGAGATACCTACAGCGTGAGCTATGAGAAAGCGCCACGCTTCCCGAAGGGAGA

AAGGCGGACAGGTATCCGGTAAGCGGCAGGGTCGGAACAGGAGAGCGCACGAGGGAGCTTCCAGGGGGAAACGCCTGGTA

TCTTTATAGTCCTGTCGGGTTTCGCCACCTCTGACTTGAGCGTCGATTTTTGTGATGCTCGTCAGGGGGGCGGAGCCTAT

GGAAAAACGCCAGCAACGCGGCCTTTTTACGGTTCCTGGCCTTTTGCTGGCCTTTTGCTCACATGTTCTTTCCTGCGTTA

TCCCCTGATTCTGTGGATAACCGTATTACCGCCTTTGAGTGAGCTGATACCGCTCGCCGCAGCCGAACGACCGAGCGCAG

CGAGTCAGTGAGCGAGGAAGCGGAAGAGCGCCTGATGCGGTATTTTCTCCTTACGCATCTGTGCGGTATTTCACACCGCA

TATATGGTGCACTCTCAGTACAATCTGCTCTGATGCCGCATAGTTAAGCCAGTATACACTCCGCTATCGCTACGTGACTG

GGTCATGGCTGCGCCCCGACACCCGCCAACACCCGCTGACGCGCCCTGACGGGCTTGTCTGCTCCCGGCATCCGCTTACA

GACAAGCTGTGACCGTCTCCGGGAGCTGCATGTGTCAGAGGTTTTCACCGTCATCACCGAAACGCGCGAGGCAGCTGCGG

TAAAGCTCATCAGCGTGGTCGTGAAGCGATTCACAGATGTCTGCCTGTTCATCCGCGTCCAGCTCGTTGAGTTTCTCCAG

AAGCGTTAATGTCTGGCTTCTGATAAAGCGGGCCATGTTAAGGGCGGTTTTTTCCTGTTTGGTCACTGATGCCTCCGTGT

AAGGGGGATTTCTGTTCATGGGGGTAATGATACCGATGAAACGAGAGAGGATGCTCACGATACGGGTTACTGATGATGAA

CATGCCCGGTTACTGGAACGTTGTGAGGGTAAACAACTGGCGGTATGGATGCGGCGGGACCAGAGAAAAATCACTCAGGG

TCAATGCCAGCGCTTCGTTAATACAGATGTAGGTGTTCCACAGGGTAGCCAGCAGCATCCTGCGATGCAGATCCGGAACA

TAATGGTGCAGGGCGCTGACTTCCGCGTTTCCAGACTTTACGAAACACGGAAACCGAAGACCATTCATGTTGTTGCTCAG

GTCGCAGACGTTTTGCAGCAGCAGTCGCTTCACGTTCGCTCGCGTATCGGTGATTCATTCTGCTAACCAGTAAGGCAACC

CCGCCAGCCTAGCCGGGTCCTCAACGACAGGAGCACGATCATGCGCACCCGTGGGGCCGCCATGCCGGCGATAATGGCCT

GCTTCTCGCCGAAACGTTTGGTGGCGGGACCAGTGACGAAGGCTTGAGCGAGGGCGTGCAAGATTCCGAATACCGCAAGC

GACAGGCCGATCATCGTCGCGCTCCAGCGAAAGCGGTCCTCGCCGAAAATGACCCAGAGCGCTGCCGGCACCTGTCCTAC

GAGTTGCATGATAAAGAAGACAGTCATAAGTGCGGCGACGATAGTCATGCCCCGCGCCCACCGGAAGGAGCTGACTGGGT

TGAAGGCTCTCAAGGGCATCGGTCGAGATCCCGGTGCCTAATGAGTGAGCTAACTTACATTAATTGCGTTGCGCTCACTG

CCCGCTTTCCAGTCGGGAAACCTGTCGTGCCAGCTGCATTAATGAATCGGCCAACGCGCGGGGAGAGGCGGTTTGCGTAT

TGGGCGCCAGGGTGGTTTTTCTTTTCACCAGTGAGACGGGCAACAGCTGATTGCCCTTCACCGCCTGGCCCTGAGAGAGT

TGCAGCAAGCGGTCCACGCTGGTTTGCCCCAGCAGGCGAAAATCCTGTTTGATGGTGGTTAACGGCGGGATATAACATGA

GCTGTCTTCGGTATCGTCGTATCCCACTACCGAGATATCCGCACCAACGCGCAGCCCGGACTCGGTAATGGCGCGCATTG

CGCCCAGCGCCATCTGATCGTTGGCAACCAGCATCGCAGTGGGAACGATGCCCTCATTCAGCATTTGCATGGTTTGTTGA

AAACCGGACATGGCACTCCAGTCGCCTTCCCGTTCCGCTATCGGCTGAATTTGATTGCGAGTGAGATATTTATGCCAGCC

AGCCAGACGCAGACGCGCCGAGACAGAACTTAATGGGCCCGCTAACAGCGCGATTTGCTGGTGACCCAATGCGACCAGAT

GCTCCACGCCCAGTCGCGTACCGTCTTCATGGGAGAAAATAATACTGTTGATGGGTGTCTGGTCAGAGACATCAAGAAAT

AACGCCGGAACATTAGTGCAGGCAGCTTCCACAGCAATGGCATCCTGGTCATCCAGCGGATAGTTAATGATCAGCCCACT

GACGCGTTGCGCGAGAAGATTGTGCACCGCCGCTTTACAGGCTTCGACGCCGCTTCGTTCTACCATCGACACCACCACGC

TGGCACCCAGTTGATCGGCGCGAGATTTAATCGCCGCGACAATTTGCGACGGCGCGTGCAGGGCCAGACTGGAGGTGGCA

ACGCCAATCAGCAACGACTGTTTGCCCGCCAGTTGTTGTGCCACGCGGTTGGGAATGTAATTCAGCTCCGCCATCGCCGC

TTCCACTTTTTCCCGCGTTTTCGCAGAAACGTGGCTGGCCTGGTTCACCACGCGGGAAACGGTCTGATAAGAGACACCGG

CATACTCTGCGACATCGTATAACGTTACTGGTTTCACATTCACCACCCTGAATTGACTCTCTTCCGGGCGCTATCATGCC

ATACCGCGAAAGGTTTTGCGCCATTCGATGGTGTCCGGGATCTCGACGCTCTCCCTTATGCGACTCCTGCATTAGGAAGC

AGCCCAGTAGTAGGTTGAGGCCGTTGAGCACCGCCGCCGCAAGGAATGGTGCATGCAAGGAGATGGCGCCCAACAGTCCC

CCGGCCACGGGGCCTGCCACCATACCCACGCCGAAACAAGCGCTCATGAGCCCGAAGTGGCGAGCCCGATCTTCCCCATC

GGTGATGTCGGCGATATAGGCGCCAGCAACCGCACCTGTGGCGCCGGTGATGCCGGCCACGATGCGTCCGGCGTAGAGGA

TCGAGATCTCGATCCCGCGAAATTAATACGACTCACTATAGGGGAATTGTGAGCGGATAACAATTCCCCTCTAGAAATAA

TTTTGATTTAACTTTAAGAAGGAGATATACCATGAAACATCACCATCACCATCACCCCATGAGCGATTACGACATCCCCA

CTACTGAGAATCTTTATTTTCAGGGCGCCATGGCGATGCTGCCTCCGAGCGATAGCAAAGATCCGCGTCCGTGGCAGATT

CTGAGCCAGGCACTGGGTTTTCCGAATTATGATCAAGAACTGTGGTGGCAGAATACCGCAGAAACCCTGAATCGTGTTCT

GGAACAGTGTGATTATAGCGTTCATCTGCAGTATAAATACCTGGCCTTCTACCACAAATATATCCTGCCGAGCCTGGGTC

CGTTTCGTCGTCCGGGTGTTGAACCGGAATATATCAGCGGTCTGAGTCATGGTGGTCATCCGCTGCAAATTAGCGTGAAA

ATTGATAAAAGCAAGACCATTTGTCGTCTGGGTCTGCAGGCAATTGGTCCGCTGGCAGGCACCGCACGTGATCCGCTGAA

TAGCTTTGGTGATCGTGAACTGCTGAAAAATCTGGCAACCCTGCTGCCGCATGTTGATCTGCGTCTGTTTGATCATTTTA

ATGCACAGGTTGGTCTGGATCGTGCACAGTGTGCAGTTGCAACCACCAAACTGATTAAAGAAAGCCATAATATTGTGTGC

ACCAGCCTGGATCTGAAAGATGGTGAAGTTATTCCGAAAGTGTACTTTAGCACCATTCCTAAAGGTCTGGTTACCGAAAC

ACCGCTGTTCGATCTGACCTTTGCAGCAATTGAGCAGATGGAAGTGTATCATAAAGATGCACCGCTGCGTACCGCACTGA

GCAGCCTGAAAGATTTTCTGCGTCCGCGTGTTCCGACCGATGCAAGCATTACCCCTCCGCTGACCGGTCTGATTGGTGTT

GATTGTATTGATCCGATGCTGAGCCGTCTGAAAGTTTATCTGGCCACCTTTCGTATGGATCTGAGCCTGATTCGTGATTA

TTGGACCTTAGGTGGTCTGCTGACCGATGCCGGTACAATGAAAGGCCTGGAAATGGTTGAAACCCTGGCAAAAACACTGA

AACTGGGTGATGAAGCATGTGAAACACTGGATGCAGAACGTCTGCCGTTTGGTATTAACTATGCAATGAAACCGGGTACA

GCAGAACTGGCACCGCCTCAGATCTATTTTCCGCTGCTGGGTATTAATGATGGCTTTATTGCAGATGCCCTGGTGGAATT

TTTTCAGTATATGGGTTGGGAAGATCAGGCCAATCGTTATAAAGATGAACTGAAAGCCAAATTTCCGAACGTGGATATTA

GCCAGACCAAAAATGTTCATCGTTGGCTGGGTGTTGCATATAGCGAAACCAAAGGTCCGAGCATGAACATCTATTATGAT

GTTGTTGCAGGTAATGTGGCACGCGTTTAACTCGAGCACCACCACCACCACCACTGAGATCCGGCTGCTAACAAAGCCCG

AAAGGAAGCTGAGTTGGCTGCTGCCACCGCTGAGCAATAACTAGCATAACCCCTTGGGGCCTCTAAACGGGTCTTGAGGG

GTTTTTTGCTGAAAGGAGGAACTATATCCGGAT

>pET-M11-AtaPTY344S (Genbank: OQ725937)

TGGCGAATGGGACGCGCCCTGTAGCGGCGCATTAAGCGCGGCGGGTGTGGTGGTTACGCGCAGCGTGACCGCTACACTTG

CCAGCGCCCTAGCGCCCGCTCCTTTCGCTTTCTTCCCTTCCTTTCTCGCCACGTTCGCCGGCTTTCCCCGTCAAGCTCTA

AATCGGGGGCTCCCTTTAGGGTTCCGATTTAGTGCTTTACGGCACCTCGACCCCAAAAAACTTGATTAGGGTGATGGTTC

ACGTAGTGGGCCATCGCCCTGATAGACGGTTTTTCGCCCTTTGACGTTGGAGTCCACGTTCTTTAATAGTGGACTCTTGT

TCCAAACTGGAACAACACTCAACCCTATCTCGGTCTATTCTTTTGATTTATAAGGGATTTTGCCGATTTCGGCCTATTGG

TTAAAAAATGAGCTGATTTAACAAAAATTTAACGCGAATTTTAACAAAATATTAACGTTTACAATTTCAGGTGGCACTTT

TCGGGGAAATGTGCGCGGAACCCCTATTTGTTTATTTTTCTAAATACATTCAAATATGTATCCGCTCATGAATTAATTCT

TAGAAAAACTCATCGAGCATCAAATGAAACTGCAATTTATTCATATCAGGATTATCAATACCATATTTTTGAAAAAGCCG

TTTCTGTAATGAAGGAGAAAACTCACCGAGGCAGTTCCATAGGATGGCAAGATCCTGGTATCGGTCTGCGATTCCGACTC

GTCCAACATCAATACAACCTATTAATTTCCCCTCGTCAAAAATAAGGTTATCAAGTGAGAAATCACCATGAGTGACGACT

GAATCCGGTGAGAATGGCAAAAGTTTATGCATTTCTTTCCAGACTTGTTCAACAGGCCAGCCATTACGCTCGTCATCAAA

ATCACTCGCATCAACCAAACCGTTATTCATTCGTGATTGCGCCTGAGCGAGACGAAATACGCGATCGCTGTTAAAAGGAC

AATTACAAACAGGAATCGAATGCAACCGGCGCAGGAACACTGCCAGCGCATCAACAATATTTTCACCTGAATCAGGATAT

TCTTCTAATACCTGGAATGCTGTTTTCCCGGGGATCGCAGTGGTGAGTAACCATGCATCATCAGGAGTACGGATAAAATG

CTTGATGGTCGGAAGAGGCATAAATTCCGTCAGCCAGTTTAGTCTGACCATCTCATCTGTAACATCATTGGCAACGCTAC

CTTTGCCATGTTTCAGAAACAACTCTGGCGCATCGGGCTTCCCATACAATCGATAGATTGTCGCACCTGATTGCCCGACA

TTATCGCGAGCCCATTTATACCCATATAAATCAGCATCCATGTTGGAATTTAATCGCGGCCTAGAGCAAGACGTTTCCCG

TTGAATATGGCTCATAACACCCCTTGTATTACTGTTTATGTAAGCAGACAGTTTTATTGTTCATGACCAAAATCCCTTAA

CGTGAGTTTTCGTTCCACTGAGCGTCAGACCCCGTAGAAAAGATCAAAGGATCTTCTTGAGATCCTTTTTTTCTGCGCGT

AATCTGCTGCTTGCAAACAAAAAAACCACCGCTACCAGCGGTGGTTTGTTTGCCGGATCAAGAGCTACCAACTCTTTTTC

CGAAGGTAACTGGCTTCAGCAGAGCGCAGATACCAAATACTGTCCTTCTAGTGTAGCCGTAGTTAGGCCACCACTTCAAG

AACTCTGTAGCACCGCCTACATACCTCGCTCTGCTAATCCTGTTACCAGTGGCTGCTGCCAGTGGCGATAAGTCGTGTCT

TACCGGGTTGGACTCAAGACGATAGTTACCGGATAAGGCGCAGCGGTCGGGCTGAACGGGGGGTTCGTGCACACAGCCCA

GCTTGGAGCGAACGACCTACACCGAACTGAGATACCTACAGCGTGAGCTATGAGAAAGCGCCACGCTTCCCGAAGGGAGA

AAGGCGGACAGGTATCCGGTAAGCGGCAGGGTCGGAACAGGAGAGCGCACGAGGGAGCTTCCAGGGGGAAACGCCTGGTA

TCTTTATAGTCCTGTCGGGTTTCGCCACCTCTGACTTGAGCGTCGATTTTTGTGATGCTCGTCAGGGGGGCGGAGCCTAT

GGAAAAACGCCAGCAACGCGGCCTTTTTACGGTTCCTGGCCTTTTGCTGGCCTTTTGCTCACATGTTCTTTCCTGCGTTA

TCCCCTGATTCTGTGGATAACCGTATTACCGCCTTTGAGTGAGCTGATACCGCTCGCCGCAGCCGAACGACCGAGCGCAG

CGAGTCAGTGAGCGAGGAAGCGGAAGAGCGCCTGATGCGGTATTTTCTCCTTACGCATCTGTGCGGTATTTCACACCGCA

TATATGGTGCACTCTCAGTACAATCTGCTCTGATGCCGCATAGTTAAGCCAGTATACACTCCGCTATCGCTACGTGACTG

GGTCATGGCTGCGCCCCGACACCCGCCAACACCCGCTGACGCGCCCTGACGGGCTTGTCTGCTCCCGGCATCCGCTTACA

GACAAGCTGTGACCGTCTCCGGGAGCTGCATGTGTCAGAGGTTTTCACCGTCATCACCGAAACGCGCGAGGCAGCTGCGG

TAAAGCTCATCAGCGTGGTCGTGAAGCGATTCACAGATGTCTGCCTGTTCATCCGCGTCCAGCTCGTTGAGTTTCTCCAG

AAGCGTTAATGTCTGGCTTCTGATAAAGCGGGCCATGTTAAGGGCGGTTTTTTCCTGTTTGGTCACTGATGCCTCCGTGT

AAGGGGGATTTCTGTTCATGGGGGTAATGATACCGATGAAACGAGAGAGGATGCTCACGATACGGGTTACTGATGATGAA

CATGCCCGGTTACTGGAACGTTGTGAGGGTAAACAACTGGCGGTATGGATGCGGCGGGACCAGAGAAAAATCACTCAGGG

TCAATGCCAGCGCTTCGTTAATACAGATGTAGGTGTTCCACAGGGTAGCCAGCAGCATCCTGCGATGCAGATCCGGAACA

TAATGGTGCAGGGCGCTGACTTCCGCGTTTCCAGACTTTACGAAACACGGAAACCGAAGACCATTCATGTTGTTGCTCAG

GTCGCAGACGTTTTGCAGCAGCAGTCGCTTCACGTTCGCTCGCGTATCGGTGATTCATTCTGCTAACCAGTAAGGCAACC

CCGCCAGCCTAGCCGGGTCCTCAACGACAGGAGCACGATCATGCGCACCCGTGGGGCCGCCATGCCGGCGATAATGGCCT

GCTTCTCGCCGAAACGTTTGGTGGCGGGACCAGTGACGAAGGCTTGAGCGAGGGCGTGCAAGATTCCGAATACCGCAAGC

GACAGGCCGATCATCGTCGCGCTCCAGCGAAAGCGGTCCTCGCCGAAAATGACCCAGAGCGCTGCCGGCACCTGTCCTAC

GAGTTGCATGATAAAGAAGACAGTCATAAGTGCGGCGACGATAGTCATGCCCCGCGCCCACCGGAAGGAGCTGACTGGGT

TGAAGGCTCTCAAGGGCATCGGTCGAGATCCCGGTGCCTAATGAGTGAGCTAACTTACATTAATTGCGTTGCGCTCACTG

CCCGCTTTCCAGTCGGGAAACCTGTCGTGCCAGCTGCATTAATGAATCGGCCAACGCGCGGGGAGAGGCGGTTTGCGTAT

TGGGCGCCAGGGTGGTTTTTCTTTTCACCAGTGAGACGGGCAACAGCTGATTGCCCTTCACCGCCTGGCCCTGAGAGAGT

TGCAGCAAGCGGTCCACGCTGGTTTGCCCCAGCAGGCGAAAATCCTGTTTGATGGTGGTTAACGGCGGGATATAACATGA

GCTGTCTTCGGTATCGTCGTATCCCACTACCGAGATATCCGCACCAACGCGCAGCCCGGACTCGGTAATGGCGCGCATTG

CGCCCAGCGCCATCTGATCGTTGGCAACCAGCATCGCAGTGGGAACGATGCCCTCATTCAGCATTTGCATGGTTTGTTGA

AAACCGGACATGGCACTCCAGTCGCCTTCCCGTTCCGCTATCGGCTGAATTTGATTGCGAGTGAGATATTTATGCCAGCC

AGCCAGACGCAGACGCGCCGAGACAGAACTTAATGGGCCCGCTAACAGCGCGATTTGCTGGTGACCCAATGCGACCAGAT

GCTCCACGCCCAGTCGCGTACCGTCTTCATGGGAGAAAATAATACTGTTGATGGGTGTCTGGTCAGAGACATCAAGAAAT

AACGCCGGAACATTAGTGCAGGCAGCTTCCACAGCAATGGCATCCTGGTCATCCAGCGGATAGTTAATGATCAGCCCACT

GACGCGTTGCGCGAGAAGATTGTGCACCGCCGCTTTACAGGCTTCGACGCCGCTTCGTTCTACCATCGACACCACCACGC

TGGCACCCAGTTGATCGGCGCGAGATTTAATCGCCGCGACAATTTGCGACGGCGCGTGCAGGGCCAGACTGGAGGTGGCA

ACGCCAATCAGCAACGACTGTTTGCCCGCCAGTTGTTGTGCCACGCGGTTGGGAATGTAATTCAGCTCCGCCATCGCCGC

TTCCACTTTTTCCCGCGTTTTCGCAGAAACGTGGCTGGCCTGGTTCACCACGCGGGAAACGGTCTGATAAGAGACACCGG

CATACTCTGCGACATCGTATAACGTTACTGGTTTCACATTCACCACCCTGAATTGACTCTCTTCCGGGCGCTATCATGCC

ATACCGCGAAAGGTTTTGCGCCATTCGATGGTGTCCGGGATCTCGACGCTCTCCCTTATGCGACTCCTGCATTAGGAAGC

AGCCCAGTAGTAGGTTGAGGCCGTTGAGCACCGCCGCCGCAAGGAATGGTGCATGCAAGGAGATGGCGCCCAACAGTCCC

CCGGCCACGGGGCCTGCCACCATACCCACGCCGAAACAAGCGCTCATGAGCCCGAAGTGGCGAGCCCGATCTTCCCCATC

GGTGATGTCGGCGATATAGGCGCCAGCAACCGCACCTGTGGCGCCGGTGATGCCGGCCACGATGCGTCCGGCGTAGAGGA

TCGAGATCTCGATCCCGCGAAATTAATACGACTCACTATAGGGGAATTGTGAGCGGATAACAATTCCCCTCTAGAAATAA

TTTTGATTTAACTTTAAGAAGGAGATATACCATGAAACATCACCATCACCATCACCCCATGAGCGATTACGACATCCCCA

CTACTGAGAATCTTTATTTTCAGGGCGCCATGGCGATGCTGCCTCCGAGCGATAGCAAAGATCCGCGTCCGTGGCAGATT

CTGAGCCAGGCACTGGGTTTTCCGAATTATGATCAAGAACTGTGGTGGCAGAATACCGCAGAAACCCTGAATCGTGTTCT

GGAACAGTGTGATTATAGCGTTCATCTGCAGTATAAATACCTGGCCTTCTACCACAAATATATCCTGCCGAGCCTGGGTC

CGTTTCGTCGTCCGGGTGTTGAACCGGAATATATCAGCGGTCTGAGTCATGGTGGTCATCCGCTGGAAATTAGCGTGAAA

ATTGATAAAAGCAAGACCATTTGTCGTCTGGGTCTGCAGGCAATTGGTCCGCTGGCAGGCACCGCACGTGATCCGCTGAA

TAGCTTTGGTGATCGTGAACTGCTGAAAAATCTGGCAACCCTGCTGCCGCATGTTGATCTGCGTCTGTTTGATCATTTTA

ATGCACAGGTTGGTCTGGATCGTGCACAGTGTGCAGTTGCAACCACCAAACTGATTAAAGAAAGCCATAATATTGTGTGC

ACCAGCCTGGATCTGAAAGATGGTGAAGTTATTCCGAAAGTGTACTTTAGCACCATTCCTAAAGGTCTGGTTACCGAAAC

ACCGCTGTTCGATCTGACCTTTGCAGCAATTGAGCAGATGGAAGTGTATCATAAAGATGCACCGCTGCGTACCGCACTGA

GCAGCCTGAAAGATTTTCTGCGTCCGCGTGTTCCGACCGATGCAAGCATTACCCCTCCGCTGACCGGTCTGATTGGTGTT

GATTGTATTGATCCGATGCTGAGCCGTCTGAAAGTTTATCTGGCCACCTTTCGTATGGATCTGAGCCTGATTCGTGATTA

TTGGACCTTAGGTGGTCTGCTGACCGATGCCGGTACAATGAAAGGCCTGGAAATGGTTGAAACCCTGGCAAAAACACTGA

AACTGGGTGATGAAGCATGTGAAACACTGGATGCAGAACGTCTGCCGTTTGGTATTAACTATGCAATGAAACCGGGTACA

GCAGAACTGGCACCGCCTCAGATCAGCTTTCCGCTGCTGGGTATTAATGATGGCTTTATTGCAGATGCCCTGGTGGAATT

TTTTCAGTATATGGGTTGGGAAGATCAGGCCAATCGTTATAAAGATGAACTGAAAGCCAAATTTCCGAACGTGGATATTA

GCCAGACCAAAAATGTTCATCGTTGGCTGGGTGTTGCATATAGCGAAACCAAAGGTCCGAGCATGAACATCTATTATGAT

GTTGTTGCAGGTAATGTGGCACGCGTTTAACTCGAGCACCACCACCACCACCACTGAGATCCGGCTGCTAACAAAGCCCG

AAAGGAAGCTGAGTTGGCTGCTGCCACCGCTGAGCAATAACTAGCATAACCCCTTGGGGCCTCTAAACGGGTCTTGAGGG

GTTTTTTGCTGAAAGGAGGAACTATATCCGGAT

>pET-M11-AtaPTY413S (Genbank: OQ725938)

TGGCGAATGGGACGCGCCCTGTAGCGGCGCATTAAGCGCGGCGGGTGTGGTGGTTACGCGCAGCGTGACCGCTACACTTG

CCAGCGCCCTAGCGCCCGCTCCTTTCGCTTTCTTCCCTTCCTTTCTCGCCACGTTCGCCGGCTTTCCCCGTCAAGCTCTA

AATCGGGGGCTCCCTTTAGGGTTCCGATTTAGTGCTTTACGGCACCTCGACCCCAAAAAACTTGATTAGGGTGATGGTTC

ACGTAGTGGGCCATCGCCCTGATAGACGGTTTTTCGCCCTTTGACGTTGGAGTCCACGTTCTTTAATAGTGGACTCTTGT

TCCAAACTGGAACAACACTCAACCCTATCTCGGTCTATTCTTTTGATTTATAAGGGATTTTGCCGATTTCGGCCTATTGG

TTAAAAAATGAGCTGATTTAACAAAAATTTAACGCGAATTTTAACAAAATATTAACGTTTACAATTTCAGGTGGCACTTT

TCGGGGAAATGTGCGCGGAACCCCTATTTGTTTATTTTTCTAAATACATTCAAATATGTATCCGCTCATGAATTAATTCT

TAGAAAAACTCATCGAGCATCAAATGAAACTGCAATTTATTCATATCAGGATTATCAATACCATATTTTTGAAAAAGCCG

TTTCTGTAATGAAGGAGAAAACTCACCGAGGCAGTTCCATAGGATGGCAAGATCCTGGTATCGGTCTGCGATTCCGACTC

GTCCAACATCAATACAACCTATTAATTTCCCCTCGTCAAAAATAAGGTTATCAAGTGAGAAATCACCATGAGTGACGACT

GAATCCGGTGAGAATGGCAAAAGTTTATGCATTTCTTTCCAGACTTGTTCAACAGGCCAGCCATTACGCTCGTCATCAAA

ATCACTCGCATCAACCAAACCGTTATTCATTCGTGATTGCGCCTGAGCGAGACGAAATACGCGATCGCTGTTAAAAGGAC

AATTACAAACAGGAATCGAATGCAACCGGCGCAGGAACACTGCCAGCGCATCAACAATATTTTCACCTGAATCAGGATAT

TCTTCTAATACCTGGAATGCTGTTTTCCCGGGGATCGCAGTGGTGAGTAACCATGCATCATCAGGAGTACGGATAAAATG

CTTGATGGTCGGAAGAGGCATAAATTCCGTCAGCCAGTTTAGTCTGACCATCTCATCTGTAACATCATTGGCAACGCTAC

CTTTGCCATGTTTCAGAAACAACTCTGGCGCATCGGGCTTCCCATACAATCGATAGATTGTCGCACCTGATTGCCCGACA

TTATCGCGAGCCCATTTATACCCATATAAATCAGCATCCATGTTGGAATTTAATCGCGGCCTAGAGCAAGACGTTTCCCG

TTGAATATGGCTCATAACACCCCTTGTATTACTGTTTATGTAAGCAGACAGTTTTATTGTTCATGACCAAAATCCCTTAA

CGTGAGTTTTCGTTCCACTGAGCGTCAGACCCCGTAGAAAAGATCAAAGGATCTTCTTGAGATCCTTTTTTTCTGCGCGT

AATCTGCTGCTTGCAAACAAAAAAACCACCGCTACCAGCGGTGGTTTGTTTGCCGGATCAAGAGCTACCAACTCTTTTTC

CGAAGGTAACTGGCTTCAGCAGAGCGCAGATACCAAATACTGTCCTTCTAGTGTAGCCGTAGTTAGGCCACCACTTCAAG

AACTCTGTAGCACCGCCTACATACCTCGCTCTGCTAATCCTGTTACCAGTGGCTGCTGCCAGTGGCGATAAGTCGTGTCT

TACCGGGTTGGACTCAAGACGATAGTTACCGGATAAGGCGCAGCGGTCGGGCTGAACGGGGGGTTCGTGCACACAGCCCA

GCTTGGAGCGAACGACCTACACCGAACTGAGATACCTACAGCGTGAGCTATGAGAAAGCGCCACGCTTCCCGAAGGGAGA

AAGGCGGACAGGTATCCGGTAAGCGGCAGGGTCGGAACAGGAGAGCGCACGAGGGAGCTTCCAGGGGGAAACGCCTGGTA

TCTTTATAGTCCTGTCGGGTTTCGCCACCTCTGACTTGAGCGTCGATTTTTGTGATGCTCGTCAGGGGGGCGGAGCCTAT

GGAAAAACGCCAGCAACGCGGCCTTTTTACGGTTCCTGGCCTTTTGCTGGCCTTTTGCTCACATGTTCTTTCCTGCGTTA

TCCCCTGATTCTGTGGATAACCGTATTACCGCCTTTGAGTGAGCTGATACCGCTCGCCGCAGCCGAACGACCGAGCGCAG

CGAGTCAGTGAGCGAGGAAGCGGAAGAGCGCCTGATGCGGTATTTTCTCCTTACGCATCTGTGCGGTATTTCACACCGCA

TATATGGTGCACTCTCAGTACAATCTGCTCTGATGCCGCATAGTTAAGCCAGTATACACTCCGCTATCGCTACGTGACTG

GGTCATGGCTGCGCCCCGACACCCGCCAACACCCGCTGACGCGCCCTGACGGGCTTGTCTGCTCCCGGCATCCGCTTACA

GACAAGCTGTGACCGTCTCCGGGAGCTGCATGTGTCAGAGGTTTTCACCGTCATCACCGAAACGCGCGAGGCAGCTGCGG

TAAAGCTCATCAGCGTGGTCGTGAAGCGATTCACAGATGTCTGCCTGTTCATCCGCGTCCAGCTCGTTGAGTTTCTCCAG

AAGCGTTAATGTCTGGCTTCTGATAAAGCGGGCCATGTTAAGGGCGGTTTTTTCCTGTTTGGTCACTGATGCCTCCGTGT

AAGGGGGATTTCTGTTCATGGGGGTAATGATACCGATGAAACGAGAGAGGATGCTCACGATACGGGTTACTGATGATGAA

CATGCCCGGTTACTGGAACGTTGTGAGGGTAAACAACTGGCGGTATGGATGCGGCGGGACCAGAGAAAAATCACTCAGGG

TCAATGCCAGCGCTTCGTTAATACAGATGTAGGTGTTCCACAGGGTAGCCAGCAGCATCCTGCGATGCAGATCCGGAACA

TAATGGTGCAGGGCGCTGACTTCCGCGTTTCCAGACTTTACGAAACACGGAAACCGAAGACCATTCATGTTGTTGCTCAG

GTCGCAGACGTTTTGCAGCAGCAGTCGCTTCACGTTCGCTCGCGTATCGGTGATTCATTCTGCTAACCAGTAAGGCAACC

CCGCCAGCCTAGCCGGGTCCTCAACGACAGGAGCACGATCATGCGCACCCGTGGGGCCGCCATGCCGGCGATAATGGCCT

GCTTCTCGCCGAAACGTTTGGTGGCGGGACCAGTGACGAAGGCTTGAGCGAGGGCGTGCAAGATTCCGAATACCGCAAGC

GACAGGCCGATCATCGTCGCGCTCCAGCGAAAGCGGTCCTCGCCGAAAATGACCCAGAGCGCTGCCGGCACCTGTCCTAC

GAGTTGCATGATAAAGAAGACAGTCATAAGTGCGGCGACGATAGTCATGCCCCGCGCCCACCGGAAGGAGCTGACTGGGT

TGAAGGCTCTCAAGGGCATCGGTCGAGATCCCGGTGCCTAATGAGTGAGCTAACTTACATTAATTGCGTTGCGCTCACTG

CCCGCTTTCCAGTCGGGAAACCTGTCGTGCCAGCTGCATTAATGAATCGGCCAACGCGCGGGGAGAGGCGGTTTGCGTAT

TGGGCGCCAGGGTGGTTTTTCTTTTCACCAGTGAGACGGGCAACAGCTGATTGCCCTTCACCGCCTGGCCCTGAGAGAGT

TGCAGCAAGCGGTCCACGCTGGTTTGCCCCAGCAGGCGAAAATCCTGTTTGATGGTGGTTAACGGCGGGATATAACATGA

GCTGTCTTCGGTATCGTCGTATCCCACTACCGAGATATCCGCACCAACGCGCAGCCCGGACTCGGTAATGGCGCGCATTG

CGCCCAGCGCCATCTGATCGTTGGCAACCAGCATCGCAGTGGGAACGATGCCCTCATTCAGCATTTGCATGGTTTGTTGA

AAACCGGACATGGCACTCCAGTCGCCTTCCCGTTCCGCTATCGGCTGAATTTGATTGCGAGTGAGATATTTATGCCAGCC

AGCCAGACGCAGACGCGCCGAGACAGAACTTAATGGGCCCGCTAACAGCGCGATTTGCTGGTGACCCAATGCGACCAGAT

GCTCCACGCCCAGTCGCGTACCGTCTTCATGGGAGAAAATAATACTGTTGATGGGTGTCTGGTCAGAGACATCAAGAAAT

AACGCCGGAACATTAGTGCAGGCAGCTTCCACAGCAATGGCATCCTGGTCATCCAGCGGATAGTTAATGATCAGCCCACT

GACGCGTTGCGCGAGAAGATTGTGCACCGCCGCTTTACAGGCTTCGACGCCGCTTCGTTCTACCATCGACACCACCACGC

TGGCACCCAGTTGATCGGCGCGAGATTTAATCGCCGCGACAATTTGCGACGGCGCGTGCAGGGCCAGACTGGAGGTGGCA

ACGCCAATCAGCAACGACTGTTTGCCCGCCAGTTGTTGTGCCACGCGGTTGGGAATGTAATTCAGCTCCGCCATCGCCGC

TTCCACTTTTTCCCGCGTTTTCGCAGAAACGTGGCTGGCCTGGTTCACCACGCGGGAAACGGTCTGATAAGAGACACCGG

CATACTCTGCGACATCGTATAACGTTACTGGTTTCACATTCACCACCCTGAATTGACTCTCTTCCGGGCGCTATCATGCC

ATACCGCGAAAGGTTTTGCGCCATTCGATGGTGTCCGGGATCTCGACGCTCTCCCTTATGCGACTCCTGCATTAGGAAGC

AGCCCAGTAGTAGGTTGAGGCCGTTGAGCACCGCCGCCGCAAGGAATGGTGCATGCAAGGAGATGGCGCCCAACAGTCCC

CCGGCCACGGGGCCTGCCACCATACCCACGCCGAAACAAGCGCTCATGAGCCCGAAGTGGCGAGCCCGATCTTCCCCATC

GGTGATGTCGGCGATATAGGCGCCAGCAACCGCACCTGTGGCGCCGGTGATGCCGGCCACGATGCGTCCGGCGTAGAGGA

TCGAGATCTCGATCCCGCGAAATTAATACGACTCACTATAGGGGAATTGTGAGCGGATAACAATTCCCCTCTAGAAATAA

TTTTGATTTAACTTTAAGAAGGAGATATACCATGAAACATCACCATCACCATCACCCCATGAGCGATTACGACATCCCCA

CTACTGAGAATCTTTATTTTCAGGGCGCCATGGCGATGCTGCCTCCGAGCGATAGCAAAGATCCGCGTCCGTGGCAGATT

CTGAGCCAGGCACTGGGTTTTCCGAATTATGATCAAGAACTGTGGTGGCAGAATACCGCAGAAACCCTGAATCGTGTTCT

GGAACAGTGTGATTATAGCGTTCATCTGCAGTATAAATACCTGGCCTTCTACCACAAATATATCCTGCCGAGCCTGGGTC

CGTTTCGTCGTCCGGGTGTTGAACCGGAATATATCAGCGGTCTGAGTCATGGTGGTCATCCGCTGGAAATTAGCGTGAAA

ATTGATAAAAGCAAGACCATTTGTCGTCTGGGTCTGCAGGCAATTGGTCCGCTGGCAGGCACCGCACGTGATCCGCTGAA

TAGCTTTGGTGATCGTGAACTGCTGAAAAATCTGGCAACCCTGCTGCCGCATGTTGATCTGCGTCTGTTTGATCATTTTA

ATGCACAGGTTGGTCTGGATCGTGCACAGTGTGCAGTTGCAACCACCAAACTGATTAAAGAAAGCCATAATATTGTGTGC

ACCAGCCTGGATCTGAAAGATGGTGAAGTTATTCCGAAAGTGTACTTTAGCACCATTCCTAAAGGTCTGGTTACCGAAAC

ACCGCTGTTCGATCTGACCTTTGCAGCAATTGAGCAGATGGAAGTGTATCATAAAGATGCACCGCTGCGTACCGCACTGA

GCAGCCTGAAAGATTTTCTGCGTCCGCGTGTTCCGACCGATGCAAGCATTACCCCTCCGCTGACCGGTCTGATTGGTGTT

GATTGTATTGATCCGATGCTGAGCCGTCTGAAAGTTTATCTGGCCACCTTTCGTATGGATCTGAGCCTGATTCGTGATTA

TTGGACCTTAGGTGGTCTGCTGACCGATGCCGGTACAATGAAAGGCCTGGAAATGGTTGAAACCCTGGCAAAAACACTGA

AACTGGGTGATGAAGCATGTGAAACACTGGATGCAGAACGTCTGCCGTTTGGTATTAACTATGCAATGAAACCGGGTACA

GCAGAACTGGCACCGCCTCAGATCTATTTTCCGCTGCTGGGTATTAATGATGGCTTTATTGCAGATGCCCTGGTGGAATT

TTTTCAGTATATGGGTTGGGAAGATCAGGCCAATCGTTATAAAGATGAACTGAAAGCCAAATTTCCGAACGTGGATATTA

GCCAGACCAAAAATGTTCATCGTTGGCTGGGTGTTGCATATAGCGAAACCAAAGGTCCGAGCATGAACATCAGCTATGAT

GTTGTTGCAGGTAATGTGGCACGCGTTTAACTCGAGCACCACCACCACCACCACTGAGATCCGGCTGCTAACAAAGCCCG

AAAGGAAGCTGAGTTGGCTGCTGCCACCGCTGAGCAATAACTAGCATAACCCCTTGGGGCCTCTAAACGGGTCTTGAGGG

GTTTTTTGCTGAAAGGAGGAACTATATCCGGAT

>pET-M11-AtaPTY344S/Y413S (Genbank: OQ725939)

TGGCGAATGGGACGCGCCCTGTAGCGGCGCATTAAGCGCGGCGGGTGTGGTGGTTACGCGCAGCGTGACCGCTACACTTG

CCAGCGCCCTAGCGCCCGCTCCTTTCGCTTTCTTCCCTTCCTTTCTCGCCACGTTCGCCGGCTTTCCCCGTCAAGCTCTA

AATCGGGGGCTCCCTTTAGGGTTCCGATTTAGTGCTTTACGGCACCTCGACCCCAAAAAACTTGATTAGGGTGATGGTTC

ACGTAGTGGGCCATCGCCCTGATAGACGGTTTTTCGCCCTTTGACGTTGGAGTCCACGTTCTTTAATAGTGGACTCTTGT

TCCAAACTGGAACAACACTCAACCCTATCTCGGTCTATTCTTTTGATTTATAAGGGATTTTGCCGATTTCGGCCTATTGG

TTAAAAAATGAGCTGATTTAACAAAAATTTAACGCGAATTTTAACAAAATATTAACGTTTACAATTTCAGGTGGCACTTT

TCGGGGAAATGTGCGCGGAACCCCTATTTGTTTATTTTTCTAAATACATTCAAATATGTATCCGCTCATGAATTAATTCT

TAGAAAAACTCATCGAGCATCAAATGAAACTGCAATTTATTCATATCAGGATTATCAATACCATATTTTTGAAAAAGCCG

TTTCTGTAATGAAGGAGAAAACTCACCGAGGCAGTTCCATAGGATGGCAAGATCCTGGTATCGGTCTGCGATTCCGACTC

GTCCAACATCAATACAACCTATTAATTTCCCCTCGTCAAAAATAAGGTTATCAAGTGAGAAATCACCATGAGTGACGACT

GAATCCGGTGAGAATGGCAAAAGTTTATGCATTTCTTTCCAGACTTGTTCAACAGGCCAGCCATTACGCTCGTCATCAAA

ATCACTCGCATCAACCAAACCGTTATTCATTCGTGATTGCGCCTGAGCGAGACGAAATACGCGATCGCTGTTAAAAGGAC

AATTACAAACAGGAATCGAATGCAACCGGCGCAGGAACACTGCCAGCGCATCAACAATATTTTCACCTGAATCAGGATAT

TCTTCTAATACCTGGAATGCTGTTTTCCCGGGGATCGCAGTGGTGAGTAACCATGCATCATCAGGAGTACGGATAAAATG

CTTGATGGTCGGAAGAGGCATAAATTCCGTCAGCCAGTTTAGTCTGACCATCTCATCTGTAACATCATTGGCAACGCTAC

CTTTGCCATGTTTCAGAAACAACTCTGGCGCATCGGGCTTCCCATACAATCGATAGATTGTCGCACCTGATTGCCCGACA

TTATCGCGAGCCCATTTATACCCATATAAATCAGCATCCATGTTGGAATTTAATCGCGGCCTAGAGCAAGACGTTTCCCG

TTGAATATGGCTCATAACACCCCTTGTATTACTGTTTATGTAAGCAGACAGTTTTATTGTTCATGACCAAAATCCCTTAA

CGTGAGTTTTCGTTCCACTGAGCGTCAGACCCCGTAGAAAAGATCAAAGGATCTTCTTGAGATCCTTTTTTTCTGCGCGT

AATCTGCTGCTTGCAAACAAAAAAACCACCGCTACCAGCGGTGGTTTGTTTGCCGGATCAAGAGCTACCAACTCTTTTTC

CGAAGGTAACTGGCTTCAGCAGAGCGCAGATACCAAATACTGTCCTTCTAGTGTAGCCGTAGTTAGGCCACCACTTCAAG

AACTCTGTAGCACCGCCTACATACCTCGCTCTGCTAATCCTGTTACCAGTGGCTGCTGCCAGTGGCGATAAGTCGTGTCT

TACCGGGTTGGACTCAAGACGATAGTTACCGGATAAGGCGCAGCGGTCGGGCTGAACGGGGGGTTCGTGCACACAGCCCA

GCTTGGAGCGAACGACCTACACCGAACTGAGATACCTACAGCGTGAGCTATGAGAAAGCGCCACGCTTCCCGAAGGGAGA

AAGGCGGACAGGTATCCGGTAAGCGGCAGGGTCGGAACAGGAGAGCGCACGAGGGAGCTTCCAGGGGGAAACGCCTGGTA

TCTTTATAGTCCTGTCGGGTTTCGCCACCTCTGACTTGAGCGTCGATTTTTGTGATGCTCGTCAGGGGGGCGGAGCCTAT

GGAAAAACGCCAGCAACGCGGCCTTTTTACGGTTCCTGGCCTTTTGCTGGCCTTTTGCTCACATGTTCTTTCCTGCGTTA

TCCCCTGATTCTGTGGATAACCGTATTACCGCCTTTGAGTGAGCTGATACCGCTCGCCGCAGCCGAACGACCGAGCGCAG

CGAGTCAGTGAGCGAGGAAGCGGAAGAGCGCCTGATGCGGTATTTTCTCCTTACGCATCTGTGCGGTATTTCACACCGCA

TATATGGTGCACTCTCAGTACAATCTGCTCTGATGCCGCATAGTTAAGCCAGTATACACTCCGCTATCGCTACGTGACTG

GGTCATGGCTGCGCCCCGACACCCGCCAACACCCGCTGACGCGCCCTGACGGGCTTGTCTGCTCCCGGCATCCGCTTACA

GACAAGCTGTGACCGTCTCCGGGAGCTGCATGTGTCAGAGGTTTTCACCGTCATCACCGAAACGCGCGAGGCAGCTGCGG

TAAAGCTCATCAGCGTGGTCGTGAAGCGATTCACAGATGTCTGCCTGTTCATCCGCGTCCAGCTCGTTGAGTTTCTCCAG

AAGCGTTAATGTCTGGCTTCTGATAAAGCGGGCCATGTTAAGGGCGGTTTTTTCCTGTTTGGTCACTGATGCCTCCGTGT

AAGGGGGATTTCTGTTCATGGGGGTAATGATACCGATGAAACGAGAGAGGATGCTCACGATACGGGTTACTGATGATGAA

CATGCCCGGTTACTGGAACGTTGTGAGGGTAAACAACTGGCGGTATGGATGCGGCGGGACCAGAGAAAAATCACTCAGGG

TCAATGCCAGCGCTTCGTTAATACAGATGTAGGTGTTCCACAGGGTAGCCAGCAGCATCCTGCGATGCAGATCCGGAACA

TAATGGTGCAGGGCGCTGACTTCCGCGTTTCCAGACTTTACGAAACACGGAAACCGAAGACCATTCATGTTGTTGCTCAG

GTCGCAGACGTTTTGCAGCAGCAGTCGCTTCACGTTCGCTCGCGTATCGGTGATTCATTCTGCTAACCAGTAAGGCAACC

CCGCCAGCCTAGCCGGGTCCTCAACGACAGGAGCACGATCATGCGCACCCGTGGGGCCGCCATGCCGGCGATAATGGCCT

GCTTCTCGCCGAAACGTTTGGTGGCGGGACCAGTGACGAAGGCTTGAGCGAGGGCGTGCAAGATTCCGAATACCGCAAGC

GACAGGCCGATCATCGTCGCGCTCCAGCGAAAGCGGTCCTCGCCGAAAATGACCCAGAGCGCTGCCGGCACCTGTCCTAC

GAGTTGCATGATAAAGAAGACAGTCATAAGTGCGGCGACGATAGTCATGCCCCGCGCCCACCGGAAGGAGCTGACTGGGT

TGAAGGCTCTCAAGGGCATCGGTCGAGATCCCGGTGCCTAATGAGTGAGCTAACTTACATTAATTGCGTTGCGCTCACTG

CCCGCTTTCCAGTCGGGAAACCTGTCGTGCCAGCTGCATTAATGAATCGGCCAACGCGCGGGGAGAGGCGGTTTGCGTAT

TGGGCGCCAGGGTGGTTTTTCTTTTCACCAGTGAGACGGGCAACAGCTGATTGCCCTTCACCGCCTGGCCCTGAGAGAGT

TGCAGCAAGCGGTCCACGCTGGTTTGCCCCAGCAGGCGAAAATCCTGTTTGATGGTGGTTAACGGCGGGATATAACATGA

GCTGTCTTCGGTATCGTCGTATCCCACTACCGAGATATCCGCACCAACGCGCAGCCCGGACTCGGTAATGGCGCGCATTG

CGCCCAGCGCCATCTGATCGTTGGCAACCAGCATCGCAGTGGGAACGATGCCCTCATTCAGCATTTGCATGGTTTGTTGA

AAACCGGACATGGCACTCCAGTCGCCTTCCCGTTCCGCTATCGGCTGAATTTGATTGCGAGTGAGATATTTATGCCAGCC

AGCCAGACGCAGACGCGCCGAGACAGAACTTAATGGGCCCGCTAACAGCGCGATTTGCTGGTGACCCAATGCGACCAGAT

GCTCCACGCCCAGTCGCGTACCGTCTTCATGGGAGAAAATAATACTGTTGATGGGTGTCTGGTCAGAGACATCAAGAAAT

AACGCCGGAACATTAGTGCAGGCAGCTTCCACAGCAATGGCATCCTGGTCATCCAGCGGATAGTTAATGATCAGCCCACT

GACGCGTTGCGCGAGAAGATTGTGCACCGCCGCTTTACAGGCTTCGACGCCGCTTCGTTCTACCATCGACACCACCACGC

TGGCACCCAGTTGATCGGCGCGAGATTTAATCGCCGCGACAATTTGCGACGGCGCGTGCAGGGCCAGACTGGAGGTGGCA

ACGCCAATCAGCAACGACTGTTTGCCCGCCAGTTGTTGTGCCACGCGGTTGGGAATGTAATTCAGCTCCGCCATCGCCGC

TTCCACTTTTTCCCGCGTTTTCGCAGAAACGTGGCTGGCCTGGTTCACCACGCGGGAAACGGTCTGATAAGAGACACCGG

CATACTCTGCGACATCGTATAACGTTACTGGTTTCACATTCACCACCCTGAATTGACTCTCTTCCGGGCGCTATCATGCC

ATACCGCGAAAGGTTTTGCGCCATTCGATGGTGTCCGGGATCTCGACGCTCTCCCTTATGCGACTCCTGCATTAGGAAGC

AGCCCAGTAGTAGGTTGAGGCCGTTGAGCACCGCCGCCGCAAGGAATGGTGCATGCAAGGAGATGGCGCCCAACAGTCCC

CCGGCCACGGGGCCTGCCACCATACCCACGCCGAAACAAGCGCTCATGAGCCCGAAGTGGCGAGCCCGATCTTCCCCATC

GGTGATGTCGGCGATATAGGCGCCAGCAACCGCACCTGTGGCGCCGGTGATGCCGGCCACGATGCGTCCGGCGTAGAGGA

TCGAGATCTCGATCCCGCGAAATTAATACGACTCACTATAGGGGAATTGTGAGCGGATAACAATTCCCCTCTAGAAATAA

TTTTGATTTAACTTTAAGAAGGAGATATACCATGAAACATCACCATCACCATCACCCCATGAGCGATTACGACATCCCCA

CTACTGAGAATCTTTATTTTCAGGGCGCCATGGCGATGCTGCCTCCGAGCGATAGCAAAGATCCGCGTCCGTGGCAGATT

CTGAGCCAGGCACTGGGTTTTCCGAATTATGATCAAGAACTGTGGTGGCAGAATACCGCAGAAACCCTGAATCGTGTTCT

GGAACAGTGTGATTATAGCGTTCATCTGCAGTATAAATACCTGGCCTTCTACCACAAATATATCCTGCCGAGCCTGGGTC

CGTTTCGTCGTCCGGGTGTTGAACCGGAATATATCAGCGGTCTGAGTCATGGTGGTCATCCGCTGGAAATTAGCGTGAAA

ATTGATAAAAGCAAGACCATTTGTCGTCTGGGTCTGCAGGCAATTGGTCCGCTGGCAGGCACCGCACGTGATCCGCTGAA

TAGCTTTGGTGATCGTGAACTGCTGAAAAATCTGGCAACCCTGCTGCCGCATGTTGATCTGCGTCTGTTTGATCATTTTA

ATGCACAGGTTGGTCTGGATCGTGCACAGTGTGCAGTTGCAACCACCAAACTGATTAAAGAAAGCCATAATATTGTGTGC

ACCAGCCTGGATCTGAAAGATGGTGAAGTTATTCCGAAAGTGTACTTTAGCACCATTCCTAAAGGTCTGGTTACCGAAAC

ACCGCTGTTCGATCTGACCTTTGCAGCAATTGAGCAGATGGAAGTGTATCATAAAGATGCACCGCTGCGTACCGCACTGA

GCAGCCTGAAAGATTTTCTGCGTCCGCGTGTTCCGACCGATGCAAGCATTACCCCTCCGCTGACCGGTCTGATTGGTGTT

GATTGTATTGATCCGATGCTGAGCCGTCTGAAAGTTTATCTGGCCACCTTTCGTATGGATCTGAGCCTGATTCGTGATTA

TTGGACCTTAGGTGGTCTGCTGACCGATGCCGGTACAATGAAAGGCCTGGAAATGGTTGAAACCCTGGCAAAAACACTGA

AACTGGGTGATGAAGCATGTGAAACACTGGATGCAGAACGTCTGCCGTTTGGTATTAACTATGCAATGAAACCGGGTACA

GCAGAACTGGCACCGCCTCAGATCAGCTTTCCGCTGCTGGGTATTAATGATGGCTTTATTGCAGATGCCCTGGTGGAATT

TTTTCAGTATATGGGTTGGGAAGATCAGGCCAATCGTTATAAAGATGAACTGAAAGCCAAATTTCCGAACGTGGATATTA

GCCAGACCAAAAATGTTCATCGTTGGCTGGGTGTTGCATATAGCGAAACCAAAGGTCCGAGCATGAACATCAGCTATGAT

GTTGTTGCAGGTAATGTGGCACGCGTTTAACTCGAGCACCACCACCACCACCACTGAGATCCGGCTGCTAACAAAGCCCG

AAAGGAAGCTGAGTTGGCTGCTGCCACCGCTGAGCAATAACTAGCATAACCCCTTGGGGCCTCTAAACGGGTCTTGAGGG

GTTTTTTGCTGAAAGGAGGAACTATATCCGGAT

>pET-M11-AtaPT E91Q/Y344S (Genbank: OQ725940)

TGGCGAATGGGACGCGCCCTGTAGCGGCGCATTAAGCGCGGCGGGTGTGGTGGTTACGCGCAGCGTGACCGCTACACTTG

CCAGCGCCCTAGCGCCCGCTCCTTTCGCTTTCTTCCCTTCCTTTCTCGCCACGTTCGCCGGCTTTCCCCGTCAAGCTCTA

AATCGGGGGCTCCCTTTAGGGTTCCGATTTAGTGCTTTACGGCACCTCGACCCCAAAAAACTTGATTAGGGTGATGGTTC

ACGTAGTGGGCCATCGCCCTGATAGACGGTTTTTCGCCCTTTGACGTTGGAGTCCACGTTCTTTAATAGTGGACTCTTGT

TCCAAACTGGAACAACACTCAACCCTATCTCGGTCTATTCTTTTGATTTATAAGGGATTTTGCCGATTTCGGCCTATTGG

TTAAAAAATGAGCTGATTTAACAAAAATTTAACGCGAATTTTAACAAAATATTAACGTTTACAATTTCAGGTGGCACTTT

TCGGGGAAATGTGCGCGGAACCCCTATTTGTTTATTTTTCTAAATACATTCAAATATGTATCCGCTCATGAATTAATTCT

TAGAAAAACTCATCGAGCATCAAATGAAACTGCAATTTATTCATATCAGGATTATCAATACCATATTTTTGAAAAAGCCG

TTTCTGTAATGAAGGAGAAAACTCACCGAGGCAGTTCCATAGGATGGCAAGATCCTGGTATCGGTCTGCGATTCCGACTC

GTCCAACATCAATACAACCTATTAATTTCCCCTCGTCAAAAATAAGGTTATCAAGTGAGAAATCACCATGAGTGACGACT

GAATCCGGTGAGAATGGCAAAAGTTTATGCATTTCTTTCCAGACTTGTTCAACAGGCCAGCCATTACGCTCGTCATCAAA

ATCACTCGCATCAACCAAACCGTTATTCATTCGTGATTGCGCCTGAGCGAGACGAAATACGCGATCGCTGTTAAAAGGAC

AATTACAAACAGGAATCGAATGCAACCGGCGCAGGAACACTGCCAGCGCATCAACAATATTTTCACCTGAATCAGGATAT

TCTTCTAATACCTGGAATGCTGTTTTCCCGGGGATCGCAGTGGTGAGTAACCATGCATCATCAGGAGTACGGATAAAATG

CTTGATGGTCGGAAGAGGCATAAATTCCGTCAGCCAGTTTAGTCTGACCATCTCATCTGTAACATCATTGGCAACGCTAC

CTTTGCCATGTTTCAGAAACAACTCTGGCGCATCGGGCTTCCCATACAATCGATAGATTGTCGCACCTGATTGCCCGACA

TTATCGCGAGCCCATTTATACCCATATAAATCAGCATCCATGTTGGAATTTAATCGCGGCCTAGAGCAAGACGTTTCCCG

TTGAATATGGCTCATAACACCCCTTGTATTACTGTTTATGTAAGCAGACAGTTTTATTGTTCATGACCAAAATCCCTTAA

CGTGAGTTTTCGTTCCACTGAGCGTCAGACCCCGTAGAAAAGATCAAAGGATCTTCTTGAGATCCTTTTTTTCTGCGCGT

AATCTGCTGCTTGCAAACAAAAAAACCACCGCTACCAGCGGTGGTTTGTTTGCCGGATCAAGAGCTACCAACTCTTTTTC

CGAAGGTAACTGGCTTCAGCAGAGCGCAGATACCAAATACTGTCCTTCTAGTGTAGCCGTAGTTAGGCCACCACTTCAAG

AACTCTGTAGCACCGCCTACATACCTCGCTCTGCTAATCCTGTTACCAGTGGCTGCTGCCAGTGGCGATAAGTCGTGTCT

TACCGGGTTGGACTCAAGACGATAGTTACCGGATAAGGCGCAGCGGTCGGGCTGAACGGGGGGTTCGTGCACACAGCCCA

GCTTGGAGCGAACGACCTACACCGAACTGAGATACCTACAGCGTGAGCTATGAGAAAGCGCCACGCTTCCCGAAGGGAGA

AAGGCGGACAGGTATCCGGTAAGCGGCAGGGTCGGAACAGGAGAGCGCACGAGGGAGCTTCCAGGGGGAAACGCCTGGTA

TCTTTATAGTCCTGTCGGGTTTCGCCACCTCTGACTTGAGCGTCGATTTTTGTGATGCTCGTCAGGGGGGCGGAGCCTAT

GGAAAAACGCCAGCAACGCGGCCTTTTTACGGTTCCTGGCCTTTTGCTGGCCTTTTGCTCACATGTTCTTTCCTGCGTTA

TCCCCTGATTCTGTGGATAACCGTATTACCGCCTTTGAGTGAGCTGATACCGCTCGCCGCAGCCGAACGACCGAGCGCAG

CGAGTCAGTGAGCGAGGAAGCGGAAGAGCGCCTGATGCGGTATTTTCTCCTTACGCATCTGTGCGGTATTTCACACCGCA

TATATGGTGCACTCTCAGTACAATCTGCTCTGATGCCGCATAGTTAAGCCAGTATACACTCCGCTATCGCTACGTGACTG

GGTCATGGCTGCGCCCCGACACCCGCCAACACCCGCTGACGCGCCCTGACGGGCTTGTCTGCTCCCGGCATCCGCTTACA

GACAAGCTGTGACCGTCTCCGGGAGCTGCATGTGTCAGAGGTTTTCACCGTCATCACCGAAACGCGCGAGGCAGCTGCGG

TAAAGCTCATCAGCGTGGTCGTGAAGCGATTCACAGATGTCTGCCTGTTCATCCGCGTCCAGCTCGTTGAGTTTCTCCAG

AAGCGTTAATGTCTGGCTTCTGATAAAGCGGGCCATGTTAAGGGCGGTTTTTTCCTGTTTGGTCACTGATGCCTCCGTGT

AAGGGGGATTTCTGTTCATGGGGGTAATGATACCGATGAAACGAGAGAGGATGCTCACGATACGGGTTACTGATGATGAA

CATGCCCGGTTACTGGAACGTTGTGAGGGTAAACAACTGGCGGTATGGATGCGGCGGGACCAGAGAAAAATCACTCAGGG

TCAATGCCAGCGCTTCGTTAATACAGATGTAGGTGTTCCACAGGGTAGCCAGCAGCATCCTGCGATGCAGATCCGGAACA

TAATGGTGCAGGGCGCTGACTTCCGCGTTTCCAGACTTTACGAAACACGGAAACCGAAGACCATTCATGTTGTTGCTCAG

GTCGCAGACGTTTTGCAGCAGCAGTCGCTTCACGTTCGCTCGCGTATCGGTGATTCATTCTGCTAACCAGTAAGGCAACC

CCGCCAGCCTAGCCGGGTCCTCAACGACAGGAGCACGATCATGCGCACCCGTGGGGCCGCCATGCCGGCGATAATGGCCT

GCTTCTCGCCGAAACGTTTGGTGGCGGGACCAGTGACGAAGGCTTGAGCGAGGGCGTGCAAGATTCCGAATACCGCAAGC

GACAGGCCGATCATCGTCGCGCTCCAGCGAAAGCGGTCCTCGCCGAAAATGACCCAGAGCGCTGCCGGCACCTGTCCTAC

GAGTTGCATGATAAAGAAGACAGTCATAAGTGCGGCGACGATAGTCATGCCCCGCGCCCACCGGAAGGAGCTGACTGGGT

TGAAGGCTCTCAAGGGCATCGGTCGAGATCCCGGTGCCTAATGAGTGAGCTAACTTACATTAATTGCGTTGCGCTCACTG

CCCGCTTTCCAGTCGGGAAACCTGTCGTGCCAGCTGCATTAATGAATCGGCCAACGCGCGGGGAGAGGCGGTTTGCGTAT

TGGGCGCCAGGGTGGTTTTTCTTTTCACCAGTGAGACGGGCAACAGCTGATTGCCCTTCACCGCCTGGCCCTGAGAGAGT

TGCAGCAAGCGGTCCACGCTGGTTTGCCCCAGCAGGCGAAAATCCTGTTTGATGGTGGTTAACGGCGGGATATAACATGA

GCTGTCTTCGGTATCGTCGTATCCCACTACCGAGATATCCGCACCAACGCGCAGCCCGGACTCGGTAATGGCGCGCATTG

CGCCCAGCGCCATCTGATCGTTGGCAACCAGCATCGCAGTGGGAACGATGCCCTCATTCAGCATTTGCATGGTTTGTTGA

AAACCGGACATGGCACTCCAGTCGCCTTCCCGTTCCGCTATCGGCTGAATTTGATTGCGAGTGAGATATTTATGCCAGCC

AGCCAGACGCAGACGCGCCGAGACAGAACTTAATGGGCCCGCTAACAGCGCGATTTGCTGGTGACCCAATGCGACCAGAT

GCTCCACGCCCAGTCGCGTACCGTCTTCATGGGAGAAAATAATACTGTTGATGGGTGTCTGGTCAGAGACATCAAGAAAT

AACGCCGGAACATTAGTGCAGGCAGCTTCCACAGCAATGGCATCCTGGTCATCCAGCGGATAGTTAATGATCAGCCCACT

GACGCGTTGCGCGAGAAGATTGTGCACCGCCGCTTTACAGGCTTCGACGCCGCTTCGTTCTACCATCGACACCACCACGC

TGGCACCCAGTTGATCGGCGCGAGATTTAATCGCCGCGACAATTTGCGACGGCGCGTGCAGGGCCAGACTGGAGGTGGCA

ACGCCAATCAGCAACGACTGTTTGCCCGCCAGTTGTTGTGCCACGCGGTTGGGAATGTAATTCAGCTCCGCCATCGCCGC

TTCCACTTTTTCCCGCGTTTTCGCAGAAACGTGGCTGGCCTGGTTCACCACGCGGGAAACGGTCTGATAAGAGACACCGG

CATACTCTGCGACATCGTATAACGTTACTGGTTTCACATTCACCACCCTGAATTGACTCTCTTCCGGGCGCTATCATGCC

ATACCGCGAAAGGTTTTGCGCCATTCGATGGTGTCCGGGATCTCGACGCTCTCCCTTATGCGACTCCTGCATTAGGAAGC

AGCCCAGTAGTAGGTTGAGGCCGTTGAGCACCGCCGCCGCAAGGAATGGTGCATGCAAGGAGATGGCGCCCAACAGTCCC

CCGGCCACGGGGCCTGCCACCATACCCACGCCGAAACAAGCGCTCATGAGCCCGAAGTGGCGAGCCCGATCTTCCCCATC

GGTGATGTCGGCGATATAGGCGCCAGCAACCGCACCTGTGGCGCCGGTGATGCCGGCCACGATGCGTCCGGCGTAGAGGA

TCGAGATCTCGATCCCGCGAAATTAATACGACTCACTATAGGGGAATTGTGAGCGGATAACAATTCCCCTCTAGAAATAA

TTTTGATTTAACTTTAAGAAGGAGATATACCATGAAACATCACCATCACCATCACCCCATGAGCGATTACGACATCCCCA

CTACTGAGAATCTTTATTTTCAGGGCGCCATGGCGATGCTGCCTCCGAGCGATAGCAAAGATCCGCGTCCGTGGCAGATT

CTGAGCCAGGCACTGGGTTTTCCGAATTATGATCAAGAACTGTGGTGGCAGAATACCGCAGAAACCCTGAATCGTGTTCT

GGAACAGTGTGATTATAGCGTTCATCTGCAGTATAAATACCTGGCCTTCTACCACAAATATATCCTGCCGAGCCTGGGTC

CGTTTCGTCGTCCGGGTGTTGAACCGGAATATATCAGCGGTCTGAGTCATGGTGGTCATCCGCTGCAAATTAGCGTGAAA

ATTGATAAAAGCAAGACCATTTGTCGTCTGGGTCTGCAGGCAATTGGTCCGCTGGCAGGCACCGCACGTGATCCGCTGAA

TAGCTTTGGTGATCGTGAACTGCTGAAAAATCTGGCAACCCTGCTGCCGCATGTTGATCTGCGTCTGTTTGATCATTTTA

ATGCACAGGTTGGTCTGGATCGTGCACAGTGTGCAGTTGCAACCACCAAACTGATTAAAGAAAGCCATAATATTGTGTGC

ACCAGCCTGGATCTGAAAGATGGTGAAGTTATTCCGAAAGTGTACTTTAGCACCATTCCTAAAGGTCTGGTTACCGAAAC

ACCGCTGTTCGATCTGACCTTTGCAGCAATTGAGCAGATGGAAGTGTATCATAAAGATGCACCGCTGCGTACCGCACTGA

GCAGCCTGAAAGATTTTCTGCGTCCGCGTGTTCCGACCGATGCAAGCATTACCCCTCCGCTGACCGGTCTGATTGGTGTT

GATTGTATTGATCCGATGCTGAGCCGTCTGAAAGTTTATCTGGCCACCTTTCGTATGGATCTGAGCCTGATTCGTGATTA

TTGGACCTTAGGTGGTCTGCTGACCGATGCCGGTACAATGAAAGGCCTGGAAATGGTTGAAACCCTGGCAAAAACACTGA

AACTGGGTGATGAAGCATGTGAAACACTGGATGCAGAACGTCTGCCGTTTGGTATTAACTATGCAATGAAACCGGGTACA

GCAGAACTGGCACCGCCTCAGATCAGCTTTCCGCTGCTGGGTATTAATGATGGCTTTATTGCAGATGCCCTGGTGGAATT

TTTTCAGTATATGGGTTGGGAAGATCAGGCCAATCGTTATAAAGATGAACTGAAAGCCAAATTTCCGAACGTGGATATTA

GCCAGACCAAAAATGTTCATCGTTGGCTGGGTGTTGCATATAGCGAAACCAAAGGTCCGAGCATGAACATCTATTATGAT

GTTGTTGCAGGTAATGTGGCACGCGTTTAACTCGAGCACCACCACCACCACCACTGAGATCCGGCTGCTAACAAAGCCCG

AAAGGAAGCTGAGTTGGCTGCTGCCACCGCTGAGCAATAACTAGCATAACCCCTTGGGGCCTCTAAACGGGTCTTGAGGG

GTTTTTTGCTGAAAGGAGGAACTATATCCGGAT

>pET-M11-AtaPTE91Q/Y413S (Genbank: OQ725941)

TGGCGAATGGGACGCGCCCTGTAGCGGCGCATTAAGCGCGGCGGGTGTGGTGGTTACGCGCAGCGTGACCGCTACACTTG

CCAGCGCCCTAGCGCCCGCTCCTTTCGCTTTCTTCCCTTCCTTTCTCGCCACGTTCGCCGGCTTTCCCCGTCAAGCTCTA

AATCGGGGGCTCCCTTTAGGGTTCCGATTTAGTGCTTTACGGCACCTCGACCCCAAAAAACTTGATTAGGGTGATGGTTC

ACGTAGTGGGCCATCGCCCTGATAGACGGTTTTTCGCCCTTTGACGTTGGAGTCCACGTTCTTTAATAGTGGACTCTTGT

TCCAAACTGGAACAACACTCAACCCTATCTCGGTCTATTCTTTTGATTTATAAGGGATTTTGCCGATTTCGGCCTATTGG

TTAAAAAATGAGCTGATTTAACAAAAATTTAACGCGAATTTTAACAAAATATTAACGTTTACAATTTCAGGTGGCACTTT

TCGGGGAAATGTGCGCGGAACCCCTATTTGTTTATTTTTCTAAATACATTCAAATATGTATCCGCTCATGAATTAATTCT

TAGAAAAACTCATCGAGCATCAAATGAAACTGCAATTTATTCATATCAGGATTATCAATACCATATTTTTGAAAAAGCCG

TTTCTGTAATGAAGGAGAAAACTCACCGAGGCAGTTCCATAGGATGGCAAGATCCTGGTATCGGTCTGCGATTCCGACTC

GTCCAACATCAATACAACCTATTAATTTCCCCTCGTCAAAAATAAGGTTATCAAGTGAGAAATCACCATGAGTGACGACT

GAATCCGGTGAGAATGGCAAAAGTTTATGCATTTCTTTCCAGACTTGTTCAACAGGCCAGCCATTACGCTCGTCATCAAA

ATCACTCGCATCAACCAAACCGTTATTCATTCGTGATTGCGCCTGAGCGAGACGAAATACGCGATCGCTGTTAAAAGGAC

AATTACAAACAGGAATCGAATGCAACCGGCGCAGGAACACTGCCAGCGCATCAACAATATTTTCACCTGAATCAGGATAT

TCTTCTAATACCTGGAATGCTGTTTTCCCGGGGATCGCAGTGGTGAGTAACCATGCATCATCAGGAGTACGGATAAAATG

CTTGATGGTCGGAAGAGGCATAAATTCCGTCAGCCAGTTTAGTCTGACCATCTCATCTGTAACATCATTGGCAACGCTAC

CTTTGCCATGTTTCAGAAACAACTCTGGCGCATCGGGCTTCCCATACAATCGATAGATTGTCGCACCTGATTGCCCGACA

TTATCGCGAGCCCATTTATACCCATATAAATCAGCATCCATGTTGGAATTTAATCGCGGCCTAGAGCAAGACGTTTCCCG

TTGAATATGGCTCATAACACCCCTTGTATTACTGTTTATGTAAGCAGACAGTTTTATTGTTCATGACCAAAATCCCTTAA

CGTGAGTTTTCGTTCCACTGAGCGTCAGACCCCGTAGAAAAGATCAAAGGATCTTCTTGAGATCCTTTTTTTCTGCGCGT

AATCTGCTGCTTGCAAACAAAAAAACCACCGCTACCAGCGGTGGTTTGTTTGCCGGATCAAGAGCTACCAACTCTTTTTC

CGAAGGTAACTGGCTTCAGCAGAGCGCAGATACCAAATACTGTCCTTCTAGTGTAGCCGTAGTTAGGCCACCACTTCAAG

AACTCTGTAGCACCGCCTACATACCTCGCTCTGCTAATCCTGTTACCAGTGGCTGCTGCCAGTGGCGATAAGTCGTGTCT

TACCGGGTTGGACTCAAGACGATAGTTACCGGATAAGGCGCAGCGGTCGGGCTGAACGGGGGGTTCGTGCACACAGCCCA

GCTTGGAGCGAACGACCTACACCGAACTGAGATACCTACAGCGTGAGCTATGAGAAAGCGCCACGCTTCCCGAAGGGAGA

AAGGCGGACAGGTATCCGGTAAGCGGCAGGGTCGGAACAGGAGAGCGCACGAGGGAGCTTCCAGGGGGAAACGCCTGGTA

TCTTTATAGTCCTGTCGGGTTTCGCCACCTCTGACTTGAGCGTCGATTTTTGTGATGCTCGTCAGGGGGGCGGAGCCTAT

GGAAAAACGCCAGCAACGCGGCCTTTTTACGGTTCCTGGCCTTTTGCTGGCCTTTTGCTCACATGTTCTTTCCTGCGTTA

TCCCCTGATTCTGTGGATAACCGTATTACCGCCTTTGAGTGAGCTGATACCGCTCGCCGCAGCCGAACGACCGAGCGCAG

CGAGTCAGTGAGCGAGGAAGCGGAAGAGCGCCTGATGCGGTATTTTCTCCTTACGCATCTGTGCGGTATTTCACACCGCA

TATATGGTGCACTCTCAGTACAATCTGCTCTGATGCCGCATAGTTAAGCCAGTATACACTCCGCTATCGCTACGTGACTG

GGTCATGGCTGCGCCCCGACACCCGCCAACACCCGCTGACGCGCCCTGACGGGCTTGTCTGCTCCCGGCATCCGCTTACA

GACAAGCTGTGACCGTCTCCGGGAGCTGCATGTGTCAGAGGTTTTCACCGTCATCACCGAAACGCGCGAGGCAGCTGCGG

TAAAGCTCATCAGCGTGGTCGTGAAGCGATTCACAGATGTCTGCCTGTTCATCCGCGTCCAGCTCGTTGAGTTTCTCCAG

AAGCGTTAATGTCTGGCTTCTGATAAAGCGGGCCATGTTAAGGGCGGTTTTTTCCTGTTTGGTCACTGATGCCTCCGTGT

AAGGGGGATTTCTGTTCATGGGGGTAATGATACCGATGAAACGAGAGAGGATGCTCACGATACGGGTTACTGATGATGAA

CATGCCCGGTTACTGGAACGTTGTGAGGGTAAACAACTGGCGGTATGGATGCGGCGGGACCAGAGAAAAATCACTCAGGG

TCAATGCCAGCGCTTCGTTAATACAGATGTAGGTGTTCCACAGGGTAGCCAGCAGCATCCTGCGATGCAGATCCGGAACA

TAATGGTGCAGGGCGCTGACTTCCGCGTTTCCAGACTTTACGAAACACGGAAACCGAAGACCATTCATGTTGTTGCTCAG

GTCGCAGACGTTTTGCAGCAGCAGTCGCTTCACGTTCGCTCGCGTATCGGTGATTCATTCTGCTAACCAGTAAGGCAACC

CCGCCAGCCTAGCCGGGTCCTCAACGACAGGAGCACGATCATGCGCACCCGTGGGGCCGCCATGCCGGCGATAATGGCCT

GCTTCTCGCCGAAACGTTTGGTGGCGGGACCAGTGACGAAGGCTTGAGCGAGGGCGTGCAAGATTCCGAATACCGCAAGC

GACAGGCCGATCATCGTCGCGCTCCAGCGAAAGCGGTCCTCGCCGAAAATGACCCAGAGCGCTGCCGGCACCTGTCCTAC

GAGTTGCATGATAAAGAAGACAGTCATAAGTGCGGCGACGATAGTCATGCCCCGCGCCCACCGGAAGGAGCTGACTGGGT

TGAAGGCTCTCAAGGGCATCGGTCGAGATCCCGGTGCCTAATGAGTGAGCTAACTTACATTAATTGCGTTGCGCTCACTG

CCCGCTTTCCAGTCGGGAAACCTGTCGTGCCAGCTGCATTAATGAATCGGCCAACGCGCGGGGAGAGGCGGTTTGCGTAT

TGGGCGCCAGGGTGGTTTTTCTTTTCACCAGTGAGACGGGCAACAGCTGATTGCCCTTCACCGCCTGGCCCTGAGAGAGT

TGCAGCAAGCGGTCCACGCTGGTTTGCCCCAGCAGGCGAAAATCCTGTTTGATGGTGGTTAACGGCGGGATATAACATGA

GCTGTCTTCGGTATCGTCGTATCCCACTACCGAGATATCCGCACCAACGCGCAGCCCGGACTCGGTAATGGCGCGCATTG

CGCCCAGCGCCATCTGATCGTTGGCAACCAGCATCGCAGTGGGAACGATGCCCTCATTCAGCATTTGCATGGTTTGTTGA

AAACCGGACATGGCACTCCAGTCGCCTTCCCGTTCCGCTATCGGCTGAATTTGATTGCGAGTGAGATATTTATGCCAGCC

AGCCAGACGCAGACGCGCCGAGACAGAACTTAATGGGCCCGCTAACAGCGCGATTTGCTGGTGACCCAATGCGACCAGAT

GCTCCACGCCCAGTCGCGTACCGTCTTCATGGGAGAAAATAATACTGTTGATGGGTGTCTGGTCAGAGACATCAAGAAAT

AACGCCGGAACATTAGTGCAGGCAGCTTCCACAGCAATGGCATCCTGGTCATCCAGCGGATAGTTAATGATCAGCCCACT

GACGCGTTGCGCGAGAAGATTGTGCACCGCCGCTTTACAGGCTTCGACGCCGCTTCGTTCTACCATCGACACCACCACGC

TGGCACCCAGTTGATCGGCGCGAGATTTAATCGCCGCGACAATTTGCGACGGCGCGTGCAGGGCCAGACTGGAGGTGGCA

ACGCCAATCAGCAACGACTGTTTGCCCGCCAGTTGTTGTGCCACGCGGTTGGGAATGTAATTCAGCTCCGCCATCGCCGC

TTCCACTTTTTCCCGCGTTTTCGCAGAAACGTGGCTGGCCTGGTTCACCACGCGGGAAACGGTCTGATAAGAGACACCGG

CATACTCTGCGACATCGTATAACGTTACTGGTTTCACATTCACCACCCTGAATTGACTCTCTTCCGGGCGCTATCATGCC

ATACCGCGAAAGGTTTTGCGCCATTCGATGGTGTCCGGGATCTCGACGCTCTCCCTTATGCGACTCCTGCATTAGGAAGC

AGCCCAGTAGTAGGTTGAGGCCGTTGAGCACCGCCGCCGCAAGGAATGGTGCATGCAAGGAGATGGCGCCCAACAGTCCC

CCGGCCACGGGGCCTGCCACCATACCCACGCCGAAACAAGCGCTCATGAGCCCGAAGTGGCGAGCCCGATCTTCCCCATC

GGTGATGTCGGCGATATAGGCGCCAGCAACCGCACCTGTGGCGCCGGTGATGCCGGCCACGATGCGTCCGGCGTAGAGGA

TCGAGATCTCGATCCCGCGAAATTAATACGACTCACTATAGGGGAATTGTGAGCGGATAACAATTCCCCTCTAGAAATAA

TTTTGATTTAACTTTAAGAAGGAGATATACCATGAAACATCACCATCACCATCACCCCATGAGCGATTACGACATCCCCA

CTACTGAGAATCTTTATTTTCAGGGCGCCATGGCGATGCTGCCTCCGAGCGATAGCAAAGATCCGCGTCCGTGGCAGATT

CTGAGCCAGGCACTGGGTTTTCCGAATTATGATCAAGAACTGTGGTGGCAGAATACCGCAGAAACCCTGAATCGTGTTCT

GGAACAGTGTGATTATAGCGTTCATCTGCAGTATAAATACCTGGCCTTCTACCACAAATATATCCTGCCGAGCCTGGGTC

CGTTTCGTCGTCCGGGTGTTGAACCGGAATATATCAGCGGTCTGAGTCATGGTGGTCATCCGCTGCAAATTAGCGTGAAA

ATTGATAAAAGCAAGACCATTTGTCGTCTGGGTCTGCAGGCAATTGGTCCGCTGGCAGGCACCGCACGTGATCCGCTGAA

TAGCTTTGGTGATCGTGAACTGCTGAAAAATCTGGCAACCCTGCTGCCGCATGTTGATCTGCGTCTGTTTGATCATTTTA

ATGCACAGGTTGGTCTGGATCGTGCACAGTGTGCAGTTGCAACCACCAAACTGATTAAAGAAAGCCATAATATTGTGTGC

ACCAGCCTGGATCTGAAAGATGGTGAAGTTATTCCGAAAGTGTACTTTAGCACCATTCCTAAAGGTCTGGTTACCGAAAC

ACCGCTGTTCGATCTGACCTTTGCAGCAATTGAGCAGATGGAAGTGTATCATAAAGATGCACCGCTGCGTACCGCACTGA

GCAGCCTGAAAGATTTTCTGCGTCCGCGTGTTCCGACCGATGCAAGCATTACCCCTCCGCTGACCGGTCTGATTGGTGTT

GATTGTATTGATCCGATGCTGAGCCGTCTGAAAGTTTATCTGGCCACCTTTCGTATGGATCTGAGCCTGATTCGTGATTA

TTGGACCTTAGGTGGTCTGCTGACCGATGCCGGTACAATGAAAGGCCTGGAAATGGTTGAAACCCTGGCAAAAACACTGA

AACTGGGTGATGAAGCATGTGAAACACTGGATGCAGAACGTCTGCCGTTTGGTATTAACTATGCAATGAAACCGGGTACA

GCAGAACTGGCACCGCCTCAGATCTATTTTCCGCTGCTGGGTATTAATGATGGCTTTATTGCAGATGCCCTGGTGGAATT

TTTTCAGTATATGGGTTGGGAAGATCAGGCCAATCGTTATAAAGATGAACTGAAAGCCAAATTTCCGAACGTGGATATTA

GCCAGACCAAAAATGTTCATCGTTGGCTGGGTGTTGCATATAGCGAAACCAAAGGTCCGAGCATGAACATCAGCTATGAT

GTTGTTGCAGGTAATGTGGCACGCGTTTAACTCGAGCACCACCACCACCACCACTGAGATCCGGCTGCTAACAAAGCCCG

AAAGGAAGCTGAGTTGGCTGCTGCCACCGCTGAGCAATAACTAGCATAACCCCTTGGGGCCTCTAAACGGGTCTTGAGGG

GTTTTTTGCTGAAAGGAGGAACTATATCCGGAT

>pET-M11-AtaPTE91Q/Y344S/Y413S (Genbank: OQ725942)

TGGCGAATGGGACGCGCCCTGTAGCGGCGCATTAAGCGCGGCGGGTGTGGTGGTTACGCGCAGCGTGACCGCTACACTTG

CCAGCGCCCTAGCGCCCGCTCCTTTCGCTTTCTTCCCTTCCTTTCTCGCCACGTTCGCCGGCTTTCCCCGTCAAGCTCTA

AATCGGGGGCTCCCTTTAGGGTTCCGATTTAGTGCTTTACGGCACCTCGACCCCAAAAAACTTGATTAGGGTGATGGTTC

ACGTAGTGGGCCATCGCCCTGATAGACGGTTTTTCGCCCTTTGACGTTGGAGTCCACGTTCTTTAATAGTGGACTCTTGT

TCCAAACTGGAACAACACTCAACCCTATCTCGGTCTATTCTTTTGATTTATAAGGGATTTTGCCGATTTCGGCCTATTGG

TTAAAAAATGAGCTGATTTAACAAAAATTTAACGCGAATTTTAACAAAATATTAACGTTTACAATTTCAGGTGGCACTTT

TCGGGGAAATGTGCGCGGAACCCCTATTTGTTTATTTTTCTAAATACATTCAAATATGTATCCGCTCATGAATTAATTCT

TAGAAAAACTCATCGAGCATCAAATGAAACTGCAATTTATTCATATCAGGATTATCAATACCATATTTTTGAAAAAGCCG

TTTCTGTAATGAAGGAGAAAACTCACCGAGGCAGTTCCATAGGATGGCAAGATCCTGGTATCGGTCTGCGATTCCGACTC

GTCCAACATCAATACAACCTATTAATTTCCCCTCGTCAAAAATAAGGTTATCAAGTGAGAAATCACCATGAGTGACGACT

GAATCCGGTGAGAATGGCAAAAGTTTATGCATTTCTTTCCAGACTTGTTCAACAGGCCAGCCATTACGCTCGTCATCAAA

ATCACTCGCATCAACCAAACCGTTATTCATTCGTGATTGCGCCTGAGCGAGACGAAATACGCGATCGCTGTTAAAAGGAC

AATTACAAACAGGAATCGAATGCAACCGGCGCAGGAACACTGCCAGCGCATCAACAATATTTTCACCTGAATCAGGATAT

TCTTCTAATACCTGGAATGCTGTTTTCCCGGGGATCGCAGTGGTGAGTAACCATGCATCATCAGGAGTACGGATAAAATG

CTTGATGGTCGGAAGAGGCATAAATTCCGTCAGCCAGTTTAGTCTGACCATCTCATCTGTAACATCATTGGCAACGCTAC

CTTTGCCATGTTTCAGAAACAACTCTGGCGCATCGGGCTTCCCATACAATCGATAGATTGTCGCACCTGATTGCCCGACA

TTATCGCGAGCCCATTTATACCCATATAAATCAGCATCCATGTTGGAATTTAATCGCGGCCTAGAGCAAGACGTTTCCCG

TTGAATATGGCTCATAACACCCCTTGTATTACTGTTTATGTAAGCAGACAGTTTTATTGTTCATGACCAAAATCCCTTAA

CGTGAGTTTTCGTTCCACTGAGCGTCAGACCCCGTAGAAAAGATCAAAGGATCTTCTTGAGATCCTTTTTTTCTGCGCGT

AATCTGCTGCTTGCAAACAAAAAAACCACCGCTACCAGCGGTGGTTTGTTTGCCGGATCAAGAGCTACCAACTCTTTTTC

CGAAGGTAACTGGCTTCAGCAGAGCGCAGATACCAAATACTGTCCTTCTAGTGTAGCCGTAGTTAGGCCACCACTTCAAG

AACTCTGTAGCACCGCCTACATACCTCGCTCTGCTAATCCTGTTACCAGTGGCTGCTGCCAGTGGCGATAAGTCGTGTCT

TACCGGGTTGGACTCAAGACGATAGTTACCGGATAAGGCGCAGCGGTCGGGCTGAACGGGGGGTTCGTGCACACAGCCCA

GCTTGGAGCGAACGACCTACACCGAACTGAGATACCTACAGCGTGAGCTATGAGAAAGCGCCACGCTTCCCGAAGGGAGA

AAGGCGGACAGGTATCCGGTAAGCGGCAGGGTCGGAACAGGAGAGCGCACGAGGGAGCTTCCAGGGGGAAACGCCTGGTA

TCTTTATAGTCCTGTCGGGTTTCGCCACCTCTGACTTGAGCGTCGATTTTTGTGATGCTCGTCAGGGGGGCGGAGCCTAT

GGAAAAACGCCAGCAACGCGGCCTTTTTACGGTTCCTGGCCTTTTGCTGGCCTTTTGCTCACATGTTCTTTCCTGCGTTA

TCCCCTGATTCTGTGGATAACCGTATTACCGCCTTTGAGTGAGCTGATACCGCTCGCCGCAGCCGAACGACCGAGCGCAG

CGAGTCAGTGAGCGAGGAAGCGGAAGAGCGCCTGATGCGGTATTTTCTCCTTACGCATCTGTGCGGTATTTCACACCGCA

TATATGGTGCACTCTCAGTACAATCTGCTCTGATGCCGCATAGTTAAGCCAGTATACACTCCGCTATCGCTACGTGACTG

GGTCATGGCTGCGCCCCGACACCCGCCAACACCCGCTGACGCGCCCTGACGGGCTTGTCTGCTCCCGGCATCCGCTTACA

GACAAGCTGTGACCGTCTCCGGGAGCTGCATGTGTCAGAGGTTTTCACCGTCATCACCGAAACGCGCGAGGCAGCTGCGG

TAAAGCTCATCAGCGTGGTCGTGAAGCGATTCACAGATGTCTGCCTGTTCATCCGCGTCCAGCTCGTTGAGTTTCTCCAG

AAGCGTTAATGTCTGGCTTCTGATAAAGCGGGCCATGTTAAGGGCGGTTTTTTCCTGTTTGGTCACTGATGCCTCCGTGT

AAGGGGGATTTCTGTTCATGGGGGTAATGATACCGATGAAACGAGAGAGGATGCTCACGATACGGGTTACTGATGATGAA

CATGCCCGGTTACTGGAACGTTGTGAGGGTAAACAACTGGCGGTATGGATGCGGCGGGACCAGAGAAAAATCACTCAGGG

TCAATGCCAGCGCTTCGTTAATACAGATGTAGGTGTTCCACAGGGTAGCCAGCAGCATCCTGCGATGCAGATCCGGAACA

TAATGGTGCAGGGCGCTGACTTCCGCGTTTCCAGACTTTACGAAACACGGAAACCGAAGACCATTCATGTTGTTGCTCAG

GTCGCAGACGTTTTGCAGCAGCAGTCGCTTCACGTTCGCTCGCGTATCGGTGATTCATTCTGCTAACCAGTAAGGCAACC

CCGCCAGCCTAGCCGGGTCCTCAACGACAGGAGCACGATCATGCGCACCCGTGGGGCCGCCATGCCGGCGATAATGGCCT

GCTTCTCGCCGAAACGTTTGGTGGCGGGACCAGTGACGAAGGCTTGAGCGAGGGCGTGCAAGATTCCGAATACCGCAAGC

GACAGGCCGATCATCGTCGCGCTCCAGCGAAAGCGGTCCTCGCCGAAAATGACCCAGAGCGCTGCCGGCACCTGTCCTAC

GAGTTGCATGATAAAGAAGACAGTCATAAGTGCGGCGACGATAGTCATGCCCCGCGCCCACCGGAAGGAGCTGACTGGGT

TGAAGGCTCTCAAGGGCATCGGTCGAGATCCCGGTGCCTAATGAGTGAGCTAACTTACATTAATTGCGTTGCGCTCACTG

CCCGCTTTCCAGTCGGGAAACCTGTCGTGCCAGCTGCATTAATGAATCGGCCAACGCGCGGGGAGAGGCGGTTTGCGTAT

TGGGCGCCAGGGTGGTTTTTCTTTTCACCAGTGAGACGGGCAACAGCTGATTGCCCTTCACCGCCTGGCCCTGAGAGAGT

TGCAGCAAGCGGTCCACGCTGGTTTGCCCCAGCAGGCGAAAATCCTGTTTGATGGTGGTTAACGGCGGGATATAACATGA

GCTGTCTTCGGTATCGTCGTATCCCACTACCGAGATATCCGCACCAACGCGCAGCCCGGACTCGGTAATGGCGCGCATTG

CGCCCAGCGCCATCTGATCGTTGGCAACCAGCATCGCAGTGGGAACGATGCCCTCATTCAGCATTTGCATGGTTTGTTGA

AAACCGGACATGGCACTCCAGTCGCCTTCCCGTTCCGCTATCGGCTGAATTTGATTGCGAGTGAGATATTTATGCCAGCC

AGCCAGACGCAGACGCGCCGAGACAGAACTTAATGGGCCCGCTAACAGCGCGATTTGCTGGTGACCCAATGCGACCAGAT

GCTCCACGCCCAGTCGCGTACCGTCTTCATGGGAGAAAATAATACTGTTGATGGGTGTCTGGTCAGAGACATCAAGAAAT

AACGCCGGAACATTAGTGCAGGCAGCTTCCACAGCAATGGCATCCTGGTCATCCAGCGGATAGTTAATGATCAGCCCACT

GACGCGTTGCGCGAGAAGATTGTGCACCGCCGCTTTACAGGCTTCGACGCCGCTTCGTTCTACCATCGACACCACCACGC

TGGCACCCAGTTGATCGGCGCGAGATTTAATCGCCGCGACAATTTGCGACGGCGCGTGCAGGGCCAGACTGGAGGTGGCA

ACGCCAATCAGCAACGACTGTTTGCCCGCCAGTTGTTGTGCCACGCGGTTGGGAATGTAATTCAGCTCCGCCATCGCCGC

TTCCACTTTTTCCCGCGTTTTCGCAGAAACGTGGCTGGCCTGGTTCACCACGCGGGAAACGGTCTGATAAGAGACACCGG

CATACTCTGCGACATCGTATAACGTTACTGGTTTCACATTCACCACCCTGAATTGACTCTCTTCCGGGCGCTATCATGCC

ATACCGCGAAAGGTTTTGCGCCATTCGATGGTGTCCGGGATCTCGACGCTCTCCCTTATGCGACTCCTGCATTAGGAAGC

AGCCCAGTAGTAGGTTGAGGCCGTTGAGCACCGCCGCCGCAAGGAATGGTGCATGCAAGGAGATGGCGCCCAACAGTCCC

CCGGCCACGGGGCCTGCCACCATACCCACGCCGAAACAAGCGCTCATGAGCCCGAAGTGGCGAGCCCGATCTTCCCCATC

GGTGATGTCGGCGATATAGGCGCCAGCAACCGCACCTGTGGCGCCGGTGATGCCGGCCACGATGCGTCCGGCGTAGAGGA

TCGAGATCTCGATCCCGCGAAATTAATACGACTCACTATAGGGGAATTGTGAGCGGATAACAATTCCCCTCTAGAAATAA

TTTTGATTTAACTTTAAGAAGGAGATATACCATGAAACATCACCATCACCATCACCCCATGAGCGATTACGACATCCCCA

CTACTGAGAATCTTTATTTTCAGGGCGCCATGGCGATGCTGCCTCCGAGCGATAGCAAAGATCCGCGTCCGTGGCAGATT

CTGAGCCAGGCACTGGGTTTTCCGAATTATGATCAAGAACTGTGGTGGCAGAATACCGCAGAAACCCTGAATCGTGTTCT

GGAACAGTGTGATTATAGCGTTCATCTGCAGTATAAATACCTGGCCTTCTACCACAAATATATCCTGCCGAGCCTGGGTC

CGTTTCGTCGTCCGGGTGTTGAACCGGAATATATCAGCGGTCTGAGTCATGGTGGTCATCCGCTGCAAATTAGCGTGAAA

ATTGATAAAAGCAAGACCATTTGTCGTCTGGGTCTGCAGGCAATTGGTCCGCTGGCAGGCACCGCACGTGATCCGCTGAA

TAGCTTTGGTGATCGTGAACTGCTGAAAAATCTGGCAACCCTGCTGCCGCATGTTGATCTGCGTCTGTTTGATCATTTTA

ATGCACAGGTTGGTCTGGATCGTGCACAGTGTGCAGTTGCAACCACCAAACTGATTAAAGAAAGCCATAATATTGTGTGC

ACCAGCCTGGATCTGAAAGATGGTGAAGTTATTCCGAAAGTGTACTTTAGCACCATTCCTAAAGGTCTGGTTACCGAAAC

ACCGCTGTTCGATCTGACCTTTGCAGCAATTGAGCAGATGGAAGTGTATCATAAAGATGCACCGCTGCGTACCGCACTGA

GCAGCCTGAAAGATTTTCTGCGTCCGCGTGTTCCGACCGATGCAAGCATTACCCCTCCGCTGACCGGTCTGATTGGTGTT

GATTGTATTGATCCGATGCTGAGCCGTCTGAAAGTTTATCTGGCCACCTTTCGTATGGATCTGAGCCTGATTCGTGATTA

TTGGACCTTAGGTGGTCTGCTGACCGATGCCGGTACAATGAAAGGCCTGGAAATGGTTGAAACCCTGGCAAAAACACTGA

AACTGGGTGATGAAGCATGTGAAACACTGGATGCAGAACGTCTGCCGTTTGGTATTAACTATGCAATGAAACCGGGTACA

GCAGAACTGGCACCGCCTCAGATCAGCTTTCCGCTGCTGGGTATTAATGATGGCTTTATTGCAGATGCCCTGGTGGAATT

TTTTCAGTATATGGGTTGGGAAGATCAGGCCAATCGTTATAAAGATGAACTGAAAGCCAAATTTCCGAACGTGGATATTA

GCCAGACCAAAAATGTTCATCGTTGGCTGGGTGTTGCATATAGCGAAACCAAAGGTCCGAGCATGAACATCAGCTATGAT

GTTGTTGCAGGTAATGTGGCACGCGTTTAACTCGAGCACCACCACCACCACCACTGAGATCCGGCTGCTAACAAAGCCCG

AAAGGAAGCTGAGTTGGCTGCTGCCACCGCTGAGCAATAACTAGCATAACCCCTTGGGGCCTCTAAACGGGTCTTGAGGG

GTTTTTTGCTGAAAGGAGGAACTATATCCGGAT

>pBbB2c-CBGA (Genbank: OQ725943)

GACGTCTTAAGACCCACTTTCACATTTAAGTTGTTTTTCTAATCCGCATATGATCAATTCAAGGCCGAATAAGAAGGCTG

GCTCTGCACCTTGGTGATCAAATAATTCGATAGCTTGTCGTAATAATGGCGGCATACTATCAGTAGTAGGTGTTTCCCTT

TCTTCTTTAGCGACTTGATGCTCTTGATCTTCCAATACGCAACCTAAAGTAAAATGCCCCACAGCGCTGAGTGCATATAA

TGCATTCTCTAGTGAAAAACCTTGTTGGCATAAAAAGGCTAATTGATTTTCGAGAGTTTCATACTGTTTTTCTGTAGGCC

GTGTACCTAAATGTACTTTTGCTCCATCGCGATGACTTAGTAAAGCACATCTAAAACTTTTAGCGTTATTACGTAAAAAA

TCTTGCCAGCTTTCCCCTTCTAAAGGGCAAAAGTGAGTATGGTGCCTATCTAACATCTCAATGGCTAAGGCGTCGAGCAA

AGCCCGCTTATTTTTTACATGCCAATACAATGTAGGCTGCTCTACACCTAGCTTCTGGGCGAGTTTACGGGTTGTTAAAC

CTTCGATTCCGACCTCATTAAGCAGCTCTAATGCGCTGTTAATCACTTTACTTTTATCTAATCTAGACATCATTAATTCC

TAATTTTTGTTGACACTCTATCGTTGATAGAGTTATTTTACCACTCCCTATCAGTGATAGAGAAAAGAATTCAAAAGATC

TTTTAAGAAGCCTTAGGTCAATCCACAAAAGCGGCGCATAAGGAAAACTCCCTAGATTCGAGGACCAACAATGAACCACC

TAAGAGCAGAAGGCCCAGCCTCCGTGCTGGCCATTGGTACCGCCAATCCGGAAAACATTTTATTGCAGGACGAATTTCCT

GATTATTATTTTCGTGTTACCAAATCGGAACACATGACCCAGCTGAAAGAAAAATTTCGCAAAATTTGTGACAAAAGTAT

GATACGGAAACGAAATTGTTTTCTGAATGAGGAACACTTGAAGCAAAACCCTCGCTTAGTCGAACATGAGATGCAGACAC

TGGATGCCCGCCAGGACATGCTCGTGGTTGAAGTGCCGAAATTAGGTAAAGACGCGTGCGCGAAGGCGATTAAAGAGTGG

GGTCAGCCGAAATCCAAGATTACACACTTAATTTTTACCTCAGCGTCGACTACCGATATGCCTGGTGCCGACTATCATTG

TGCCAAGTTGTTGGGCTTAAGCCCCTCGGTTAAGCGTGTGATGATGTACCAACTGGGTTGTTATGGCGGTGGCACGGTGC

TGAGAATTGCGAAAGATATTGCCGAGAATAATAAAGGCGCGCGTGTTCTGGCCGTTTGCTGTGATATCATGGCATGCCTG

TTTCGTGGTCCTTCTGAGAGCGACTTAGAACTGCTGGTCGGCCAGGCTATATTCGGGGATGGTGCGGCGGCAGTCATTGT

TGGCGCGGAACCCGATGAAAGCGTTGGCGAGCGCCCAATCTTCGAATTAGTAAGCACAGGTCAGACGATATTACCAAATA

GCGAAGGCACTATCGGGGGCCACATCCGCGAAGCTGGACTGATTTTTGACCTGCATAAAGATGTCCCGATGCTGATTAGT

AATAATATCGAGAAATGTCTGATTGAGGCATTTACCCCAATTGGAATTAGCGACTGGAACAGCATTTTCTGGATAACGCA

TCCGGGCGGCAAAGCTATCCTGGATAAAGTTGAGGAAAAGCTCCACCTGAAATCTGATAAGTTTGTAGATTCCCGCCACG

TGTTGAGCGAACATGGTAATATGAGCTCCTCAACAGTCCTATTCGTTATGGATGAACTGCGCAAACGCTCGCTCGAAGAA

GGTAAAAGTACCACCGGCGATGGCTTTGAATGGGGCGTGTTATTCGGTTTTGGCCCAGGCTTAACCGTTGAGCGTGTGGT

GGTACGATCGGTTCCTATAAAATATTGACTCTAGAAATAATTTTGTTTAACTTTAAGAAGGAGATATACATATGTCCCCT

ATACTAGGTTATTGGAAAATTAAGGGCCTTGTGCAACCCACTCGACTTCTTTTGGAATATCTTGAAGAAAAATATGAAGA

GCATTTGTATGAGCGCGATGAAGGTGATAAATGGCGAAACAAAAAGTTTGAATTGGGTTTGGAGTTTCCCAATCTTCCTT

ATTATATTGATGGTGATGTTAAATTAACACAGTCTATGGCCATCATACGTTATATAGCTGACAAGCACAACATGTTGGGT

GGTTGTCCAAAAGAGCGTGCAGAGATTTCAATGCTTGAAGGAGCGGTTTTGGATATTAGATACGGTGTTTCGAGAATTGC

ATATAGTAAAGACTTTGAAACTCTCAAAGTTGATTTTCTTAGCAAGCTACCTGAAATGCTGAAAATGTTCGAAGATCGTT

TATGTCATAAAACATATTTAAATGGTGATCATGTAACCCATCCTGACTTCATGTTGTATGACGCTCTTGATGTTGTTTTA

TACATGGACCCAATGTGCCTGGATGCGTTCCCAAAATTAGTTTGTTTTAAAAAACGTATTGAAGCTATCCCACAAATTGA

TAAGTACTTGAAATCCAGCAAGTATATAGCATGGCCTTTGCAGGGCTGGCAAGCCACGTTTGGTGGTGGCGACCATCCTC

CAAAATCGGATGGTTCAACTAGTGGTTCTGGTCATCACCATCACCATCACTCCATGAGCGATTACGACATCCCCACTACT

GAGAATCTTTATTTTCAGGGCGCCATGGCAGTAAAACATTTAATTGTGCTCAAATTTAAAGACGAAATTACTGAAGCACA

AAAAGAAGAGTTTTTCAAAACATACGTCAATCTGGTGAACATCATCCCGGCCATGAAAGACGTCTATTGGGGCAAAGACG

TAACGCAGAAAAACAAGGAAGAAGGTTATACCCACATCGTGGAGGTCACTTTCGAATCAGTCGAAACGATCCAGGATTAC

ATTATTCATCCGGCACATGTAGGGTTCGGCGATGTATATCGTAGCTTCTGGGAAAAACTCCTGATTTTTGACTATACCCC

GCGTAAATAAAGAAAAGAATTCACGGAAGGAGCATGGCGATGCTGCCTCCGAGCGATAGCAAAGATCCGCGTCCGTGGCA

GATTCTGAGCCAGGCACTGGGTTTTCCGAATTATGATCAAGAACTGTGGTGGCAGAATACCGCAGAAACCCTGAATCGTG

TTCTGGAACAGTGTGATTATAGCGTTCATCTGCAGTATAAATACCTGGCCTTCTACCACAAATATATCCTGCCGAGCCTG

GGTCCGTTTCGTCGTCCGGGTGTTGAACCGGAATATATCAGCGGTCTGAGTCATGGTGGTCATCCGCTGGAAATTAGCGT

GAAAATTGATAAAAGCAAGACCATTTGTCGTCTGGGTCTGCAGGCAATTGGTCCGCTGGCAGGCACCGCACGTGATCCGC

TGAATAGCTTTGGTGATCGTGAACTGCTGAAAAATCTGGCAACCCTGCTGCCGCATGTTGATCTGCGTCTGTTTGATCAT

TTTAATGCACAGGTTGGTCTGGATCGTGCACAGTGTGCAGTTGCAACCACCAAACTGATTAAAGAAAGCCATAATATTGT

GTGCACCAGCCTGGATCTGAAAGATGGTGAAGTTATTCCGAAAGTGTACTTTAGCACCATTCCTAAAGGTCTGGTTACCG

AAACACCGCTGTTCGATCTGACCTTTGCAGCAATTGAGCAGATGGAAGTGTATCATAAAGATGCACCGCTGCGTACCGCA

CTGAGCAGCCTGAAAGATTTTCTGCGTCCGCGTGTTCCGACCGATGCAAGCATTACCCCTCCGCTGACCGGTCTGATTGG

TGTTGATTGTATTGATCCGATGCTGAGCCGTCTGAAAGTTTATCTGGCCACCTTTCGTATGGATCTGAGCCTGATTCGTG

ATTATTGGACCTTAGGTGGTCTGCTGACCGATGCCGGTACAATGAAAGGCCTGGAAATGGTTGAAACCCTGGCAAAAACA

CTGAAACTGGGTGATGAAGCATGTGAAACACTGGATGCAGAACGTCTGCCGTTTGGTATTAACTATGCAATGAAACCGGG

TACAGCAGAACTGGCACCGCCTCAGATCTATTTTCCGCTGCTGGGTATTAATGATGGCTTTATTGCAGATGCCCTGGTGG

AATTTTTTCAGTATATGGGTTGGGAAGATCAGGCCAATCGTTATAAAGATGAACTGAAAGCCAAATTTCCGAACGTGGAT

ATTAGCCAGACCAAAAATGTTCATCGTTGGCTGGGTGTTGCATATAGCGAAACCAAAGGTCCGAGCATGAACATCTATTA

TGATGTTGTTGCAGGTAATGTGGCACGCGTTTAAACGATCTTAAGTAGGCGCGGAAAATAATGGAGTTCGACTTCAACAA

ATACATGGACAGCAAAGCCATGACCGTTAATGAAGCACTGAATAAAGCAATTCCGCTGCGTTATCCGCAGAAAATCTATG

AAAGCATGCGTTATAGCCTGCTGGCAGGCGGTAAACGTGTTCGTCCGGTTCTGTGTATTGCAGCATGTGAACTGGTTGGT

GGCACCGAAGAACTGGCAATTCCGACCGCATGTGCAATTGAAATGATTCATACCATGAGCCTGATGCATGATGATCTGCC

GTGTATTGATAATGATGACCTGCGTCGTGGTAAACCGACCAATCATAAAATCTTTGGTGAAGATACCGCAGTGACCGCAG

GTAATGCACTGCATAGTTATGCATTTGAACATATTGCAGTGAGCACCAGCAAAACCGTTGGTGCAGATCGTATTCTGCGT

ATGGTTAGCGAACTGGGTCGTGCAACCGGTAGCGAAGGTGTTATGGGTGGTCAGATGGTTGATATTGCAAGTGAAGGTGA

TCCGAGCATTGATCTGCAGACCCTGGAATGGATTCATATTCATAAAACCGCAATGCTGCTGGAATGTAGCGTTGTTTGTG

GTGCAATTATTGGTGGTGCAAGCGAAATTGTTATTGAACGTGCCCGTCGTTATGCACGTTGTGTTGGTCTGCTGTTTCAG

GTTGTTGATGATATTCTGGATGTGACCAAAAGCAGTGATGAACTGGGCAAAACCGCAGGCAAAGATCTGATTAGCGATAA

AGCAACCTATCCGAAACTGATGGGTCTGGAAAAAGCCAAAGAATTTTCAGATGAACTGCTGAATCGTGCCAAAGGTGAAC

TGAGCTGTTTTGATCCGGTTAAAGCAGCACCGCTGCTGGGTCTGGCAGATTATGTTGCATTTCGTCAGAATTAAGGGATC

CAAACTCGAGTAAGGATCTCCAGGCATCAAATAAAACGAAAGGCTCAGTCGAAAGACTGGGCCTTTCGTTTTATCTGTTG

TTTGTCGGTGAACGCTCTCTACTAGAGTCACACTGGCTCACCTTCGGGTGGGCCTTTCTGCGTTTATACCTAGGCTACAG

CCGATAGTCTGGAACAGCGCACTTACGGGTTGCTGCGCAACCCAAGTGCTACCGGCGCGGCAGCGTGACCCGTGTCGGCG

GCTCCAACGGCTCGCCATCGTCCAGAAAACACGGCTCATCGGGCATCGGCAGGCGCTGCTGCCCGCGCCGTTCCCATTCC

TCCGTTTCGGTCAAGGCTGGCAGGTCTGGTTCCATGCCCGGAATGCCGGGCTGGCTGGGCGGCTCCTCGCCGGGGCCGGT

CGGTAGTTGCTGCTCGCCCGGATACAGGGTCGGGATGCGGCGCAGGTCGCCATGCCCCAACAGCGATTCGTCCTGGTCGT

CGTGATCAACCACCACGGCGGCACTGAACACCGACAGGCGCAACTGGTCGCGGGGCTGGCCCCACGCCACGCGGTCATTG

ACCACGTAGGCCAACACGGTGCCGGGGCCGTTGAGCTTCACGACGGAGATCCAGCGCTCGGCCACCAAGTCCTTGACTGC

GTATTGGACCGTCCGCAAAGAACGTCCGATGAGCTTGGAAAGTGTCTTCTGGCTGACCACCACGGCGTTCTGGTGGCCCA

TCTGCGCCACGAGGTGATGCAGCAGCATTGCCGCCGTGGGTTTCCTCGCAATAAGCCCGGCCCACGCCTCATGCGCTTTG

CGTTCCGTTTGCACCCAGTGACCGGGCTTGTTCTTGGCTTGAATGCCGATTTCTCTGGACTGCGTGGCCATGCTTATCTC

CATGCGGTAGGGGTGCCGCACGGTTGCGGCACCATGCGCAATCAGCTGCAACTTTTCGGCAGCGCGACAACAATTATGCG

TTGCGTAAAAGTGGCAGTCAATTACAGATTTTCTTTAACCTACGCAATGAGCTATTGCGGGGGGTGCCGCAATGAGCTGT

TGCGTACCCCCCTTTTTTAAGTTGTTGATTTTTAAGTCTTTCGCATTTCGCCCTATATCTAGTTCTTTGGTGCCCAAAGA

AGGGCACCCCTGCGGGGTTCCCCCACGCCTTCGGCGCGGCTCCCCCTCCGGCAAAAAGTGGCCCCTCCGGGGCTTGTTGA

TCGACTGCGCGGCCTTCGGCCTTGCCCAAGGTGGCGCTGCCCCCTTGGAACCCCCGCACTCGCCGCCGTGAGGCTCGGGG

GGCAGGCGGGCGGGCTTCGCCCTTCGACTGCCCCCACTCGCATAGGCTTGGGTCGTTCCAGGCGCGTCAAGGCCAAGCCG

CTGCGCGGTCGCTGCGCGAGCCTTGACCCGCCTTCCACTTGGTGTCCAACCGGCAAGCGAAGCGCGCAGGCCGCAGGCCG

GAGGCACTAGTGCTTGGATTCTCACCAATAAAAAACGCCCGGCGGCAACCGAGCGTTCTGAACAAATCCAGATGGAGTTC

TGAGGTCATTACTGGATCTATCAACAGGAGTCCAAGCGAGCTCGATATCAAATTACGCCCCGCCCTGCCACTCATCGCAG

TACTGTTGTAATTCATTAAGCATTCTGCCGACATGGAAGCCATCACAAACGGCATGATGAACCTGAATCGCCAGCGGCAT

CAGCACCTTGTCGCCTTGCGTATAATATTTGCCCATGGTGAAAACGGGGGCGAAGAAGTTGTCCATATTGGCCACGTTTA

AATCAAAACTGGTGAAACTCACCCAGGGATTGGCTGAGACGAAAAACATATTCTCAATAAACCCTTTAGGGAAATAGGCC

AGGTTTTCACCGTAACACGCCACATCTTGCGAATATATGTGTAGAAACTGCCGGAAATCGTCGTGGTATTCACTCCAGAG

CGATGAAAACGTTTCAGTTTGCTCATGGAAAACGGTGTAACAAGGGTGAACACTATCCCATATCACCAGCTCACCGTCTT

TCATTGCCATACGAAATTCCGGATGAGCATTCATCAGGCGGGCAAGAATGTGAATAAAGGCCGGATAAAACTTGTGCTTA

TTTTTCTTTACGGTCTTTAAAAAGGCCGTAATATCCAGCTGAACGGTCTGGTTATAGGTACATTGAGCAACTGACTGAAA

TGCCTCAAAATGTTCTTTACGATGCCATTGGGATATATCAACGGTGGTATATCCAGTGATTTTTTTCTCCATTTTAGCTT

CCTTAGCTCCTGAAAATCTCGATAACTCAAAAAATACGCCCGGTAGTGATCTTATTTCATTATGGTGAAAGTTGGAACCT

CTTACGTGCCGATCAACGTCTCATTTTCGCCAGATATC

>pBbB2c-CBGA_J23100 (Genbank: OQ725944)

GACGTCTTAAGACCCACTTTCACATTTAAGTTGTTTTTCTAATCCGCATATGATCAATTCAAGGCCGAATAAGAAGGCTG

GCTCTGCACCTTGGTGATCAAATAATTCGATAGCTTGTCGTAATAATGGCGGCATACTATCAGTAGTAGGTGTTTCCCTT

TCTTCTTTAGCGACTTGATGCTCTTGATCTTCCAATACGCAACCTAAAGTAAAATGCCCCACAGCGCTGAGTGCATATAA

TGCATTCTCTAGTGAAAAACCTTGTTGGCATAAAAAGGCTAATTGATTTTCGAGAGTTTCATACTGTTTTTCTGTAGGCC

GTGTACCTAAATGTACTTTTGCTCCATCGCGATGACTTAGTAAAGCACATCTAAAACTTTTAGCGTTATTACGTAAAAAA

TCTTGCCAGCTTTCCCCTTCTAAAGGGCAAAAGTGAGTATGGTGCCTATCTAACATCTCAATGGCTAAGGCGTCGAGCAA

AGCCCGCTTATTTTTTACATGCCAATACAATGTAGGCTGCTCTACACCTAGCTTCTGGGCGAGTTTACGGGTTGTTAAAC

CTTCGATTCCGACCTCATTAAGCAGCTCTAATGCGCTGTTAATCACTTTACTTTTATCTAATCTAGACATCATTAATTCC

TAATTTTTGTTGACACTCTATCGTTGATAGAGTTATTTTACCACTCCCTATCAGTGATAGAGAAAAGAATTCAAAAGATC

TTTTAAGAAGCCTTAGGTCAATCCACAAAAGCGGCGCATAAGGAAAACTCCCTAGATTCGAGGACCAACAATGAACCACC

TAAGAGCAGAAGGCCCAGCCTCCGTGCTGGCCATTGGTACCGCCAATCCGGAAAACATTTTATTGCAGGACGAATTTCCT

GATTATTATTTTCGTGTTACCAAATCGGAACACATGACCCAGCTGAAAGAAAAATTTCGCAAAATTTGTGACAAAAGTAT

GATACGGAAACGAAATTGTTTTCTGAATGAGGAACACTTGAAGCAAAACCCTCGCTTAGTCGAACATGAGATGCAGACAC

TGGATGCCCGCCAGGACATGCTCGTGGTTGAAGTGCCGAAATTAGGTAAAGACGCGTGCGCGAAGGCGATTAAAGAGTGG

GGTCAGCCGAAATCCAAGATTACACACTTAATTTTTACCTCAGCGTCGACTACCGATATGCCTGGTGCCGACTATCATTG

TGCCAAGTTGTTGGGCTTAAGCCCCTCGGTTAAGCGTGTGATGATGTACCAACTGGGTTGTTATGGCGGTGGCACGGTGC

TGAGAATTGCGAAAGATATTGCCGAGAATAATAAAGGCGCGCGTGTTCTGGCCGTTTGCTGTGATATCATGGCATGCCTG

TTTCGTGGTCCTTCTGAGAGCGACTTAGAACTGCTGGTCGGCCAGGCTATATTCGGGGATGGTGCGGCGGCAGTCATTGT

TGGCGCGGAACCCGATGAAAGCGTTGGCGAGCGCCCAATCTTCGAATTAGTAAGCACAGGTCAGACGATATTACCAAATA

GCGAAGGCACTATCGGGGGCCACATCCGCGAAGCTGGACTGATTTTTGACCTGCATAAAGATGTCCCGATGCTGATTAGT

AATAATATCGAGAAATGTCTGATTGAGGCATTTACCCCAATTGGAATTAGCGACTGGAACAGCATTTTCTGGATAACGCA

TCCGGGCGGCAAAGCTATCCTGGATAAAGTTGAGGAAAAGCTCCACCTGAAATCTGATAAGTTTGTAGATTCCCGCCACG

TGTTGAGCGAACATGGTAATATGAGCTCCTCAACAGTCCTATTCGTTATGGATGAACTGCGCAAACGCTCGCTCGAAGAA

GGTAAAAGTACCACCGGCGATGGCTTTGAATGGGGCGTGTTATTCGGTTTTGGCCCAGGCTTAACCGTTGAGCGTGTGGT

GGTACGATCGGTTCCTATAAAATATTGACTCTAGAAATAATTTTGTTTAACTTTAAGAAGGAGATATACATATGTCCCCT

ATACTAGGTTATTGGAAAATTAAGGGCCTTGTGCAACCCACTCGACTTCTTTTGGAATATCTTGAAGAAAAATATGAAGA

GCATTTGTATGAGCGCGATGAAGGTGATAAATGGCGAAACAAAAAGTTTGAATTGGGTTTGGAGTTTCCCAATCTTCCTT

ATTATATTGATGGTGATGTTAAATTAACACAGTCTATGGCCATCATACGTTATATAGCTGACAAGCACAACATGTTGGGT

GGTTGTCCAAAAGAGCGTGCAGAGATTTCAATGCTTGAAGGAGCGGTTTTGGATATTAGATACGGTGTTTCGAGAATTGC

ATATAGTAAAGACTTTGAAACTCTCAAAGTTGATTTTCTTAGCAAGCTACCTGAAATGCTGAAAATGTTCGAAGATCGTT

TATGTCATAAAACATATTTAAATGGTGATCATGTAACCCATCCTGACTTCATGTTGTATGACGCTCTTGATGTTGTTTTA

TACATGGACCCAATGTGCCTGGATGCGTTCCCAAAATTAGTTTGTTTTAAAAAACGTATTGAAGCTATCCCACAAATTGA

TAAGTACTTGAAATCCAGCAAGTATATAGCATGGCCTTTGCAGGGCTGGCAAGCCACGTTTGGTGGTGGCGACCATCCTC

CAAAATCGGATGGTTCAACTAGTGGTTCTGGTCATCACCATCACCATCACTCCATGAGCGATTACGACATCCCCACTACT

GAGAATCTTTATTTTCAGGGCGCCATGGCAGTAAAACATTTAATTGTGCTCAAATTTAAAGACGAAATTACTGAAGCACA

AAAAGAAGAGTTTTTCAAAACATACGTCAATCTGGTGAACATCATCCCGGCCATGAAAGACGTCTATTGGGGCAAAGACG

TAACGCAGAAAAACAAGGAAGAAGGTTATACCCACATCGTGGAGGTCACTTTCGAATCAGTCGAAACGATCCAGGATTAC

ATTATTCATCCGGCACATGTAGGGTTCGGCGATGTATATCGTAGCTTCTGGGAAAAACTCCTGATTTTTGACTATACCCC

GCGTAAATAAAGAAACTTGTTGACTTTGACGGCTAGCTCAGTCCTAGGTACAGTGCTAGCCGCAGAGAATTCACGGAAGG

AGCATGGCGATGCTGCCTCCGAGCGATAGCAAAGATCCGCGTCCGTGGCAGATTCTGAGCCAGGCACTGGGTTTTCCGAA

TTATGATCAAGAACTGTGGTGGCAGAATACCGCAGAAACCCTGAATCGTGTTCTGGAACAGTGTGATTATAGCGTTCATC

TGCAGTATAAATACCTGGCCTTCTACCACAAATATATCCTGCCGAGCCTGGGTCCGTTTCGTCGTCCGGGTGTTGAACCG

GAATATATCAGCGGTCTGAGTCATGGTGGTCATCCGCTGGAAATTAGCGTGAAAATTGATAAAAGCAAGACCATTTGTCG

TCTGGGTCTGCAGGCAATTGGTCCGCTGGCAGGCACCGCACGTGATCCGCTGAATAGCTTTGGTGATCGTGAACTGCTGA

AAAATCTGGCAACCCTGCTGCCGCATGTTGATCTGCGTCTGTTTGATCATTTTAATGCACAGGTTGGTCTGGATCGTGCA

CAGTGTGCAGTTGCAACCACCAAACTGATTAAAGAAAGCCATAATATTGTGTGCACCAGCCTGGATCTGAAAGATGGTGA

AGTTATTCCGAAAGTGTACTTTAGCACCATTCCTAAAGGTCTGGTTACCGAAACACCGCTGTTCGATCTGACCTTTGCAG

CAATTGAGCAGATGGAAGTGTATCATAAAGATGCACCGCTGCGTACCGCACTGAGCAGCCTGAAAGATTTTCTGCGTCCG

CGTGTTCCGACCGATGCAAGCATTACCCCTCCGCTGACCGGTCTGATTGGTGTTGATTGTATTGATCCGATGCTGAGCCG

TCTGAAAGTTTATCTGGCCACCTTTCGTATGGATCTGAGCCTGATTCGTGATTATTGGACCTTAGGTGGTCTGCTGACCG

ATGCCGGTACAATGAAAGGCCTGGAAATGGTTGAAACCCTGGCAAAAACACTGAAACTGGGTGATGAAGCATGTGAAACA

CTGGATGCAGAACGTCTGCCGTTTGGTATTAACTATGCAATGAAACCGGGTACAGCAGAACTGGCACCGCCTCAGATCTA

TTTTCCGCTGCTGGGTATTAATGATGGCTTTATTGCAGATGCCCTGGTGGAATTTTTTCAGTATATGGGTTGGGAAGATC

AGGCCAATCGTTATAAAGATGAACTGAAAGCCAAATTTCCGAACGTGGATATTAGCCAGACCAAAAATGTTCATCGTTGG

CTGGGTGTTGCATATAGCGAAACCAAAGGTCCGAGCATGAACATCTATTATGATGTTGTTGCAGGTAATGTGGCACGCGT

TTAAACGATCTTAAGTAGGCGCGGAAAATAATGGAGTTCGACTTCAACAAATACATGGACAGCAAAGCCATGACCGTTAA

TGAAGCACTGAATAAAGCAATTCCGCTGCGTTATCCGCAGAAAATCTATGAAAGCATGCGTTATAGCCTGCTGGCAGGCG

GTAAACGTGTTCGTCCGGTTCTGTGTATTGCAGCATGTGAACTGGTTGGTGGCACCGAAGAACTGGCAATTCCGACCGCA

TGTGCAATTGAAATGATTCATACCATGAGCCTGATGCATGATGATCTGCCGTGTATTGATAATGATGACCTGCGTCGTGG

TAAACCGACCAATCATAAAATCTTTGGTGAAGATACCGCAGTGACCGCAGGTAATGCACTGCATAGTTATGCATTTGAAC

ATATTGCAGTGAGCACCAGCAAAACCGTTGGTGCAGATCGTATTCTGCGTATGGTTAGCGAACTGGGTCGTGCAACCGGT

AGCGAAGGTGTTATGGGTGGTCAGATGGTTGATATTGCAAGTGAAGGTGATCCGAGCATTGATCTGCAGACCCTGGAATG

GATTCATATTCATAAAACCGCAATGCTGCTGGAATGTAGCGTTGTTTGTGGTGCAATTATTGGTGGTGCAAGCGAAATTG

TTATTGAACGTGCCCGTCGTTATGCACGTTGTGTTGGTCTGCTGTTTCAGGTTGTTGATGATATTCTGGATGTGACCAAA

AGCAGTGATGAACTGGGCAAAACCGCAGGCAAAGATCTGATTAGCGATAAAGCAACCTATCCGAAACTGATGGGTCTGGA

AAAAGCCAAAGAATTTTCAGATGAACTGCTGAATCGTGCCAAAGGTGAACTGAGCTGTTTTGATCCGGTTAAAGCAGCAC

CGCTGCTGGGTCTGGCAGATTATGTTGCATTTCGTCAGAATTAAGGGATCCAAACTCGAGTAAGGATCTCCAGGCATCAA

ATAAAACGAAAGGCTCAGTCGAAAGACTGGGCCTTTCGTTTTATCTGTTGTTTGTCGGTGAACGCTCTCTACTAGAGTCA

CACTGGCTCACCTTCGGGTGGGCCTTTCTGCGTTTATACCTAGGCTACAGCCGATAGTCTGGAACAGCGCACTTACGGGT

TGCTGCGCAACCCAAGTGCTACCGGCGCGGCAGCGTGACCCGTGTCGGCGGCTCCAACGGCTCGCCATCGTCCAGAAAAC

ACGGCTCATCGGGCATCGGCAGGCGCTGCTGCCCGCGCCGTTCCCATTCCTCCGTTTCGGTCAAGGCTGGCAGGTCTGGT

TCCATGCCCGGAATGCCGGGCTGGCTGGGCGGCTCCTCGCCGGGGCCGGTCGGTAGTTGCTGCTCGCCCGGATACAGGGT

CGGGATGCGGCGCAGGTCGCCATGCCCCAACAGCGATTCGTCCTGGTCGTCGTGATCAACCACCACGGCGGCACTGAACA

CCGACAGGCGCAACTGGTCGCGGGGCTGGCCCCACGCCACGCGGTCATTGACCACGTAGGCCAACACGGTGCCGGGGCCG

TTGAGCTTCACGACGGAGATCCAGCGCTCGGCCACCAAGTCCTTGACTGCGTATTGGACCGTCCGCAAAGAACGTCCGAT

GAGCTTGGAAAGTGTCTTCTGGCTGACCACCACGGCGTTCTGGTGGCCCATCTGCGCCACGAGGTGATGCAGCAGCATTG

CCGCCGTGGGTTTCCTCGCAATAAGCCCGGCCCACGCCTCATGCGCTTTGCGTTCCGTTTGCACCCAGTGACCGGGCTTG

TTCTTGGCTTGAATGCCGATTTCTCTGGACTGCGTGGCCATGCTTATCTCCATGCGGTAGGGGTGCCGCACGGTTGCGGC

ACCATGCGCAATCAGCTGCAACTTTTCGGCAGCGCGACAACAATTATGCGTTGCGTAAAAGTGGCAGTCAATTACAGATT

TTCTTTAACCTACGCAATGAGCTATTGCGGGGGGTGCCGCAATGAGCTGTTGCGTACCCCCCTTTTTTAAGTTGTTGATT

TTTAAGTCTTTCGCATTTCGCCCTATATCTAGTTCTTTGGTGCCCAAAGAAGGGCACCCCTGCGGGGTTCCCCCACGCCT

TCGGCGCGGCTCCCCCTCCGGCAAAAAGTGGCCCCTCCGGGGCTTGTTGATCGACTGCGCGGCCTTCGGCCTTGCCCAAG

GTGGCGCTGCCCCCTTGGAACCCCCGCACTCGCCGCCGTGAGGCTCGGGGGGCAGGCGGGCGGGCTTCGCCCTTCGACTG

CCCCCACTCGCATAGGCTTGGGTCGTTCCAGGCGCGTCAAGGCCAAGCCGCTGCGCGGTCGCTGCGCGAGCCTTGACCCG

CCTTCCACTTGGTGTCCAACCGGCAAGCGAAGCGCGCAGGCCGCAGGCCGGAGGCACTAGTGCTTGGATTCTCACCAATA

AAAAACGCCCGGCGGCAACCGAGCGTTCTGAACAAATCCAGATGGAGTTCTGAGGTCATTACTGGATCTATCAACAGGAG

TCCAAGCGAGCTCGATATCAAATTACGCCCCGCCCTGCCACTCATCGCAGTACTGTTGTAATTCATTAAGCATTCTGCCG

ACATGGAAGCCATCACAAACGGCATGATGAACCTGAATCGCCAGCGGCATCAGCACCTTGTCGCCTTGCGTATAATATTT

GCCCATGGTGAAAACGGGGGCGAAGAAGTTGTCCATATTGGCCACGTTTAAATCAAAACTGGTGAAACTCACCCAGGGAT

TGGCTGAGACGAAAAACATATTCTCAATAAACCCTTTAGGGAAATAGGCCAGGTTTTCACCGTAACACGCCACATCTTGC

GAATATATGTGTAGAAACTGCCGGAAATCGTCGTGGTATTCACTCCAGAGCGATGAAAACGTTTCAGTTTGCTCATGGAA

AACGGTGTAACAAGGGTGAACACTATCCCATATCACCAGCTCACCGTCTTTCATTGCCATACGAAATTCCGGATGAGCAT

TCATCAGGCGGGCAAGAATGTGAATAAAGGCCGGATAAAACTTGTGCTTATTTTTCTTTACGGTCTTTAAAAAGGCCGTA

ATATCCAGCTGAACGGTCTGGTTATAGGTACATTGAGCAACTGACTGAAATGCCTCAAAATGTTCTTTACGATGCCATTG

GGATATATCAACGGTGGTATATCCAGTGATTTTTTTCTCCATTTTAGCTTCCTTAGCTCCTGAAAATCTCGATAACTCAA

AAAATACGCCCGGTAGTGATCTTATTTCATTATGGTGAAAGTTGGAACCTCTTACGTGCCGATCAACGTCTCATTTTCGC

CAGATATC

>pBbB2c-CBGA_J23105 (Genbank: OQ725945)

GACGTCTTAAGACCCACTTTCACATTTAAGTTGTTTTTCTAATCCGCATATGATCAATTCAAGGCCGAATAAGAAGGCTG

GCTCTGCACCTTGGTGATCAAATAATTCGATAGCTTGTCGTAATAATGGCGGCATACTATCAGTAGTAGGTGTTTCCCTT

TCTTCTTTAGCGACTTGATGCTCTTGATCTTCCAATACGCAACCTAAAGTAAAATGCCCCACAGCGCTGAGTGCATATAA

TGCATTCTCTAGTGAAAAACCTTGTTGGCATAAAAAGGCTAATTGATTTTCGAGAGTTTCATACTGTTTTTCTGTAGGCC

GTGTACCTAAATGTACTTTTGCTCCATCGCGATGACTTAGTAAAGCACATCTAAAACTTTTAGCGTTATTACGTAAAAAA

TCTTGCCAGCTTTCCCCTTCTAAAGGGCAAAAGTGAGTATGGTGCCTATCTAACATCTCAATGGCTAAGGCGTCGAGCAA

AGCCCGCTTATTTTTTACATGCCAATACAATGTAGGCTGCTCTACACCTAGCTTCTGGGCGAGTTTACGGGTTGTTAAAC

CTTCGATTCCGACCTCATTAAGCAGCTCTAATGCGCTGTTAATCACTTTACTTTTATCTAATCTAGACATCATTAATTCC

TAATTTTTGTTGACACTCTATCGTTGATAGAGTTATTTTACCACTCCCTATCAGTGATAGAGAAAAGAATTCAAAAGATC

TTTTAAGAAGCCTTAGGTCAATCCACAAAAGCGGCGCATAAGGAAAACTCCCTAGATTCGAGGACCAACAATGAACCACC

TAAGAGCAGAAGGCCCAGCCTCCGTGCTGGCCATTGGTACCGCCAATCCGGAAAACATTTTATTGCAGGACGAATTTCCT

GATTATTATTTTCGTGTTACCAAATCGGAACACATGACCCAGCTGAAAGAAAAATTTCGCAAAATTTGTGACAAAAGTAT

GATACGGAAACGAAATTGTTTTCTGAATGAGGAACACTTGAAGCAAAACCCTCGCTTAGTCGAACATGAGATGCAGACAC

TGGATGCCCGCCAGGACATGCTCGTGGTTGAAGTGCCGAAATTAGGTAAAGACGCGTGCGCGAAGGCGATTAAAGAGTGG

GGTCAGCCGAAATCCAAGATTACACACTTAATTTTTACCTCAGCGTCGACTACCGATATGCCTGGTGCCGACTATCATTG

TGCCAAGTTGTTGGGCTTAAGCCCCTCGGTTAAGCGTGTGATGATGTACCAACTGGGTTGTTATGGCGGTGGCACGGTGC

TGAGAATTGCGAAAGATATTGCCGAGAATAATAAAGGCGCGCGTGTTCTGGCCGTTTGCTGTGATATCATGGCATGCCTG

TTTCGTGGTCCTTCTGAGAGCGACTTAGAACTGCTGGTCGGCCAGGCTATATTCGGGGATGGTGCGGCGGCAGTCATTGT

TGGCGCGGAACCCGATGAAAGCGTTGGCGAGCGCCCAATCTTCGAATTAGTAAGCACAGGTCAGACGATATTACCAAATA

GCGAAGGCACTATCGGGGGCCACATCCGCGAAGCTGGACTGATTTTTGACCTGCATAAAGATGTCCCGATGCTGATTAGT

AATAATATCGAGAAATGTCTGATTGAGGCATTTACCCCAATTGGAATTAGCGACTGGAACAGCATTTTCTGGATAACGCA

TCCGGGCGGCAAAGCTATCCTGGATAAAGTTGAGGAAAAGCTCCACCTGAAATCTGATAAGTTTGTAGATTCCCGCCACG

TGTTGAGCGAACATGGTAATATGAGCTCCTCAACAGTCCTATTCGTTATGGATGAACTGCGCAAACGCTCGCTCGAAGAA

GGTAAAAGTACCACCGGCGATGGCTTTGAATGGGGCGTGTTATTCGGTTTTGGCCCAGGCTTAACCGTTGAGCGTGTGGT

GGTACGATCGGTTCCTATAAAATATTGACTCTAGAAATAATTTTGTTTAACTTTAAGAAGGAGATATACATATGTCCCCT

ATACTAGGTTATTGGAAAATTAAGGGCCTTGTGCAACCCACTCGACTTCTTTTGGAATATCTTGAAGAAAAATATGAAGA

GCATTTGTATGAGCGCGATGAAGGTGATAAATGGCGAAACAAAAAGTTTGAATTGGGTTTGGAGTTTCCCAATCTTCCTT

ATTATATTGATGGTGATGTTAAATTAACACAGTCTATGGCCATCATACGTTATATAGCTGACAAGCACAACATGTTGGGT

GGTTGTCCAAAAGAGCGTGCAGAGATTTCAATGCTTGAAGGAGCGGTTTTGGATATTAGATACGGTGTTTCGAGAATTGC

ATATAGTAAAGACTTTGAAACTCTCAAAGTTGATTTTCTTAGCAAGCTACCTGAAATGCTGAAAATGTTCGAAGATCGTT

TATGTCATAAAACATATTTAAATGGTGATCATGTAACCCATCCTGACTTCATGTTGTATGACGCTCTTGATGTTGTTTTA

TACATGGACCCAATGTGCCTGGATGCGTTCCCAAAATTAGTTTGTTTTAAAAAACGTATTGAAGCTATCCCACAAATTGA

TAAGTACTTGAAATCCAGCAAGTATATAGCATGGCCTTTGCAGGGCTGGCAAGCCACGTTTGGTGGTGGCGACCATCCTC

CAAAATCGGATGGTTCAACTAGTGGTTCTGGTCATCACCATCACCATCACTCCATGAGCGATTACGACATCCCCACTACT

GAGAATCTTTATTTTCAGGGCGCCATGGCAGTAAAACATTTAATTGTGCTCAAATTTAAAGACGAAATTACTGAAGCACA

AAAAGAAGAGTTTTTCAAAACATACGTCAATCTGGTGAACATCATCCCGGCCATGAAAGACGTCTATTGGGGCAAAGACG

TAACGCAGAAAAACAAGGAAGAAGGTTATACCCACATCGTGGAGGTCACTTTCGAATCAGTCGAAACGATCCAGGATTAC

ATTATTCATCCGGCACATGTAGGGTTCGGCGATGTATATCGTAGCTTCTGGGAAAAACTCCTGATTTTTGACTATACCCC

GCGTAAATAAAGAAACTTGTTGACTTTTACGGCTAGCTCAGTCCTAGGTACTATGCTAGCCGCAGAGAATTCACGGAAGG

AGCATGGCGATGCTGCCTCCGAGCGATAGCAAAGATCCGCGTCCGTGGCAGATTCTGAGCCAGGCACTGGGTTTTCCGAA

TTATGATCAAGAACTGTGGTGGCAGAATACCGCAGAAACCCTGAATCGTGTTCTGGAACAGTGTGATTATAGCGTTCATC

TGCAGTATAAATACCTGGCCTTCTACCACAAATATATCCTGCCGAGCCTGGGTCCGTTTCGTCGTCCGGGTGTTGAACCG

GAATATATCAGCGGTCTGAGTCATGGTGGTCATCCGCTGGAAATTAGCGTGAAAATTGATAAAAGCAAGACCATTTGTCG

TCTGGGTCTGCAGGCAATTGGTCCGCTGGCAGGCACCGCACGTGATCCGCTGAATAGCTTTGGTGATCGTGAACTGCTGA

AAAATCTGGCAACCCTGCTGCCGCATGTTGATCTGCGTCTGTTTGATCATTTTAATGCACAGGTTGGTCTGGATCGTGCA

CAGTGTGCAGTTGCAACCACCAAACTGATTAAAGAAAGCCATAATATTGTGTGCACCAGCCTGGATCTGAAAGATGGTGA

AGTTATTCCGAAAGTGTACTTTAGCACCATTCCTAAAGGTCTGGTTACCGAAACACCGCTGTTCGATCTGACCTTTGCAG

CAATTGAGCAGATGGAAGTGTATCATAAAGATGCACCGCTGCGTACCGCACTGAGCAGCCTGAAAGATTTTCTGCGTCCG

CGTGTTCCGACCGATGCAAGCATTACCCCTCCGCTGACCGGTCTGATTGGTGTTGATTGTATTGATCCGATGCTGAGCCG

TCTGAAAGTTTATCTGGCCACCTTTCGTATGGATCTGAGCCTGATTCGTGATTATTGGACCTTAGGTGGTCTGCTGACCG

ATGCCGGTACAATGAAAGGCCTGGAAATGGTTGAAACCCTGGCAAAAACACTGAAACTGGGTGATGAAGCATGTGAAACA

CTGGATGCAGAACGTCTGCCGTTTGGTATTAACTATGCAATGAAACCGGGTACAGCAGAACTGGCACCGCCTCAGATCTA

TTTTCCGCTGCTGGGTATTAATGATGGCTTTATTGCAGATGCCCTGGTGGAATTTTTTCAGTATATGGGTTGGGAAGATC

AGGCCAATCGTTATAAAGATGAACTGAAAGCCAAATTTCCGAACGTGGATATTAGCCAGACCAAAAATGTTCATCGTTGG

CTGGGTGTTGCATATAGCGAAACCAAAGGTCCGAGCATGAACATCTATTATGATGTTGTTGCAGGTAATGTGGCACGCGT

TTAAACGATCTTAAGTAGGCGCGGAAAATAATGGAGTTCGACTTCAACAAATACATGGACAGCAAAGCCATGACCGTTAA

TGAAGCACTGAATAAAGCAATTCCGCTGCGTTATCCGCAGAAAATCTATGAAAGCATGCGTTATAGCCTGCTGGCAGGCG

GTAAACGTGTTCGTCCGGTTCTGTGTATTGCAGCATGTGAACTGGTTGGTGGCACCGAAGAACTGGCAATTCCGACCGCA

TGTGCAATTGAAATGATTCATACCATGAGCCTGATGCATGATGATCTGCCGTGTATTGATAATGATGACCTGCGTCGTGG

TAAACCGACCAATCATAAAATCTTTGGTGAAGATACCGCAGTGACCGCAGGTAATGCACTGCATAGTTATGCATTTGAAC

ATATTGCAGTGAGCACCAGCAAAACCGTTGGTGCAGATCGTATTCTGCGTATGGTTAGCGAACTGGGTCGTGCAACCGGT

AGCGAAGGTGTTATGGGTGGTCAGATGGTTGATATTGCAAGTGAAGGTGATCCGAGCATTGATCTGCAGACCCTGGAATG

GATTCATATTCATAAAACCGCAATGCTGCTGGAATGTAGCGTTGTTTGTGGTGCAATTATTGGTGGTGCAAGCGAAATTG

TTATTGAACGTGCCCGTCGTTATGCACGTTGTGTTGGTCTGCTGTTTCAGGTTGTTGATGATATTCTGGATGTGACCAAA

AGCAGTGATGAACTGGGCAAAACCGCAGGCAAAGATCTGATTAGCGATAAAGCAACCTATCCGAAACTGATGGGTCTGGA

AAAAGCCAAAGAATTTTCAGATGAACTGCTGAATCGTGCCAAAGGTGAACTGAGCTGTTTTGATCCGGTTAAAGCAGCAC

CGCTGCTGGGTCTGGCAGATTATGTTGCATTTCGTCAGAATTAAGGGATCCAAACTCGAGTAAGGATCTCCAGGCATCAA

ATAAAACGAAAGGCTCAGTCGAAAGACTGGGCCTTTCGTTTTATCTGTTGTTTGTCGGTGAACGCTCTCTACTAGAGTCA

CACTGGCTCACCTTCGGGTGGGCCTTTCTGCGTTTATACCTAGGCTACAGCCGATAGTCTGGAACAGCGCACTTACGGGT

TGCTGCGCAACCCAAGTGCTACCGGCGCGGCAGCGTGACCCGTGTCGGCGGCTCCAACGGCTCGCCATCGTCCAGAAAAC

ACGGCTCATCGGGCATCGGCAGGCGCTGCTGCCCGCGCCGTTCCCATTCCTCCGTTTCGGTCAAGGCTGGCAGGTCTGGT

TCCATGCCCGGAATGCCGGGCTGGCTGGGCGGCTCCTCGCCGGGGCCGGTCGGTAGTTGCTGCTCGCCCGGATACAGGGT

CGGGATGCGGCGCAGGTCGCCATGCCCCAACAGCGATTCGTCCTGGTCGTCGTGATCAACCACCACGGCGGCACTGAACA

CCGACAGGCGCAACTGGTCGCGGGGCTGGCCCCACGCCACGCGGTCATTGACCACGTAGGCCAACACGGTGCCGGGGCCG

TTGAGCTTCACGACGGAGATCCAGCGCTCGGCCACCAAGTCCTTGACTGCGTATTGGACCGTCCGCAAAGAACGTCCGAT

GAGCTTGGAAAGTGTCTTCTGGCTGACCACCACGGCGTTCTGGTGGCCCATCTGCGCCACGAGGTGATGCAGCAGCATTG

CCGCCGTGGGTTTCCTCGCAATAAGCCCGGCCCACGCCTCATGCGCTTTGCGTTCCGTTTGCACCCAGTGACCGGGCTTG

TTCTTGGCTTGAATGCCGATTTCTCTGGACTGCGTGGCCATGCTTATCTCCATGCGGTAGGGGTGCCGCACGGTTGCGGC

ACCATGCGCAATCAGCTGCAACTTTTCGGCAGCGCGACAACAATTATGCGTTGCGTAAAAGTGGCAGTCAATTACAGATT

TTCTTTAACCTACGCAATGAGCTATTGCGGGGGGTGCCGCAATGAGCTGTTGCGTACCCCCCTTTTTTAAGTTGTTGATT

TTTAAGTCTTTCGCATTTCGCCCTATATCTAGTTCTTTGGTGCCCAAAGAAGGGCACCCCTGCGGGGTTCCCCCACGCCT

TCGGCGCGGCTCCCCCTCCGGCAAAAAGTGGCCCCTCCGGGGCTTGTTGATCGACTGCGCGGCCTTCGGCCTTGCCCAAG

GTGGCGCTGCCCCCTTGGAACCCCCGCACTCGCCGCCGTGAGGCTCGGGGGGCAGGCGGGCGGGCTTCGCCCTTCGACTG

CCCCCACTCGCATAGGCTTGGGTCGTTCCAGGCGCGTCAAGGCCAAGCCGCTGCGCGGTCGCTGCGCGAGCCTTGACCCG

CCTTCCACTTGGTGTCCAACCGGCAAGCGAAGCGCGCAGGCCGCAGGCCGGAGGCACTAGTGCTTGGATTCTCACCAATA

AAAAACGCCCGGCGGCAACCGAGCGTTCTGAACAAATCCAGATGGAGTTCTGAGGTCATTACTGGATCTATCAACAGGAG

TCCAAGCGAGCTCGATATCAAATTACGCCCCGCCCTGCCACTCATCGCAGTACTGTTGTAATTCATTAAGCATTCTGCCG

ACATGGAAGCCATCACAAACGGCATGATGAACCTGAATCGCCAGCGGCATCAGCACCTTGTCGCCTTGCGTATAATATTT

GCCCATGGTGAAAACGGGGGCGAAGAAGTTGTCCATATTGGCCACGTTTAAATCAAAACTGGTGAAACTCACCCAGGGAT

TGGCTGAGACGAAAAACATATTCTCAATAAACCCTTTAGGGAAATAGGCCAGGTTTTCACCGTAACACGCCACATCTTGC

GAATATATGTGTAGAAACTGCCGGAAATCGTCGTGGTATTCACTCCAGAGCGATGAAAACGTTTCAGTTTGCTCATGGAA

AACGGTGTAACAAGGGTGAACACTATCCCATATCACCAGCTCACCGTCTTTCATTGCCATACGAAATTCCGGATGAGCAT

TCATCAGGCGGGCAAGAATGTGAATAAAGGCCGGATAAAACTTGTGCTTATTTTTCTTTACGGTCTTTAAAAAGGCCGTA

ATATCCAGCTGAACGGTCTGGTTATAGGTACATTGAGCAACTGACTGAAATGCCTCAAAATGTTCTTTACGATGCCATTG

GGATATATCAACGGTGGTATATCCAGTGATTTTTTTCTCCATTTTAGCTTCCTTAGCTCCTGAAAATCTCGATAACTCAA

AAAATACGCCCGGTAGTGATCTTATTTCATTATGGTGAAAGTTGGAACCTCTTACGTGCCGATCAACGTCTCATTTTCGC

CAGATATC

>pBbB2c-CBGA_J23116 (Genbank: OQ725946)

GACGTCTTAAGACCCACTTTCACATTTAAGTTGTTTTTCTAATCCGCATATGATCAATTCAAGGCCGAATAAGAAGGCTG

GCTCTGCACCTTGGTGATCAAATAATTCGATAGCTTGTCGTAATAATGGCGGCATACTATCAGTAGTAGGTGTTTCCCTT

TCTTCTTTAGCGACTTGATGCTCTTGATCTTCCAATACGCAACCTAAAGTAAAATGCCCCACAGCGCTGAGTGCATATAA

TGCATTCTCTAGTGAAAAACCTTGTTGGCATAAAAAGGCTAATTGATTTTCGAGAGTTTCATACTGTTTTTCTGTAGGCC

GTGTACCTAAATGTACTTTTGCTCCATCGCGATGACTTAGTAAAGCACATCTAAAACTTTTAGCGTTATTACGTAAAAAA

TCTTGCCAGCTTTCCCCTTCTAAAGGGCAAAAGTGAGTATGGTGCCTATCTAACATCTCAATGGCTAAGGCGTCGAGCAA

AGCCCGCTTATTTTTTACATGCCAATACAATGTAGGCTGCTCTACACCTAGCTTCTGGGCGAGTTTACGGGTTGTTAAAC

CTTCGATTCCGACCTCATTAAGCAGCTCTAATGCGCTGTTAATCACTTTACTTTTATCTAATCTAGACATCATTAATTCC

TAATTTTTGTTGACACTCTATCGTTGATAGAGTTATTTTACCACTCCCTATCAGTGATAGAGAAAAGAATTCAAAAGATC

TTTTAAGAAGCCTTAGGTCAATCCACAAAAGCGGCGCATAAGGAAAACTCCCTAGATTCGAGGACCAACAATGAACCACC

TAAGAGCAGAAGGCCCAGCCTCCGTGCTGGCCATTGGTACCGCCAATCCGGAAAACATTTTATTGCAGGACGAATTTCCT

GATTATTATTTTCGTGTTACCAAATCGGAACACATGACCCAGCTGAAAGAAAAATTTCGCAAAATTTGTGACAAAAGTAT

GATACGGAAACGAAATTGTTTTCTGAATGAGGAACACTTGAAGCAAAACCCTCGCTTAGTCGAACATGAGATGCAGACAC

TGGATGCCCGCCAGGACATGCTCGTGGTTGAAGTGCCGAAATTAGGTAAAGACGCGTGCGCGAAGGCGATTAAAGAGTGG

GGTCAGCCGAAATCCAAGATTACACACTTAATTTTTACCTCAGCGTCGACTACCGATATGCCTGGTGCCGACTATCATTG

TGCCAAGTTGTTGGGCTTAAGCCCCTCGGTTAAGCGTGTGATGATGTACCAACTGGGTTGTTATGGCGGTGGCACGGTGC

TGAGAATTGCGAAAGATATTGCCGAGAATAATAAAGGCGCGCGTGTTCTGGCCGTTTGCTGTGATATCATGGCATGCCTG

TTTCGTGGTCCTTCTGAGAGCGACTTAGAACTGCTGGTCGGCCAGGCTATATTCGGGGATGGTGCGGCGGCAGTCATTGT

TGGCGCGGAACCCGATGAAAGCGTTGGCGAGCGCCCAATCTTCGAATTAGTAAGCACAGGTCAGACGATATTACCAAATA

GCGAAGGCACTATCGGGGGCCACATCCGCGAAGCTGGACTGATTTTTGACCTGCATAAAGATGTCCCGATGCTGATTAGT

AATAATATCGAGAAATGTCTGATTGAGGCATTTACCCCAATTGGAATTAGCGACTGGAACAGCATTTTCTGGATAACGCA

TCCGGGCGGCAAAGCTATCCTGGATAAAGTTGAGGAAAAGCTCCACCTGAAATCTGATAAGTTTGTAGATTCCCGCCACG

TGTTGAGCGAACATGGTAATATGAGCTCCTCAACAGTCCTATTCGTTATGGATGAACTGCGCAAACGCTCGCTCGAAGAA

GGTAAAAGTACCACCGGCGATGGCTTTGAATGGGGCGTGTTATTCGGTTTTGGCCCAGGCTTAACCGTTGAGCGTGTGGT

GGTACGATCGGTTCCTATAAAATATTGACTCTAGAAATAATTTTGTTTAACTTTAAGAAGGAGATATACATATGTCCCCT

ATACTAGGTTATTGGAAAATTAAGGGCCTTGTGCAACCCACTCGACTTCTTTTGGAATATCTTGAAGAAAAATATGAAGA

GCATTTGTATGAGCGCGATGAAGGTGATAAATGGCGAAACAAAAAGTTTGAATTGGGTTTGGAGTTTCCCAATCTTCCTT

ATTATATTGATGGTGATGTTAAATTAACACAGTCTATGGCCATCATACGTTATATAGCTGACAAGCACAACATGTTGGGT

GGTTGTCCAAAAGAGCGTGCAGAGATTTCAATGCTTGAAGGAGCGGTTTTGGATATTAGATACGGTGTTTCGAGAATTGC

ATATAGTAAAGACTTTGAAACTCTCAAAGTTGATTTTCTTAGCAAGCTACCTGAAATGCTGAAAATGTTCGAAGATCGTT

TATGTCATAAAACATATTTAAATGGTGATCATGTAACCCATCCTGACTTCATGTTGTATGACGCTCTTGATGTTGTTTTA

TACATGGACCCAATGTGCCTGGATGCGTTCCCAAAATTAGTTTGTTTTAAAAAACGTATTGAAGCTATCCCACAAATTGA

TAAGTACTTGAAATCCAGCAAGTATATAGCATGGCCTTTGCAGGGCTGGCAAGCCACGTTTGGTGGTGGCGACCATCCTC

CAAAATCGGATGGTTCAACTAGTGGTTCTGGTCATCACCATCACCATCACTCCATGAGCGATTACGACATCCCCACTACT

GAGAATCTTTATTTTCAGGGCGCCATGGCAGTAAAACATTTAATTGTGCTCAAATTTAAAGACGAAATTACTGAAGCACA

AAAAGAAGAGTTTTTCAAAACATACGTCAATCTGGTGAACATCATCCCGGCCATGAAAGACGTCTATTGGGGCAAAGACG

TAACGCAGAAAAACAAGGAAGAAGGTTATACCCACATCGTGGAGGTCACTTTCGAATCAGTCGAAACGATCCAGGATTAC

ATTATTCATCCGGCACATGTAGGGTTCGGCGATGTATATCGTAGCTTCTGGGAAAAACTCCTGATTTTTGACTATACCCC

GCGTAAATAAAGAAACTTGTTGACTTTGACAGCTAGCTCAGTCCTAGGGACTATGCTAGCCGCAGAGAATTCACGGAAGG

AGCATGGCGATGCTGCCTCCGAGCGATAGCAAAGATCCGCGTCCGTGGCAGATTCTGAGCCAGGCACTGGGTTTTCCGAA

TTATGATCAAGAACTGTGGTGGCAGAATACCGCAGAAACCCTGAATCGTGTTCTGGAACAGTGTGATTATAGCGTTCATC

TGCAGTATAAATACCTGGCCTTCTACCACAAATATATCCTGCCGAGCCTGGGTCCGTTTCGTCGTCCGGGTGTTGAACCG

GAATATATCAGCGGTCTGAGTCATGGTGGTCATCCGCTGGAAATTAGCGTGAAAATTGATAAAAGCAAGACCATTTGTCG

TCTGGGTCTGCAGGCAATTGGTCCGCTGGCAGGCACCGCACGTGATCCGCTGAATAGCTTTGGTGATCGTGAACTGCTGA

AAAATCTGGCAACCCTGCTGCCGCATGTTGATCTGCGTCTGTTTGATCATTTTAATGCACAGGTTGGTCTGGATCGTGCA

CAGTGTGCAGTTGCAACCACCAAACTGATTAAAGAAAGCCATAATATTGTGTGCACCAGCCTGGATCTGAAAGATGGTGA

AGTTATTCCGAAAGTGTACTTTAGCACCATTCCTAAAGGTCTGGTTACCGAAACACCGCTGTTCGATCTGACCTTTGCAG

CAATTGAGCAGATGGAAGTGTATCATAAAGATGCACCGCTGCGTACCGCACTGAGCAGCCTGAAAGATTTTCTGCGTCCG

CGTGTTCCGACCGATGCAAGCATTACCCCTCCGCTGACCGGTCTGATTGGTGTTGATTGTATTGATCCGATGCTGAGCCG

TCTGAAAGTTTATCTGGCCACCTTTCGTATGGATCTGAGCCTGATTCGTGATTATTGGACCTTAGGTGGTCTGCTGACCG

ATGCCGGTACAATGAAAGGCCTGGAAATGGTTGAAACCCTGGCAAAAACACTGAAACTGGGTGATGAAGCATGTGAAACA

CTGGATGCAGAACGTCTGCCGTTTGGTATTAACTATGCAATGAAACCGGGTACAGCAGAACTGGCACCGCCTCAGATCTA

TTTTCCGCTGCTGGGTATTAATGATGGCTTTATTGCAGATGCCCTGGTGGAATTTTTTCAGTATATGGGTTGGGAAGATC

AGGCCAATCGTTATAAAGATGAACTGAAAGCCAAATTTCCGAACGTGGATATTAGCCAGACCAAAAATGTTCATCGTTGG

CTGGGTGTTGCATATAGCGAAACCAAAGGTCCGAGCATGAACATCTATTATGATGTTGTTGCAGGTAATGTGGCACGCGT

TTAAACGATCTTAAGTAGGCGCGGAAAATAATGGAGTTCGACTTCAACAAATACATGGACAGCAAAGCCATGACCGTTAA

TGAAGCACTGAATAAAGCAATTCCGCTGCGTTATCCGCAGAAAATCTATGAAAGCATGCGTTATAGCCTGCTGGCAGGCG

GTAAACGTGTTCGTCCGGTTCTGTGTATTGCAGCATGTGAACTGGTTGGTGGCACCGAAGAACTGGCAATTCCGACCGCA

TGTGCAATTGAAATGATTCATACCATGAGCCTGATGCATGATGATCTGCCGTGTATTGATAATGATGACCTGCGTCGTGG

TAAACCGACCAATCATAAAATCTTTGGTGAAGATACCGCAGTGACCGCAGGTAATGCACTGCATAGTTATGCATTTGAAC

ATATTGCAGTGAGCACCAGCAAAACCGTTGGTGCAGATCGTATTCTGCGTATGGTTAGCGAACTGGGTCGTGCAACCGGT

AGCGAAGGTGTTATGGGTGGTCAGATGGTTGATATTGCAAGTGAAGGTGATCCGAGCATTGATCTGCAGACCCTGGAATG

GATTCATATTCATAAAACCGCAATGCTGCTGGAATGTAGCGTTGTTTGTGGTGCAATTATTGGTGGTGCAAGCGAAATTG

TTATTGAACGTGCCCGTCGTTATGCACGTTGTGTTGGTCTGCTGTTTCAGGTTGTTGATGATATTCTGGATGTGACCAAA

AGCAGTGATGAACTGGGCAAAACCGCAGGCAAAGATCTGATTAGCGATAAAGCAACCTATCCGAAACTGATGGGTCTGGA

AAAAGCCAAAGAATTTTCAGATGAACTGCTGAATCGTGCCAAAGGTGAACTGAGCTGTTTTGATCCGGTTAAAGCAGCAC

CGCTGCTGGGTCTGGCAGATTATGTTGCATTTCGTCAGAATTAAGGGATCCAAACTCGAGTAAGGATCTCCAGGCATCAA

ATAAAACGAAAGGCTCAGTCGAAAGACTGGGCCTTTCGTTTTATCTGTTGTTTGTCGGTGAACGCTCTCTACTAGAGTCA

CACTGGCTCACCTTCGGGTGGGCCTTTCTGCGTTTATACCTAGGCTACAGCCGATAGTCTGGAACAGCGCACTTACGGGT

TGCTGCGCAACCCAAGTGCTACCGGCGCGGCAGCGTGACCCGTGTCGGCGGCTCCAACGGCTCGCCATCGTCCAGAAAAC

ACGGCTCATCGGGCATCGGCAGGCGCTGCTGCCCGCGCCGTTCCCATTCCTCCGTTTCGGTCAAGGCTGGCAGGTCTGGT

TCCATGCCCGGAATGCCGGGCTGGCTGGGCGGCTCCTCGCCGGGGCCGGTCGGTAGTTGCTGCTCGCCCGGATACAGGGT

CGGGATGCGGCGCAGGTCGCCATGCCCCAACAGCGATTCGTCCTGGTCGTCGTGATCAACCACCACGGCGGCACTGAACA

CCGACAGGCGCAACTGGTCGCGGGGCTGGCCCCACGCCACGCGGTCATTGACCACGTAGGCCAACACGGTGCCGGGGCCG

TTGAGCTTCACGACGGAGATCCAGCGCTCGGCCACCAAGTCCTTGACTGCGTATTGGACCGTCCGCAAAGAACGTCCGAT

GAGCTTGGAAAGTGTCTTCTGGCTGACCACCACGGCGTTCTGGTGGCCCATCTGCGCCACGAGGTGATGCAGCAGCATTG

CCGCCGTGGGTTTCCTCGCAATAAGCCCGGCCCACGCCTCATGCGCTTTGCGTTCCGTTTGCACCCAGTGACCGGGCTTG

TTCTTGGCTTGAATGCCGATTTCTCTGGACTGCGTGGCCATGCTTATCTCCATGCGGTAGGGGTGCCGCACGGTTGCGGC

ACCATGCGCAATCAGCTGCAACTTTTCGGCAGCGCGACAACAATTATGCGTTGCGTAAAAGTGGCAGTCAATTACAGATT

TTCTTTAACCTACGCAATGAGCTATTGCGGGGGGTGCCGCAATGAGCTGTTGCGTACCCCCCTTTTTTAAGTTGTTGATT

TTTAAGTCTTTCGCATTTCGCCCTATATCTAGTTCTTTGGTGCCCAAAGAAGGGCACCCCTGCGGGGTTCCCCCACGCCT

TCGGCGCGGCTCCCCCTCCGGCAAAAAGTGGCCCCTCCGGGGCTTGTTGATCGACTGCGCGGCCTTCGGCCTTGCCCAAG

GTGGCGCTGCCCCCTTGGAACCCCCGCACTCGCCGCCGTGAGGCTCGGGGGGCAGGCGGGCGGGCTTCGCCCTTCGACTG

CCCCCACTCGCATAGGCTTGGGTCGTTCCAGGCGCGTCAAGGCCAAGCCGCTGCGCGGTCGCTGCGCGAGCCTTGACCCG

CCTTCCACTTGGTGTCCAACCGGCAAGCGAAGCGCGCAGGCCGCAGGCCGGAGGCACTAGTGCTTGGATTCTCACCAATA

AAAAACGCCCGGCGGCAACCGAGCGTTCTGAACAAATCCAGATGGAGTTCTGAGGTCATTACTGGATCTATCAACAGGAG

TCCAAGCGAGCTCGATATCAAATTACGCCCCGCCCTGCCACTCATCGCAGTACTGTTGTAATTCATTAAGCATTCTGCCG

ACATGGAAGCCATCACAAACGGCATGATGAACCTGAATCGCCAGCGGCATCAGCACCTTGTCGCCTTGCGTATAATATTT

GCCCATGGTGAAAACGGGGGCGAAGAAGTTGTCCATATTGGCCACGTTTAAATCAAAACTGGTGAAACTCACCCAGGGAT

TGGCTGAGACGAAAAACATATTCTCAATAAACCCTTTAGGGAAATAGGCCAGGTTTTCACCGTAACACGCCACATCTTGC

GAATATATGTGTAGAAACTGCCGGAAATCGTCGTGGTATTCACTCCAGAGCGATGAAAACGTTTCAGTTTGCTCATGGAA

AACGGTGTAACAAGGGTGAACACTATCCCATATCACCAGCTCACCGTCTTTCATTGCCATACGAAATTCCGGATGAGCAT

TCATCAGGCGGGCAAGAATGTGAATAAAGGCCGGATAAAACTTGTGCTTATTTTTCTTTACGGTCTTTAAAAAGGCCGTA

ATATCCAGCTGAACGGTCTGGTTATAGGTACATTGAGCAACTGACTGAAATGCCTCAAAATGTTCTTTACGATGCCATTG

GGATATATCAACGGTGGTATATCCAGTGATTTTTTTCTCCATTTTAGCTTCCTTAGCTCCTGAAAATCTCGATAACTCAA

AAAATACGCCCGGTAGTGATCTTATTTCATTATGGTGAAAGTTGGAACCTCTTACGTGCCGATCAACGTCTCATTTTCGC

CAGATATC

>pBbB2c-CBGA_J23119(7688 bp) (Genbank: OQ725947)

GACGTCTTAAGACCCACTTTCACATTTAAGTTGTTTTTCTAATCCGCATATGATCAATTCAAGGCCGAATAAGAAGGCTG

GCTCTGCACCTTGGTGATCAAATAATTCGATAGCTTGTCGTAATAATGGCGGCATACTATCAGTAGTAGGTGTTTCCCTT

TCTTCTTTAGCGACTTGATGCTCTTGATCTTCCAATACGCAACCTAAAGTAAAATGCCCCACAGCGCTGAGTGCATATAA

TGCATTCTCTAGTGAAAAACCTTGTTGGCATAAAAAGGCTAATTGATTTTCGAGAGTTTCATACTGTTTTTCTGTAGGCC

GTGTACCTAAATGTACTTTTGCTCCATCGCGATGACTTAGTAAAGCACATCTAAAACTTTTAGCGTTATTACGTAAAAAA

TCTTGCCAGCTTTCCCCTTCTAAAGGGCAAAAGTGAGTATGGTGCCTATCTAACATCTCAATGGCTAAGGCGTCGAGCAA

AGCCCGCTTATTTTTTACATGCCAATACAATGTAGGCTGCTCTACACCTAGCTTCTGGGCGAGTTTACGGGTTGTTAAAC

CTTCGATTCCGACCTCATTAAGCAGCTCTAATGCGCTGTTAATCACTTTACTTTTATCTAATCTAGACATCATTAATTCC

TAATTTTTGTTGACACTCTATCGTTGATAGAGTTATTTTACCACTCCCTATCAGTGATAGAGAAAAGAATTCAAAAGATC

TTTTAAGAAGCCTTAGGTCAATCCACAAAAGCGGCGCATAAGGAAAACTCCCTAGATTCGAGGACCAACAATGAACCACC

TAAGAGCAGAAGGCCCAGCCTCCGTGCTGGCCATTGGTACCGCCAATCCGGAAAACATTTTATTGCAGGACGAATTTCCT

GATTATTATTTTCGTGTTACCAAATCGGAACACATGACCCAGCTGAAAGAAAAATTTCGCAAAATTTGTGACAAAAGTAT

GATACGGAAACGAAATTGTTTTCTGAATGAGGAACACTTGAAGCAAAACCCTCGCTTAGTCGAACATGAGATGCAGACAC

TGGATGCCCGCCAGGACATGCTCGTGGTTGAAGTGCCGAAATTAGGTAAAGACGCGTGCGCGAAGGCGATTAAAGAGTGG

GGTCAGCCGAAATCCAAGATTACACACTTAATTTTTACCTCAGCGTCGACTACCGATATGCCTGGTGCCGACTATCATTG

TGCCAAGTTGTTGGGCTTAAGCCCCTCGGTTAAGCGTGTGATGATGTACCAACTGGGTTGTTATGGCGGTGGCACGGTGC

TGAGAATTGCGAAAGATATTGCCGAGAATAATAAAGGCGCGCGTGTTCTGGCCGTTTGCTGTGATATCATGGCATGCCTG

TTTCGTGGTCCTTCTGAGAGCGACTTAGAACTGCTGGTCGGCCAGGCTATATTCGGGGATGGTGCGGCGGCAGTCATTGT

TGGCGCGGAACCCGATGAAAGCGTTGGCGAGCGCCCAATCTTCGAATTAGTAAGCACAGGTCAGACGATATTACCAAATA

GCGAAGGCACTATCGGGGGCCACATCCGCGAAGCTGGACTGATTTTTGACCTGCATAAAGATGTCCCGATGCTGATTAGT

AATAATATCGAGAAATGTCTGATTGAGGCATTTACCCCAATTGGAATTAGCGACTGGAACAGCATTTTCTGGATAACGCA

TCCGGGCGGCAAAGCTATCCTGGATAAAGTTGAGGAAAAGCTCCACCTGAAATCTGATAAGTTTGTAGATTCCCGCCACG

TGTTGAGCGAACATGGTAATATGAGCTCCTCAACAGTCCTATTCGTTATGGATGAACTGCGCAAACGCTCGCTCGAAGAA

GGTAAAAGTACCACCGGCGATGGCTTTGAATGGGGCGTGTTATTCGGTTTTGGCCCAGGCTTAACCGTTGAGCGTGTGGT

GGTACGATCGGTTCCTATAAAATATTGACTCTAGAAATAATTTTGTTTAACTTTAAGAAGGAGATATACATATGTCCCCT

ATACTAGGTTATTGGAAAATTAAGGGCCTTGTGCAACCCACTCGACTTCTTTTGGAATATCTTGAAGAAAAATATGAAGA

GCATTTGTATGAGCGCGATGAAGGTGATAAATGGCGAAACAAAAAGTTTGAATTGGGTTTGGAGTTTCCCAATCTTCCTT

ATTATATTGATGGTGATGTTAAATTAACACAGTCTATGGCCATCATACGTTATATAGCTGACAAGCACAACATGTTGGGT

GGTTGTCCAAAAGAGCGTGCAGAGATTTCAATGCTTGAAGGAGCGGTTTTGGATATTAGATACGGTGTTTCGAGAATTGC

ATATAGTAAAGACTTTGAAACTCTCAAAGTTGATTTTCTTAGCAAGCTACCTGAAATGCTGAAAATGTTCGAAGATCGTT

TATGTCATAAAACATATTTAAATGGTGATCATGTAACCCATCCTGACTTCATGTTGTATGACGCTCTTGATGTTGTTTTA

TACATGGACCCAATGTGCCTGGATGCGTTCCCAAAATTAGTTTGTTTTAAAAAACGTATTGAAGCTATCCCACAAATTGA

TAAGTACTTGAAATCCAGCAAGTATATAGCATGGCCTTTGCAGGGCTGGCAAGCCACGTTTGGTGGTGGCGACCATCCTC

CAAAATCGGATGGTTCAACTAGTGGTTCTGGTCATCACCATCACCATCACTCCATGAGCGATTACGACATCCCCACTACT

GAGAATCTTTATTTTCAGGGCGCCATGGCAGTAAAACATTTAATTGTGCTCAAATTTAAAGACGAAATTACTGAAGCACA

AAAAGAAGAGTTTTTCAAAACATACGTCAATCTGGTGAACATCATCCCGGCCATGAAAGACGTCTATTGGGGCAAAGACG

TAACGCAGAAAAACAAGGAAGAAGGTTATACCCACATCGTGGAGGTCACTTTCGAATCAGTCGAAACGATCCAGGATTAC

ATTATTCATCCGGCACATGTAGGGTTCGGCGATGTATATCGTAGCTTCTGGGAAAAACTCCTGATTTTTGACTATACCCC

GCGTAAATAAAGAAACTTGTTGACTTTGACAGCTAGCTCAGTCCTAGGTATAATGCTAGCCGCAGAGAATTCACGGAAGG

AGCATGGCGATGCTGCCTCCGAGCGATAGCAAAGATCCGCGTCCGTGGCAGATTCTGAGCCAGGCACTGGGTTTTCCGAA

TTATGATCAAGAACTGTGGTGGCAGAATACCGCAGAAACCCTGAATCGTGTTCTGGAACAGTGTGATTATAGCGTTCATC

TGCAGTATAAATACCTGGCCTTCTACCACAAATATATCCTGCCGAGCCTGGGTCCGTTTCGTCGTCCGGGTGTTGAACCG

GAATATATCAGCGGTCTGAGTCATGGTGGTCATCCGCTGGAAATTAGCGTGAAAATTGATAAAAGCAAGACCATTTGTCG

TCTGGGTCTGCAGGCAATTGGTCCGCTGGCAGGCACCGCACGTGATCCGCTGAATAGCTTTGGTGATCGTGAACTGCTGA

AAAATCTGGCAACCCTGCTGCCGCATGTTGATCTGCGTCTGTTTGATCATTTTAATGCACAGGTTGGTCTGGATCGTGCA

CAGTGTGCAGTTGCAACCACCAAACTGATTAAAGAAAGCCATAATATTGTGTGCACCAGCCTGGATCTGAAAGATGGTGA

AGTTATTCCGAAAGTGTACTTTAGCACCATTCCTAAAGGTCTGGTTACCGAAACACCGCTGTTCGATCTGACCTTTGCAG

CAATTGAGCAGATGGAAGTGTATCATAAAGATGCACCGCTGCGTACCGCACTGAGCAGCCTGAAAGATTTTCTGCGTCCG

CGTGTTCCGACCGATGCAAGCATTACCCCTCCGCTGACCGGTCTGATTGGTGTTGATTGTATTGATCCGATGCTGAGCCG

TCTGAAAGTTTATCTGGCCACCTTTCGTATGGATCTGAGCCTGATTCGTGATTATTGGACCTTAGGTGGTCTGCTGACCG

ATGCCGGTACAATGAAAGGCCTGGAAATGGTTGAAACCCTGGCAAAAACACTGAAACTGGGTGATGAAGCATGTGAAACA

CTGGATGCAGAACGTCTGCCGTTTGGTATTAACTATGCAATGAAACCGGGTACAGCAGAACTGGCACCGCCTCAGATCTA

TTTTCCGCTGCTGGGTATTAATGATGGCTTTATTGCAGATGCCCTGGTGGAATTTTTTCAGTATATGGGTTGGGAAGATC

AGGCCAATCGTTATAAAGATGAACTGAAAGCCAAATTTCCGAACGTGGATATTAGCCAGACCAAAAATGTTCATCGTTGG

CTGGGTGTTGCATATAGCGAAACCAAAGGTCCGAGCATGAACATCTATTATGATGTTGTTGCAGGTAATGTGGCACGCGT

TTAAACGATCTTAAGTAGGCGCGGAAAATAATGGAGTTCGACTTCAACAAATACATGGACAGCAAAGCCATGACCGTTAA

TGAAGCACTGAATAAAGCAATTCCGCTGCGTTATCCGCAGAAAATCTATGAAAGCATGCGTTATAGCCTGCTGGCAGGCG

GTAAACGTGTTCGTCCGGTTCTGTGTATTGCAGCATGTGAACTGGTTGGTGGCACCGAAGAACTGGCAATTCCGACCGCA

TGTGCAATTGAAATGATTCATACCATGAGCCTGATGCATGATGATCTGCCGTGTATTGATAATGATGACCTGCGTCGTGG

TAAACCGACCAATCATAAAATCTTTGGTGAAGATACCGCAGTGACCGCAGGTAATGCACTGCATAGTTATGCATTTGAAC

ATATTGCAGTGAGCACCAGCAAAACCGTTGGTGCAGATCGTATTCTGCGTATGGTTAGCGAACTGGGTCGTGCAACCGGT

AGCGAAGGTGTTATGGGTGGTCAGATGGTTGATATTGCAAGTGAAGGTGATCCGAGCATTGATCTGCAGACCCTGGAATG

GATTCATATTCATAAAACCGCAATGCTGCTGGAATGTAGCGTTGTTTGTGGTGCAATTATTGGTGGTGCAAGCGAAATTG

TTATTGAACGTGCCCGTCGTTATGCACGTTGTGTTGGTCTGCTGTTTCAGGTTGTTGATGATATTCTGGATGTGACCAAA

AGCAGTGATGAACTGGGCAAAACCGCAGGCAAAGATCTGATTAGCGATAAAGCAACCTATCCGAAACTGATGGGTCTGGA

AAAAGCCAAAGAATTTTCAGATGAACTGCTGAATCGTGCCAAAGGTGAACTGAGCTGTTTTGATCCGGTTAAAGCAGCAC

CGCTGCTGGGTCTGGCAGATTATGTTGCATTTCGTCAGAATTAAGGGATCCAAACTCGAGTAAGGATCTCCAGGCATCAA

ATAAAACGAAAGGCTCAGTCGAAAGACTGGGCCTTTCGTTTTATCTGTTGTTTGTCGGTGAACGCTCTCTACTAGAGTCA

CACTGGCTCACCTTCGGGTGGGCCTTTCTGCGTTTATACCTAGGCTACAGCCGATAGTCTGGAACAGCGCACTTACGGGT

TGCTGCGCAACCCAAGTGCTACCGGCGCGGCAGCGTGACCCGTGTCGGCGGCTCCAACGGCTCGCCATCGTCCAGAAAAC

ACGGCTCATCGGGCATCGGCAGGCGCTGCTGCCCGCGCCGTTCCCATTCCTCCGTTTCGGTCAAGGCTGGCAGGTCTGGT

TCCATGCCCGGAATGCCGGGCTGGCTGGGCGGCTCCTCGCCGGGGCCGGTCGGTAGTTGCTGCTCGCCCGGATACAGGGT

CGGGATGCGGCGCAGGTCGCCATGCCCCAACAGCGATTCGTCCTGGTCGTCGTGATCAACCACCACGGCGGCACTGAACA

CCGACAGGCGCAACTGGTCGCGGGGCTGGCCCCACGCCACGCGGTCATTGACCACGTAGGCCAACACGGTGCCGGGGCCG

TTGAGCTTCACGACGGAGATCCAGCGCTCGGCCACCAAGTCCTTGACTGCGTATTGGACCGTCCGCAAAGAACGTCCGAT

GAGCTTGGAAAGTGTCTTCTGGCTGACCACCACGGCGTTCTGGTGGCCCATCTGCGCCACGAGGTGATGCAGCAGCATTG

CCGCCGTGGGTTTCCTCGCAATAAGCCCGGCCCACGCCTCATGCGCTTTGCGTTCCGTTTGCACCCAGTGACCGGGCTTG

TTCTTGGCTTGAATGCCGATTTCTCTGGACTGCGTGGCCATGCTTATCTCCATGCGGTAGGGGTGCCGCACGGTTGCGGC

ACCATGCGCAATCAGCTGCAACTTTTCGGCAGCGCGACAACAATTATGCGTTGCGTAAAAGTGGCAGTCAATTACAGATT

TTCTTTAACCTACGCAATGAGCTATTGCGGGGGGTGCCGCAATGAGCTGTTGCGTACCCCCCTTTTTTAAGTTGTTGATT

TTTAAGTCTTTCGCATTTCGCCCTATATCTAGTTCTTTGGTGCCCAAAGAAGGGCACCCCTGCGGGGTTCCCCCACGCCT

TCGGCGCGGCTCCCCCTCCGGCAAAAAGTGGCCCCTCCGGGGCTTGTTGATCGACTGCGCGGCCTTCGGCCTTGCCCAAG

GTGGCGCTGCCCCCTTGGAACCCCCGCACTCGCCGCCGTGAGGCTCGGGGGGCAGGCGGGCGGGCTTCGCCCTTCGACTG

CCCCCACTCGCATAGGCTTGGGTCGTTCCAGGCGCGTCAAGGCCAAGCCGCTGCGCGGTCGCTGCGCGAGCCTTGACCCG

CCTTCCACTTGGTGTCCAACCGGCAAGCGAAGCGCGCAGGCCGCAGGCCGGAGGCACTAGTGCTTGGATTCTCACCAATA

AAAAACGCCCGGCGGCAACCGAGCGTTCTGAACAAATCCAGATGGAGTTCTGAGGTCATTACTGGATCTATCAACAGGAG

TCCAAGCGAGCTCGATATCAAATTACGCCCCGCCCTGCCACTCATCGCAGTACTGTTGTAATTCATTAAGCATTCTGCCG

ACATGGAAGCCATCACAAACGGCATGATGAACCTGAATCGCCAGCGGCATCAGCACCTTGTCGCCTTGCGTATAATATTT

GCCCATGGTGAAAACGGGGGCGAAGAAGTTGTCCATATTGGCCACGTTTAAATCAAAACTGGTGAAACTCACCCAGGGAT

TGGCTGAGACGAAAAACATATTCTCAATAAACCCTTTAGGGAAATAGGCCAGGTTTTCACCGTAACACGCCACATCTTGC

GAATATATGTGTAGAAACTGCCGGAAATCGTCGTGGTATTCACTCCAGAGCGATGAAAACGTTTCAGTTTGCTCATGGAA

AACGGTGTAACAAGGGTGAACACTATCCCATATCACCAGCTCACCGTCTTTCATTGCCATACGAAATTCCGGATGAGCAT

TCATCAGGCGGGCAAGAATGTGAATAAAGGCCGGATAAAACTTGTGCTTATTTTTCTTTACGGTCTTTAAAAAGGCCGTA

ATATCCAGCTGAACGGTCTGGTTATAGGTACATTGAGCAACTGACTGAAATGCCTCAAAATGTTCTTTACGATGCCATTG

GGATATATCAACGGTGGTATATCCAGTGATTTTTTTCTCCATTTTAGCTTCCTTAGCTCCTGAAAATCTCGATAACTCAA

AAAATACGCCCGGTAGTGATCTTATTTCATTATGGTGAAAGTTGGAACCTCTTACGTGCCGATCAACGTCTCATTTTCGC

CAGATATC

>pBbB2c-CBGA_J23150(7688 bp) (Genbank: OQ725948)

GACGTCTTAAGACCCACTTTCACATTTAAGTTGTTTTTCTAATCCGCATATGATCAATTCAAGGCCGAATAAGAAGGCTG

GCTCTGCACCTTGGTGATCAAATAATTCGATAGCTTGTCGTAATAATGGCGGCATACTATCAGTAGTAGGTGTTTCCCTT

TCTTCTTTAGCGACTTGATGCTCTTGATCTTCCAATACGCAACCTAAAGTAAAATGCCCCACAGCGCTGAGTGCATATAA

TGCATTCTCTAGTGAAAAACCTTGTTGGCATAAAAAGGCTAATTGATTTTCGAGAGTTTCATACTGTTTTTCTGTAGGCC

GTGTACCTAAATGTACTTTTGCTCCATCGCGATGACTTAGTAAAGCACATCTAAAACTTTTAGCGTTATTACGTAAAAAA

TCTTGCCAGCTTTCCCCTTCTAAAGGGCAAAAGTGAGTATGGTGCCTATCTAACATCTCAATGGCTAAGGCGTCGAGCAA

AGCCCGCTTATTTTTTACATGCCAATACAATGTAGGCTGCTCTACACCTAGCTTCTGGGCGAGTTTACGGGTTGTTAAAC

CTTCGATTCCGACCTCATTAAGCAGCTCTAATGCGCTGTTAATCACTTTACTTTTATCTAATCTAGACATCATTAATTCC

TAATTTTTGTTGACACTCTATCGTTGATAGAGTTATTTTACCACTCCCTATCAGTGATAGAGAAAAGAATTCAAAAGATC

TTTTAAGAAGCCTTAGGTCAATCCACAAAAGCGGCGCATAAGGAAAACTCCCTAGATTCGAGGACCAACAATGAACCACC

TAAGAGCAGAAGGCCCAGCCTCCGTGCTGGCCATTGGTACCGCCAATCCGGAAAACATTTTATTGCAGGACGAATTTCCT

GATTATTATTTTCGTGTTACCAAATCGGAACACATGACCCAGCTGAAAGAAAAATTTCGCAAAATTTGTGACAAAAGTAT

GATACGGAAACGAAATTGTTTTCTGAATGAGGAACACTTGAAGCAAAACCCTCGCTTAGTCGAACATGAGATGCAGACAC

TGGATGCCCGCCAGGACATGCTCGTGGTTGAAGTGCCGAAATTAGGTAAAGACGCGTGCGCGAAGGCGATTAAAGAGTGG

GGTCAGCCGAAATCCAAGATTACACACTTAATTTTTACCTCAGCGTCGACTACCGATATGCCTGGTGCCGACTATCATTG

TGCCAAGTTGTTGGGCTTAAGCCCCTCGGTTAAGCGTGTGATGATGTACCAACTGGGTTGTTATGGCGGTGGCACGGTGC

TGAGAATTGCGAAAGATATTGCCGAGAATAATAAAGGCGCGCGTGTTCTGGCCGTTTGCTGTGATATCATGGCATGCCTG

TTTCGTGGTCCTTCTGAGAGCGACTTAGAACTGCTGGTCGGCCAGGCTATATTCGGGGATGGTGCGGCGGCAGTCATTGT

TGGCGCGGAACCCGATGAAAGCGTTGGCGAGCGCCCAATCTTCGAATTAGTAAGCACAGGTCAGACGATATTACCAAATA

GCGAAGGCACTATCGGGGGCCACATCCGCGAAGCTGGACTGATTTTTGACCTGCATAAAGATGTCCCGATGCTGATTAGT

AATAATATCGAGAAATGTCTGATTGAGGCATTTACCCCAATTGGAATTAGCGACTGGAACAGCATTTTCTGGATAACGCA

TCCGGGCGGCAAAGCTATCCTGGATAAAGTTGAGGAAAAGCTCCACCTGAAATCTGATAAGTTTGTAGATTCCCGCCACG

TGTTGAGCGAACATGGTAATATGAGCTCCTCAACAGTCCTATTCGTTATGGATGAACTGCGCAAACGCTCGCTCGAAGAA

GGTAAAAGTACCACCGGCGATGGCTTTGAATGGGGCGTGTTATTCGGTTTTGGCCCAGGCTTAACCGTTGAGCGTGTGGT

GGTACGATCGGTTCCTATAAAATATTGACTCTAGAAATAATTTTGTTTAACTTTAAGAAGGAGATATACATATGTCCCCT

ATACTAGGTTATTGGAAAATTAAGGGCCTTGTGCAACCCACTCGACTTCTTTTGGAATATCTTGAAGAAAAATATGAAGA

GCATTTGTATGAGCGCGATGAAGGTGATAAATGGCGAAACAAAAAGTTTGAATTGGGTTTGGAGTTTCCCAATCTTCCTT

ATTATATTGATGGTGATGTTAAATTAACACAGTCTATGGCCATCATACGTTATATAGCTGACAAGCACAACATGTTGGGT

GGTTGTCCAAAAGAGCGTGCAGAGATTTCAATGCTTGAAGGAGCGGTTTTGGATATTAGATACGGTGTTTCGAGAATTGC

ATATAGTAAAGACTTTGAAACTCTCAAAGTTGATTTTCTTAGCAAGCTACCTGAAATGCTGAAAATGTTCGAAGATCGTT

TATGTCATAAAACATATTTAAATGGTGATCATGTAACCCATCCTGACTTCATGTTGTATGACGCTCTTGATGTTGTTTTA

TACATGGACCCAATGTGCCTGGATGCGTTCCCAAAATTAGTTTGTTTTAAAAAACGTATTGAAGCTATCCCACAAATTGA

TAAGTACTTGAAATCCAGCAAGTATATAGCATGGCCTTTGCAGGGCTGGCAAGCCACGTTTGGTGGTGGCGACCATCCTC

CAAAATCGGATGGTTCAACTAGTGGTTCTGGTCATCACCATCACCATCACTCCATGAGCGATTACGACATCCCCACTACT

GAGAATCTTTATTTTCAGGGCGCCATGGCAGTAAAACATTTAATTGTGCTCAAATTTAAAGACGAAATTACTGAAGCACA

AAAAGAAGAGTTTTTCAAAACATACGTCAATCTGGTGAACATCATCCCGGCCATGAAAGACGTCTATTGGGGCAAAGACG

TAACGCAGAAAAACAAGGAAGAAGGTTATACCCACATCGTGGAGGTCACTTTCGAATCAGTCGAAACGATCCAGGATTAC

ATTATTCATCCGGCACATGTAGGGTTCGGCGATGTATATCGTAGCTTCTGGGAAAAACTCCTGATTTTTGACTATACCCC

GCGTAAATAAAGAAACTTGTTGACTTTTACGGCTAGCTCAGTCCTAGGTATTATGCTAGCCGCAGAGAATTCACGGAAGG

AGCATGGCGATGCTGCCTCCGAGCGATAGCAAAGATCCGCGTCCGTGGCAGATTCTGAGCCAGGCACTGGGTTTTCCGAA

TTATGATCAAGAACTGTGGTGGCAGAATACCGCAGAAACCCTGAATCGTGTTCTGGAACAGTGTGATTATAGCGTTCATC

TGCAGTATAAATACCTGGCCTTCTACCACAAATATATCCTGCCGAGCCTGGGTCCGTTTCGTCGTCCGGGTGTTGAACCG

GAATATATCAGCGGTCTGAGTCATGGTGGTCATCCGCTGGAAATTAGCGTGAAAATTGATAAAAGCAAGACCATTTGTCG

TCTGGGTCTGCAGGCAATTGGTCCGCTGGCAGGCACCGCACGTGATCCGCTGAATAGCTTTGGTGATCGTGAACTGCTGA

AAAATCTGGCAACCCTGCTGCCGCATGTTGATCTGCGTCTGTTTGATCATTTTAATGCACAGGTTGGTCTGGATCGTGCA

CAGTGTGCAGTTGCAACCACCAAACTGATTAAAGAAAGCCATAATATTGTGTGCACCAGCCTGGATCTGAAAGATGGTGA

AGTTATTCCGAAAGTGTACTTTAGCACCATTCCTAAAGGTCTGGTTACCGAAACACCGCTGTTCGATCTGACCTTTGCAG

CAATTGAGCAGATGGAAGTGTATCATAAAGATGCACCGCTGCGTACCGCACTGAGCAGCCTGAAAGATTTTCTGCGTCCG

CGTGTTCCGACCGATGCAAGCATTACCCCTCCGCTGACCGGTCTGATTGGTGTTGATTGTATTGATCCGATGCTGAGCCG

TCTGAAAGTTTATCTGGCCACCTTTCGTATGGATCTGAGCCTGATTCGTGATTATTGGACCTTAGGTGGTCTGCTGACCG

ATGCCGGTACAATGAAAGGCCTGGAAATGGTTGAAACCCTGGCAAAAACACTGAAACTGGGTGATGAAGCATGTGAAACA

CTGGATGCAGAACGTCTGCCGTTTGGTATTAACTATGCAATGAAACCGGGTACAGCAGAACTGGCACCGCCTCAGATCTA

TTTTCCGCTGCTGGGTATTAATGATGGCTTTATTGCAGATGCCCTGGTGGAATTTTTTCAGTATATGGGTTGGGAAGATC

AGGCCAATCGTTATAAAGATGAACTGAAAGCCAAATTTCCGAACGTGGATATTAGCCAGACCAAAAATGTTCATCGTTGG

CTGGGTGTTGCATATAGCGAAACCAAAGGTCCGAGCATGAACATCTATTATGATGTTGTTGCAGGTAATGTGGCACGCGT

TTAAACGATCTTAAGTAGGCGCGGAAAATAATGGAGTTCGACTTCAACAAATACATGGACAGCAAAGCCATGACCGTTAA

TGAAGCACTGAATAAAGCAATTCCGCTGCGTTATCCGCAGAAAATCTATGAAAGCATGCGTTATAGCCTGCTGGCAGGCG

GTAAACGTGTTCGTCCGGTTCTGTGTATTGCAGCATGTGAACTGGTTGGTGGCACCGAAGAACTGGCAATTCCGACCGCA

TGTGCAATTGAAATGATTCATACCATGAGCCTGATGCATGATGATCTGCCGTGTATTGATAATGATGACCTGCGTCGTGG

TAAACCGACCAATCATAAAATCTTTGGTGAAGATACCGCAGTGACCGCAGGTAATGCACTGCATAGTTATGCATTTGAAC

ATATTGCAGTGAGCACCAGCAAAACCGTTGGTGCAGATCGTATTCTGCGTATGGTTAGCGAACTGGGTCGTGCAACCGGT

AGCGAAGGTGTTATGGGTGGTCAGATGGTTGATATTGCAAGTGAAGGTGATCCGAGCATTGATCTGCAGACCCTGGAATG

GATTCATATTCATAAAACCGCAATGCTGCTGGAATGTAGCGTTGTTTGTGGTGCAATTATTGGTGGTGCAAGCGAAATTG

TTATTGAACGTGCCCGTCGTTATGCACGTTGTGTTGGTCTGCTGTTTCAGGTTGTTGATGATATTCTGGATGTGACCAAA

AGCAGTGATGAACTGGGCAAAACCGCAGGCAAAGATCTGATTAGCGATAAAGCAACCTATCCGAAACTGATGGGTCTGGA

AAAAGCCAAAGAATTTTCAGATGAACTGCTGAATCGTGCCAAAGGTGAACTGAGCTGTTTTGATCCGGTTAAAGCAGCAC

CGCTGCTGGGTCTGGCAGATTATGTTGCATTTCGTCAGAATTAAGGGATCCAAACTCGAGTAAGGATCTCCAGGCATCAA

ATAAAACGAAAGGCTCAGTCGAAAGACTGGGCCTTTCGTTTTATCTGTTGTTTGTCGGTGAACGCTCTCTACTAGAGTCA

CACTGGCTCACCTTCGGGTGGGCCTTTCTGCGTTTATACCTAGGCTACAGCCGATAGTCTGGAACAGCGCACTTACGGGT

TGCTGCGCAACCCAAGTGCTACCGGCGCGGCAGCGTGACCCGTGTCGGCGGCTCCAACGGCTCGCCATCGTCCAGAAAAC

ACGGCTCATCGGGCATCGGCAGGCGCTGCTGCCCGCGCCGTTCCCATTCCTCCGTTTCGGTCAAGGCTGGCAGGTCTGGT

TCCATGCCCGGAATGCCGGGCTGGCTGGGCGGCTCCTCGCCGGGGCCGGTCGGTAGTTGCTGCTCGCCCGGATACAGGGT

CGGGATGCGGCGCAGGTCGCCATGCCCCAACAGCGATTCGTCCTGGTCGTCGTGATCAACCACCACGGCGGCACTGAACA

CCGACAGGCGCAACTGGTCGCGGGGCTGGCCCCACGCCACGCGGTCATTGACCACGTAGGCCAACACGGTGCCGGGGCCG

TTGAGCTTCACGACGGAGATCCAGCGCTCGGCCACCAAGTCCTTGACTGCGTATTGGACCGTCCGCAAAGAACGTCCGAT

GAGCTTGGAAAGTGTCTTCTGGCTGACCACCACGGCGTTCTGGTGGCCCATCTGCGCCACGAGGTGATGCAGCAGCATTG

CCGCCGTGGGTTTCCTCGCAATAAGCCCGGCCCACGCCTCATGCGCTTTGCGTTCCGTTTGCACCCAGTGACCGGGCTTG

TTCTTGGCTTGAATGCCGATTTCTCTGGACTGCGTGGCCATGCTTATCTCCATGCGGTAGGGGTGCCGCACGGTTGCGGC

ACCATGCGCAATCAGCTGCAACTTTTCGGCAGCGCGACAACAATTATGCGTTGCGTAAAAGTGGCAGTCAATTACAGATT

TTCTTTAACCTACGCAATGAGCTATTGCGGGGGGTGCCGCAATGAGCTGTTGCGTACCCCCCTTTTTTAAGTTGTTGATT

TTTAAGTCTTTCGCATTTCGCCCTATATCTAGTTCTTTGGTGCCCAAAGAAGGGCACCCCTGCGGGGTTCCCCCACGCCT

TCGGCGCGGCTCCCCCTCCGGCAAAAAGTGGCCCCTCCGGGGCTTGTTGATCGACTGCGCGGCCTTCGGCCTTGCCCAAG

GTGGCGCTGCCCCCTTGGAACCCCCGCACTCGCCGCCGTGAGGCTCGGGGGGCAGGCGGGCGGGCTTCGCCCTTCGACTG

CCCCCACTCGCATAGGCTTGGGTCGTTCCAGGCGCGTCAAGGCCAAGCCGCTGCGCGGTCGCTGCGCGAGCCTTGACCCG

CCTTCCACTTGGTGTCCAACCGGCAAGCGAAGCGCGCAGGCCGCAGGCCGGAGGCACTAGTGCTTGGATTCTCACCAATA

AAAAACGCCCGGCGGCAACCGAGCGTTCTGAACAAATCCAGATGGAGTTCTGAGGTCATTACTGGATCTATCAACAGGAG

TCCAAGCGAGCTCGATATCAAATTACGCCCCGCCCTGCCACTCATCGCAGTACTGTTGTAATTCATTAAGCATTCTGCCG

ACATGGAAGCCATCACAAACGGCATGATGAACCTGAATCGCCAGCGGCATCAGCACCTTGTCGCCTTGCGTATAATATTT

GCCCATGGTGAAAACGGGGGCGAAGAAGTTGTCCATATTGGCCACGTTTAAATCAAAACTGGTGAAACTCACCCAGGGAT

TGGCTGAGACGAAAAACATATTCTCAATAAACCCTTTAGGGAAATAGGCCAGGTTTTCACCGTAACACGCCACATCTTGC

GAATATATGTGTAGAAACTGCCGGAAATCGTCGTGGTATTCACTCCAGAGCGATGAAAACGTTTCAGTTTGCTCATGGAA

AACGGTGTAACAAGGGTGAACACTATCCCATATCACCAGCTCACCGTCTTTCATTGCCATACGAAATTCCGGATGAGCAT

TCATCAGGCGGGCAAGAATGTGAATAAAGGCCGGATAAAACTTGTGCTTATTTTTCTTTACGGTCTTTAAAAAGGCCGTA

ATATCCAGCTGAACGGTCTGGTTATAGGTACATTGAGCAACTGACTGAAATGCCTCAAAATGTTCTTTACGATGCCATTG

GGATATATCAACGGTGGTATATCCAGTGATTTTTTTCTCCATTTTAGCTTCCTTAGCTCCTGAAAATCTCGATAACTCAA

AAAATACGCCCGGTAGTGATCTTATTTCATTATGGTGAAAGTTGGAACCTCTTACGTGCCGATCAACGTCTCATTTTCGC

CAGATATC

>pBbB2c-CBG (Genbank: OQ725949)

GACGTCTTAAGACCCACTTTCACATTTAAGTTGTTTTTCTAATCCGCATATGATCAATTCAAGGCCGAATAAGAAGGCTG

GCTCTGCACCTTGGTGATCAAATAATTCGATAGCTTGTCGTAATAATGGCGGCATACTATCAGTAGTAGGTGTTTCCCTT

TCTTCTTTAGCGACTTGATGCTCTTGATCTTCCAATACGCAACCTAAAGTAAAATGCCCCACAGCGCTGAGTGCATATAA

TGCATTCTCTAGTGAAAAACCTTGTTGGCATAAAAAGGCTAATTGATTTTCGAGAGTTTCATACTGTTTTTCTGTAGGCC

GTGTACCTAAATGTACTTTTGCTCCATCGCGATGACTTAGTAAAGCACATCTAAAACTTTTAGCGTTATTACGTAAAAAA

TCTTGCCAGCTTTCCCCTTCTAAAGGGCAAAAGTGAGTATGGTGCCTATCTAACATCTCAATGGCTAAGGCGTCGAGCAA

AGCCCGCTTATTTTTTACATGCCAATACAATGTAGGCTGCTCTACACCTAGCTTCTGGGCGAGTTTACGGGTTGTTAAAC

CTTCGATTCCGACCTCATTAAGCAGCTCTAATGCGCTGTTAATCACTTTACTTTTATCTAATCTAGACATCATTAATTCC

TAATTTTTGTTGACACTCTATCGTTGATAGAGTTATTTTACCACTCCCTATCAGTGATAGAGAAAAGAATTCAAAAGATC

TTTTAAGAAGCCTTAGGTCAATCCACAAAAGCGGCGCATAAGGAAAACTCCCTAGATTCGAGGACCAACAATGAACCACC

TAAGAGCAGAAGGCCCAGCCTCCGTGCTGGCCATTGGTACCGCCAATCCGGAAAACATTTTATTGCAGGACGAATTTCCT

GATTATTATTTTCGTGTTACCAAATCGGAACACATGACCCAGCTGAAAGAAAAATTTCGCAAAATTTGTGACAAAAGTAT

GATACGGAAACGAAATTGTTTTCTGAATGAGGAACACTTGAAGCAAAACCCTCGCTTAGTCGAACATGAGATGCAGACAC

TGGATGCCCGCCAGGACATGCTCGTGGTTGAAGTGCCGAAATTAGGTAAAGACGCGTGCGCGAAGGCGATTAAAGAGTGG

GGTCAGCCGAAATCCAAGATTACACACTTAATTTTTACCTCAGCGTCGACTACCGATATGCCTGGTGCCGACTATCATTG

TGCCAAGTTGTTGGGCTTAAGCCCCTCGGTTAAGCGTGTGATGATGTACCAACTGGGTTGTTATGGCGGTGGCACGGTGC

TGAGAATTGCGAAAGATATTGCCGAGAATAATAAAGGCGCGCGTGTTCTGGCCGTTTGCTGTGATATCATGGCATGCCTG

TTTCGTGGTCCTTCTGAGAGCGACTTAGAACTGCTGGTCGGCCAGGCTATATTCGGGGATGGTGCGGCGGCAGTCATTGT

TGGCGCGGAACCCGATGAAAGCGTTGGCGAGCGCCCAATCTTCGAATTAGTAAGCACAGGTCAGACGATATTACCAAATA

GCGAAGGCACTATCGGGGGCCACATCCGCGAAGCTGGACTGATTTTTGACCTGCATAAAGATGTCCCGATGCTGATTAGT

AATAATATCGAGAAATGTCTGATTGAGGCATTTACCCCAATTGGAATTAGCGACTGGAACAGCATTTTCTGGATAACGCA

TCCGGGCGGCAAAGCTATCCTGGATAAAGTTGAGGAAAAGCTCCACCTGAAATCTGATAAGTTTGTAGATTCCCGCCACG

TGTTGAGCGAACATGGTAATATGAGCTCCTCAACAGTCCTATTCGTTATGGATGAACTGCGCAAACGCTCGCTCGAAGAA

GGTAAAAGTACCACCGGCGATGGCTTTGAATGGGGCGTGTTATTCGGTTTTGGCCCAGGCTTAACCGTTGAGCGTGTGGT

GGTACGATCGGTTCCTATAAAATATTGACTCTAGAAATAATTTTGTTTAACTTTAAGAAGGAGATATACATATGCTGCCT

CCGAGCGATAGCAAAGATCCGCGTCCGTGGCAGATTCTGAGCCAGGCACTGGGTTTTCCGAATTATGATCAAGAACTGTG

GTGGCAGAATACCGCAGAAACCCTGAATCGTGTTCTGGAACAGTGTGATTATAGCGTTCATCTGCAGTATAAATACCTGG

CCTTCTACCACAAATATATCCTGCCGAGCCTGGGTCCGTTTCGTCGTCCGGGTGTTGAACCGGAATATATCAGCGGTCTG

AGTCATGGTGGTCATCCGCTGGAAATTAGCGTGAAAATTGATAAAAGCAAGACCATTTGTCGTCTGGGTCTGCAGGCAAT

TGGTCCGCTGGCAGGCACCGCACGTGATCCGCTGAATAGCTTTGGTGATCGTGAACTGCTGAAAAATCTGGCAACCCTGC

TGCCGCATGTTGATCTGCGTCTGTTTGATCATTTTAATGCACAGGTTGGTCTGGATCGTGCACAGTGTGCAGTTGCAACC

ACCAAACTGATTAAAGAAAGCCATAATATTGTGTGCACCAGCCTGGATCTGAAAGATGGTGAAGTTATTCCGAAAGTGTA

CTTTAGCACCATTCCTAAAGGTCTGGTTACCGAAACACCGCTGTTCGATCTGACCTTTGCAGCAATTGAGCAGATGGAAG

TGTATCATAAAGATGCACCGCTGCGTACCGCACTGAGCAGCCTGAAAGATTTTCTGCGTCCGCGTGTTCCGACCGATGCA

AGCATTACCCCTCCGCTGACCGGTCTGATTGGTGTTGATTGTATTGATCCGATGCTGAGCCGTCTGAAAGTTTATCTGGC

CACCTTTCGTATGGATCTGAGCCTGATTCGTGATTATTGGACCTTAGGTGGTCTGCTGACCGATGCCGGTACAATGAAAG

GCCTGGAAATGGTTGAAACCCTGGCAAAAACACTGAAACTGGGTGATGAAGCATGTGAAACACTGGATGCAGAACGTCTG

CCGTTTGGTATTAACTATGCAATGAAACCGGGTACAGCAGAACTGGCACCGCCTCAGATCTATTTTCCGCTGCTGGGTAT

TAATGATGGCTTTATTGCAGATGCCCTGGTGGAATTTTTTCAGTATATGGGTTGGGAAGATCAGGCCAATCGTTATAAAG

ATGAACTGAAAGCCAAATTTCCGAACGTGGATATTAGCCAGACCAAAAATGTTCATCGTTGGCTGGGTGTTGCATATAGC

GAAACCAAAGGTCCGAGCATGAACATCTATTATGATGTTGTTGCAGGTAATGTGGCACGCGTTTAAAGAAAAGAATTCAC

GATCTTAAGTAGGCGCGGAAAATAATGGAGTTCGACTTCAACAAATACATGGACAGCAAAGCCATGACCGTTAATGAAGC

ACTGAATAAAGCAATTCCGCTGCGTTATCCGCAGAAAATCTATGAAAGCATGCGTTATAGCCTGCTGGCAGGCGGTAAAC

GTGTTCGTCCGGTTCTGTGTATTGCAGCATGTGAACTGGTTGGTGGCACCGAAGAACTGGCAATTCCGACCGCATGTGCA

ATTGAAATGATTCATACCATGAGCCTGATGCATGATGATCTGCCGTGTATTGATAATGATGACCTGCGTCGTGGTAAACC

GACCAATCATAAAATCTTTGGTGAAGATACCGCAGTGACCGCAGGTAATGCACTGCATAGTTATGCATTTGAACATATTG

CAGTGAGCACCAGCAAAACCGTTGGTGCAGATCGTATTCTGCGTATGGTTAGCGAACTGGGTCGTGCAACCGGTAGCGAA

GGTGTTATGGGTGGTCAGATGGTTGATATTGCAAGTGAAGGTGATCCGAGCATTGATCTGCAGACCCTGGAATGGATTCA

TATTCATAAAACCGCAATGCTGCTGGAATGTAGCGTTGTTTGTGGTGCAATTATTGGTGGTGCAAGCGAAATTGTTATTG

AACGTGCCCGTCGTTATGCACGTTGTGTTGGTCTGCTGTTTCAGGTTGTTGATGATATTCTGGATGTGACCAAAAGCAGT

GATGAACTGGGCAAAACCGCAGGCAAAGATCTGATTAGCGATAAAGCAACCTATCCGAAACTGATGGGTCTGGAAAAAGC

CAAAGAATTTTCAGATGAACTGCTGAATCGTGCCAAAGGTGAACTGAGCTGTTTTGATCCGGTTAAAGCAGCACCGCTGC

TGGGTCTGGCAGATTATGTTGCATTTCGTCAGAATTAAGGGATCCAAACTCGAGTAAGGATCTCCAGGCATCAAATAAAA

CGAAAGGCTCAGTCGAAAGACTGGGCCTTTCGTTTTATCTGTTGTTTGTCGGTGAACGCTCTCTACTAGAGTCACACTGG

CTCACCTTCGGGTGGGCCTTTCTGCGTTTATACCTAGGCTACAGCCGATAGTCTGGAACAGCGCACTTACGGGTTGCTGC

GCAACCCAAGTGCTACCGGCGCGGCAGCGTGACCCGTGTCGGCGGCTCCAACGGCTCGCCATCGTCCAGAAAACACGGCT

CATCGGGCATCGGCAGGCGCTGCTGCCCGCGCCGTTCCCATTCCTCCGTTTCGGTCAAGGCTGGCAGGTCTGGTTCCATG

CCCGGAATGCCGGGCTGGCTGGGCGGCTCCTCGCCGGGGCCGGTCGGTAGTTGCTGCTCGCCCGGATACAGGGTCGGGAT

GCGGCGCAGGTCGCCATGCCCCAACAGCGATTCGTCCTGGTCGTCGTGATCAACCACCACGGCGGCACTGAACACCGACA

GGCGCAACTGGTCGCGGGGCTGGCCCCACGCCACGCGGTCATTGACCACGTAGGCCAACACGGTGCCGGGGCCGTTGAGC

TTCACGACGGAGATCCAGCGCTCGGCCACCAAGTCCTTGACTGCGTATTGGACCGTCCGCAAAGAACGTCCGATGAGCTT

GGAAAGTGTCTTCTGGCTGACCACCACGGCGTTCTGGTGGCCCATCTGCGCCACGAGGTGATGCAGCAGCATTGCCGCCG

TGGGTTTCCTCGCAATAAGCCCGGCCCACGCCTCATGCGCTTTGCGTTCCGTTTGCACCCAGTGACCGGGCTTGTTCTTG

GCTTGAATGCCGATTTCTCTGGACTGCGTGGCCATGCTTATCTCCATGCGGTAGGGGTGCCGCACGGTTGCGGCACCATG

CGCAATCAGCTGCAACTTTTCGGCAGCGCGACAACAATTATGCGTTGCGTAAAAGTGGCAGTCAATTACAGATTTTCTTT

AACCTACGCAATGAGCTATTGCGGGGGGTGCCGCAATGAGCTGTTGCGTACCCCCCTTTTTTAAGTTGTTGATTTTTAAG

TCTTTCGCATTTCGCCCTATATCTAGTTCTTTGGTGCCCAAAGAAGGGCACCCCTGCGGGGTTCCCCCACGCCTTCGGCG

CGGCTCCCCCTCCGGCAAAAAGTGGCCCCTCCGGGGCTTGTTGATCGACTGCGCGGCCTTCGGCCTTGCCCAAGGTGGCG

CTGCCCCCTTGGAACCCCCGCACTCGCCGCCGTGAGGCTCGGGGGGCAGGCGGGCGGGCTTCGCCCTTCGACTGCCCCCA

CTCGCATAGGCTTGGGTCGTTCCAGGCGCGTCAAGGCCAAGCCGCTGCGCGGTCGCTGCGCGAGCCTTGACCCGCCTTCC

ACTTGGTGTCCAACCGGCAAGCGAAGCGCGCAGGCCGCAGGCCGGAGGCACTAGTGCTTGGATTCTCACCAATAAAAAAC

GCCCGGCGGCAACCGAGCGTTCTGAACAAATCCAGATGGAGTTCTGAGGTCATTACTGGATCTATCAACAGGAGTCCAAG

CGAGCTCGATATCAAATTACGCCCCGCCCTGCCACTCATCGCAGTACTGTTGTAATTCATTAAGCATTCTGCCGACATGG

AAGCCATCACAAACGGCATGATGAACCTGAATCGCCAGCGGCATCAGCACCTTGTCGCCTTGCGTATAATATTTGCCCAT

GGTGAAAACGGGGGCGAAGAAGTTGTCCATATTGGCCACGTTTAAATCAAAACTGGTGAAACTCACCCAGGGATTGGCTG

AGACGAAAAACATATTCTCAATAAACCCTTTAGGGAAATAGGCCAGGTTTTCACCGTAACACGCCACATCTTGCGAATAT

ATGTGTAGAAACTGCCGGAAATCGTCGTGGTATTCACTCCAGAGCGATGAAAACGTTTCAGTTTGCTCATGGAAAACGGT

GTAACAAGGGTGAACACTATCCCATATCACCAGCTCACCGTCTTTCATTGCCATACGAAATTCCGGATGAGCATTCATCA

GGCGGGCAAGAATGTGAATAAAGGCCGGATAAAACTTGTGCTTATTTTTCTTTACGGTCTTTAAAAAGGCCGTAATATCC

AGCTGAACGGTCTGGTTATAGGTACATTGAGCAACTGACTGAAATGCCTCAAAATGTTCTTTACGATGCCATTGGGATAT

ATCAACGGTGGTATATCCAGTGATTTTTTTCTCCATTTTAGCTTCCTTAGCTCCTGAAAATCTCGATAACTCAAAAAATA

CGCCCGGTAGTGATCTTATTTCATTATGGTGAAAGTTGGAACCTCTTACGTGCCGATCAACGTCTCATTTTCGCCAGATA

TC

>pBbB1c-CBGA (Genbank: OQ725950)

GACGTCGACACCATCGAATGGTGCAAAACCTTTCGCGGTATGGCATGATAGCGCCCGGAAGAGAGTCAATTCAGGGTGGT

GAATGTGAAACCAGTAACGTTATACGATGTCGCAGAGTATGCCGGTGTCTCTTATCAGACCGTTTCCCGCGTGGTGAACC

AGGCCAGCCACGTTTCTGCGAAAACGCGGGAAAAAGTGGAAGCGGCGATGGCGGAGCTGAATTACATTCCCAACCGCGTG

GCACAACAACTGGCGGGCAAACAGTCGTTGCTGATTGGCGTTGCCACCTCCAGTCTGGCCCTGCACGCGCCGTCGCAAAT

TGTCGCGGCGATTAAATCTCGCGCCGATCAACTGGGTGCCAGCGTGGTGGTGTCGATGGTAGAACGAAGCGGCGTCGAAG

CCTGTAAAGCGGCGGTGCACAATCTTCTCGCGCAACGCGTCAGTGGGCTGATCATTAACTATCCGCTGGATGACCAGGAT

GCCATTGCTGTGGAAGCTGCCTGCACTAATGTTCCGGCGTTATTTCTTGATGTCTCTGACCAGACACCCATCAACAGTAT

TATTTTCTCCCATGAAGACGGTACGCGACTGGGCGTGGAGCATCTGGTCGCATTGGGTCACCAGCAAATCGCGCTGTTAG

CGGGCCCATTAAGTTCTGTCTCGGCGCGTCTGCGTCTGGCTGGCTGGCATAAATATCTCACTCGCAATCAAATTCAGCCG

ATAGCGGAACGGGAAGGCGACTGGAGTGCCATGTCCGGTTTTCAACAAACCATGCAAATGCTGAATGAGGGCATCGTTCC

CACTGCGATGCTGGTTGCCAACGATCAGATGGCGCTGGGCGCAATGCGCGCCATTACCGAGTCCGGGCTGCGCGTTGGTG

CGGATATCTCGGTAGTGGGATACGACGATACCGAAGACAGCTCATGTTATATCCCGCCGTTAACCACCATCAAACAGGAT

TTTCGCCTGCTGGGGCAAACCAGCGTGGACCGCTTGCTGCAACTCTCTCAGGGCCAGGCGGTGAAGGGCAATCAGCTGTT

GCCCGTCTCACTGGTGAAAAGAAAAACCACCCTGGCGCCCAATACGCAAACCGCCTCTCCCCGCGCGTTGGCCGATTCAT

TAATGCAGCTGGCACGACAGGTTTCCCGACTGGAAAGCGGGCAGTGAGCGCAACGCAATTAATGTAAGTTAGCGCGAATT

GATCTGGTTTGACAGCTTATCATCGACTGCACGGTGCACCAATGCTTCTGGCGTCAGGCAGCCATCGGAAGCTGTGGTAT

GGCTGTGCAGGTCGTAAATCACTGCATAATTCGTGTCGCTCAAGGCGCACTCCCGTTCTGGATAATGTTTTTTGCGCCGA

CATCATAACGGTTCTGGCAAATATTCTGAAATGAGCTGTTGACAATTAATCATCCGGCTCGTATAATGTGTGGAATTGTG

AGCGGATAACAATTTCAGAATTCAAAAGATCTTTTAAGAAGGAGATATACATATGAACCACCTAAGAGCAGAAGGCCCAG

CCTCCGTGCTGGCCATTGGTACCGCCAATCCGGAAAACATTTTATTGCAGGACGAATTTCCTGATTATTATTTTCGTGTT

ACCAAATCGGAACACATGACCCAGCTGAAAGAAAAATTTCGCAAAATTTGTGACAAAAGTATGATACGGAAACGAAATTG

TTTTCTGAATGAGGAACACTTGAAGCAAAACCCTCGCTTAGTCGAACATGAGATGCAGACACTGGATGCCCGCCAGGACA

TGCTCGTGGTTGAAGTGCCGAAATTAGGTAAAGACGCGTGCGCGAAGGCGATTAAAGAGTGGGGTCAGCCGAAATCCAAG

ATTACACACTTAATTTTTACCTCAGCGTCGACTACCGATATGCCTGGTGCCGACTATCATTGTGCCAAGTTGTTGGGCTT

AAGCCCCTCGGTTAAGCGTGTGATGATGTACCAACTGGGTTGTTATGGCGGTGGCACGGTGCTGAGAATTGCGAAAGATA

TTGCCGAGAATAATAAAGGCGCGCGTGTTCTGGCCGTTTGCTGTGATATCATGGCATGCCTGTTTCGTGGTCCTTCTGAG

AGCGACTTAGAACTGCTGGTCGGCCAGGCTATATTCGGGGATGGTGCGGCGGCAGTCATTGTTGGCGCGGAACCCGATGA

AAGCGTTGGCGAGCGCCCAATCTTCGAATTAGTAAGCACAGGTCAGACGATATTACCAAATAGCGAAGGCACTATCGGGG

GCCACATCCGCGAAGCTGGACTGATTTTTGACCTGCATAAAGATGTCCCGATGCTGATTAGTAATAATATCGAGAAATGT

CTGATTGAGGCATTTACCCCAATTGGAATTAGCGACTGGAACAGCATTTTCTGGATAACGCATCCGGGCGGCAAAGCTAT

CCTGGATAAAGTTGAGGAAAAGCTCCACCTGAAATCTGATAAGTTTGTAGATTCCCGCCACGTGTTGAGCGAACATGGTA

ATATGAGCTCCTCAACAGTCCTATTCGTTATGGATGAACTGCGCAAACGCTCGCTCGAAGAAGGTAAAAGTACCACCGGC

GATGGCTTTGAATGGGGCGTGTTATTCGGTTTTGGCCCAGGCTTAACCGTTGAGCGTGTGGTGGTACGATCGGTTCCTAT

AAAATATTGACTCTAGAAATAATTTTGTTTAACTTTAAGAAGGAGATATACATATGTCCCCTATACTAGGTTATTGGAAA

ATTAAGGGCCTTGTGCAACCCACTCGACTTCTTTTGGAATATCTTGAAGAAAAATATGAAGAGCATTTGTATGAGCGCGA

TGAAGGTGATAAATGGCGAAACAAAAAGTTTGAATTGGGTTTGGAGTTTCCCAATCTTCCTTATTATATTGATGGTGATG

TTAAATTAACACAGTCTATGGCCATCATACGTTATATAGCTGACAAGCACAACATGTTGGGTGGTTGTCCAAAAGAGCGT

GCAGAGATTTCAATGCTTGAAGGAGCGGTTTTGGATATTAGATACGGTGTTTCGAGAATTGCATATAGTAAAGACTTTGA

AACTCTCAAAGTTGATTTTCTTAGCAAGCTACCTGAAATGCTGAAAATGTTCGAAGATCGTTTATGTCATAAAACATATT

TAAATGGTGATCATGTAACCCATCCTGACTTCATGTTGTATGACGCTCTTGATGTTGTTTTATACATGGACCCAATGTGC

CTGGATGCGTTCCCAAAATTAGTTTGTTTTAAAAAACGTATTGAAGCTATCCCACAAATTGATAAGTACTTGAAATCCAG

CAAGTATATAGCATGGCCTTTGCAGGGCTGGCAAGCCACGTTTGGTGGTGGCGACCATCCTCCAAAATCGGATGGTTCAA

CTAGTGGTTCTGGTCATCACCATCACCATCACTCCATGAGCGATTACGACATCCCCACTACTGAGAATCTTTATTTTCAG

GGCGCCATGGCAGTAAAACATTTAATTGTGCTCAAATTTAAAGACGAAATTACTGAAGCACAAAAAGAAGAGTTTTTCAA

AACATACGTCAATCTGGTGAACATCATCCCGGCCATGAAAGACGTCTATTGGGGCAAAGACGTAACGCAGAAAAACAAGG

AAGAAGGTTATACCCACATCGTGGAGGTCACTTTCGAATCAGTCGAAACGATCCAGGATTACATTATTCATCCGGCACAT

GTAGGGTTCGGCGATGTATATCGTAGCTTCTGGGAAAAACTCCTGATTTTTGACTATACCCCGCGTAAATAAAGAAAAGA

ATTCACGGAAGGAGCATGGCGATGCTGCCTCCGAGCGATAGCAAAGATCCGCGTCCGTGGCAGATTCTGAGCCAGGCACT

GGGTTTTCCGAATTATGATCAAGAACTGTGGTGGCAGAATACCGCAGAAACCCTGAATCGTGTTCTGGAACAGTGTGATT

ATAGCGTTCATCTGCAGTATAAATACCTGGCCTTCTACCACAAATATATCCTGCCGAGCCTGGGTCCGTTTCGTCGTCCG

GGTGTTGAACCGGAATATATCAGCGGTCTGAGTCATGGTGGTCATCCGCTGGAAATTAGCGTGAAAATTGATAAAAGCAA

GACCATTTGTCGTCTGGGTCTGCAGGCAATTGGTCCGCTGGCAGGCACCGCACGTGATCCGCTGAATAGCTTTGGTGATC

GTGAACTGCTGAAAAATCTGGCAACCCTGCTGCCGCATGTTGATCTGCGTCTGTTTGATCATTTTAATGCACAGGTTGGT

CTGGATCGTGCACAGTGTGCAGTTGCAACCACCAAACTGATTAAAGAAAGCCATAATATTGTGTGCACCAGCCTGGATCT

GAAAGATGGTGAAGTTATTCCGAAAGTGTACTTTAGCACCATTCCTAAAGGTCTGGTTACCGAAACACCGCTGTTCGATC

TGACCTTTGCAGCAATTGAGCAGATGGAAGTGTATCATAAAGATGCACCGCTGCGTACCGCACTGAGCAGCCTGAAAGAT

TTTCTGCGTCCGCGTGTTCCGACCGATGCAAGCATTACCCCTCCGCTGACCGGTCTGATTGGTGTTGATTGTATTGATCC

GATGCTGAGCCGTCTGAAAGTTTATCTGGCCACCTTTCGTATGGATCTGAGCCTGATTCGTGATTATTGGACCTTAGGTG

GTCTGCTGACCGATGCCGGTACAATGAAAGGCCTGGAAATGGTTGAAACCCTGGCAAAAACACTGAAACTGGGTGATGAA

GCATGTGAAACACTGGATGCAGAACGTCTGCCGTTTGGTATTAACTATGCAATGAAACCGGGTACAGCAGAACTGGCACC

GCCTCAGATCTATTTTCCGCTGCTGGGTATTAATGATGGCTTTATTGCAGATGCCCTGGTGGAATTTTTTCAGTATATGG

GTTGGGAAGATCAGGCCAATCGTTATAAAGATGAACTGAAAGCCAAATTTCCGAACGTGGATATTAGCCAGACCAAAAAT

GTTCATCGTTGGCTGGGTGTTGCATATAGCGAAACCAAAGGTCCGAGCATGAACATCTATTATGATGTTGTTGCAGGTAA

TGTGGCACGCGTTTAAACGATCTTAAGTAGGCGCGGAAAATAATGGAGTTCGACTTCAACAAATACATGGACAGCAAAGC

CATGACCGTTAATGAAGCACTGAATAAAGCAATTCCGCTGCGTTATCCGCAGAAAATCTATGAAAGCATGCGTTATAGCC

TGCTGGCAGGCGGTAAACGTGTTCGTCCGGTTCTGTGTATTGCAGCATGTGAACTGGTTGGTGGCACCGAAGAACTGGCA

ATTCCGACCGCATGTGCAATTGAAATGATTCATACCATGAGCCTGATGCATGATGATCTGCCGTGTATTGATAATGATGA

CCTGCGTCGTGGTAAACCGACCAATCATAAAATCTTTGGTGAAGATACCGCAGTGACCGCAGGTAATGCACTGCATAGTT

ATGCATTTGAACATATTGCAGTGAGCACCAGCAAAACCGTTGGTGCAGATCGTATTCTGCGTATGGTTAGCGAACTGGGT

CGTGCAACCGGTAGCGAAGGTGTTATGGGTGGTCAGATGGTTGATATTGCAAGTGAAGGTGATCCGAGCATTGATCTGCA

GACCCTGGAATGGATTCATATTCATAAAACCGCAATGCTGCTGGAATGTAGCGTTGTTTGTGGTGCAATTATTGGTGGTG

CAAGCGAAATTGTTATTGAACGTGCCCGTCGTTATGCACGTTGTGTTGGTCTGCTGTTTCAGGTTGTTGATGATATTCTG

GATGTGACCAAAAGCAGTGATGAACTGGGCAAAACCGCAGGCAAAGATCTGATTAGCGATAAAGCAACCTATCCGAAACT

GATGGGTCTGGAAAAAGCCAAAGAATTTTCAGATGAACTGCTGAATCGTGCCAAAGGTGAACTGAGCTGTTTTGATCCGG

TTAAAGCAGCACCGCTGCTGGGTCTGGCAGATTATGTTGCATTTCGTCAGAATTAAGGGATCCAAACTCGAGTAAGGATC

TCCAGGCATCAAATAAAACGAAAGGCTCAGTCGAAAGACTGGGCCTTTCGTTTTATCTGTTGTTTGTCGGTGAACGCTCT

CTACTAGAGTCACACTGGCTCACCTTCGGGTGGGCCTTTCTGCGTTTATACCTAGGCTACAGCCGATAGTCTGGAACAGC

GCACTTACGGGTTGCTGCGCAACCCAAGTGCTACCGGCGCGGCAGCGTGACCCGTGTCGGCGGCTCCAACGGCTCGCCAT

CGTCCAGAAAACACGGCTCATCGGGCATCGGCAGGCGCTGCTGCCCGCGCCGTTCCCATTCCTCCGTTTCGGTCAAGGCT

GGCAGGTCTGGTTCCATGCCCGGAATGCCGGGCTGGCTGGGCGGCTCCTCGCCGGGGCCGGTCGGTAGTTGCTGCTCGCC

CGGATACAGGGTCGGGATGCGGCGCAGGTCGCCATGCCCCAACAGCGATTCGTCCTGGTCGTCGTGATCAACCACCACGG

CGGCACTGAACACCGACAGGCGCAACTGGTCGCGGGGCTGGCCCCACGCCACGCGGTCATTGACCACGTAGGCCAACACG

GTGCCGGGGCCGTTGAGCTTCACGACGGAGATCCAGCGCTCGGCCACCAAGTCCTTGACTGCGTATTGGACCGTCCGCAA

AGAACGTCCGATGAGCTTGGAAAGTGTCTTCTGGCTGACCACCACGGCGTTCTGGTGGCCCATCTGCGCCACGAGGTGAT

GCAGCAGCATTGCCGCCGTGGGTTTCCTCGCAATAAGCCCGGCCCACGCCTCATGCGCTTTGCGTTCCGTTTGCACCCAG

TGACCGGGCTTGTTCTTGGCTTGAATGCCGATTTCTCTGGACTGCGTGGCCATGCTTATCTCCATGCGGTAGGGGTGCCG

CACGGTTGCGGCACCATGCGCAATCAGCTGCAACTTTTCGGCAGCGCGACAACAATTATGCGTTGCGTAAAAGTGGCAGT

CAATTACAGATTTTCTTTAACCTACGCAATGAGCTATTGCGGGGGGTGCCGCAATGAGCTGTTGCGTACCCCCCTTTTTT

AAGTTGTTGATTTTTAAGTCTTTCGCATTTCGCCCTATATCTAGTTCTTTGGTGCCCAAAGAAGGGCACCCCTGCGGGGT

TCCCCCACGCCTTCGGCGCGGCTCCCCCTCCGGCAAAAAGTGGCCCCTCCGGGGCTTGTTGATCGACTGCGCGGCCTTCG

GCCTTGCCCAAGGTGGCGCTGCCCCCTTGGAACCCCCGCACTCGCCGCCGTGAGGCTCGGGGGGCAGGCGGGCGGGCTTC

GCCCTTCGACTGCCCCCACTCGCATAGGCTTGGGTCGTTCCAGGCGCGTCAAGGCCAAGCCGCTGCGCGGTCGCTGCGCG

AGCCTTGACCCGCCTTCCACTTGGTGTCCAACCGGCAAGCGAAGCGCGCAGGCCGCAGGCCGGAGGCACTAGTGCTTGGA

TTCTCACCAATAAAAAACGCCCGGCGGCAACCGAGCGTTCTGAACAAATCCAGATGGAGTTCTGAGGTCATTACTGGATC

TATCAACAGGAGTCCAAGCGAGCTCGATATCAAATTACGCCCCGCCCTGCCACTCATCGCAGTACTGTTGTAATTCATTA

AGCATTCTGCCGACATGGAAGCCATCACAAACGGCATGATGAACCTGAATCGCCAGCGGCATCAGCACCTTGTCGCCTTG

CGTATAATATTTGCCCATGGTGAAAACGGGGGCGAAGAAGTTGTCCATATTGGCCACGTTTAAATCAAAACTGGTGAAAC

TCACCCAGGGATTGGCTGAGACGAAAAACATATTCTCAATAAACCCTTTAGGGAAATAGGCCAGGTTTTCACCGTAACAC

GCCACATCTTGCGAATATATGTGTAGAAACTGCCGGAAATCGTCGTGGTATTCACTCCAGAGCGATGAAAACGTTTCAGT

TTGCTCATGGAAAACGGTGTAACAAGGGTGAACACTATCCCATATCACCAGCTCACCGTCTTTCATTGCCATACGAAATT

CCGGATGAGCATTCATCAGGCGGGCAAGAATGTGAATAAAGGCCGGATAAAACTTGTGCTTATTTTTCTTTACGGTCTTT

AAAAAGGCCGTAATATCCAGCTGAACGGTCTGGTTATAGGTACATTGAGCAACTGACTGAAATGCCTCAAAATGTTCTTT

ACGATGCCATTGGGATATATCAACGGTGGTATATCCAGTGATTTTTTTCTCCATTTTAGCTTCCTTAGCTCCTGAAAATC

TCGATAACTCAAAAAATACGCCCGGTAGTGATCTTATTTCATTATGGTGAAAGTTGGAACCTCTTACGTGCCGATCAACG

TCTCATTTTCGCCAGATATC

>pBbB1c-CBGA_Ptrc(8422 bp) (Genbank: OQ725951)

GACGTCGACACCATCGAATGGTGCAAAACCTTTCGCGGTATGGCATGATAGCGCCCGGAAGAGAGTCAATTCAGGGTGGT

GAATGTGAAACCAGTAACGTTATACGATGTCGCAGAGTATGCCGGTGTCTCTTATCAGACCGTTTCCCGCGTGGTGAACC

AGGCCAGCCACGTTTCTGCGAAAACGCGGGAAAAAGTGGAAGCGGCGATGGCGGAGCTGAATTACATTCCCAACCGCGTG

GCACAACAACTGGCGGGCAAACAGTCGTTGCTGATTGGCGTTGCCACCTCCAGTCTGGCCCTGCACGCGCCGTCGCAAAT

TGTCGCGGCGATTAAATCTCGCGCCGATCAACTGGGTGCCAGCGTGGTGGTGTCGATGGTAGAACGAAGCGGCGTCGAAG

CCTGTAAAGCGGCGGTGCACAATCTTCTCGCGCAACGCGTCAGTGGGCTGATCATTAACTATCCGCTGGATGACCAGGAT

GCCATTGCTGTGGAAGCTGCCTGCACTAATGTTCCGGCGTTATTTCTTGATGTCTCTGACCAGACACCCATCAACAGTAT

TATTTTCTCCCATGAAGACGGTACGCGACTGGGCGTGGAGCATCTGGTCGCATTGGGTCACCAGCAAATCGCGCTGTTAG

CGGGCCCATTAAGTTCTGTCTCGGCGCGTCTGCGTCTGGCTGGCTGGCATAAATATCTCACTCGCAATCAAATTCAGCCG

ATAGCGGAACGGGAAGGCGACTGGAGTGCCATGTCCGGTTTTCAACAAACCATGCAAATGCTGAATGAGGGCATCGTTCC

CACTGCGATGCTGGTTGCCAACGATCAGATGGCGCTGGGCGCAATGCGCGCCATTACCGAGTCCGGGCTGCGCGTTGGTG

CGGATATCTCGGTAGTGGGATACGACGATACCGAAGACAGCTCATGTTATATCCCGCCGTTAACCACCATCAAACAGGAT

TTTCGCCTGCTGGGGCAAACCAGCGTGGACCGCTTGCTGCAACTCTCTCAGGGCCAGGCGGTGAAGGGCAATCAGCTGTT

GCCCGTCTCACTGGTGAAAAGAAAAACCACCCTGGCGCCCAATACGCAAACCGCCTCTCCCCGCGCGTTGGCCGATTCAT

TAATGCAGCTGGCACGACAGGTTTCCCGACTGGAAAGCGGGCAGTGAGCGCAACGCAATTAATGTAAGTTAGCGCGAATT

GATCTGGTTTGACAGCTTATCATCGACTGCACGGTGCACCAATGCTTCTGGCGTCAGGCAGCCATCGGAAGCTGTGGTAT

GGCTGTGCAGGTCGTAAATCACTGCATAATTCGTGTCGCTCAAGGCGCACTCCCGTTCTGGATAATGTTTTTTGCGCCGA

CATCATAACGGTTCTGGCAAATATTCTGAAATGAGCTGTTGACAATTAATCATCCGGCTCGTATAATGTGTGGAATTGTG

AGCGGATAACAATTTCAGAATTCAAAAGATCTTTTAAGAAGGAGATATACATATGAACCACCTAAGAGCAGAAGGCCCAG

CCTCCGTGCTGGCCATTGGTACCGCCAATCCGGAAAACATTTTATTGCAGGACGAATTTCCTGATTATTATTTTCGTGTT

ACCAAATCGGAACACATGACCCAGCTGAAAGAAAAATTTCGCAAAATTTGTGACAAAAGTATGATACGGAAACGAAATTG

TTTTCTGAATGAGGAACACTTGAAGCAAAACCCTCGCTTAGTCGAACATGAGATGCAGACACTGGATGCCCGCCAGGACA

TGCTCGTGGTTGAAGTGCCGAAATTAGGTAAAGACGCGTGCGCGAAGGCGATTAAAGAGTGGGGTCAGCCGAAATCCAAG

ATTACACACTTAATTTTTACCTCAGCGTCGACTACCGATATGCCTGGTGCCGACTATCATTGTGCCAAGTTGTTGGGCTT

AAGCCCCTCGGTTAAGCGTGTGATGATGTACCAACTGGGTTGTTATGGCGGTGGCACGGTGCTGAGAATTGCGAAAGATA

TTGCCGAGAATAATAAAGGCGCGCGTGTTCTGGCCGTTTGCTGTGATATCATGGCATGCCTGTTTCGTGGTCCTTCTGAG

AGCGACTTAGAACTGCTGGTCGGCCAGGCTATATTCGGGGATGGTGCGGCGGCAGTCATTGTTGGCGCGGAACCCGATGA

AAGCGTTGGCGAGCGCCCAATCTTCGAATTAGTAAGCACAGGTCAGACGATATTACCAAATAGCGAAGGCACTATCGGGG

GCCACATCCGCGAAGCTGGACTGATTTTTGACCTGCATAAAGATGTCCCGATGCTGATTAGTAATAATATCGAGAAATGT

CTGATTGAGGCATTTACCCCAATTGGAATTAGCGACTGGAACAGCATTTTCTGGATAACGCATCCGGGCGGCAAAGCTAT

CCTGGATAAAGTTGAGGAAAAGCTCCACCTGAAATCTGATAAGTTTGTAGATTCCCGCCACGTGTTGAGCGAACATGGTA

ATATGAGCTCCTCAACAGTCCTATTCGTTATGGATGAACTGCGCAAACGCTCGCTCGAAGAAGGTAAAAGTACCACCGGC

GATGGCTTTGAATGGGGCGTGTTATTCGGTTTTGGCCCAGGCTTAACCGTTGAGCGTGTGGTGGTACGATCGGTTCCTAT

AAAATATTGACTCTAGAAATAATTTTGTTTAACTTTAAGAAGGAGATATACATATGTCCCCTATACTAGGTTATTGGAAA

ATTAAGGGCCTTGTGCAACCCACTCGACTTCTTTTGGAATATCTTGAAGAAAAATATGAAGAGCATTTGTATGAGCGCGA

TGAAGGTGATAAATGGCGAAACAAAAAGTTTGAATTGGGTTTGGAGTTTCCCAATCTTCCTTATTATATTGATGGTGATG

TTAAATTAACACAGTCTATGGCCATCATACGTTATATAGCTGACAAGCACAACATGTTGGGTGGTTGTCCAAAAGAGCGT

GCAGAGATTTCAATGCTTGAAGGAGCGGTTTTGGATATTAGATACGGTGTTTCGAGAATTGCATATAGTAAAGACTTTGA

AACTCTCAAAGTTGATTTTCTTAGCAAGCTACCTGAAATGCTGAAAATGTTCGAAGATCGTTTATGTCATAAAACATATT

TAAATGGTGATCATGTAACCCATCCTGACTTCATGTTGTATGACGCTCTTGATGTTGTTTTATACATGGACCCAATGTGC

CTGGATGCGTTCCCAAAATTAGTTTGTTTTAAAAAACGTATTGAAGCTATCCCACAAATTGATAAGTACTTGAAATCCAG

CAAGTATATAGCATGGCCTTTGCAGGGCTGGCAAGCCACGTTTGGTGGTGGCGACCATCCTCCAAAATCGGATGGTTCAA

CTAGTGGTTCTGGTCATCACCATCACCATCACTCCATGAGCGATTACGACATCCCCACTACTGAGAATCTTTATTTTCAG

GGCGCCATGGCAGTAAAACATTTAATTGTGCTCAAATTTAAAGACGAAATTACTGAAGCACAAAAAGAAGAGTTTTTCAA

AACATACGTCAATCTGGTGAACATCATCCCGGCCATGAAAGACGTCTATTGGGGCAAAGACGTAACGCAGAAAAACAAGG

AAGAAGGTTATACCCACATCGTGGAGGTCACTTTCGAATCAGTCGAAACGATCCAGGATTACATTATTCATCCGGCACAT

GTAGGGTTCGGCGATGTATATCGTAGCTTCTGGGAAAAACTCCTGATTTTTGACTATACCCCGCGTAAATAAAGCGCAGC

TTGTTGACTTTGACAATTAATCATCCGGCTCGTATAATGTGTGGAATTGTGAGCGGATAACAAGAAGCTGAGTTGGAAAA

GAATTCACGGAAGGAGCATGGCGATGCTGCCTCCGAGCGATAGCAAAGATCCGCGTCCGTGGCAGATTCTGAGCCAGGCA

CTGGGTTTTCCGAATTATGATCAAGAACTGTGGTGGCAGAATACCGCAGAAACCCTGAATCGTGTTCTGGAACAGTGTGA

TTATAGCGTTCATCTGCAGTATAAATACCTGGCCTTCTACCACAAATATATCCTGCCGAGCCTGGGTCCGTTTCGTCGTC

CGGGTGTTGAACCGGAATATATCAGCGGTCTGAGTCATGGTGGTCATCCGCTGGAAATTAGCGTGAAAATTGATAAAAGC

AAGACCATTTGTCGTCTGGGTCTGCAGGCAATTGGTCCGCTGGCAGGCACCGCACGTGATCCGCTGAATAGCTTTGGTGA

TCGTGAACTGCTGAAAAATCTGGCAACCCTGCTGCCGCATGTTGATCTGCGTCTGTTTGATCATTTTAATGCACAGGTTG

GTCTGGATCGTGCACAGTGTGCAGTTGCAACCACCAAACTGATTAAAGAAAGCCATAATATTGTGTGCACCAGCCTGGAT

CTGAAAGATGGTGAAGTTATTCCGAAAGTGTACTTTAGCACCATTCCTAAAGGTCTGGTTACCGAAACACCGCTGTTCGA

TCTGACCTTTGCAGCAATTGAGCAGATGGAAGTGTATCATAAAGATGCACCGCTGCGTACCGCACTGAGCAGCCTGAAAG

ATTTTCTGCGTCCGCGTGTTCCGACCGATGCAAGCATTACCCCTCCGCTGACCGGTCTGATTGGTGTTGATTGTATTGAT

CCGATGCTGAGCCGTCTGAAAGTTTATCTGGCCACCTTTCGTATGGATCTGAGCCTGATTCGTGATTATTGGACCTTAGG

TGGTCTGCTGACCGATGCCGGTACAATGAAAGGCCTGGAAATGGTTGAAACCCTGGCAAAAACACTGAAACTGGGTGATG

AAGCATGTGAAACACTGGATGCAGAACGTCTGCCGTTTGGTATTAACTATGCAATGAAACCGGGTACAGCAGAACTGGCA

CCGCCTCAGATCTATTTTCCGCTGCTGGGTATTAATGATGGCTTTATTGCAGATGCCCTGGTGGAATTTTTTCAGTATAT

GGGTTGGGAAGATCAGGCCAATCGTTATAAAGATGAACTGAAAGCCAAATTTCCGAACGTGGATATTAGCCAGACCAAAA

ATGTTCATCGTTGGCTGGGTGTTGCATATAGCGAAACCAAAGGTCCGAGCATGAACATCTATTATGATGTTGTTGCAGGT

AATGTGGCACGCGTTTAAACGATCTTAAGTAGGCGCGGAAAATAATGGAGTTCGACTTCAACAAATACATGGACAGCAAA

GCCATGACCGTTAATGAAGCACTGAATAAAGCAATTCCGCTGCGTTATCCGCAGAAAATCTATGAAAGCATGCGTTATAG

CCTGCTGGCAGGCGGTAAACGTGTTCGTCCGGTTCTGTGTATTGCAGCATGTGAACTGGTTGGTGGCACCGAAGAACTGG

CAATTCCGACCGCATGTGCAATTGAAATGATTCATACCATGAGCCTGATGCATGATGATCTGCCGTGTATTGATAATGAT

GACCTGCGTCGTGGTAAACCGACCAATCATAAAATCTTTGGTGAAGATACCGCAGTGACCGCAGGTAATGCACTGCATAG

TTATGCATTTGAACATATTGCAGTGAGCACCAGCAAAACCGTTGGTGCAGATCGTATTCTGCGTATGGTTAGCGAACTGG

GTCGTGCAACCGGTAGCGAAGGTGTTATGGGTGGTCAGATGGTTGATATTGCAAGTGAAGGTGATCCGAGCATTGATCTG

CAGACCCTGGAATGGATTCATATTCATAAAACCGCAATGCTGCTGGAATGTAGCGTTGTTTGTGGTGCAATTATTGGTGG

TGCAAGCGAAATTGTTATTGAACGTGCCCGTCGTTATGCACGTTGTGTTGGTCTGCTGTTTCAGGTTGTTGATGATATTC

TGGATGTGACCAAAAGCAGTGATGAACTGGGCAAAACCGCAGGCAAAGATCTGATTAGCGATAAAGCAACCTATCCGAAA

CTGATGGGTCTGGAAAAAGCCAAAGAATTTTCAGATGAACTGCTGAATCGTGCCAAAGGTGAACTGAGCTGTTTTGATCC

GGTTAAAGCAGCACCGCTGCTGGGTCTGGCAGATTATGTTGCATTTCGTCAGAATTAAGGGATCCAAACTCGAGTAAGGA

TCTCCAGGCATCAAATAAAACGAAAGGCTCAGTCGAAAGACTGGGCCTTTCGTTTTATCTGTTGTTTGTCGGTGAACGCT

CTCTACTAGAGTCACACTGGCTCACCTTCGGGTGGGCCTTTCTGCGTTTATACCTAGGCTACAGCCGATAGTCTGGAACA

GCGCACTTACGGGTTGCTGCGCAACCCAAGTGCTACCGGCGCGGCAGCGTGACCCGTGTCGGCGGCTCCAACGGCTCGCC

ATCGTCCAGAAAACACGGCTCATCGGGCATCGGCAGGCGCTGCTGCCCGCGCCGTTCCCATTCCTCCGTTTCGGTCAAGG

CTGGCAGGTCTGGTTCCATGCCCGGAATGCCGGGCTGGCTGGGCGGCTCCTCGCCGGGGCCGGTCGGTAGTTGCTGCTCG

CCCGGATACAGGGTCGGGATGCGGCGCAGGTCGCCATGCCCCAACAGCGATTCGTCCTGGTCGTCGTGATCAACCACCAC

GGCGGCACTGAACACCGACAGGCGCAACTGGTCGCGGGGCTGGCCCCACGCCACGCGGTCATTGACCACGTAGGCCAACA

CGGTGCCGGGGCCGTTGAGCTTCACGACGGAGATCCAGCGCTCGGCCACCAAGTCCTTGACTGCGTATTGGACCGTCCGC

AAAGAACGTCCGATGAGCTTGGAAAGTGTCTTCTGGCTGACCACCACGGCGTTCTGGTGGCCCATCTGCGCCACGAGGTG

ATGCAGCAGCATTGCCGCCGTGGGTTTCCTCGCAATAAGCCCGGCCCACGCCTCATGCGCTTTGCGTTCCGTTTGCACCC

AGTGACCGGGCTTGTTCTTGGCTTGAATGCCGATTTCTCTGGACTGCGTGGCCATGCTTATCTCCATGCGGTAGGGGTGC

CGCACGGTTGCGGCACCATGCGCAATCAGCTGCAACTTTTCGGCAGCGCGACAACAATTATGCGTTGCGTAAAAGTGGCA

GTCAATTACAGATTTTCTTTAACCTACGCAATGAGCTATTGCGGGGGGTGCCGCAATGAGCTGTTGCGTACCCCCCTTTT

TTAAGTTGTTGATTTTTAAGTCTTTCGCATTTCGCCCTATATCTAGTTCTTTGGTGCCCAAAGAAGGGCACCCCTGCGGG

GTTCCCCCACGCCTTCGGCGCGGCTCCCCCTCCGGCAAAAAGTGGCCCCTCCGGGGCTTGTTGATCGACTGCGCGGCCTT

CGGCCTTGCCCAAGGTGGCGCTGCCCCCTTGGAACCCCCGCACTCGCCGCCGTGAGGCTCGGGGGGCAGGCGGGCGGGCT

TCGCCCTTCGACTGCCCCCACTCGCATAGGCTTGGGTCGTTCCAGGCGCGTCAAGGCCAAGCCGCTGCGCGGTCGCTGCG

CGAGCCTTGACCCGCCTTCCACTTGGTGTCCAACCGGCAAGCGAAGCGCGCAGGCCGCAGGCCGGAGGCACTAGTGCTTG

GATTCTCACCAATAAAAAACGCCCGGCGGCAACCGAGCGTTCTGAACAAATCCAGATGGAGTTCTGAGGTCATTACTGGA

TCTATCAACAGGAGTCCAAGCGAGCTCGATATCAAATTACGCCCCGCCCTGCCACTCATCGCAGTACTGTTGTAATTCAT

TAAGCATTCTGCCGACATGGAAGCCATCACAAACGGCATGATGAACCTGAATCGCCAGCGGCATCAGCACCTTGTCGCCT

TGCGTATAATATTTGCCCATGGTGAAAACGGGGGCGAAGAAGTTGTCCATATTGGCCACGTTTAAATCAAAACTGGTGAA

ACTCACCCAGGGATTGGCTGAGACGAAAAACATATTCTCAATAAACCCTTTAGGGAAATAGGCCAGGTTTTCACCGTAAC

ACGCCACATCTTGCGAATATATGTGTAGAAACTGCCGGAAATCGTCGTGGTATTCACTCCAGAGCGATGAAAACGTTTCA

GTTTGCTCATGGAAAACGGTGTAACAAGGGTGAACACTATCCCATATCACCAGCTCACCGTCTTTCATTGCCATACGAAA

TTCCGGATGAGCATTCATCAGGCGGGCAAGAATGTGAATAAAGGCCGGATAAAACTTGTGCTTATTTTTCTTTACGGTCT

TTAAAAAGGCCGTAATATCCAGCTGAACGGTCTGGTTATAGGTACATTGAGCAACTGACTGAAATGCCTCAAAATGTTCT

TTACGATGCCATTGGGATATATCAACGGTGGTATATCCAGTGATTTTTTTCTCCATTTTAGCTTCCTTAGCTCCTGAAAA

TCTCGATAACTCAAAAAATACGCCCGGTAGTGATCTTATTTCATTATGGTGAAAGTTGGAACCTCTTACGTGCCGATCAA

CGTCTCATTTTCGCCAGATATC

>pBbB1c-CBGA_PlacUV5 (Genbank: OQ725952)

GACGTCGACACCATCGAATGGTGCAAAACCTTTCGCGGTATGGCATGATAGCGCCCGGAAGAGAGTCAATTCAGGGTGGT

GAATGTGAAACCAGTAACGTTATACGATGTCGCAGAGTATGCCGGTGTCTCTTATCAGACCGTTTCCCGCGTGGTGAACC

AGGCCAGCCACGTTTCTGCGAAAACGCGGGAAAAAGTGGAAGCGGCGATGGCGGAGCTGAATTACATTCCCAACCGCGTG

GCACAACAACTGGCGGGCAAACAGTCGTTGCTGATTGGCGTTGCCACCTCCAGTCTGGCCCTGCACGCGCCGTCGCAAAT

TGTCGCGGCGATTAAATCTCGCGCCGATCAACTGGGTGCCAGCGTGGTGGTGTCGATGGTAGAACGAAGCGGCGTCGAAG

CCTGTAAAGCGGCGGTGCACAATCTTCTCGCGCAACGCGTCAGTGGGCTGATCATTAACTATCCGCTGGATGACCAGGAT

GCCATTGCTGTGGAAGCTGCCTGCACTAATGTTCCGGCGTTATTTCTTGATGTCTCTGACCAGACACCCATCAACAGTAT

TATTTTCTCCCATGAAGACGGTACGCGACTGGGCGTGGAGCATCTGGTCGCATTGGGTCACCAGCAAATCGCGCTGTTAG

CGGGCCCATTAAGTTCTGTCTCGGCGCGTCTGCGTCTGGCTGGCTGGCATAAATATCTCACTCGCAATCAAATTCAGCCG

ATAGCGGAACGGGAAGGCGACTGGAGTGCCATGTCCGGTTTTCAACAAACCATGCAAATGCTGAATGAGGGCATCGTTCC

CACTGCGATGCTGGTTGCCAACGATCAGATGGCGCTGGGCGCAATGCGCGCCATTACCGAGTCCGGGCTGCGCGTTGGTG

CGGATATCTCGGTAGTGGGATACGACGATACCGAAGACAGCTCATGTTATATCCCGCCGTTAACCACCATCAAACAGGAT

TTTCGCCTGCTGGGGCAAACCAGCGTGGACCGCTTGCTGCAACTCTCTCAGGGCCAGGCGGTGAAGGGCAATCAGCTGTT

GCCCGTCTCACTGGTGAAAAGAAAAACCACCCTGGCGCCCAATACGCAAACCGCCTCTCCCCGCGCGTTGGCCGATTCAT

TAATGCAGCTGGCACGACAGGTTTCCCGACTGGAAAGCGGGCAGTGAGCGCAACGCAATTAATGTAAGTTAGCGCGAATT

GATCTGGTTTGACAGCTTATCATCGACTGCACGGTGCACCAATGCTTCTGGCGTCAGGCAGCCATCGGAAGCTGTGGTAT

GGCTGTGCAGGTCGTAAATCACTGCATAATTCGTGTCGCTCAAGGCGCACTCCCGTTCTGGATAATGTTTTTTGCGCCGA

CATCATAACGGTTCTGGCAAATATTCTGAAATGAGCTGTTGACAATTAATCATCCGGCTCGTATAATGTGTGGAATTGTG

AGCGGATAACAATTTCAGAATTCAAAAGATCTTTTAAGAAGGAGATATACATATGAACCACCTAAGAGCAGAAGGCCCAG

CCTCCGTGCTGGCCATTGGTACCGCCAATCCGGAAAACATTTTATTGCAGGACGAATTTCCTGATTATTATTTTCGTGTT

ACCAAATCGGAACACATGACCCAGCTGAAAGAAAAATTTCGCAAAATTTGTGACAAAAGTATGATACGGAAACGAAATTG

TTTTCTGAATGAGGAACACTTGAAGCAAAACCCTCGCTTAGTCGAACATGAGATGCAGACACTGGATGCCCGCCAGGACA

TGCTCGTGGTTGAAGTGCCGAAATTAGGTAAAGACGCGTGCGCGAAGGCGATTAAAGAGTGGGGTCAGCCGAAATCCAAG

ATTACACACTTAATTTTTACCTCAGCGTCGACTACCGATATGCCTGGTGCCGACTATCATTGTGCCAAGTTGTTGGGCTT

AAGCCCCTCGGTTAAGCGTGTGATGATGTACCAACTGGGTTGTTATGGCGGTGGCACGGTGCTGAGAATTGCGAAAGATA

TTGCCGAGAATAATAAAGGCGCGCGTGTTCTGGCCGTTTGCTGTGATATCATGGCATGCCTGTTTCGTGGTCCTTCTGAG

AGCGACTTAGAACTGCTGGTCGGCCAGGCTATATTCGGGGATGGTGCGGCGGCAGTCATTGTTGGCGCGGAACCCGATGA

AAGCGTTGGCGAGCGCCCAATCTTCGAATTAGTAAGCACAGGTCAGACGATATTACCAAATAGCGAAGGCACTATCGGGG

GCCACATCCGCGAAGCTGGACTGATTTTTGACCTGCATAAAGATGTCCCGATGCTGATTAGTAATAATATCGAGAAATGT

CTGATTGAGGCATTTACCCCAATTGGAATTAGCGACTGGAACAGCATTTTCTGGATAACGCATCCGGGCGGCAAAGCTAT

CCTGGATAAAGTTGAGGAAAAGCTCCACCTGAAATCTGATAAGTTTGTAGATTCCCGCCACGTGTTGAGCGAACATGGTA

ATATGAGCTCCTCAACAGTCCTATTCGTTATGGATGAACTGCGCAAACGCTCGCTCGAAGAAGGTAAAAGTACCACCGGC

GATGGCTTTGAATGGGGCGTGTTATTCGGTTTTGGCCCAGGCTTAACCGTTGAGCGTGTGGTGGTACGATCGGTTCCTAT

AAAATATTGACTCTAGAAATAATTTTGTTTAACTTTAAGAAGGAGATATACATATGTCCCCTATACTAGGTTATTGGAAA

ATTAAGGGCCTTGTGCAACCCACTCGACTTCTTTTGGAATATCTTGAAGAAAAATATGAAGAGCATTTGTATGAGCGCGA

TGAAGGTGATAAATGGCGAAACAAAAAGTTTGAATTGGGTTTGGAGTTTCCCAATCTTCCTTATTATATTGATGGTGATG

TTAAATTAACACAGTCTATGGCCATCATACGTTATATAGCTGACAAGCACAACATGTTGGGTGGTTGTCCAAAAGAGCGT

GCAGAGATTTCAATGCTTGAAGGAGCGGTTTTGGATATTAGATACGGTGTTTCGAGAATTGCATATAGTAAAGACTTTGA

AACTCTCAAAGTTGATTTTCTTAGCAAGCTACCTGAAATGCTGAAAATGTTCGAAGATCGTTTATGTCATAAAACATATT

TAAATGGTGATCATGTAACCCATCCTGACTTCATGTTGTATGACGCTCTTGATGTTGTTTTATACATGGACCCAATGTGC

CTGGATGCGTTCCCAAAATTAGTTTGTTTTAAAAAACGTATTGAAGCTATCCCACAAATTGATAAGTACTTGAAATCCAG

CAAGTATATAGCATGGCCTTTGCAGGGCTGGCAAGCCACGTTTGGTGGTGGCGACCATCCTCCAAAATCGGATGGTTCAA

CTAGTGGTTCTGGTCATCACCATCACCATCACTCCATGAGCGATTACGACATCCCCACTACTGAGAATCTTTATTTTCAG

GGCGCCATGGCAGTAAAACATTTAATTGTGCTCAAATTTAAAGACGAAATTACTGAAGCACAAAAAGAAGAGTTTTTCAA

AACATACGTCAATCTGGTGAACATCATCCCGGCCATGAAAGACGTCTATTGGGGCAAAGACGTAACGCAGAAAAACAAGG

AAGAAGGTTATACCCACATCGTGGAGGTCACTTTCGAATCAGTCGAAACGATCCAGGATTACATTATTCATCCGGCACAT

GTAGGGTTCGGCGATGTATATCGTAGCTTCTGGGAAAAACTCCTGATTTTTGACTATACCCCGCGTAAATAAAGCGCAGC

TTGTTGACTTTTACACTTTATGCTTCCGGCTCGTATAATGTGTGGAATTGTGAGCGGATAACAAGAAGCTGAGTTGGAAA

AGAATTCACGGAAGGAGCATGGCGATGCTGCCTCCGAGCGATAGCAAAGATCCGCGTCCGTGGCAGATTCTGAGCCAGGC

ACTGGGTTTTCCGAATTATGATCAAGAACTGTGGTGGCAGAATACCGCAGAAACCCTGAATCGTGTTCTGGAACAGTGTG

ATTATAGCGTTCATCTGCAGTATAAATACCTGGCCTTCTACCACAAATATATCCTGCCGAGCCTGGGTCCGTTTCGTCGT

CCGGGTGTTGAACCGGAATATATCAGCGGTCTGAGTCATGGTGGTCATCCGCTGGAAATTAGCGTGAAAATTGATAAAAG

CAAGACCATTTGTCGTCTGGGTCTGCAGGCAATTGGTCCGCTGGCAGGCACCGCACGTGATCCGCTGAATAGCTTTGGTG

ATCGTGAACTGCTGAAAAATCTGGCAACCCTGCTGCCGCATGTTGATCTGCGTCTGTTTGATCATTTTAATGCACAGGTT

GGTCTGGATCGTGCACAGTGTGCAGTTGCAACCACCAAACTGATTAAAGAAAGCCATAATATTGTGTGCACCAGCCTGGA

TCTGAAAGATGGTGAAGTTATTCCGAAAGTGTACTTTAGCACCATTCCTAAAGGTCTGGTTACCGAAACACCGCTGTTCG

ATCTGACCTTTGCAGCAATTGAGCAGATGGAAGTGTATCATAAAGATGCACCGCTGCGTACCGCACTGAGCAGCCTGAAA

GATTTTCTGCGTCCGCGTGTTCCGACCGATGCAAGCATTACCCCTCCGCTGACCGGTCTGATTGGTGTTGATTGTATTGA

TCCGATGCTGAGCCGTCTGAAAGTTTATCTGGCCACCTTTCGTATGGATCTGAGCCTGATTCGTGATTATTGGACCTTAG

GTGGTCTGCTGACCGATGCCGGTACAATGAAAGGCCTGGAAATGGTTGAAACCCTGGCAAAAACACTGAAACTGGGTGAT

GAAGCATGTGAAACACTGGATGCAGAACGTCTGCCGTTTGGTATTAACTATGCAATGAAACCGGGTACAGCAGAACTGGC

ACCGCCTCAGATCTATTTTCCGCTGCTGGGTATTAATGATGGCTTTATTGCAGATGCCCTGGTGGAATTTTTTCAGTATA

TGGGTTGGGAAGATCAGGCCAATCGTTATAAAGATGAACTGAAAGCCAAATTTCCGAACGTGGATATTAGCCAGACCAAA

AATGTTCATCGTTGGCTGGGTGTTGCATATAGCGAAACCAAAGGTCCGAGCATGAACATCTATTATGATGTTGTTGCAGG

TAATGTGGCACGCGTTTAAACGATCTTAAGTAGGCGCGGAAAATAATGGAGTTCGACTTCAACAAATACATGGACAGCAA

AGCCATGACCGTTAATGAAGCACTGAATAAAGCAATTCCGCTGCGTTATCCGCAGAAAATCTATGAAAGCATGCGTTATA

GCCTGCTGGCAGGCGGTAAACGTGTTCGTCCGGTTCTGTGTATTGCAGCATGTGAACTGGTTGGTGGCACCGAAGAACTG

GCAATTCCGACCGCATGTGCAATTGAAATGATTCATACCATGAGCCTGATGCATGATGATCTGCCGTGTATTGATAATGA

TGACCTGCGTCGTGGTAAACCGACCAATCATAAAATCTTTGGTGAAGATACCGCAGTGACCGCAGGTAATGCACTGCATA

GTTATGCATTTGAACATATTGCAGTGAGCACCAGCAAAACCGTTGGTGCAGATCGTATTCTGCGTATGGTTAGCGAACTG

GGTCGTGCAACCGGTAGCGAAGGTGTTATGGGTGGTCAGATGGTTGATATTGCAAGTGAAGGTGATCCGAGCATTGATCT

GCAGACCCTGGAATGGATTCATATTCATAAAACCGCAATGCTGCTGGAATGTAGCGTTGTTTGTGGTGCAATTATTGGTG

GTGCAAGCGAAATTGTTATTGAACGTGCCCGTCGTTATGCACGTTGTGTTGGTCTGCTGTTTCAGGTTGTTGATGATATT

CTGGATGTGACCAAAAGCAGTGATGAACTGGGCAAAACCGCAGGCAAAGATCTGATTAGCGATAAAGCAACCTATCCGAA

ACTGATGGGTCTGGAAAAAGCCAAAGAATTTTCAGATGAACTGCTGAATCGTGCCAAAGGTGAACTGAGCTGTTTTGATC

CGGTTAAAGCAGCACCGCTGCTGGGTCTGGCAGATTATGTTGCATTTCGTCAGAATTAAGGGATCCAAACTCGAGTAAGG

ATCTCCAGGCATCAAATAAAACGAAAGGCTCAGTCGAAAGACTGGGCCTTTCGTTTTATCTGTTGTTTGTCGGTGAACGC

TCTCTACTAGAGTCACACTGGCTCACCTTCGGGTGGGCCTTTCTGCGTTTATACCTAGGCTACAGCCGATAGTCTGGAAC

AGCGCACTTACGGGTTGCTGCGCAACCCAAGTGCTACCGGCGCGGCAGCGTGACCCGTGTCGGCGGCTCCAACGGCTCGC

CATCGTCCAGAAAACACGGCTCATCGGGCATCGGCAGGCGCTGCTGCCCGCGCCGTTCCCATTCCTCCGTTTCGGTCAAG

GCTGGCAGGTCTGGTTCCATGCCCGGAATGCCGGGCTGGCTGGGCGGCTCCTCGCCGGGGCCGGTCGGTAGTTGCTGCTC

GCCCGGATACAGGGTCGGGATGCGGCGCAGGTCGCCATGCCCCAACAGCGATTCGTCCTGGTCGTCGTGATCAACCACCA

CGGCGGCACTGAACACCGACAGGCGCAACTGGTCGCGGGGCTGGCCCCACGCCACGCGGTCATTGACCACGTAGGCCAAC

ACGGTGCCGGGGCCGTTGAGCTTCACGACGGAGATCCAGCGCTCGGCCACCAAGTCCTTGACTGCGTATTGGACCGTCCG

CAAAGAACGTCCGATGAGCTTGGAAAGTGTCTTCTGGCTGACCACCACGGCGTTCTGGTGGCCCATCTGCGCCACGAGGT

GATGCAGCAGCATTGCCGCCGTGGGTTTCCTCGCAATAAGCCCGGCCCACGCCTCATGCGCTTTGCGTTCCGTTTGCACC

CAGTGACCGGGCTTGTTCTTGGCTTGAATGCCGATTTCTCTGGACTGCGTGGCCATGCTTATCTCCATGCGGTAGGGGTG

CCGCACGGTTGCGGCACCATGCGCAATCAGCTGCAACTTTTCGGCAGCGCGACAACAATTATGCGTTGCGTAAAAGTGGC

AGTCAATTACAGATTTTCTTTAACCTACGCAATGAGCTATTGCGGGGGGTGCCGCAATGAGCTGTTGCGTACCCCCCTTT

TTTAAGTTGTTGATTTTTAAGTCTTTCGCATTTCGCCCTATATCTAGTTCTTTGGTGCCCAAAGAAGGGCACCCCTGCGG

GGTTCCCCCACGCCTTCGGCGCGGCTCCCCCTCCGGCAAAAAGTGGCCCCTCCGGGGCTTGTTGATCGACTGCGCGGCCT

TCGGCCTTGCCCAAGGTGGCGCTGCCCCCTTGGAACCCCCGCACTCGCCGCCGTGAGGCTCGGGGGGCAGGCGGGCGGGC

TTCGCCCTTCGACTGCCCCCACTCGCATAGGCTTGGGTCGTTCCAGGCGCGTCAAGGCCAAGCCGCTGCGCGGTCGCTGC

GCGAGCCTTGACCCGCCTTCCACTTGGTGTCCAACCGGCAAGCGAAGCGCGCAGGCCGCAGGCCGGAGGCACTAGTGCTT

GGATTCTCACCAATAAAAAACGCCCGGCGGCAACCGAGCGTTCTGAACAAATCCAGATGGAGTTCTGAGGTCATTACTGG

ATCTATCAACAGGAGTCCAAGCGAGCTCGATATCAAATTACGCCCCGCCCTGCCACTCATCGCAGTACTGTTGTAATTCA

TTAAGCATTCTGCCGACATGGAAGCCATCACAAACGGCATGATGAACCTGAATCGCCAGCGGCATCAGCACCTTGTCGCC

TTGCGTATAATATTTGCCCATGGTGAAAACGGGGGCGAAGAAGTTGTCCATATTGGCCACGTTTAAATCAAAACTGGTGA

AACTCACCCAGGGATTGGCTGAGACGAAAAACATATTCTCAATAAACCCTTTAGGGAAATAGGCCAGGTTTTCACCGTAA

CACGCCACATCTTGCGAATATATGTGTAGAAACTGCCGGAAATCGTCGTGGTATTCACTCCAGAGCGATGAAAACGTTTC

AGTTTGCTCATGGAAAACGGTGTAACAAGGGTGAACACTATCCCATATCACCAGCTCACCGTCTTTCATTGCCATACGAA

ATTCCGGATGAGCATTCATCAGGCGGGCAAGAATGTGAATAAAGGCCGGATAAAACTTGTGCTTATTTTTCTTTACGGTC

TTTAAAAAGGCCGTAATATCCAGCTGAACGGTCTGGTTATAGGTACATTGAGCAACTGACTGAAATGCCTCAAAATGTTC

TTTACGATGCCATTGGGATATATCAACGGTGGTATATCCAGTGATTTTTTTCTCCATTTTAGCTTCCTTAGCTCCTGAAA

ATCTCGATAACTCAAAAAATACGCCCGGTAGTGATCTTATTTCATTATGGTGAAAGTTGGAACCTCTTACGTGCCGATCA

ACGTCTCATTTTCGCCAGATATC

>pBbB5c-CBGA (Genbank: OQ725953)

GACGTCGGTGCCTAATGAGTGAGCTAACTTACATTAATTGCGTTGCGCTCACTGCCCGCTTTCCAGTCGGGAAACCTGTC

GTGCCAGCTGCATTAATGAATCGGCCAACGCGCGGGGAGAGGCGGTTTGCGTATTGGGCGCCAGGGTGGTTTTTCTTTTC

ACCAGTGAGACGGGCAACAGCTGATTGCCCTTCACCGCCTGGCCCTGAGAGAGTTGCAGCAAGCGGTCCACGCTGGTTTG

CCCCAGCAGGCGAAAATCCTGTTTGATGGTGGTTAACGGCGGGATATAACATGAGCTGTCTTCGGTATCGTCGTATCCCA

CTACCGAGATGTCCGCACCAACGCGCAGCCCGGACTCGGTAATGGCGCGCATTGCGCCCAGCGCCATCTGATCGTTGGCA

ACCAGCATCGCAGTGGGAACGATGCCCTCATTCAGCATTTGCATGGTTTGTTGAAAACCGGACATGGCACTCCAGTCGCC

TTCCCGTTCCGCTATCGGCTGAATTTGATTGCGAGTGAGATATTTATGCCAGCCAGCCAGACGCAGACGCGCCGAGACAG

AACTTAATGGGCCCGCTAACAGCGCGATTTGCTGGTGACCCAATGCGACCAGATGCTCCACGCCCAGTCGCGTACCGTCT

TCATGGGAGAAAATAATACTGTTGATGGGTGTCTGGTCAGAGACATCAAGAAATAACGCCGGAACATTAGTGCAGGCAGC

TTCCACAGCAATGGCATCCTGGTCATCCAGCGGATAGTTAATGATCAGCCCACTGACGCGTTGCGCGAGAAGATTGTGCA

CCGCCGCTTTACAGGCTTCGACGCCGCTTCGTTCTACCATCGACACCACCACGCTGGCACCCAGTTGATCGGCGCGAGAT

TTAATCGCCGCGACAATTTGCGACGGCGCGTGCAGGGCCAGACTGGAGGTGGCAACGCCAATCAGCAACGACTGTTTGCC

CGCCAGTTGTTGTGCCACGCGGTTGGGAATGTAATTCAGCTCCGCCATCGCCGCTTCCACTTTTTCCCGCGTTTTCGCAG

AAACGTGGCTGGCCTGGTTCACCACGCGGGAAACGGTCTGATAAGAGACACCGGCATACTCTGCGACATCGTATAACGTT

ACTGGTTTCACATTCACCACCCTGAATTGACTCTCTTCCGGGCGCTATCATGCCATACCGCGAAAGGTTTTGCGCCATTC

GATGGTGTCCGGGATCTCGACGCTCTCCCTTATGCGACTCCTGCATTAGGAAGCAGCCCAGTAGTAGGTTGAGGCCGTTG

AGCACCGCCGCCGCAAGGAATGGTGCATGCAAGGAGATGGCGCCCAACAGTCCCCCGGCCACGGGGCCTGCCACCATACC

CACGCCGAAACAAGCGCTCATGAGCCCGAAGTGGCGAGCCCGATCTTCCCCATCGGTGATGTCGGCGATATAGGCGCCAG

CAACCGCACCTGTGGCGCCGGTGATGCCGGCCACGATGCGTCCGGCGTAGAGGATCGAGATCGTTTAGGCACCCCAGGCT

TTACACTTTATGCTTCCGGCTCGTATAATGTGTGGAATTGTGAGCGGATAACAATTTCAGAATTCAAAAGATCTTTTAAG

AAGGAGATATACATATGAACCACCTAAGAGCAGAAGGCCCAGCCTCCGTGCTGGCCATTGGTACCGCCAATCCGGAAAAC

ATTTTATTGCAGGACGAATTTCCTGATTATTATTTTCGTGTTACCAAATCGGAACACATGACCCAGCTGAAAGAAAAATT

TCGCAAAATTTGTGACAAAAGTATGATACGGAAACGAAATTGTTTTCTGAATGAGGAACACTTGAAGCAAAACCCTCGCT

TAGTCGAACATGAGATGCAGACACTGGATGCCCGCCAGGACATGCTCGTGGTTGAAGTGCCGAAATTAGGTAAAGACGCG

TGCGCGAAGGCGATTAAAGAGTGGGGTCAGCCGAAATCCAAGATTACACACTTAATTTTTACCTCAGCGTCGACTACCGA

TATGCCTGGTGCCGACTATCATTGTGCCAAGTTGTTGGGCTTAAGCCCCTCGGTTAAGCGTGTGATGATGTACCAACTGG

GTTGTTATGGCGGTGGCACGGTGCTGAGAATTGCGAAAGATATTGCCGAGAATAATAAAGGCGCGCGTGTTCTGGCCGTT

TGCTGTGATATCATGGCATGCCTGTTTCGTGGTCCTTCTGAGAGCGACTTAGAACTGCTGGTCGGCCAGGCTATATTCGG

GGATGGTGCGGCGGCAGTCATTGTTGGCGCGGAACCCGATGAAAGCGTTGGCGAGCGCCCAATCTTCGAATTAGTAAGCA

CAGGTCAGACGATATTACCAAATAGCGAAGGCACTATCGGGGGCCACATCCGCGAAGCTGGACTGATTTTTGACCTGCAT

AAAGATGTCCCGATGCTGATTAGTAATAATATCGAGAAATGTCTGATTGAGGCATTTACCCCAATTGGAATTAGCGACTG

GAACAGCATTTTCTGGATAACGCATCCGGGCGGCAAAGCTATCCTGGATAAAGTTGAGGAAAAGCTCCACCTGAAATCTG

ATAAGTTTGTAGATTCCCGCCACGTGTTGAGCGAACATGGTAATATGAGCTCCTCAACAGTCCTATTCGTTATGGATGAA

CTGCGCAAACGCTCGCTCGAAGAAGGTAAAAGTACCACCGGCGATGGCTTTGAATGGGGCGTGTTATTCGGTTTTGGCCC

AGGCTTAACCGTTGAGCGTGTGGTGGTACGATCGGTTCCTATAAAATATTGACTCTAGAAATAATTTTGTTTAACTTTAA

GAAGGAGATATACATATGTCCCCTATACTAGGTTATTGGAAAATTAAGGGCCTTGTGCAACCCACTCGACTTCTTTTGGA

ATATCTTGAAGAAAAATATGAAGAGCATTTGTATGAGCGCGATGAAGGTGATAAATGGCGAAACAAAAAGTTTGAATTGG

GTTTGGAGTTTCCCAATCTTCCTTATTATATTGATGGTGATGTTAAATTAACACAGTCTATGGCCATCATACGTTATATA

GCTGACAAGCACAACATGTTGGGTGGTTGTCCAAAAGAGCGTGCAGAGATTTCAATGCTTGAAGGAGCGGTTTTGGATAT

TAGATACGGTGTTTCGAGAATTGCATATAGTAAAGACTTTGAAACTCTCAAAGTTGATTTTCTTAGCAAGCTACCTGAAA

TGCTGAAAATGTTCGAAGATCGTTTATGTCATAAAACATATTTAAATGGTGATCATGTAACCCATCCTGACTTCATGTTG

TATGACGCTCTTGATGTTGTTTTATACATGGACCCAATGTGCCTGGATGCGTTCCCAAAATTAGTTTGTTTTAAAAAACG

TATTGAAGCTATCCCACAAATTGATAAGTACTTGAAATCCAGCAAGTATATAGCATGGCCTTTGCAGGGCTGGCAAGCCA

CGTTTGGTGGTGGCGACCATCCTCCAAAATCGGATGGTTCAACTAGTGGTTCTGGTCATCACCATCACCATCACTCCATG

AGCGATTACGACATCCCCACTACTGAGAATCTTTATTTTCAGGGCGCCATGGCAGTAAAACATTTAATTGTGCTCAAATT

TAAAGACGAAATTACTGAAGCACAAAAAGAAGAGTTTTTCAAAACATACGTCAATCTGGTGAACATCATCCCGGCCATGA

AAGACGTCTATTGGGGCAAAGACGTAACGCAGAAAAACAAGGAAGAAGGTTATACCCACATCGTGGAGGTCACTTTCGAA

TCAGTCGAAACGATCCAGGATTACATTATTCATCCGGCACATGTAGGGTTCGGCGATGTATATCGTAGCTTCTGGGAAAA

ACTCCTGATTTTTGACTATACCCCGCGTAAATAAAGAAAAGAATTCACGGAAGGAGCATGGCGATGCTGCCTCCGAGCGA

TAGCAAAGATCCGCGTCCGTGGCAGATTCTGAGCCAGGCACTGGGTTTTCCGAATTATGATCAAGAACTGTGGTGGCAGA

ATACCGCAGAAACCCTGAATCGTGTTCTGGAACAGTGTGATTATAGCGTTCATCTGCAGTATAAATACCTGGCCTTCTAC

CACAAATATATCCTGCCGAGCCTGGGTCCGTTTCGTCGTCCGGGTGTTGAACCGGAATATATCAGCGGTCTGAGTCATGG

TGGTCATCCGCTGGAAATTAGCGTGAAAATTGATAAAAGCAAGACCATTTGTCGTCTGGGTCTGCAGGCAATTGGTCCGC

TGGCAGGCACCGCACGTGATCCGCTGAATAGCTTTGGTGATCGTGAACTGCTGAAAAATCTGGCAACCCTGCTGCCGCAT

GTTGATCTGCGTCTGTTTGATCATTTTAATGCACAGGTTGGTCTGGATCGTGCACAGTGTGCAGTTGCAACCACCAAACT

GATTAAAGAAAGCCATAATATTGTGTGCACCAGCCTGGATCTGAAAGATGGTGAAGTTATTCCGAAAGTGTACTTTAGCA

CCATTCCTAAAGGTCTGGTTACCGAAACACCGCTGTTCGATCTGACCTTTGCAGCAATTGAGCAGATGGAAGTGTATCAT

AAAGATGCACCGCTGCGTACCGCACTGAGCAGCCTGAAAGATTTTCTGCGTCCGCGTGTTCCGACCGATGCAAGCATTAC

CCCTCCGCTGACCGGTCTGATTGGTGTTGATTGTATTGATCCGATGCTGAGCCGTCTGAAAGTTTATCTGGCCACCTTTC

GTATGGATCTGAGCCTGATTCGTGATTATTGGACCTTAGGTGGTCTGCTGACCGATGCCGGTACAATGAAAGGCCTGGAA

ATGGTTGAAACCCTGGCAAAAACACTGAAACTGGGTGATGAAGCATGTGAAACACTGGATGCAGAACGTCTGCCGTTTGG

TATTAACTATGCAATGAAACCGGGTACAGCAGAACTGGCACCGCCTCAGATCTATTTTCCGCTGCTGGGTATTAATGATG

GCTTTATTGCAGATGCCCTGGTGGAATTTTTTCAGTATATGGGTTGGGAAGATCAGGCCAATCGTTATAAAGATGAACTG

AAAGCCAAATTTCCGAACGTGGATATTAGCCAGACCAAAAATGTTCATCGTTGGCTGGGTGTTGCATATAGCGAAACCAA

AGGTCCGAGCATGAACATCTATTATGATGTTGTTGCAGGTAATGTGGCACGCGTTTAAACGATCTTAAGTAGGCGCGGAA

AATAATGGAGTTCGACTTCAACAAATACATGGACAGCAAAGCCATGACCGTTAATGAAGCACTGAATAAAGCAATTCCGC

TGCGTTATCCGCAGAAAATCTATGAAAGCATGCGTTATAGCCTGCTGGCAGGCGGTAAACGTGTTCGTCCGGTTCTGTGT

ATTGCAGCATGTGAACTGGTTGGTGGCACCGAAGAACTGGCAATTCCGACCGCATGTGCAATTGAAATGATTCATACCAT

GAGCCTGATGCATGATGATCTGCCGTGTATTGATAATGATGACCTGCGTCGTGGTAAACCGACCAATCATAAAATCTTTG

GTGAAGATACCGCAGTGACCGCAGGTAATGCACTGCATAGTTATGCATTTGAACATATTGCAGTGAGCACCAGCAAAACC

GTTGGTGCAGATCGTATTCTGCGTATGGTTAGCGAACTGGGTCGTGCAACCGGTAGCGAAGGTGTTATGGGTGGTCAGAT

GGTTGATATTGCAAGTGAAGGTGATCCGAGCATTGATCTGCAGACCCTGGAATGGATTCATATTCATAAAACCGCAATGC

TGCTGGAATGTAGCGTTGTTTGTGGTGCAATTATTGGTGGTGCAAGCGAAATTGTTATTGAACGTGCCCGTCGTTATGCA

CGTTGTGTTGGTCTGCTGTTTCAGGTTGTTGATGATATTCTGGATGTGACCAAAAGCAGTGATGAACTGGGCAAAACCGC

AGGCAAAGATCTGATTAGCGATAAAGCAACCTATCCGAAACTGATGGGTCTGGAAAAAGCCAAAGAATTTTCAGATGAAC

TGCTGAATCGTGCCAAAGGTGAACTGAGCTGTTTTGATCCGGTTAAAGCAGCACCGCTGCTGGGTCTGGCAGATTATGTT

GCATTTCGTCAGAATTAAGGGATCCAAACTCGAGTAAGGATCTCCAGGCATCAAATAAAACGAAAGGCTCAGTCGAAAGA

CTGGGCCTTTCGTTTTATCTGTTGTTTGTCGGTGAACGCTCTCTACTAGAGTCACACTGGCTCACCTTCGGGTGGGCCTT

TCTGCGTTTATACCTAGGCTACAGCCGATAGTCTGGAACAGCGCACTTACGGGTTGCTGCGCAACCCAAGTGCTACCGGC

GCGGCAGCGTGACCCGTGTCGGCGGCTCCAACGGCTCGCCATCGTCCAGAAAACACGGCTCATCGGGCATCGGCAGGCGC

TGCTGCCCGCGCCGTTCCCATTCCTCCGTTTCGGTCAAGGCTGGCAGGTCTGGTTCCATGCCCGGAATGCCGGGCTGGCT

GGGCGGCTCCTCGCCGGGGCCGGTCGGTAGTTGCTGCTCGCCCGGATACAGGGTCGGGATGCGGCGCAGGTCGCCATGCC

CCAACAGCGATTCGTCCTGGTCGTCGTGATCAACCACCACGGCGGCACTGAACACCGACAGGCGCAACTGGTCGCGGGGC

TGGCCCCACGCCACGCGGTCATTGACCACGTAGGCCAACACGGTGCCGGGGCCGTTGAGCTTCACGACGGAGATCCAGCG

CTCGGCCACCAAGTCCTTGACTGCGTATTGGACCGTCCGCAAAGAACGTCCGATGAGCTTGGAAAGTGTCTTCTGGCTGA

CCACCACGGCGTTCTGGTGGCCCATCTGCGCCACGAGGTGATGCAGCAGCATTGCCGCCGTGGGTTTCCTCGCAATAAGC

CCGGCCCACGCCTCATGCGCTTTGCGTTCCGTTTGCACCCAGTGACCGGGCTTGTTCTTGGCTTGAATGCCGATTTCTCT

GGACTGCGTGGCCATGCTTATCTCCATGCGGTAGGGGTGCCGCACGGTTGCGGCACCATGCGCAATCAGCTGCAACTTTT

CGGCAGCGCGACAACAATTATGCGTTGCGTAAAAGTGGCAGTCAATTACAGATTTTCTTTAACCTACGCAATGAGCTATT

GCGGGGGGTGCCGCAATGAGCTGTTGCGTACCCCCCTTTTTTAAGTTGTTGATTTTTAAGTCTTTCGCATTTCGCCCTAT

ATCTAGTTCTTTGGTGCCCAAAGAAGGGCACCCCTGCGGGGTTCCCCCACGCCTTCGGCGCGGCTCCCCCTCCGGCAAAA

AGTGGCCCCTCCGGGGCTTGTTGATCGACTGCGCGGCCTTCGGCCTTGCCCAAGGTGGCGCTGCCCCCTTGGAACCCCCG

CACTCGCCGCCGTGAGGCTCGGGGGGCAGGCGGGCGGGCTTCGCCCTTCGACTGCCCCCACTCGCATAGGCTTGGGTCGT

TCCAGGCGCGTCAAGGCCAAGCCGCTGCGCGGTCGCTGCGCGAGCCTTGACCCGCCTTCCACTTGGTGTCCAACCGGCAA

GCGAAGCGCGCAGGCCGCAGGCCGGAGGCACTAGTGCTTGGATTCTCACCAATAAAAAACGCCCGGCGGCAACCGAGCGT

TCTGAACAAATCCAGATGGAGTTCTGAGGTCATTACTGGATCTATCAACAGGAGTCCAAGCGAGCTCGATATCAAATTAC

GCCCCGCCCTGCCACTCATCGCAGTACTGTTGTAATTCATTAAGCATTCTGCCGACATGGAAGCCATCACAAACGGCATG

ATGAACCTGAATCGCCAGCGGCATCAGCACCTTGTCGCCTTGCGTATAATATTTGCCCATGGTGAAAACGGGGGCGAAGA

AGTTGTCCATATTGGCCACGTTTAAATCAAAACTGGTGAAACTCACCCAGGGATTGGCTGAGACGAAAAACATATTCTCA

ATAAACCCTTTAGGGAAATAGGCCAGGTTTTCACCGTAACACGCCACATCTTGCGAATATATGTGTAGAAACTGCCGGAA

ATCGTCGTGGTATTCACTCCAGAGCGATGAAAACGTTTCAGTTTGCTCATGGAAAACGGTGTAACAAGGGTGAACACTAT

CCCATATCACCAGCTCACCGTCTTTCATTGCCATACGAAATTCCGGATGAGCATTCATCAGGCGGGCAAGAATGTGAATA

AAGGCCGGATAAAACTTGTGCTTATTTTTCTTTACGGTCTTTAAAAAGGCCGTAATATCCAGCTGAACGGTCTGGTTATA

GGTACATTGAGCAACTGACTGAAATGCCTCAAAATGTTCTTTACGATGCCATTGGGATATATCAACGGTGGTATATCCAG

TGATTTTTTTCTCCATTTTAGCTTCCTTAGCTCCTGAAAATCTCGATAACTCAAAAAATACGCCCGGTAGTGATCTTATT

TCATTATGGTGAAAGTTGGAACCTCTTACGTGCCGATCAACGTCTCATTTTCGCCAGATATC

>pBbB5c-CBGA_Ptrc (Genbank: OQ725954)

GACGTCGGTGCCTAATGAGTGAGCTAACTTACATTAATTGCGTTGCGCTCACTGCCCGCTTTCCAGTCGGGAAACCTGTC

GTGCCAGCTGCATTAATGAATCGGCCAACGCGCGGGGAGAGGCGGTTTGCGTATTGGGCGCCAGGGTGGTTTTTCTTTTC

ACCAGTGAGACGGGCAACAGCTGATTGCCCTTCACCGCCTGGCCCTGAGAGAGTTGCAGCAAGCGGTCCACGCTGGTTTG

CCCCAGCAGGCGAAAATCCTGTTTGATGGTGGTTAACGGCGGGATATAACATGAGCTGTCTTCGGTATCGTCGTATCCCA

CTACCGAGATGTCCGCACCAACGCGCAGCCCGGACTCGGTAATGGCGCGCATTGCGCCCAGCGCCATCTGATCGTTGGCA

ACCAGCATCGCAGTGGGAACGATGCCCTCATTCAGCATTTGCATGGTTTGTTGAAAACCGGACATGGCACTCCAGTCGCC

TTCCCGTTCCGCTATCGGCTGAATTTGATTGCGAGTGAGATATTTATGCCAGCCAGCCAGACGCAGACGCGCCGAGACAG

AACTTAATGGGCCCGCTAACAGCGCGATTTGCTGGTGACCCAATGCGACCAGATGCTCCACGCCCAGTCGCGTACCGTCT

TCATGGGAGAAAATAATACTGTTGATGGGTGTCTGGTCAGAGACATCAAGAAATAACGCCGGAACATTAGTGCAGGCAGC

TTCCACAGCAATGGCATCCTGGTCATCCAGCGGATAGTTAATGATCAGCCCACTGACGCGTTGCGCGAGAAGATTGTGCA

CCGCCGCTTTACAGGCTTCGACGCCGCTTCGTTCTACCATCGACACCACCACGCTGGCACCCAGTTGATCGGCGCGAGAT

TTAATCGCCGCGACAATTTGCGACGGCGCGTGCAGGGCCAGACTGGAGGTGGCAACGCCAATCAGCAACGACTGTTTGCC

CGCCAGTTGTTGTGCCACGCGGTTGGGAATGTAATTCAGCTCCGCCATCGCCGCTTCCACTTTTTCCCGCGTTTTCGCAG

AAACGTGGCTGGCCTGGTTCACCACGCGGGAAACGGTCTGATAAGAGACACCGGCATACTCTGCGACATCGTATAACGTT

ACTGGTTTCACATTCACCACCCTGAATTGACTCTCTTCCGGGCGCTATCATGCCATACCGCGAAAGGTTTTGCGCCATTC

GATGGTGTCCGGGATCTCGACGCTCTCCCTTATGCGACTCCTGCATTAGGAAGCAGCCCAGTAGTAGGTTGAGGCCGTTG

AGCACCGCCGCCGCAAGGAATGGTGCATGCAAGGAGATGGCGCCCAACAGTCCCCCGGCCACGGGGCCTGCCACCATACC

CACGCCGAAACAAGCGCTCATGAGCCCGAAGTGGCGAGCCCGATCTTCCCCATCGGTGATGTCGGCGATATAGGCGCCAG

CAACCGCACCTGTGGCGCCGGTGATGCCGGCCACGATGCGTCCGGCGTAGAGGATCGAGATCGTTTAGGCACCCCAGGCT

TTACACTTTATGCTTCCGGCTCGTATAATGTGTGGAATTGTGAGCGGATAACAATTTCAGAATTCAAAAGATCTTTTAAG

AAGGAGATATACATATGAACCACCTAAGAGCAGAAGGCCCAGCCTCCGTGCTGGCCATTGGTACCGCCAATCCGGAAAAC

ATTTTATTGCAGGACGAATTTCCTGATTATTATTTTCGTGTTACCAAATCGGAACACATGACCCAGCTGAAAGAAAAATT

TCGCAAAATTTGTGACAAAAGTATGATACGGAAACGAAATTGTTTTCTGAATGAGGAACACTTGAAGCAAAACCCTCGCT

TAGTCGAACATGAGATGCAGACACTGGATGCCCGCCAGGACATGCTCGTGGTTGAAGTGCCGAAATTAGGTAAAGACGCG

TGCGCGAAGGCGATTAAAGAGTGGGGTCAGCCGAAATCCAAGATTACACACTTAATTTTTACCTCAGCGTCGACTACCGA

TATGCCTGGTGCCGACTATCATTGTGCCAAGTTGTTGGGCTTAAGCCCCTCGGTTAAGCGTGTGATGATGTACCAACTGG

GTTGTTATGGCGGTGGCACGGTGCTGAGAATTGCGAAAGATATTGCCGAGAATAATAAAGGCGCGCGTGTTCTGGCCGTT

TGCTGTGATATCATGGCATGCCTGTTTCGTGGTCCTTCTGAGAGCGACTTAGAACTGCTGGTCGGCCAGGCTATATTCGG

GGATGGTGCGGCGGCAGTCATTGTTGGCGCGGAACCCGATGAAAGCGTTGGCGAGCGCCCAATCTTCGAATTAGTAAGCA

CAGGTCAGACGATATTACCAAATAGCGAAGGCACTATCGGGGGCCACATCCGCGAAGCTGGACTGATTTTTGACCTGCAT

AAAGATGTCCCGATGCTGATTAGTAATAATATCGAGAAATGTCTGATTGAGGCATTTACCCCAATTGGAATTAGCGACTG

GAACAGCATTTTCTGGATAACGCATCCGGGCGGCAAAGCTATCCTGGATAAAGTTGAGGAAAAGCTCCACCTGAAATCTG

ATAAGTTTGTAGATTCCCGCCACGTGTTGAGCGAACATGGTAATATGAGCTCCTCAACAGTCCTATTCGTTATGGATGAA

CTGCGCAAACGCTCGCTCGAAGAAGGTAAAAGTACCACCGGCGATGGCTTTGAATGGGGCGTGTTATTCGGTTTTGGCCC

AGGCTTAACCGTTGAGCGTGTGGTGGTACGATCGGTTCCTATAAAATATTGACTCTAGAAATAATTTTGTTTAACTTTAA

GAAGGAGATATACATATGTCCCCTATACTAGGTTATTGGAAAATTAAGGGCCTTGTGCAACCCACTCGACTTCTTTTGGA

ATATCTTGAAGAAAAATATGAAGAGCATTTGTATGAGCGCGATGAAGGTGATAAATGGCGAAACAAAAAGTTTGAATTGG

GTTTGGAGTTTCCCAATCTTCCTTATTATATTGATGGTGATGTTAAATTAACACAGTCTATGGCCATCATACGTTATATA

GCTGACAAGCACAACATGTTGGGTGGTTGTCCAAAAGAGCGTGCAGAGATTTCAATGCTTGAAGGAGCGGTTTTGGATAT

TAGATACGGTGTTTCGAGAATTGCATATAGTAAAGACTTTGAAACTCTCAAAGTTGATTTTCTTAGCAAGCTACCTGAAA

TGCTGAAAATGTTCGAAGATCGTTTATGTCATAAAACATATTTAAATGGTGATCATGTAACCCATCCTGACTTCATGTTG

TATGACGCTCTTGATGTTGTTTTATACATGGACCCAATGTGCCTGGATGCGTTCCCAAAATTAGTTTGTTTTAAAAAACG

TATTGAAGCTATCCCACAAATTGATAAGTACTTGAAATCCAGCAAGTATATAGCATGGCCTTTGCAGGGCTGGCAAGCCA

CGTTTGGTGGTGGCGACCATCCTCCAAAATCGGATGGTTCAACTAGTGGTTCTGGTCATCACCATCACCATCACTCCATG

AGCGATTACGACATCCCCACTACTGAGAATCTTTATTTTCAGGGCGCCATGGCAGTAAAACATTTAATTGTGCTCAAATT

TAAAGACGAAATTACTGAAGCACAAAAAGAAGAGTTTTTCAAAACATACGTCAATCTGGTGAACATCATCCCGGCCATGA

AAGACGTCTATTGGGGCAAAGACGTAACGCAGAAAAACAAGGAAGAAGGTTATACCCACATCGTGGAGGTCACTTTCGAA

TCAGTCGAAACGATCCAGGATTACATTATTCATCCGGCACATGTAGGGTTCGGCGATGTATATCGTAGCTTCTGGGAAAA

ACTCCTGATTTTTGACTATACCCCGCGTAAATAAAGCGCAGCTTGTTGACTTTGACAATTAATCATCCGGCTCGTATAAT

GTGTGGAATTGTGAGCGGATAACAAGAAGCTGAGTTGGAAAAGAATTCACGGAAGGAGCATGGCGATGCTGCCTCCGAGC

GATAGCAAAGATCCGCGTCCGTGGCAGATTCTGAGCCAGGCACTGGGTTTTCCGAATTATGATCAAGAACTGTGGTGGCA

GAATACCGCAGAAACCCTGAATCGTGTTCTGGAACAGTGTGATTATAGCGTTCATCTGCAGTATAAATACCTGGCCTTCT

ACCACAAATATATCCTGCCGAGCCTGGGTCCGTTTCGTCGTCCGGGTGTTGAACCGGAATATATCAGCGGTCTGAGTCAT

GGTGGTCATCCGCTGGAAATTAGCGTGAAAATTGATAAAAGCAAGACCATTTGTCGTCTGGGTCTGCAGGCAATTGGTCC

GCTGGCAGGCACCGCACGTGATCCGCTGAATAGCTTTGGTGATCGTGAACTGCTGAAAAATCTGGCAACCCTGCTGCCGC

ATGTTGATCTGCGTCTGTTTGATCATTTTAATGCACAGGTTGGTCTGGATCGTGCACAGTGTGCAGTTGCAACCACCAAA

CTGATTAAAGAAAGCCATAATATTGTGTGCACCAGCCTGGATCTGAAAGATGGTGAAGTTATTCCGAAAGTGTACTTTAG

CACCATTCCTAAAGGTCTGGTTACCGAAACACCGCTGTTCGATCTGACCTTTGCAGCAATTGAGCAGATGGAAGTGTATC

ATAAAGATGCACCGCTGCGTACCGCACTGAGCAGCCTGAAAGATTTTCTGCGTCCGCGTGTTCCGACCGATGCAAGCATT

ACCCCTCCGCTGACCGGTCTGATTGGTGTTGATTGTATTGATCCGATGCTGAGCCGTCTGAAAGTTTATCTGGCCACCTT

TCGTATGGATCTGAGCCTGATTCGTGATTATTGGACCTTAGGTGGTCTGCTGACCGATGCCGGTACAATGAAAGGCCTGG

AAATGGTTGAAACCCTGGCAAAAACACTGAAACTGGGTGATGAAGCATGTGAAACACTGGATGCAGAACGTCTGCCGTTT

GGTATTAACTATGCAATGAAACCGGGTACAGCAGAACTGGCACCGCCTCAGATCTATTTTCCGCTGCTGGGTATTAATGA

TGGCTTTATTGCAGATGCCCTGGTGGAATTTTTTCAGTATATGGGTTGGGAAGATCAGGCCAATCGTTATAAAGATGAAC

TGAAAGCCAAATTTCCGAACGTGGATATTAGCCAGACCAAAAATGTTCATCGTTGGCTGGGTGTTGCATATAGCGAAACC

AAAGGTCCGAGCATGAACATCTATTATGATGTTGTTGCAGGTAATGTGGCACGCGTTTAAACGATCTTAAGTAGGCGCGG

AAAATAATGGAGTTCGACTTCAACAAATACATGGACAGCAAAGCCATGACCGTTAATGAAGCACTGAATAAAGCAATTCC

GCTGCGTTATCCGCAGAAAATCTATGAAAGCATGCGTTATAGCCTGCTGGCAGGCGGTAAACGTGTTCGTCCGGTTCTGT

GTATTGCAGCATGTGAACTGGTTGGTGGCACCGAAGAACTGGCAATTCCGACCGCATGTGCAATTGAAATGATTCATACC

ATGAGCCTGATGCATGATGATCTGCCGTGTATTGATAATGATGACCTGCGTCGTGGTAAACCGACCAATCATAAAATCTT

TGGTGAAGATACCGCAGTGACCGCAGGTAATGCACTGCATAGTTATGCATTTGAACATATTGCAGTGAGCACCAGCAAAA

CCGTTGGTGCAGATCGTATTCTGCGTATGGTTAGCGAACTGGGTCGTGCAACCGGTAGCGAAGGTGTTATGGGTGGTCAG

ATGGTTGATATTGCAAGTGAAGGTGATCCGAGCATTGATCTGCAGACCCTGGAATGGATTCATATTCATAAAACCGCAAT

GCTGCTGGAATGTAGCGTTGTTTGTGGTGCAATTATTGGTGGTGCAAGCGAAATTGTTATTGAACGTGCCCGTCGTTATG

CACGTTGTGTTGGTCTGCTGTTTCAGGTTGTTGATGATATTCTGGATGTGACCAAAAGCAGTGATGAACTGGGCAAAACC

GCAGGCAAAGATCTGATTAGCGATAAAGCAACCTATCCGAAACTGATGGGTCTGGAAAAAGCCAAAGAATTTTCAGATGA

ACTGCTGAATCGTGCCAAAGGTGAACTGAGCTGTTTTGATCCGGTTAAAGCAGCACCGCTGCTGGGTCTGGCAGATTATG

TTGCATTTCGTCAGAATTAAGGGATCCAAACTCGAGTAAGGATCTCCAGGCATCAAATAAAACGAAAGGCTCAGTCGAAA

GACTGGGCCTTTCGTTTTATCTGTTGTTTGTCGGTGAACGCTCTCTACTAGAGTCACACTGGCTCACCTTCGGGTGGGCC

TTTCTGCGTTTATACCTAGGCTACAGCCGATAGTCTGGAACAGCGCACTTACGGGTTGCTGCGCAACCCAAGTGCTACCG

GCGCGGCAGCGTGACCCGTGTCGGCGGCTCCAACGGCTCGCCATCGTCCAGAAAACACGGCTCATCGGGCATCGGCAGGC

GCTGCTGCCCGCGCCGTTCCCATTCCTCCGTTTCGGTCAAGGCTGGCAGGTCTGGTTCCATGCCCGGAATGCCGGGCTGG

CTGGGCGGCTCCTCGCCGGGGCCGGTCGGTAGTTGCTGCTCGCCCGGATACAGGGTCGGGATGCGGCGCAGGTCGCCATG

CCCCAACAGCGATTCGTCCTGGTCGTCGTGATCAACCACCACGGCGGCACTGAACACCGACAGGCGCAACTGGTCGCGGG

GCTGGCCCCACGCCACGCGGTCATTGACCACGTAGGCCAACACGGTGCCGGGGCCGTTGAGCTTCACGACGGAGATCCAG

CGCTCGGCCACCAAGTCCTTGACTGCGTATTGGACCGTCCGCAAAGAACGTCCGATGAGCTTGGAAAGTGTCTTCTGGCT

GACCACCACGGCGTTCTGGTGGCCCATCTGCGCCACGAGGTGATGCAGCAGCATTGCCGCCGTGGGTTTCCTCGCAATAA

GCCCGGCCCACGCCTCATGCGCTTTGCGTTCCGTTTGCACCCAGTGACCGGGCTTGTTCTTGGCTTGAATGCCGATTTCT

CTGGACTGCGTGGCCATGCTTATCTCCATGCGGTAGGGGTGCCGCACGGTTGCGGCACCATGCGCAATCAGCTGCAACTT

TTCGGCAGCGCGACAACAATTATGCGTTGCGTAAAAGTGGCAGTCAATTACAGATTTTCTTTAACCTACGCAATGAGCTA

TTGCGGGGGGTGCCGCAATGAGCTGTTGCGTACCCCCCTTTTTTAAGTTGTTGATTTTTAAGTCTTTCGCATTTCGCCCT

ATATCTAGTTCTTTGGTGCCCAAAGAAGGGCACCCCTGCGGGGTTCCCCCACGCCTTCGGCGCGGCTCCCCCTCCGGCAA

AAAGTGGCCCCTCCGGGGCTTGTTGATCGACTGCGCGGCCTTCGGCCTTGCCCAAGGTGGCGCTGCCCCCTTGGAACCCC

CGCACTCGCCGCCGTGAGGCTCGGGGGGCAGGCGGGCGGGCTTCGCCCTTCGACTGCCCCCACTCGCATAGGCTTGGGTC

GTTCCAGGCGCGTCAAGGCCAAGCCGCTGCGCGGTCGCTGCGCGAGCCTTGACCCGCCTTCCACTTGGTGTCCAACCGGC

AAGCGAAGCGCGCAGGCCGCAGGCCGGAGGCACTAGTGCTTGGATTCTCACCAATAAAAAACGCCCGGCGGCAACCGAGC

GTTCTGAACAAATCCAGATGGAGTTCTGAGGTCATTACTGGATCTATCAACAGGAGTCCAAGCGAGCTCGATATCAAATT

ACGCCCCGCCCTGCCACTCATCGCAGTACTGTTGTAATTCATTAAGCATTCTGCCGACATGGAAGCCATCACAAACGGCA

TGATGAACCTGAATCGCCAGCGGCATCAGCACCTTGTCGCCTTGCGTATAATATTTGCCCATGGTGAAAACGGGGGCGAA

GAAGTTGTCCATATTGGCCACGTTTAAATCAAAACTGGTGAAACTCACCCAGGGATTGGCTGAGACGAAAAACATATTCT

CAATAAACCCTTTAGGGAAATAGGCCAGGTTTTCACCGTAACACGCCACATCTTGCGAATATATGTGTAGAAACTGCCGG

AAATCGTCGTGGTATTCACTCCAGAGCGATGAAAACGTTTCAGTTTGCTCATGGAAAACGGTGTAACAAGGGTGAACACT

ATCCCATATCACCAGCTCACCGTCTTTCATTGCCATACGAAATTCCGGATGAGCATTCATCAGGCGGGCAAGAATGTGAA

TAAAGGCCGGATAAAACTTGTGCTTATTTTTCTTTACGGTCTTTAAAAAGGCCGTAATATCCAGCTGAACGGTCTGGTTA

TAGGTACATTGAGCAACTGACTGAAATGCCTCAAAATGTTCTTTACGATGCCATTGGGATATATCAACGGTGGTATATCC

AGTGATTTTTTTCTCCATTTTAGCTTCCTTAGCTCCTGAAAATCTCGATAACTCAAAAAATACGCCCGGTAGTGATCTTA

TTTCATTATGGTGAAAGTTGGAACCTCTTACGTGCCGATCAACGTCTCATTTTCGCCAGATATC

>pBbB5c-CBGA_PlacUV5 (Genbank: OQ725955)

GACGTCGGTGCCTAATGAGTGAGCTAACTTACATTAATTGCGTTGCGCTCACTGCCCGCTTTCCAGTCGGGAAACCTGTC

GTGCCAGCTGCATTAATGAATCGGCCAACGCGCGGGGAGAGGCGGTTTGCGTATTGGGCGCCAGGGTGGTTTTTCTTTTC

ACCAGTGAGACGGGCAACAGCTGATTGCCCTTCACCGCCTGGCCCTGAGAGAGTTGCAGCAAGCGGTCCACGCTGGTTTG

CCCCAGCAGGCGAAAATCCTGTTTGATGGTGGTTAACGGCGGGATATAACATGAGCTGTCTTCGGTATCGTCGTATCCCA

CTACCGAGATGTCCGCACCAACGCGCAGCCCGGACTCGGTAATGGCGCGCATTGCGCCCAGCGCCATCTGATCGTTGGCA

ACCAGCATCGCAGTGGGAACGATGCCCTCATTCAGCATTTGCATGGTTTGTTGAAAACCGGACATGGCACTCCAGTCGCC

TTCCCGTTCCGCTATCGGCTGAATTTGATTGCGAGTGAGATATTTATGCCAGCCAGCCAGACGCAGACGCGCCGAGACAG

AACTTAATGGGCCCGCTAACAGCGCGATTTGCTGGTGACCCAATGCGACCAGATGCTCCACGCCCAGTCGCGTACCGTCT

TCATGGGAGAAAATAATACTGTTGATGGGTGTCTGGTCAGAGACATCAAGAAATAACGCCGGAACATTAGTGCAGGCAGC

TTCCACAGCAATGGCATCCTGGTCATCCAGCGGATAGTTAATGATCAGCCCACTGACGCGTTGCGCGAGAAGATTGTGCA

CCGCCGCTTTACAGGCTTCGACGCCGCTTCGTTCTACCATCGACACCACCACGCTGGCACCCAGTTGATCGGCGCGAGAT

TTAATCGCCGCGACAATTTGCGACGGCGCGTGCAGGGCCAGACTGGAGGTGGCAACGCCAATCAGCAACGACTGTTTGCC

CGCCAGTTGTTGTGCCACGCGGTTGGGAATGTAATTCAGCTCCGCCATCGCCGCTTCCACTTTTTCCCGCGTTTTCGCAG

AAACGTGGCTGGCCTGGTTCACCACGCGGGAAACGGTCTGATAAGAGACACCGGCATACTCTGCGACATCGTATAACGTT

ACTGGTTTCACATTCACCACCCTGAATTGACTCTCTTCCGGGCGCTATCATGCCATACCGCGAAAGGTTTTGCGCCATTC

GATGGTGTCCGGGATCTCGACGCTCTCCCTTATGCGACTCCTGCATTAGGAAGCAGCCCAGTAGTAGGTTGAGGCCGTTG

AGCACCGCCGCCGCAAGGAATGGTGCATGCAAGGAGATGGCGCCCAACAGTCCCCCGGCCACGGGGCCTGCCACCATACC

CACGCCGAAACAAGCGCTCATGAGCCCGAAGTGGCGAGCCCGATCTTCCCCATCGGTGATGTCGGCGATATAGGCGCCAG

CAACCGCACCTGTGGCGCCGGTGATGCCGGCCACGATGCGTCCGGCGTAGAGGATCGAGATCGTTTAGGCACCCCAGGCT

TTACACTTTATGCTTCCGGCTCGTATAATGTGTGGAATTGTGAGCGGATAACAATTTCAGAATTCAAAAGATCTTTTAAG

AAGGAGATATACATATGAACCACCTAAGAGCAGAAGGCCCAGCCTCCGTGCTGGCCATTGGTACCGCCAATCCGGAAAAC

ATTTTATTGCAGGACGAATTTCCTGATTATTATTTTCGTGTTACCAAATCGGAACACATGACCCAGCTGAAAGAAAAATT

TCGCAAAATTTGTGACAAAAGTATGATACGGAAACGAAATTGTTTTCTGAATGAGGAACACTTGAAGCAAAACCCTCGCT

TAGTCGAACATGAGATGCAGACACTGGATGCCCGCCAGGACATGCTCGTGGTTGAAGTGCCGAAATTAGGTAAAGACGCG

TGCGCGAAGGCGATTAAAGAGTGGGGTCAGCCGAAATCCAAGATTACACACTTAATTTTTACCTCAGCGTCGACTACCGA

TATGCCTGGTGCCGACTATCATTGTGCCAAGTTGTTGGGCTTAAGCCCCTCGGTTAAGCGTGTGATGATGTACCAACTGG

GTTGTTATGGCGGTGGCACGGTGCTGAGAATTGCGAAAGATATTGCCGAGAATAATAAAGGCGCGCGTGTTCTGGCCGTT

TGCTGTGATATCATGGCATGCCTGTTTCGTGGTCCTTCTGAGAGCGACTTAGAACTGCTGGTCGGCCAGGCTATATTCGG

GGATGGTGCGGCGGCAGTCATTGTTGGCGCGGAACCCGATGAAAGCGTTGGCGAGCGCCCAATCTTCGAATTAGTAAGCA

CAGGTCAGACGATATTACCAAATAGCGAAGGCACTATCGGGGGCCACATCCGCGAAGCTGGACTGATTTTTGACCTGCAT

AAAGATGTCCCGATGCTGATTAGTAATAATATCGAGAAATGTCTGATTGAGGCATTTACCCCAATTGGAATTAGCGACTG

GAACAGCATTTTCTGGATAACGCATCCGGGCGGCAAAGCTATCCTGGATAAAGTTGAGGAAAAGCTCCACCTGAAATCTG

ATAAGTTTGTAGATTCCCGCCACGTGTTGAGCGAACATGGTAATATGAGCTCCTCAACAGTCCTATTCGTTATGGATGAA

CTGCGCAAACGCTCGCTCGAAGAAGGTAAAAGTACCACCGGCGATGGCTTTGAATGGGGCGTGTTATTCGGTTTTGGCCC

AGGCTTAACCGTTGAGCGTGTGGTGGTACGATCGGTTCCTATAAAATATTGACTCTAGAAATAATTTTGTTTAACTTTAA

GAAGGAGATATACATATGTCCCCTATACTAGGTTATTGGAAAATTAAGGGCCTTGTGCAACCCACTCGACTTCTTTTGGA

ATATCTTGAAGAAAAATATGAAGAGCATTTGTATGAGCGCGATGAAGGTGATAAATGGCGAAACAAAAAGTTTGAATTGG

GTTTGGAGTTTCCCAATCTTCCTTATTATATTGATGGTGATGTTAAATTAACACAGTCTATGGCCATCATACGTTATATA

GCTGACAAGCACAACATGTTGGGTGGTTGTCCAAAAGAGCGTGCAGAGATTTCAATGCTTGAAGGAGCGGTTTTGGATAT

TAGATACGGTGTTTCGAGAATTGCATATAGTAAAGACTTTGAAACTCTCAAAGTTGATTTTCTTAGCAAGCTACCTGAAA

TGCTGAAAATGTTCGAAGATCGTTTATGTCATAAAACATATTTAAATGGTGATCATGTAACCCATCCTGACTTCATGTTG

TATGACGCTCTTGATGTTGTTTTATACATGGACCCAATGTGCCTGGATGCGTTCCCAAAATTAGTTTGTTTTAAAAAACG

TATTGAAGCTATCCCACAAATTGATAAGTACTTGAAATCCAGCAAGTATATAGCATGGCCTTTGCAGGGCTGGCAAGCCA

CGTTTGGTGGTGGCGACCATCCTCCAAAATCGGATGGTTCAACTAGTGGTTCTGGTCATCACCATCACCATCACTCCATG

AGCGATTACGACATCCCCACTACTGAGAATCTTTATTTTCAGGGCGCCATGGCAGTAAAACATTTAATTGTGCTCAAATT

TAAAGACGAAATTACTGAAGCACAAAAAGAAGAGTTTTTCAAAACATACGTCAATCTGGTGAACATCATCCCGGCCATGA

AAGACGTCTATTGGGGCAAAGACGTAACGCAGAAAAACAAGGAAGAAGGTTATACCCACATCGTGGAGGTCACTTTCGAA

TCAGTCGAAACGATCCAGGATTACATTATTCATCCGGCACATGTAGGGTTCGGCGATGTATATCGTAGCTTCTGGGAAAA

ACTCCTGATTTTTGACTATACCCCGCGTAAATAAAGCGCAGCTTGTTGACTTTTACACTTTATGCTTCCGGCTCGTATAA

TGTGTGGAATTGTGAGCGGATAACAAGAAGCTGAGTTGGAAAAGAATTCACGGAAGGAGCATGGCGATGCTGCCTCCGAG

CGATAGCAAAGATCCGCGTCCGTGGCAGATTCTGAGCCAGGCACTGGGTTTTCCGAATTATGATCAAGAACTGTGGTGGC

AGAATACCGCAGAAACCCTGAATCGTGTTCTGGAACAGTGTGATTATAGCGTTCATCTGCAGTATAAATACCTGGCCTTC

TACCACAAATATATCCTGCCGAGCCTGGGTCCGTTTCGTCGTCCGGGTGTTGAACCGGAATATATCAGCGGTCTGAGTCA

TGGTGGTCATCCGCTGGAAATTAGCGTGAAAATTGATAAAAGCAAGACCATTTGTCGTCTGGGTCTGCAGGCAATTGGTC

CGCTGGCAGGCACCGCACGTGATCCGCTGAATAGCTTTGGTGATCGTGAACTGCTGAAAAATCTGGCAACCCTGCTGCCG

CATGTTGATCTGCGTCTGTTTGATCATTTTAATGCACAGGTTGGTCTGGATCGTGCACAGTGTGCAGTTGCAACCACCAA

ACTGATTAAAGAAAGCCATAATATTGTGTGCACCAGCCTGGATCTGAAAGATGGTGAAGTTATTCCGAAAGTGTACTTTA

GCACCATTCCTAAAGGTCTGGTTACCGAAACACCGCTGTTCGATCTGACCTTTGCAGCAATTGAGCAGATGGAAGTGTAT

CATAAAGATGCACCGCTGCGTACCGCACTGAGCAGCCTGAAAGATTTTCTGCGTCCGCGTGTTCCGACCGATGCAAGCAT

TACCCCTCCGCTGACCGGTCTGATTGGTGTTGATTGTATTGATCCGATGCTGAGCCGTCTGAAAGTTTATCTGGCCACCT

TTCGTATGGATCTGAGCCTGATTCGTGATTATTGGACCTTAGGTGGTCTGCTGACCGATGCCGGTACAATGAAAGGCCTG

GAAATGGTTGAAACCCTGGCAAAAACACTGAAACTGGGTGATGAAGCATGTGAAACACTGGATGCAGAACGTCTGCCGTT

TGGTATTAACTATGCAATGAAACCGGGTACAGCAGAACTGGCACCGCCTCAGATCTATTTTCCGCTGCTGGGTATTAATG

ATGGCTTTATTGCAGATGCCCTGGTGGAATTTTTTCAGTATATGGGTTGGGAAGATCAGGCCAATCGTTATAAAGATGAA

CTGAAAGCCAAATTTCCGAACGTGGATATTAGCCAGACCAAAAATGTTCATCGTTGGCTGGGTGTTGCATATAGCGAAAC

CAAAGGTCCGAGCATGAACATCTATTATGATGTTGTTGCAGGTAATGTGGCACGCGTTTAAACGATCTTAAGTAGGCGCG

GAAAATAATGGAGTTCGACTTCAACAAATACATGGACAGCAAAGCCATGACCGTTAATGAAGCACTGAATAAAGCAATTC

CGCTGCGTTATCCGCAGAAAATCTATGAAAGCATGCGTTATAGCCTGCTGGCAGGCGGTAAACGTGTTCGTCCGGTTCTG

TGTATTGCAGCATGTGAACTGGTTGGTGGCACCGAAGAACTGGCAATTCCGACCGCATGTGCAATTGAAATGATTCATAC

CATGAGCCTGATGCATGATGATCTGCCGTGTATTGATAATGATGACCTGCGTCGTGGTAAACCGACCAATCATAAAATCT

TTGGTGAAGATACCGCAGTGACCGCAGGTAATGCACTGCATAGTTATGCATTTGAACATATTGCAGTGAGCACCAGCAAA

ACCGTTGGTGCAGATCGTATTCTGCGTATGGTTAGCGAACTGGGTCGTGCAACCGGTAGCGAAGGTGTTATGGGTGGTCA

GATGGTTGATATTGCAAGTGAAGGTGATCCGAGCATTGATCTGCAGACCCTGGAATGGATTCATATTCATAAAACCGCAA

TGCTGCTGGAATGTAGCGTTGTTTGTGGTGCAATTATTGGTGGTGCAAGCGAAATTGTTATTGAACGTGCCCGTCGTTAT

GCACGTTGTGTTGGTCTGCTGTTTCAGGTTGTTGATGATATTCTGGATGTGACCAAAAGCAGTGATGAACTGGGCAAAAC

CGCAGGCAAAGATCTGATTAGCGATAAAGCAACCTATCCGAAACTGATGGGTCTGGAAAAAGCCAAAGAATTTTCAGATG

AACTGCTGAATCGTGCCAAAGGTGAACTGAGCTGTTTTGATCCGGTTAAAGCAGCACCGCTGCTGGGTCTGGCAGATTAT

GTTGCATTTCGTCAGAATTAAGGGATCCAAACTCGAGTAAGGATCTCCAGGCATCAAATAAAACGAAAGGCTCAGTCGAA

AGACTGGGCCTTTCGTTTTATCTGTTGTTTGTCGGTGAACGCTCTCTACTAGAGTCACACTGGCTCACCTTCGGGTGGGC

CTTTCTGCGTTTATACCTAGGCTACAGCCGATAGTCTGGAACAGCGCACTTACGGGTTGCTGCGCAACCCAAGTGCTACC

GGCGCGGCAGCGTGACCCGTGTCGGCGGCTCCAACGGCTCGCCATCGTCCAGAAAACACGGCTCATCGGGCATCGGCAGG

CGCTGCTGCCCGCGCCGTTCCCATTCCTCCGTTTCGGTCAAGGCTGGCAGGTCTGGTTCCATGCCCGGAATGCCGGGCTG

GCTGGGCGGCTCCTCGCCGGGGCCGGTCGGTAGTTGCTGCTCGCCCGGATACAGGGTCGGGATGCGGCGCAGGTCGCCAT

GCCCCAACAGCGATTCGTCCTGGTCGTCGTGATCAACCACCACGGCGGCACTGAACACCGACAGGCGCAACTGGTCGCGG

GGCTGGCCCCACGCCACGCGGTCATTGACCACGTAGGCCAACACGGTGCCGGGGCCGTTGAGCTTCACGACGGAGATCCA

GCGCTCGGCCACCAAGTCCTTGACTGCGTATTGGACCGTCCGCAAAGAACGTCCGATGAGCTTGGAAAGTGTCTTCTGGC

TGACCACCACGGCGTTCTGGTGGCCCATCTGCGCCACGAGGTGATGCAGCAGCATTGCCGCCGTGGGTTTCCTCGCAATA

AGCCCGGCCCACGCCTCATGCGCTTTGCGTTCCGTTTGCACCCAGTGACCGGGCTTGTTCTTGGCTTGAATGCCGATTTC

TCTGGACTGCGTGGCCATGCTTATCTCCATGCGGTAGGGGTGCCGCACGGTTGCGGCACCATGCGCAATCAGCTGCAACT

TTTCGGCAGCGCGACAACAATTATGCGTTGCGTAAAAGTGGCAGTCAATTACAGATTTTCTTTAACCTACGCAATGAGCT

ATTGCGGGGGGTGCCGCAATGAGCTGTTGCGTACCCCCCTTTTTTAAGTTGTTGATTTTTAAGTCTTTCGCATTTCGCCC

TATATCTAGTTCTTTGGTGCCCAAAGAAGGGCACCCCTGCGGGGTTCCCCCACGCCTTCGGCGCGGCTCCCCCTCCGGCA

AAAAGTGGCCCCTCCGGGGCTTGTTGATCGACTGCGCGGCCTTCGGCCTTGCCCAAGGTGGCGCTGCCCCCTTGGAACCC

CCGCACTCGCCGCCGTGAGGCTCGGGGGGCAGGCGGGCGGGCTTCGCCCTTCGACTGCCCCCACTCGCATAGGCTTGGGT

CGTTCCAGGCGCGTCAAGGCCAAGCCGCTGCGCGGTCGCTGCGCGAGCCTTGACCCGCCTTCCACTTGGTGTCCAACCGG

CAAGCGAAGCGCGCAGGCCGCAGGCCGGAGGCACTAGTGCTTGGATTCTCACCAATAAAAAACGCCCGGCGGCAACCGAG

CGTTCTGAACAAATCCAGATGGAGTTCTGAGGTCATTACTGGATCTATCAACAGGAGTCCAAGCGAGCTCGATATCAAAT

TACGCCCCGCCCTGCCACTCATCGCAGTACTGTTGTAATTCATTAAGCATTCTGCCGACATGGAAGCCATCACAAACGGC

ATGATGAACCTGAATCGCCAGCGGCATCAGCACCTTGTCGCCTTGCGTATAATATTTGCCCATGGTGAAAACGGGGGCGA

AGAAGTTGTCCATATTGGCCACGTTTAAATCAAAACTGGTGAAACTCACCCAGGGATTGGCTGAGACGAAAAACATATTC

TCAATAAACCCTTTAGGGAAATAGGCCAGGTTTTCACCGTAACACGCCACATCTTGCGAATATATGTGTAGAAACTGCCG

GAAATCGTCGTGGTATTCACTCCAGAGCGATGAAAACGTTTCAGTTTGCTCATGGAAAACGGTGTAACAAGGGTGAACAC

TATCCCATATCACCAGCTCACCGTCTTTCATTGCCATACGAAATTCCGGATGAGCATTCATCAGGCGGGCAAGAATGTGA

ATAAAGGCCGGATAAAACTTGTGCTTATTTTTCTTTACGGTCTTTAAAAAGGCCGTAATATCCAGCTGAACGGTCTGGTT

ATAGGTACATTGAGCAACTGACTGAAATGCCTCAAAATGTTCTTTACGATGCCATTGGGATATATCAACGGTGGTATATC

CAGTGATTTTTTTCTCCATTTTAGCTTCCTTAGCTCCTGAAAATCTCGATAACTCAAAAAATACGCCCGGTAGTGATCTT

ATTTCATTATGGTGAAAGTTGGAACCTCTTACGTGCCGATCAACGTCTCATTTTCGCCAGATATC

>pBbB2c-CBGA_pBAD(8938 bp) (Genbank: OQ725956)

GACGTCTTAAGACCCACTTTCACATTTAAGTTGTTTTTCTAATCCGCATATGATCAATTCAAGGCCGAATAAGAAGGCTG

GCTCTGCACCTTGGTGATCAAATAATTCGATAGCTTGTCGTAATAATGGCGGCATACTATCAGTAGTAGGTGTTTCCCTT

TCTTCTTTAGCGACTTGATGCTCTTGATCTTCCAATACGCAACCTAAAGTAAAATGCCCCACAGCGCTGAGTGCATATAA

TGCATTCTCTAGTGAAAAACCTTGTTGGCATAAAAAGGCTAATTGATTTTCGAGAGTTTCATACTGTTTTTCTGTAGGCC

GTGTACCTAAATGTACTTTTGCTCCATCGCGATGACTTAGTAAAGCACATCTAAAACTTTTAGCGTTATTACGTAAAAAA

TCTTGCCAGCTTTCCCCTTCTAAAGGGCAAAAGTGAGTATGGTGCCTATCTAACATCTCAATGGCTAAGGCGTCGAGCAA

AGCCCGCTTATTTTTTACATGCCAATACAATGTAGGCTGCTCTACACCTAGCTTCTGGGCGAGTTTACGGGTTGTTAAAC

CTTCGATTCCGACCTCATTAAGCAGCTCTAATGCGCTGTTAATCACTTTACTTTTATCTAATCTAGACATCATTAATTCC

TAATTTTTGTTGACACTCTATCGTTGATAGAGTTATTTTACCACTCCCTATCAGTGATAGAGAAAAGAATTCAAAAGATC

TTTTAAGAAGCCTTAGGTCAATCCACAAAAGCGGCGCATAAGGAAAACTCCCTAGATTCGAGGACCAACAATGAACCACC

TAAGAGCAGAAGGCCCAGCCTCCGTGCTGGCCATTGGTACCGCCAATCCGGAAAACATTTTATTGCAGGACGAATTTCCT

GATTATTATTTTCGTGTTACCAAATCGGAACACATGACCCAGCTGAAAGAAAAATTTCGCAAAATTTGTGACAAAAGTAT

GATACGGAAACGAAATTGTTTTCTGAATGAGGAACACTTGAAGCAAAACCCTCGCTTAGTCGAACATGAGATGCAGACAC

TGGATGCCCGCCAGGACATGCTCGTGGTTGAAGTGCCGAAATTAGGTAAAGACGCGTGCGCGAAGGCGATTAAAGAGTGG

GGTCAGCCGAAATCCAAGATTACACACTTAATTTTTACCTCAGCGTCGACTACCGATATGCCTGGTGCCGACTATCATTG

TGCCAAGTTGTTGGGCTTAAGCCCCTCGGTTAAGCGTGTGATGATGTACCAACTGGGTTGTTATGGCGGTGGCACGGTGC

TGAGAATTGCGAAAGATATTGCCGAGAATAATAAAGGCGCGCGTGTTCTGGCCGTTTGCTGTGATATCATGGCATGCCTG

TTTCGTGGTCCTTCTGAGAGCGACTTAGAACTGCTGGTCGGCCAGGCTATATTCGGGGATGGTGCGGCGGCAGTCATTGT

TGGCGCGGAACCCGATGAAAGCGTTGGCGAGCGCCCAATCTTCGAATTAGTAAGCACAGGTCAGACGATATTACCAAATA

GCGAAGGCACTATCGGGGGCCACATCCGCGAAGCTGGACTGATTTTTGACCTGCATAAAGATGTCCCGATGCTGATTAGT

AATAATATCGAGAAATGTCTGATTGAGGCATTTACCCCAATTGGAATTAGCGACTGGAACAGCATTTTCTGGATAACGCA

TCCGGGCGGCAAAGCTATCCTGGATAAAGTTGAGGAAAAGCTCCACCTGAAATCTGATAAGTTTGTAGATTCCCGCCACG

TGTTGAGCGAACATGGTAATATGAGCTCCTCAACAGTCCTATTCGTTATGGATGAACTGCGCAAACGCTCGCTCGAAGAA

GGTAAAAGTACCACCGGCGATGGCTTTGAATGGGGCGTGTTATTCGGTTTTGGCCCAGGCTTAACCGTTGAGCGTGTGGT

GGTACGATCGGTTCCTATAAAATATTGACTCTAGAAATAATTTTGTTTAACTTTAAGAAGGAGATATACATATGTCCCCT

ATACTAGGTTATTGGAAAATTAAGGGCCTTGTGCAACCCACTCGACTTCTTTTGGAATATCTTGAAGAAAAATATGAAGA

GCATTTGTATGAGCGCGATGAAGGTGATAAATGGCGAAACAAAAAGTTTGAATTGGGTTTGGAGTTTCCCAATCTTCCTT

ATTATATTGATGGTGATGTTAAATTAACACAGTCTATGGCCATCATACGTTATATAGCTGACAAGCACAACATGTTGGGT

GGTTGTCCAAAAGAGCGTGCAGAGATTTCAATGCTTGAAGGAGCGGTTTTGGATATTAGATACGGTGTTTCGAGAATTGC

ATATAGTAAAGACTTTGAAACTCTCAAAGTTGATTTTCTTAGCAAGCTACCTGAAATGCTGAAAATGTTCGAAGATCGTT

TATGTCATAAAACATATTTAAATGGTGATCATGTAACCCATCCTGACTTCATGTTGTATGACGCTCTTGATGTTGTTTTA

TACATGGACCCAATGTGCCTGGATGCGTTCCCAAAATTAGTTTGTTTTAAAAAACGTATTGAAGCTATCCCACAAATTGA

TAAGTACTTGAAATCCAGCAAGTATATAGCATGGCCTTTGCAGGGCTGGCAAGCCACGTTTGGTGGTGGCGACCATCCTC

CAAAATCGGATGGTTCAACTAGTGGTTCTGGTCATCACCATCACCATCACTCCATGAGCGATTACGACATCCCCACTACT

GAGAATCTTTATTTTCAGGGCGCCATGGCAGTAAAACATTTAATTGTGCTCAAATTTAAAGACGAAATTACTGAAGCACA

AAAAGAAGAGTTTTTCAAAACATACGTCAATCTGGTGAACATCATCCCGGCCATGAAAGACGTCTATTGGGGCAAAGACG

TAACGCAGAAAAACAAGGAAGAAGGTTATACCCACATCGTGGAGGTCACTTTCGAATCAGTCGAAACGATCCAGGATTAC

ATTATTCATCCGGCACATGTAGGGTTCGGCGATGTATATCGTAGCTTCTGGGAAAAACTCCTGATTTTTGACTATACCCC

GCGTAAATAAAGAAAAGAACTAACAAAGCCCGAAAGAGAATATAAAAAGCCAGATTATTAATCCGGCTTTTTTATTATTT

AGGAAGCTGAGTTGGTTCGACGTCTTATGACAACTTGACGGCTACATCATTCACTTTTTCTTCACAACCGGCACGGAACT

CGCTCGGGCTGGCCCCGGTGCATTTTTTAAATACCCGCGAGAAATAGAGTTGATCGTCAAAACCAACATTGCGACCGACG

GTGGCGATAGGCATCCGGGTGGTGCTCAAAAGCAGCTTCGCCTGGCTGATACGTTGGTCCTCGCGCCAGCTTAAGACGCT

AATCCCTAACTGCTGGCGGAAAAGATGTGACAGACGCGACGGCGACAAGCAAACATGCTGTGCGACGCTGGCGATATCAA

AATTGCTGTCTGCCAGGTGATCGCTGATGTACTGACAAGCCTCGCGTACCCGATTATCCATCGGTGGATGGAGCGACTCG

TTAATCGCTTCCATGCGCCGCAGTAACAATTGCTCAAGCAGATTTATCGCCAGCAGCTCCGAATAGCGCCCTTCCCCTTG

CCCGGCGTTAATGATTTGCCCAAACAGGTCGCTGAAATGCGGCTGGTGCGCTTCATCCGGGCGAAAGAACCCCGTATTGG

CAAATATTGACGGCCAGTTAAGCCATTCATGCCAGTAGGCGCGCGGACGAAAGTAAACCCACTGGTGATACCATTCGCGA

GCCTCCGGATGACGACCGTAGTGATGAATCTCTCCTGGCGGGAACAGCAAAATATCACCCGGTCGGCAAACAAATTCTCG

TCCCTGATTTTTCACCACCCCCTGACCGCGAATGGTGAGATTGAGAATATAACCTTTCATTCCCAGCGGTCGGTCGATAA

AAAAATCGAGATAACCGTTGGCCTCAATCGGCGTTAAACCCGCCACCAGATGGGCATTAAACGAGTATCCCGGCAGCAGG

GGATCATTTTGCGCTTCAGCCATACTTTTCATACTCCCGCCATTCAGAGAAGAAACCAATTGTCCATATTGCATCAGACA

TTGCCGTCACTGCGTCTTTTACTGGCTCTTCTCGCTAACCAAACCGGTAACCCCGCTTATTAAAAGCATTCTGTAACAAA

GCGGGACCAAAGCCATGACAAAAACGCGTAACAAAAGTGTCTATAATCACGGCAGAAAAGTCCACATTGATTATTTGCAC

GGCGTCACACTTTGCTATGCCATAGCATTTTTATCCATAAGATTAGCGGATTCTACCTGACGCTTTTTATCGCAACTCTC

TACTGTTTCTCCATACCCGTTTTTTTGGGAATTCAAAAGATCACGGAAGGAGCATGGCGATGCTGCCTCCGAGCGATAGC

AAAGATCCGCGTCCGTGGCAGATTCTGAGCCAGGCACTGGGTTTTCCGAATTATGATCAAGAACTGTGGTGGCAGAATAC

CGCAGAAACCCTGAATCGTGTTCTGGAACAGTGTGATTATAGCGTTCATCTGCAGTATAAATACCTGGCCTTCTACCACA

AATATATCCTGCCGAGCCTGGGTCCGTTTCGTCGTCCGGGTGTTGAACCGGAATATATCAGCGGTCTGAGTCATGGTGGT

CATCCGCTGGAAATTAGCGTGAAAATTGATAAAAGCAAGACCATTTGTCGTCTGGGTCTGCAGGCAATTGGTCCGCTGGC

AGGCACCGCACGTGATCCGCTGAATAGCTTTGGTGATCGTGAACTGCTGAAAAATCTGGCAACCCTGCTGCCGCATGTTG

ATCTGCGTCTGTTTGATCATTTTAATGCACAGGTTGGTCTGGATCGTGCACAGTGTGCAGTTGCAACCACCAAACTGATT

AAAGAAAGCCATAATATTGTGTGCACCAGCCTGGATCTGAAAGATGGTGAAGTTATTCCGAAAGTGTACTTTAGCACCAT

TCCTAAAGGTCTGGTTACCGAAACACCGCTGTTCGATCTGACCTTTGCAGCAATTGAGCAGATGGAAGTGTATCATAAAG

ATGCACCGCTGCGTACCGCACTGAGCAGCCTGAAAGATTTTCTGCGTCCGCGTGTTCCGACCGATGCAAGCATTACCCCT

CCGCTGACCGGTCTGATTGGTGTTGATTGTATTGATCCGATGCTGAGCCGTCTGAAAGTTTATCTGGCCACCTTTCGTAT

GGATCTGAGCCTGATTCGTGATTATTGGACCTTAGGTGGTCTGCTGACCGATGCCGGTACAATGAAAGGCCTGGAAATGG

TTGAAACCCTGGCAAAAACACTGAAACTGGGTGATGAAGCATGTGAAACACTGGATGCAGAACGTCTGCCGTTTGGTATT

AACTATGCAATGAAACCGGGTACAGCAGAACTGGCACCGCCTCAGATCTATTTTCCGCTGCTGGGTATTAATGATGGCTT

TATTGCAGATGCCCTGGTGGAATTTTTTCAGTATATGGGTTGGGAAGATCAGGCCAATCGTTATAAAGATGAACTGAAAG

CCAAATTTCCGAACGTGGATATTAGCCAGACCAAAAATGTTCATCGTTGGCTGGGTGTTGCATATAGCGAAACCAAAGGT

CCGAGCATGAACATCTATTATGATGTTGTTGCAGGTAATGTGGCACGCGTTTAAACGATCTTAAGTAGGCGCGGAAAATA

ATGGAGTTCGACTTCAACAAATACATGGACAGCAAAGCCATGACCGTTAATGAAGCACTGAATAAAGCAATTCCGCTGCG

TTATCCGCAGAAAATCTATGAAAGCATGCGTTATAGCCTGCTGGCAGGCGGTAAACGTGTTCGTCCGGTTCTGTGTATTG

CAGCATGTGAACTGGTTGGTGGCACCGAAGAACTGGCAATTCCGACCGCATGTGCAATTGAAATGATTCATACCATGAGC

CTGATGCATGATGATCTGCCGTGTATTGATAATGATGACCTGCGTCGTGGTAAACCGACCAATCATAAAATCTTTGGTGA

AGATACCGCAGTGACCGCAGGTAATGCACTGCATAGTTATGCATTTGAACATATTGCAGTGAGCACCAGCAAAACCGTTG

GTGCAGATCGTATTCTGCGTATGGTTAGCGAACTGGGTCGTGCAACCGGTAGCGAAGGTGTTATGGGTGGTCAGATGGTT

GATATTGCAAGTGAAGGTGATCCGAGCATTGATCTGCAGACCCTGGAATGGATTCATATTCATAAAACCGCAATGCTGCT

GGAATGTAGCGTTGTTTGTGGTGCAATTATTGGTGGTGCAAGCGAAATTGTTATTGAACGTGCCCGTCGTTATGCACGTT

GTGTTGGTCTGCTGTTTCAGGTTGTTGATGATATTCTGGATGTGACCAAAAGCAGTGATGAACTGGGCAAAACCGCAGGC

AAAGATCTGATTAGCGATAAAGCAACCTATCCGAAACTGATGGGTCTGGAAAAAGCCAAAGAATTTTCAGATGAACTGCT

GAATCGTGCCAAAGGTGAACTGAGCTGTTTTGATCCGGTTAAAGCAGCACCGCTGCTGGGTCTGGCAGATTATGTTGCAT

TTCGTCAGAATTAAGGGATCCAAACTCGAGTAAGGATCTCCAGGCATCAAATAAAACGAAAGGCTCAGTCGAAAGACTGG

GCCTTTCGTTTTATCTGTTGTTTGTCGGTGAACGCTCTCTACTAGAGTCACACTGGCTCACCTTCGGGTGGGCCTTTCTG

CGTTTATACCTAGGCTACAGCCGATAGTCTGGAACAGCGCACTTACGGGTTGCTGCGCAACCCAAGTGCTACCGGCGCGG

CAGCGTGACCCGTGTCGGCGGCTCCAACGGCTCGCCATCGTCCAGAAAACACGGCTCATCGGGCATCGGCAGGCGCTGCT

GCCCGCGCCGTTCCCATTCCTCCGTTTCGGTCAAGGCTGGCAGGTCTGGTTCCATGCCCGGAATGCCGGGCTGGCTGGGC

GGCTCCTCGCCGGGGCCGGTCGGTAGTTGCTGCTCGCCCGGATACAGGGTCGGGATGCGGCGCAGGTCGCCATGCCCCAA

CAGCGATTCGTCCTGGTCGTCGTGATCAACCACCACGGCGGCACTGAACACCGACAGGCGCAACTGGTCGCGGGGCTGGC

CCCACGCCACGCGGTCATTGACCACGTAGGCCAACACGGTGCCGGGGCCGTTGAGCTTCACGACGGAGATCCAGCGCTCG

GCCACCAAGTCCTTGACTGCGTATTGGACCGTCCGCAAAGAACGTCCGATGAGCTTGGAAAGTGTCTTCTGGCTGACCAC

CACGGCGTTCTGGTGGCCCATCTGCGCCACGAGGTGATGCAGCAGCATTGCCGCCGTGGGTTTCCTCGCAATAAGCCCGG

CCCACGCCTCATGCGCTTTGCGTTCCGTTTGCACCCAGTGACCGGGCTTGTTCTTGGCTTGAATGCCGATTTCTCTGGAC

TGCGTGGCCATGCTTATCTCCATGCGGTAGGGGTGCCGCACGGTTGCGGCACCATGCGCAATCAGCTGCAACTTTTCGGC

AGCGCGACAACAATTATGCGTTGCGTAAAAGTGGCAGTCAATTACAGATTTTCTTTAACCTACGCAATGAGCTATTGCGG

GGGGTGCCGCAATGAGCTGTTGCGTACCCCCCTTTTTTAAGTTGTTGATTTTTAAGTCTTTCGCATTTCGCCCTATATCT

AGTTCTTTGGTGCCCAAAGAAGGGCACCCCTGCGGGGTTCCCCCACGCCTTCGGCGCGGCTCCCCCTCCGGCAAAAAGTG

GCCCCTCCGGGGCTTGTTGATCGACTGCGCGGCCTTCGGCCTTGCCCAAGGTGGCGCTGCCCCCTTGGAACCCCCGCACT

CGCCGCCGTGAGGCTCGGGGGGCAGGCGGGCGGGCTTCGCCCTTCGACTGCCCCCACTCGCATAGGCTTGGGTCGTTCCA

GGCGCGTCAAGGCCAAGCCGCTGCGCGGTCGCTGCGCGAGCCTTGACCCGCCTTCCACTTGGTGTCCAACCGGCAAGCGA

AGCGCGCAGGCCGCAGGCCGGAGGCACTAGTGCTTGGATTCTCACCAATAAAAAACGCCCGGCGGCAACCGAGCGTTCTG

AACAAATCCAGATGGAGTTCTGAGGTCATTACTGGATCTATCAACAGGAGTCCAAGCGAGCTCGATATCAAATTACGCCC

CGCCCTGCCACTCATCGCAGTACTGTTGTAATTCATTAAGCATTCTGCCGACATGGAAGCCATCACAAACGGCATGATGA

ACCTGAATCGCCAGCGGCATCAGCACCTTGTCGCCTTGCGTATAATATTTGCCCATGGTGAAAACGGGGGCGAAGAAGTT

GTCCATATTGGCCACGTTTAAATCAAAACTGGTGAAACTCACCCAGGGATTGGCTGAGACGAAAAACATATTCTCAATAA

ACCCTTTAGGGAAATAGGCCAGGTTTTCACCGTAACACGCCACATCTTGCGAATATATGTGTAGAAACTGCCGGAAATCG

TCGTGGTATTCACTCCAGAGCGATGAAAACGTTTCAGTTTGCTCATGGAAAACGGTGTAACAAGGGTGAACACTATCCCA

TATCACCAGCTCACCGTCTTTCATTGCCATACGAAATTCCGGATGAGCATTCATCAGGCGGGCAAGAATGTGAATAAAGG

CCGGATAAAACTTGTGCTTATTTTTCTTTACGGTCTTTAAAAAGGCCGTAATATCCAGCTGAACGGTCTGGTTATAGGTA

CATTGAGCAACTGACTGAAATGCCTCAAAATGTTCTTTACGATGCCATTGGGATATATCAACGGTGGTATATCCAGTGAT

TTTTTTCTCCATTTTAGCTTCCTTAGCTCCTGAAAATCTCGATAACTCAAAAAATACGCCCGGTAGTGATCTTATTTCAT

TATGGTGAAAGTTGGAACCTCTTACGTGCCGATCAACGTCTCATTTTCGCCAGATATC

TKS OAC GPPS AtaPT

>pMVA (Genbank: OQ725957)

ATTTAGAAAAATAAACAAATAGGGGTTCCGCGCACATTTCCCCGAAAAGTGCCACCTGACGTCGGTGCCTAATGAGTGAG

CTAACTTACATTAATTGCGTTGCGCTCACTGCCCGCTTTCCAGTCGGGAAACCTGTCGTGCCAGCTGCATTAATGAATCG

GCCAACGCGCGGGGAGAGGCGGTTTGCGTATTGGGCGCCAGGGTGGTTTTTCTTTTCACCAGTGAGACGGGCAACAGCTG

ATTGCCCTTCACCGCCTGGCCCTGAGAGAGTTGCAGCAAGCGGTCCACGCTGGTTTGCCCCAGCAGGCGAAAATCCTGTT

TGATGGTGGTTAACGGCGGGATATAACATGAGCTGTCTTCGGTATCGTCGTATCCCACTACCGAGATGTCCGCACCAACG

CGCAGCCCGGACTCGGTAATGGCGCGCATTGCGCCCAGCGCCATCTGATCGTTGGCAACCAGCATCGCAGTGGGAACGAT

GCCCTCATTCAGCATTTGCATGGTTTGTTGAAAACCGGACATGGCACTCCAGTCGCCTTCCCGTTCCGCTATCGGCTGAA

TTTGATTGCGAGTGAGATATTTATGCCAGCCAGCCAGACGCAGACGCGCCGAGACAGAACTTAATGGGCCCGCTAACAGC

GCGATTTGCTGGTGACCCAATGCGACCAGATGCTCCACGCCCAGTCGCGTACCGTCTTCATGGGAGAAAATAATACTGTT

GATGGGTGTCTGGTCAGAGACATCAAGAAATAACGCCGGAACATTAGTGCAGGCAGCTTCCACAGCAATGGCATCCTGGT

CATCCAGCGGATAGTTAATGATCAGCCCACTGACGCGTTGCGCGAGAAGATTGTGCACCGCCGCTTTACAGGCTTCGACG

CCGCTTCGTTCTACCATCGACACCACCACGCTGGCACCCAGTTGATCGGCGCGAGATTTAATCGCCGCGACAATTTGCGA

CGGCGCGTGCAGGGCCAGACTGGAGGTGGCAACGCCAATCAGCAACGACTGTTTGCCCGCCAGTTGTTGTGCCACGCGGT

TGGGAATGTAATTCAGCTCCGCCATCGCCGCTTCCACTTTTTCCCGCGTTTTCGCAGAAACGTGGCTGGCCTGGTTCACC

ACGCGGGAAACGGTCTGATAAGAGACACCGGCATACTCTGCGACATCGTATAACGTTACTGGTTTCACATTCACCACCCT

GAATTGACTCTCTTCCGGGCGCTATCATGCCATACCGCGAAAGGTTTTGCGCCATTCGATGGTGTCCGGGATCTCGACGC

TCTCCCTTATGCGACTCCTGCATTAGGAAGCAGCCCAGTAGTAGGTTGAGGCCGTTGAGCACCGCCGCCGCAAGGAATGG

TGCATGCAAGGAGATGGCGCCCAACAGTCCCCCGGCCACGGGGCCTGCCACCATACCCACGCCGAAACAAGCGCTCATGA

GCCCGAAGTGGCGAGCCCGATCTTCCCCATCGGTGATGTCGGCGATATAGGCGCCAGCAACCGCACCTGTGGCGCCGGTG

ATGCCGGCCACGATGCGTCCGGCGTAGAGGATCGAGATCGTTTAGGCACCCCAGGCTTTACACTTTATGCTTCCGGCTCG

TATAATGTGTGGAATTGTGAGCGGATAACAATTTCAGAATTCAAAAGATCTTAGGAGGAATATAAAATGAAAAATTGTGT

CATCGTCAGTGCGGTACGTACTGCTATCGGTAGTTTTAACGGTTCACTCGCTTCCACCAGCGCCATCGACCTGGGGGCGA

CAGTAATTAAAGCCGCCATTGAACGTGCAAAAATCGATTCACAACACGTTGATGAAGTGATTATGGGTAACGTGTTACAA

GCCGGGCTGGGGCAAAATCCGGCGCGTCAGGCACTGTTAAAAAGCGGGCTGGCAGAAACGGTGTGCGGATTCACGGTCAA

TAAAGTATGTGGTTCGGGTCTTAAAAGTGTGGCGCTTGCCGCCCAGGCCATTCAGGCAGGTCAGGCGCAGAGCATTGTGG

CGGGGGGTATGGAAAATATGAGTTTAGCCCCCTACTTACTCGATGCAAAAGCACGCTCTGGTTATCGTCTTGGAGACGGA

CAGGTTTATGACGTAATCCTGCGCGATGGCCTGATGTGCGCCACCCATGGTTATCATATGGGGATTACCGCCGAAAACGT

GGCTAAAGAGTACGGAATTACCCGTGAAATGCAGGATGAACTGGCGCTACATTCACAGCGTAAAGCGGCAGCCGCAATTG

AGTCCGGTGCTTTTACAGCCGAAATCGTCCCGGTAAATGTTGTCACTCGAAAGAAAACCTTCGTCTTCAGTCAAGACGAG

TTCCCGAAAGCGAACTCAACGGCTGAAGCGTTAGGTGCATTGCGCCCGGCCTTCGATAAAGCAGGAACAGTCACCGCTGG

GAACGCGTCTGGTATTAACGACGGTGCTGCCGCTCTGGTGATTATGGAAGAATCTGCGGCGCTGGCAGCAGGCCTTACCC

CCCTGGCTCGCATTAAAAGTTATGCCAGCGGTGGCGTGCCCCCCGCATTGATGGGTATGGGGCCAGTACCTGCCACGCAA

AAAGCGTTACAACTGGCGGGGCTGCAACTGGCGGATATTGATCTCATTGAGGCTAATGAAGCATTTGCTGCACAGTTCCT

TGCCGTTGGGAAAAACCTGGGCTTTGATTCTGAGAAAGTGAATGTCAACGGCGGGGCCATCGCGCTCGGGCATCCTATCG

GTGCCAGTGGTGCTCGTATTCTGGTCACACTATTACATGCCATGCAGGCACGCGATAAAACGCTGGGGCTGGCAACACTG

TGCATTGGCGGCGGTCAGGGAATTGCGATGGTGATTGAACGGTTGAATTGAGGATCTTGAATTAAGGAGGACAGCTAAAT

GACAATAGGTATCGATAAAATAAACTTTTACGTTCCAAAGTACTATGTAGACATGGCTAAATTAGCAGAAGCACGCCAAG

TAGACCCAAACAAATTTTTAATTGGAATTGGTCAAACTGAAATGGCTGTTAGTCCTGTAAACCAAGACATCGTTTCAATG

GGCGCTAACGCTGCTAAGGACATTATAACAGACGAAGACAAAAAGAAAATTGGTATGGTAATTGTGGCAACTGAATCAGC

AGTTGATGCTGCTAAAGCAGCCGCTGTTCAAATTCACAACTTATTAGGTATTCAACCTTTTGCACGCTGCTTTGAAATGA

AAGAAGCTTGTTATGCTGCAACACCAGCAATTCAATTAGCTAAAGATTATTTAGCAACTAGACCGAATGAAAAAGTATTA

GTTATTGCTACAGATACAGCACGTTATGGATTGAACTCAGGCGGCGAGCCAACACAAGGTGCTGGCGCAGTTGCGATGGT

TATTGCACATAATCCAAGCATTTTGGCATTAAATGAAGATGCTGTTGCTTACACTGAAGACGTTTATGATTTCTGGCGTC

CAACTGGACATAAATATCCATTAGTTGATGGTGCATTATCTAAAGATGCTTATATCCGCTCATTCCAACAAAGCTGGAAT

GAATACGCAAAACGTCAAGGTAAGTCGCTAGCTGACTTCGCATCTCTATGCTTCCATGTTCCATTTACAAAAATGGGTAA

AAAGGCATTAGAGTCAATCATTGATAACGCTGATGAAACAACTCAAGAGCGTTTACGTTCAGGATATGAAGATGCTGTAG

ATTATAACCGTTATGTCGGTAATATTTATACTGGATCATTATATTTAAGCCTAATATCATTACTTGAAAATCGAGATTTA

CAAGCTGGTGAAACAATCGGTTTATTCAGTTATGGCTCAGGTTCAGTTGGTGAATTTTATAGTGCGACATTAGTTGAAGG

CTACAAAGATCATTTAGATCAAGCTGCACATAAAGCATTATTAAATAACCGTACTGAAGTATCTGTTGATGCATATGAAA

CATTCTTCAAACGTTTTGATGACGTTGAATTTGACGAAGAACAAGATGCTGTTCATGAAGATCGTCATATTTTCTACTTA

TCAAATATTGAAAATAACGTTCGCGAATATCACAGACCAGAGTAATTAGGATCTATTCAGGAAACAGACCATGTCCATGC

AAAGTTTAGATAAGAATTTTCGACATTTATCTCGTAAAGAAAAGTTACAACAATTGGTTGATAAGCAATGGTTATCAGAA

GAACAATTCGACATTTTACTGAATCATCCATTAATCGATGAAGAAGTAGCCAATAGTTTAATTGAAAATGTCATCGCGCA

AGGTGCATTACCCGTTGGATTATTACCGAATATCATTGTGGACGATAAGGCATATGTTGTACCTATGATGGTGGAAGAGC

CTTCAGTTGTCGCTGCAGCTAGTTATGGTGCAAAGCTAGTGAATCAGACTGGCGGATTTAAAACGGTATCTTCTGAACGT

ATTATGATAGGTCAAATCGTCTTTGATGGCGTTGACGATACTGAAAAATTATCAGCAGACATTAAAGCTTTAGAAAAGCA

AATTCATAAAATTGCGGATGAGGCATATCCTTCTATTAAAGCGCGTGGTGGTGGTTACCAACGTATAGCGATTGATACAT

TTCCTGAGCAACAGTTACTATCTTTAAAAGTATTTGTTGATACGAAAGATGCTATGGGCGCTAATATGCTTAATACGATT

TTAGAGGCCATAACTGCATTTTTAAAAAATGAATTTCCGCAAAGCGACATTTTAATGAGTATTTTATCCAATCATGCAAC

AGCGTCCGTTGTTAAAGTTCAAGGCGAAATTGATGTTAAAGATTTAGCAAGGGGCGAGAGAACTGGAGAAGAGGTTGCCA

AACGAATGGAACGTGCTTCTGTATTGGCCCAAGTAGATATTCATCGTGCAGCAACACATAATAAAGGTGTTATGAATGGC

ATACATGCTGTTGTTTTAGCAACAGGAAATGATACGCGTGGTGCAGAAGCAAGTGCGCATGCATACGCGAGTCGTGACGG

ACAGTATCGTGGTATTGCTACATGGCGTTACGATCAAGATCGTCAACGATTGATTGGTACAATTGAAGTGCCTATGACAT

TGGCAATCGTTGGCGGTGGTACAAAAGTATTACCAATTGCTAAAGCTTCATTAGAGCTACTAAATGTAGAGTCAGCACAA

GAATTAGGTCATGTAGTTGCTGCCGTTGGTTTAGCGCAAAACTTTGCAGCATGTCGCGCGCTTGTGTCAGAAGGTATTCA

ACAAGGTCATATGAGTTTACAATATAAATCATTAGCTATCGTTGTAGGGGCAAAAGGTGATGAAATTGCTAAAGTAGCTG

AAGCTTTGAAAAAAGAACCCCGTGCAAATACACAAGCAGCGGAACATATTTTACAAGAAATTAGACAACAATAAGGATCT

TTTTAAGGATCTCCAGGCATCAAATAAAACGAAAGGCTCAGTCGAAAGACTGGGCCTTTCGTTTTATCTGTTGTTTGTCG

GTGAACGCTCTCTACTAGAGTCACACTGGCTCACCTTCGGGTGGGCCTTTCTGCGTTTATAGCGAATTGATCTGGTTTGA

CAGCTTATCATCGACTGCACGGTGCACCAATGCTTCTGGCGTCAGGCAGCCATCGGAAGCTGTGGTATGGCTGTGCAGGT

CGTAAATCACTGCATAATTCGTGTCGCTCAAGGCGCACTCCCGTTCTGGATAATGTTTTTTGCGCCGACATCATAACGGT

TCTGGCAAATATTCTGAAATGAGCTGTTGACAATTAATCATCCGGCTCGTATAATGTGTGGAATTGTGAGCGGATAACAA

TTTCAGGATCTAGGAGGAAATAACCATGTCTCTGCCATTCCTGACGTCTGCGCCAGGTAAGGTGATCATCTTCGGCGAGC

ACTCTGCGGTGTACAATAAGCCGGCCGTCGCCGCCTCTGTGTCTGCGTTACGCACCTACCTGCTGATCAGCGAATCTTCT

GCACCGGACACGATCGAGCTGGACTTTCCGGACATCAGCTTCAACCACAAGTGGAGCATCAACGACTTCAACGCGATCAC

GGAGGACCAGGTGAACAGCCAAAAGCTGGCCAAAGCCCAGCAAGCAACCGACGGTCTGTCTCAGGAGCTGGTGTCTCTGC

TGGACCCGCTGTTAGCGCAGTTAAGCGAGAGCTTCCATTACCACGCCGCGTTCTGCTTCCTGTACATGTTCGTTTGCCTG

TGCCCGCACGCAAAGAACATCAAGTTCAGCCTGAAGAGCACGCTGCCGATTGGCGCAGGCTTAGGCTCTAGCGCATCTAT

CAGCGTGAGCCTGGCGCTGGCGATGGCCTATCTGGGTGGCCTGATTGGCAGCAACGACCTGGAGAAACTGAGCGAAAACG

ACAAGCACATCGTGAACCAGTGGGCCTTTATCGGCGAGAAGTGCATTCATGGCACCCCGAGCGGCATTGACAACGCAGTT

GCCACGTATGGCAACGCCCTGCTGTTCGAGAAAGACAGCCACAACGGCACGATCAACACGAACAACTTCAAGTTCCTGGA

CGACTTCCCGGCGATCCCGATGATTCTGACCTACACCCGTATCCCACGCAGCACCAAGGATTTAGTCGCCCGCGTGCGTG

TTTTAGTCACCGAAAAGTTCCCGGAGGTGATGAAGCCGATCCTGGACGCGATGGGCGAGTGCGCGCTGCAGGGTCTGGAG

ATCATGACCAAGCTGAGCAAGTGCAAGGGCACCGACGATGAGGCGGTGGAGACCAACAATGAGCTGTACGAGCAGCTGCT

GGAGCTGATCCGTATCAATCACGGCCTGCTGGTCTCTATCGGTGTGTCTCACCCGGGCCTGGAACTGATCAAAAACCTGA

GCGACGACCTGCGCATTGGCTCTACGAAATTAACGGGTGCAGGTGGCGGTGGCTGCTCTTTAACGCTGCTGCGCCGTGAC

ATTACGCAGGAGCAAATCGACAGCTTCAAGAAGAAGCTGCAGGACGACTTCAGCTACGAGACGTTCGAGACGGACCTGGG

CGGCACGGGCTGTTGCCTGCTGAGCGCCAAAAATCTGAACAAGGACCTGAAGATCAAAAGCCTGGTGTTCCAGCTGTTCG

AAAACAAGACGACCACGAAGCAGCAGATCGACGACCTGTTACTGCCGGGTAACACCAATCTGCCGTGGACGTCTTAAGGA

TCTAGGAGGGAGATCATATGAGCGAATTACGTGCATTCAGCGCGCCAGGTAAGGCACTGCTGGCCGGTGGCTACCTGGTG

TTAGACACCAAGTACGAGGCGTTCGTCGTCGGCTTATCTGCCCGTATGCATGCAGTTGCCCACCCGTATGGTAGCCTGCA

GGGCTCTGACAAGTTCGAAGTGCGTGTGAAGAGCAAGCAGTTCAAGGACGGCGAGTGGCTGTACCACATTAGCCCAAAGA

GCGGCTTCATCCCGGTTAGCATTGGTGGCAGCAAGAACCCATTTATCGAGAAGGTCATTGCCAACGTCTTCAGCTACTTC

AAGCCGAATATGGACGATTACTGCAACCGCAACCTGTTCGTCATCGACATTTTCAGCGACGACGCGTACCACAGCCAAGA

GGACTCTGTTACGGAGCATCGTGGTAACCGCCGCCTGAGCTTCCACAGCCATCGCATTGAGGAGGTGCCGAAGACGGGTC

TGGGTTCTAGCGCCGGTTTAGTTACCGTCTTAACGACGGCGTTAGCGAGCTTCTTCGTGAGCGACCTGGAGAACAACGTG

GACAAGTACCGCGAAGTGATTCATAACCTGGCGCAGGTGGCACATTGTCAGGCCCAAGGTAAGATTGGCTCTGGTTTTGA

TGTGGCAGCGGCCGCCTATGGCTCTATCCGCTATCGCCGCTTTCCGCCGGCCCTGATCAGCAATCTGCCGGACATCGGCT

CTGCGACGTATGGTAGCAAACTGGCGCATCTGGTGGACGAAGAAGACTGGAACATCACCATTAAGTCTAATCACCTGCCG

AGCGGCTTAACGTTATGGATGGGCGATATCAAGAACGGCAGCGAAACGGTTAAGCTGGTGCAGAAAGTGAAAAACTGGTA

CGACAGCCACATGCCGGAAAGCCTGAAGATTTACACGGAGCTGGACCACGCCAATAGCCGTTTCATGGATGGTCTGAGCA

AGCTGGACCGCCTGCACGAAACCCACGACGACTACAGCGACCAAATCTTCGAGAGCCTGGAGCGCAATGACTGCACCTGC

CAGAAGTACCCGGAGATCACGGAGGTCCGCGATGCCGTGGCAACGATTCGCCGTAGCTTCCGCAAAATTACGAAGGAGAG

CGGCGCGGATATCGAACCACCGGTCCAGACGTCTCTGCTGGACGACTGTCAAACCTTAAAGGGCGTGTTAACGTGCCTGA

TTCCGGGCGCGGGTGGTTACGACGCCATTGCCGTCATCACGAAACAGGACGTCGATCTGCGCGCACAAACGGCCAACGAC

AAACGTTTCAGCAAAGTCCAATGGCTGGATGTTACGCAGGCCGACTGGGGTGTTCGCAAGGAGAAGGACCCGGAAACGTA

TCTGGATAAGTGAGGATCTAGGAGGATTATGAGATGACCGTTTACACAGCATCCGTTACCGCACCCGTCAACATCGCAAC

CCTTAAGTATTGGGGGAAAAGGGACACGAAGTTGAATCTGCCCACCAATTCGTCCATATCAGTGACTTTATCGCAAGATG

ACCTCAGAACGTTGACCTCTGCGGCTACTGCACCTGAGTTTGAACGCGACACTTTGTGGTTAAATGGAGAACCACACAGC

ATCGACAATGAAAGAACTCAAAATTGTCTGCGCGACCTACGCCAATTAAGAAAGGAAATGGAATCGAAGGACGCCTCATT

GCCCACATTATCTCAATGGAAACTCCACATTGTCTCCGAAAATAACTTTCCTACAGCAGCTGGTTTAGCTTCCTCCGCTG

CTGGCTTTGCTGCATTGGTCTCTGCAATTGCTAAGTTATACCAATTACCACAGTCAACTTCAGAAATATCTAGAATAGCA

AGAAAGGGGTCTGGTTCAGCTTGTAGATCGTTGTTTGGCGGATACGTGGCCTGGGAAATGGGAAAAGCTGAAGATGGTCA

TGATTCCATGGCAGTACAAATCGCAGACAGCTCTGACTGGCCTCAGATGAAAGCTTGTGTCCTAGTTGTCAGCGATATTA

AAAAGGATGTGAGTTCCACTCAGGGTATGCAATTGACCGTGGCAACCTCCGAACTATTTAAAGAAAGAATTGAACATGTC

GTACCAAAGAGATTTGAAGTCATGCGTAAAGCCATTGTTGAAAAAGATTTCGCCACCTTTGCAAAGGAAACAATGATGGA

TTCCAACTCTTTCCATGCCACATGTTTGGACTCTTTCCCTCCAATATTCTACATGAATGACACTTCCAAGCGTATCATCA

GTTGGTGCCACACCATTAATCAGTTTTACGGAGAAACAATCGTTGCATACACGTTTGATGCAGGTCCAAATGCTGTGTTG

TACTACTTAGCTGAAAATGAGTCGAAACTCTTTGCATTTATCTATAAATTGTTTGGCTCTGTTCCTGGATGGGACAAGAA

ATTTACTACTGAGCAGCTTGAGGCTTTCAACCATCAATTTGAATCATCTAACTTTACTGCACGTGAATTGGATCTTGAGT

TGCAAAAGGATGTTGCCAGAGTGATTTTAACTCAAGTCGGTTCAGGCCCACAAGAAACAAACGAATCTTTGATTGACGCA

AAGACTGGTCTACCAAAGGAATAAGGATCTAGGAGGTAATGATAATGCAAACGGAACACGTCATTTTATTGAATGCACAG

GGAGTTCCCACGGGTACGCTGGAAAAGTATGCCGCACACACGGCAGACACCCGCTTACATCTCGCGTTCTCCAGTTGGCT

GTTTAATGCCAAAGGACAATTATTAGTTACCCGCCGCGCACTGAGCAAAAAAGCATGGCCTGGCGTGTGGACTAACTCGG

TTTGTGGGCACCCACAACTGGGAGAAAGCAACGAAGACGCAGTGATCCGCCGTTGCCGTTATGAGCTTGGCGTGGAAATT

ACGCCTCCTGAATCTATCTATCCTGACTTTCGCTACCGCGCCACCGATCCGAGTGGCATTGTGGAAAATGAAGTGTGTCC

GGTATTTGCCGCACGCACCACTAGTGCGTTACAGATCAATGATGATGAAGTGATGGATTATCAATGGTGTGATTTAGCAG

ATGTATTACACGGTATTGATGCCACGCCGTGGGCGTTCAGTCCGTGGATGGTGATGCAGGCGACAAATCGCGAAGCCAGA

AAACGATTATCTGCATTTACCCAGCTTAAATAAGGATCCAAACTCGAGTAAGGATCTCCAGGCATCAAATAAAACGAAAG

GCTCAGTCGAAAGACTGGGCCTTTCGTTTTATCTGTTGTTTGTCGGTGAACGCTCTCTACTAGAGTCACACTGGCTCACC

TTCGGGTGGGCCTTTCTGCGTTTATACCTAGGGATATATTCCGCTTCCTCGCTCACTGACTCGCTACGCTCGGTCGTTCG

ACTGCGGCGAGCGGAAATGGCTTACGAACGGGGCGGAGATTTCCTGGAAGATGCCAGGAAGATACTTAACAGGGAAGTGA

GAGGGCCGCGGCAAAGCCGTTTTTCCATAGGCTCCGCCCCCCTGACAAGCATCACGAAATCTGACGCTCAAATCAGTGGT

GGCGAAACCCGACAGGACTATAAAGATACCAGGCGTTTCCCCCTGGCGGCTCCCTCGTGCGCTCTCCTGTTCCTGCCTTT

CGGTTTACCGGTGTCATTCCGCTGTTATGGCCGCGTTTGTCTCATTCCACGCCTGACACTCAGTTCCGGGTAGGCAGTTC

GCTCCAAGCTGGACTGTATGCACGAACCCCCCGTTCAGTCCGACCGCTGCGCCTTATCCGGTAACTATCGTCTTGAGTCC

AACCCGGAAAGACATGCAAAAGCACCACTGGCAGCAGCCACTGGTAATTGATTTAGAGGAGTTAGTCTTGAAGTCATGCG

CCGGTTAAGGCTAAACTGAAAGGACAAGTTTTGGTGACTGCGCTCCTCCAAGCCAGTTACCTCGGTTCAAAGAGTTGGTA

GCTCAGAGAACCTTCGAAAAACCGCCCTGCAAGGCGGTTTTTTCGTTTTCAGAGCAAGAGATTACGCGCAGACCAAAACG

ATCTCAAGAAGATCATCTTATTAATCAGATAAAATATTTCTAGATTTCAGTGCAATTTATCTCTTCAAATGTAGCACCTG

AAGTCAGCCCCATACGATATAAGTTGTTACTAGTGCTTGGATTCTCACCAATAAAAAACGCCCGGCGGCAACCGAGCGTT

CTGAACAAATCCAGATGGAGTTCTGAGGTCATTACTGGATCTATCAACAGGAGTCCAAGCGAGCTCGTAAACTTGGTCTG

ACAGTTACCTCAGAAGAACTCGTCAAGAAGGCGATAGAAGGCGATGCGCTGCGAATCGGGAGCGGCGATACCGTAAAGCA

CGAGGAAGCGGTCAGCCCATTCGCCGCCAAGCTCTTCAGCAATATCACGGGTAGCCAACGCTATGTCCTGATAGCGGTCC

GCCACACCCAGCCGGCCACAGTCGATGAATCCAGAAAAGCGGCCATTTTCCACCATGATATTCGGCAAGCAGGCATCGCC

ATGGGTCACGACGAGATCCTCGCCGTCGGGCATGCGCGCCTTGAGCCTGGCGAACAGTTCGGCTGGCGCGAGCCCCTGAT

GCTCTTCGTCCAGATCATCCTGATCGACAAGACCGGCTTCCATCCGAGTACGTGCTCGCTCGATGCGATGTTTCGCTTGG

TGGTCGAATGGGCAGGTAGCCGGATCAAGCGTATGCAGCCGCCGCATTGCATCAGCCATGATGGATACTTTCTCGGCAGG

AGCAAGGTGAGATGACAGGAGATCCTGCCCCGGCACTTCGCCCAATAGCAGCCAGTCCCTTCCCGCTTCAGTGACAACGT

CGAGCACAGCTGCGCAAGGAACGCCCGTCGTGGCCAGCCACGATAGCCGCGCTGCCTCGTCCTGCAGTTCATTCAGGGCA

CCGGACAGGTCGGTCTTGACAAAAAGAACCGGGCGCCCCTGCGCTGACAGCCGGAACACGGCGGCATCAGAGCAGCCGAT

TGTCTGTTGTGCCCAGTCATAGCCGAATAGCCTCTCCACCCAAGCGGCCGGAGAACCTGCGTGCAATCCATCTTGTTCAA

TCATGCGAAACGATCCTCATCCTGTCTCTTGATCAGATCATGATCCCCTGCGCCATCAGATCCTTGGCGGCAAGAAAGCC

ATCCAGTTTACTTTGCAGGGCTTCCCAACCTTACCAGAGGGCGCCCCAGCTGGCAATTCCGACGTCGA

>pMVA_NR2 (Genbank: OQ725958)

ATTTAGAAAAATAAACAAATAGGGGTTCCGCGCACATTTCCCCGAAAAGTGCCACCTGACGTCGGTGCCTAATGAGTGAG

CTAACTTACATTAATTGCGTTGCGCTCACTGCCCGCTTTCCAGTCGGGAAACCTGTCGTGCCAGCTGCATTAATGAATCG

GCCAACGCGCGGGGAGAGGCGGTTTGCGTATTGGGCGCCAGGGTGGTTTTTCTTTTCACCAGTGAGACGGGCAACAGCTG

ATTGCCCTTCACCGCCTGGCCCTGAGAGAGTTGCAGCAAGCGGTCCACGCTGGTTTGCCCCAGCAGGCGAAAATCCTGTT

TGATGGTGGTTAACGGCGGGATATAACATGAGCTGTCTTCGGTATCGTCGTATCCCACTACCGAGATGTCCGCACCAACG

CGCAGCCCGGACTCGGTAATGGCGCGCATTGCGCCCAGCGCCATCTGATCGTTGGCAACCAGCATCGCAGTGGGAACGAT

GCCCTCATTCAGCATTTGCATGGTTTGTTGAAAACCGGACATGGCACTCCAGTCGCCTTCCCGTTCCGCTATCGGCTGAA

TTTGATTGCGAGTGAGATATTTATGCCAGCCAGCCAGACGCAGACGCGCCGAGACAGAACTTAATGGGCCCGCTAACAGC

GCGATTTGCTGGTGACCCAATGCGACCAGATGCTCCACGCCCAGTCGCGTACCGTCTTCATGGGAGAAAATAATACTGTT

GATGGGTGTCTGGTCAGAGACATCAAGAAATAACGCCGGAACATTAGTGCAGGCAGCTTCCACAGCAATGGCATCCTGGT

CATCCAGCGGATAGTTAATGATCAGCCCACTGACGCGTTGCGCGAGAAGATTGTGCACCGCCGCTTTACAGGCTTCGACG

CCGCTTCGTTCTACCATCGACACCACCACGCTGGCACCCAGTTGATCGGCGCGAGATTTAATCGCCGCGACAATTTGCGA

CGGCGCGTGCAGGGCCAGACTGGAGGTGGCAACGCCAATCAGCAACGACTGTTTGCCCGCCAGTTGTTGTGCCACGCGGT

TGGGAATGTAATTCAGCTCCGCCATCGCCGCTTCCACTTTTTCCCGCGTTTTCGCAGAAACGTGGCTGGCCTGGTTCACC

ACGCGGGAAACGGTCTGATAAGAGACACCGGCATACTCTGCGACATCGTATAACGTTACTGGTTTCACATTCACCACCCT

GAATTGACTCTCTTCCGGGCGCTATCATGCCATACCGCGAAAGGTTTTGCGCCATTCGATGGTGTCCGGGATCTCGACGC

TCTCCCTTATGCGACTCCTGCATTAGGAAGCAGCCCAGTAGTAGGTTGAGGCCGTTGAGCACCGCCGCCGCAAGGAATGG

TGCATGCAAGGAGATGGCGCCCAACAGTCCCCCGGCCACGGGGCCTGCCACCATACCCACGCCGAAACAAGCGCTCATGA

GCCCGAAGTGGCGAGCCCGATCTTCCCCATCGGTGATGTCGGCGATATAGGCGCCAGCAACCGCACCTGTGGCGCCGGTG

ATGCCGGCCACGATGCGTCCGGCGTAGAGGATCGAGATCGTTTAGGCACCCCAGGCTTTACACTTTATGCTTCCGGCTCG

TATAATGTGTGGAATTGTGAGCGGATAACAATTTCAGAATTCAAAAGATCTTAGGAGGAATATAAAATGAAAAATTGTGT

CATCGTCAGTGCGGTACGTACTGCTATCGGTAGTTTTAACGGTTCACTCGCTTCCACCAGCGCCATCGACCTGGGGGCGA

CAGTAATTAAAGCCGCCATTGAACGTGCAAAAATCGATTCACAACACGTTGATGAAGTGATTATGGGTAACGTGTTACAA

GCCGGGCTGGGGCAAAATCCGGCGCGTCAGGCACTGTTAAAAAGCGGGCTGGCAGAAACGGTGTGCGGATTCACGGTCAA

TAAAGTATGTGGTTCGGGTCTTAAAAGTGTGGCGCTTGCCGCCCAGGCCATTCAGGCAGGTCAGGCGCAGAGCATTGTGG

CGGGGGGTATGGAAAATATGAGTTTAGCCCCCTACTTACTCGATGCAAAAGCACGCTCTGGTTATCGTCTTGGAGACGGA

CAGGTTTATGACGTAATCCTGCGCGATGGCCTGATGTGCGCCACCCATGGTTATCATATGGGGATTACCGCCGAAAACGT

GGCTAAAGAGTACGGAATTACCCGTGAAATGCAGGATGAACTGGCGCTACATTCACAGCGTAAAGCGGCAGCCGCAATTG

AGTCCGGTGCTTTTACAGCCGAAATCGTCCCGGTAAATGTTGTCACTCGAAAGAAAACCTTCGTCTTCAGTCAAGACGAG

TTCCCGAAAGCGAACTCAACGGCTGAAGCGTTAGGTGCATTGCGCCCGGCCTTCGATAAAGCAGGAACAGTCACCGCTGG

GAACGCGTCTGGTATTAACGACGGTGCTGCCGCTCTGGTGATTATGGAAGAATCTGCGGCGCTGGCAGCAGGCCTTACCC

CCCTGGCTCGCATTAAAAGTTATGCCAGCGGTGGCGTGCCCCCCGCATTGATGGGTATGGGGCCAGTACCTGCCACGCAA

AAAGCGTTACAACTGGCGGGGCTGCAACTGGCGGATATTGATCTCATTGAGGCTAATGAAGCATTTGCTGCACAGTTCCT

TGCCGTTGGGAAAAACCTGGGCTTTGATTCTGAGAAAGTGAATGTCAACGGCGGGGCCATCGCGCTCGGGCATCCTATCG

GTGCCAGTGGTGCTCGTATTCTGGTCACACTATTACATGCCATGCAGGCACGCGATAAAACGCTGGGGCTGGCAACACTG

TGCATTGGCGGCGGTCAGGGAATTGCGATGGTGATTGAACGGTTGAATTGAGGATCTTGAATTAAGGAGGACAGCTAAAT

GACAATAGGTATCGATAAAATAAACTTTTACGTTCCAAAGTACTATGTAGACATGGCTAAATTAGCAGAAGCACGCCAAG

TAGACCCAAACAAATTTTTAATTGGAATTGGTCAAACTGAAATGGCTGTTAGTCCTGTAAACCAAGACATCGTTTCAATG

GGCGCTAACGCTGCTAAGGACATTATAACAGACGAAGACAAAAAGAAAATTGGTATGGTAATTGTGGCAACTGAATCAGC

AGTTGATGCTGCTAAAGCAGCCGCTGTTCAAATTCACAACTTATTAGGTATTCAACCTTTTGCACGCTGCTTTGAAATGA

AAGAAGCTTGTTATGCTGCAACACCAGCAATTCAATTAGCTAAAGATTATTTAGCAACTAGACCGAATGAAAAAGTATTA

GTTATTGCTACAGATACAGCACGTTATGGATTGAACTCAGGCGGCGAGCCAACACAAGGTGCTGGCGCAGTTGCGATGGT

TATTGCACATAATCCAAGCATTTTGGCATTAAATGAAGATGCTGTTGCTTACACTGAAGACGTTTATGATTTCTGGCGTC

CAACTGGACATAAATATCCATTAGTTGATGGTGCATTATCTAAAGATGCTTATATCCGCTCATTCCAACAAAGCTGGAAT

GAATACGCAAAACGTCAAGGTAAGTCGCTAGCTGACTTCGCATCTCTATGCTTCCATGTTCCATTTACAAAAATGGGTAA

AAAGGCATTAGAGTCAATCATTGATAACGCTGATGAAACAACTCAAGAGCGTTTACGTTCAGGATATGAAGATGCTGTAG

ATTATAACCGTTATGTCGGTAATATTTATACTGGATCATTATATTTAAGCCTAATATCATTACTTGAAAATCGAGATTTA

CAAGCTGGTGAAACAATCGGTTTATTCAGTTATGGCTCAGGTTCAGTTGGTGAATTTTATAGTGCGACATTAGTTGAAGG

CTACAAAGATCATTTAGATCAAGCTGCACATAAAGCATTATTAAATAACCGTACTGAAGTATCTGTTGATGCATATGAAA

CATTCTTCAAACGTTTTGATGACGTTGAATTTGACGAAGAACAAGATGCTGTTCATGAAGATCGTCATATTTTCTACTTA

TCAAATATTGAAAATAACGTTCGCGAATATCACAGACCAGAGTAATTAGGATCTATTCAGGAAACAGACCATGTCCATGC

AAAGTTTAGATAAGAATTTTCGACATTTATCTCGTAAAGAAAAGTTACAACAATTGGTTGATAAGCAATGGTTATCAGAA

GAACAATTCGACATTTTACTGAATCATCCATTAATCGATGAAGAAGTAGCCAATAGTTTAATTGAAAATGTCATCGCGCA

AGGTGCATTACCCGTTGGATTATTACCGAATATCATTGTGGACGATAAGGCATATGTTGTACCTATGATGGTGGAAGAGC

CTTCAGTTGTCGCTGCAGCTAGTTATGGTGCAAAGCTAGTGAATCAGACTGGCGGATTTAAAACGGTATCTTCTGAACGT

ATTATGATAGGTCAAATCGTCTTTGATGGCGTTGACGATACTGAAAAATTATCAGCAGACATTAAAGCTTTAGAAAAGCA

AATTCATAAAATTGCGGATGAGGCATATCCTTCTATTAAAGCGCGTGGTGGTGGTTACCAACGTATAGCGATTGATACAT

TTCCTGAGCAACAGTTACTATCTTTAAAAGTATTTGTTGATACGAAAGATGCTATGGGCGCTAATATGCTTAATACGATT

TTAGAGGCCATAACTGCATTTTTAAAAAATGAATTTCCGCAAAGCGACATTTTAATGAGTATTTTATCCAATCATGCAAC

AGCGTCCGTTGTTAAAGTTCAAGGCGAAATTGATGTTAAAGATTTAGCAAGGGGCGAGAGAACTGGAGAAGAGGTTGCCA

AACGAATGGAACGTGCTTCTGTATTGGCCCAAGTAGATATTCATCGTGCAGCAACACATAATAAAGGTGTTATGAATGGC

ATACATGCTGTTGTTTTAGCAACAGGAAATGATACGCGTGGTGCAGAAGCAAGTGCGCATGCATACGCGAGTCGTGACGG

ACAGTATCGTGGTATTGCTACATGGCGTTACGATCAAGATCGTCAACGATTGATTGGTACAATTGAAGTGCCTATGACAT

TGGCAATCGTTGGCGGTGGTACAAAAGTATTACCAATTGCTAAAGCTTCATTAGAGCTACTAAATGTAGAGTCAGCACAA

GAATTAGGTCATGTAGTTGCTGCCGTTGGTTTAGCGCAAAACTTTGCAGCATGTCGCGCGCTTGTGTCAGAAGGTATTCA

ACAAGGTCATATGAGTTTACAATATAAATCATTAGCTATCGTTGTAGGGGCAAAAGGTGATGAAATTGCTAAAGTAGCTG

AAGCTTTGAAAAAAGAACCCCGTGCAAATACACAAGCAGCGGAACATATTTTACAAGAAATTAGACAACAATAAGGATCT

TGACAGCTAGCTCAGTCCTAGGGACTATGCTAGCGGATCTAGGAGGAAATAACCATGTCTCTGCCATTCCTGACGTCTGC

GCCAGGTAAGGTGATCATCTTCGGCGAGCACTCTGCGGTGTACAATAAGCCGGCCGTCGCCGCCTCTGTGTCTGCGTTAC

GCACCTACCTGCTGATCAGCGAATCTTCTGCACCGGACACGATCGAGCTGGACTTTCCGGACATCAGCTTCAACCACAAG

TGGAGCATCAACGACTTCAACGCGATCACGGAGGACCAGGTGAACAGCCAAAAGCTGGCCAAAGCCCAGCAAGCAACCGA

CGGTCTGTCTCAGGAGCTGGTGTCTCTGCTGGACCCGCTGTTAGCGCAGTTAAGCGAGAGCTTCCATTACCACGCCGCGT

TCTGCTTCCTGTACATGTTCGTTTGCCTGTGCCCGCACGCAAAGAACATCAAGTTCAGCCTGAAGAGCACGCTGCCGATT

GGCGCAGGCTTAGGCTCTAGCGCATCTATCAGCGTGAGCCTGGCGCTGGCGATGGCCTATCTGGGTGGCCTGATTGGCAG

CAACGACCTGGAGAAACTGAGCGAAAACGACAAGCACATCGTGAACCAGTGGGCCTTTATCGGCGAGAAGTGCATTCATG

GCACCCCGAGCGGCATTGACAACGCAGTTGCCACGTATGGCAACGCCCTGCTGTTCGAGAAAGACAGCCACAACGGCACG

ATCAACACGAACAACTTCAAGTTCCTGGACGACTTCCCGGCGATCCCGATGATTCTGACCTACACCCGTATCCCACGCAG

CACCAAGGATTTAGTCGCCCGCGTGCGTGTTTTAGTCACCGAAAAGTTCCCGGAGGTGATGAAGCCGATCCTGGACGCGA

TGGGCGAGTGCGCGCTGCAGGGTCTGGAGATCATGACCAAGCTGAGCAAGTGCAAGGGCACCGACGATGAGGCGGTGGAG

ACCAACAATGAGCTGTACGAGCAGCTGCTGGAGCTGATCCGTATCAATCACGGCCTGCTGGTCTCTATCGGTGTGTCTCA

CCCGGGCCTGGAACTGATCAAAAACCTGAGCGACGACCTGCGCATTGGCTCTACGAAATTAACGGGTGCAGGTGGCGGTG

GCTGCTCTTTAACGCTGCTGCGCCGTGACATTACGCAGGAGCAAATCGACAGCTTCAAGAAGAAGCTGCAGGACGACTTC

AGCTACGAGACGTTCGAGACGGACCTGGGCGGCACGGGCTGTTGCCTGCTGAGCGCCAAAAATCTGAACAAGGACCTGAA

GATCAAAAGCCTGGTGTTCCAGCTGTTCGAAAACAAGACGACCACGAAGCAGCAGATCGACGACCTGTTACTGCCGGGTA

ACACCAATCTGCCGTGGACGTCTTAAGGATCTAGGAGGGAGATCATATGAGCGAATTACGTGCATTCAGCGCGCCAGGTA

AGGCACTGCTGGCCGGTGGCTACCTGGTGTTAGACACCAAGTACGAGGCGTTCGTCGTCGGCTTATCTGCCCGTATGCAT

GCAGTTGCCCACCCGTATGGTAGCCTGCAGGGCTCTGACAAGTTCGAAGTGCGTGTGAAGAGCAAGCAGTTCAAGGACGG

CGAGTGGCTGTACCACATTAGCCCAAAGAGCGGCTTCATCCCGGTTAGCATTGGTGGCAGCAAGAACCCATTTATCGAGA

AGGTCATTGCCAACGTCTTCAGCTACTTCAAGCCGAATATGGACGATTACTGCAACCGCAACCTGTTCGTCATCGACATT

TTCAGCGACGACGCGTACCACAGCCAAGAGGACTCTGTTACGGAGCATCGTGGTAACCGCCGCCTGAGCTTCCACAGCCA

TCGCATTGAGGAGGTGCCGAAGACGGGTCTGGGTTCTAGCGCCGGTTTAGTTACCGTCTTAACGACGGCGTTAGCGAGCT

TCTTCGTGAGCGACCTGGAGAACAACGTGGACAAGTACCGCGAAGTGATTCATAACCTGGCGCAGGTGGCACATTGTCAG

GCCCAAGGTAAGATTGGCTCTGGTTTTGATGTGGCAGCGGCCGCCTATGGCTCTATCCGCTATCGCCGCTTTCCGCCGGC

CCTGATCAGCAATCTGCCGGACATCGGCTCTGCGACGTATGGTAGCAAACTGGCGCATCTGGTGGACGAAGAAGACTGGA

ACATCACCATTAAGTCTAATCACCTGCCGAGCGGCTTAACGTTATGGATGGGCGATATCAAGAACGGCAGCGAAACGGTT

AAGCTGGTGCAGAAAGTGAAAAACTGGTACGACAGCCACATGCCGGAAAGCCTGAAGATTTACACGGAGCTGGACCACGC

CAATAGCCGTTTCATGGATGGTCTGAGCAAGCTGGACCGCCTGCACGAAACCCACGACGACTACAGCGACCAAATCTTCG

AGAGCCTGGAGCGCAATGACTGCACCTGCCAGAAGTACCCGGAGATCACGGAGGTCCGCGATGCCGTGGCAACGATTCGC

CGTAGCTTCCGCAAAATTACGAAGGAGAGCGGCGCGGATATCGAACCACCGGTCCAGACGTCTCTGCTGGACGACTGTCA

AACCTTAAAGGGCGTGTTAACGTGCCTGATTCCGGGCGCGGGTGGTTACGACGCCATTGCCGTCATCACGAAACAGGACG

TCGATCTGCGCGCACAAACGGCCAACGACAAACGTTTCAGCAAAGTCCAATGGCTGGATGTTACGCAGGCCGACTGGGGT

GTTCGCAAGGAGAAGGACCCGGAAACGTATCTGGATAAGTGAGGATCTAGGAGGATTATGAGATGACCGTTTACACAGCA

TCCGTTACCGCACCCGTCAACATCGCAACCCTTAAGTATTGGGGGAAAAGGGACACGAAGTTGAATCTGCCCACCAATTC

GTCCATATCAGTGACTTTATCGCAAGATGACCTCAGAACGTTGACCTCTGCGGCTACTGCACCTGAGTTTGAACGCGACA

CTTTGTGGTTAAATGGAGAACCACACAGCATCGACAATGAAAGAACTCAAAATTGTCTGCGCGACCTACGCCAATTAAGA

AAGGAAATGGAATCGAAGGACGCCTCATTGCCCACATTATCTCAATGGAAACTCCACATTGTCTCCGAAAATAACTTTCC

TACAGCAGCTGGTTTAGCTTCCTCCGCTGCTGGCTTTGCTGCATTGGTCTCTGCAATTGCTAAGTTATACCAATTACCAC

AGTCAACTTCAGAAATATCTAGAATAGCAAGAAAGGGGTCTGGTTCAGCTTGTAGATCGTTGTTTGGCGGATACGTGGCC

TGGGAAATGGGAAAAGCTGAAGATGGTCATGATTCCATGGCAGTACAAATCGCAGACAGCTCTGACTGGCCTCAGATGAA

AGCTTGTGTCCTAGTTGTCAGCGATATTAAAAAGGATGTGAGTTCCACTCAGGGTATGCAATTGACCGTGGCAACCTCCG

AACTATTTAAAGAAAGAATTGAACATGTCGTACCAAAGAGATTTGAAGTCATGCGTAAAGCCATTGTTGAAAAAGATTTC

GCCACCTTTGCAAAGGAAACAATGATGGATTCCAACTCTTTCCATGCCACATGTTTGGACTCTTTCCCTCCAATATTCTA

CATGAATGACACTTCCAAGCGTATCATCAGTTGGTGCCACACCATTAATCAGTTTTACGGAGAAACAATCGTTGCATACA

CGTTTGATGCAGGTCCAAATGCTGTGTTGTACTACTTAGCTGAAAATGAGTCGAAACTCTTTGCATTTATCTATAAATTG

TTTGGCTCTGTTCCTGGATGGGACAAGAAATTTACTACTGAGCAGCTTGAGGCTTTCAACCATCAATTTGAATCATCTAA

CTTTACTGCACGTGAATTGGATCTTGAGTTGCAAAAGGATGTTGCCAGAGTGATTTTAACTCAAGTCGGTTCAGGCCCAC

AAGAAACAAACGAATCTTTGATTGACGCAAAGACTGGTCTACCAAAGGAATAAGGATCTAGGAGGTAATGATAATGCAAA

CGGAACACGTCATTTTATTGAATGCACAGGGAGTTCCCACGGGTACGCTGGAAAAGTATGCCGCACACACGGCAGACACC

CGCTTACATCTCGCGTTCTCCAGTTGGCTGTTTAATGCCAAAGGACAATTATTAGTTACCCGCCGCGCACTGAGCAAAAA

AGCATGGCCTGGCGTGTGGACTAACTCGGTTTGTGGGCACCCACAACTGGGAGAAAGCAACGAAGACGCAGTGATCCGCC

GTTGCCGTTATGAGCTTGGCGTGGAAATTACGCCTCCTGAATCTATCTATCCTGACTTTCGCTACCGCGCCACCGATCCG

AGTGGCATTGTGGAAAATGAAGTGTGTCCGGTATTTGCCGCACGCACCACTAGTGCGTTACAGATCAATGATGATGAAGT

GATGGATTATCAATGGTGTGATTTAGCAGATGTATTACACGGTATTGATGCCACGCCGTGGGCGTTCAGTCCGTGGATGG

TGATGCAGGCGACAAATCGCGAAGCCAGAAAACGATTATCTGCATTTACCCAGCTTAAATAAGGATCCAAACTCGAGTAA

GGATCTCCAGGCATCAAATAAAACGAAAGGCTCAGTCGAAAGACTGGGCCTTTCGTTTTATCTGTTGTTTGTCGGTGAAC

GCTCTCTACTAGAGTCACACTGGCTCACCTTCGGGTGGGCCTTTCTGCGTTTATACCTAGGGATATATTCCGCTTCCTCG

CTCACTGACTCGCTACGCTCGGTCGTTCGACTGCGGCGAGCGGAAATGGCTTACGAACGGGGCGGAGATTTCCTGGAAGA

TGCCAGGAAGATACTTAACAGGGAAGTGAGAGGGCCGCGGCAAAGCCGTTTTTCCATAGGCTCCGCCCCCCTGACAAGCA

TCACGAAATCTGACGCTCAAATCAGTGGTGGCGAAACCCGACAGGACTATAAAGATACCAGGCGTTTCCCCCTGGCGGCT

CCCTCGTGCGCTCTCCTGTTCCTGCCTTTCGGTTTACCGGTGTCATTCCGCTGTTATGGCCGCGTTTGTCTCATTCCACG

CCTGACACTCAGTTCCGGGTAGGCAGTTCGCTCCAAGCTGGACTGTATGCACGAACCCCCCGTTCAGTCCGACCGCTGCG

CCTTATCCGGTAACTATCGTCTTGAGTCCAACCCGGAAAGACATGCAAAAGCACCACTGGCAGCAGCCACTGGTAATTGA

TTTAGAGGAGTTAGTCTTGAAGTCATGCGCCGGTTAAGGCTAAACTGAAAGGACAAGTTTTGGTGACTGCGCTCCTCCAA

GCCAGTTACCTCGGTTCAAAGAGTTGGTAGCTCAGAGAACCTTCGAAAAACCGCCCTGCAAGGCGGTTTTTTCGTTTTCA

GAGCAAGAGATTACGCGCAGACCAAAACGATCTCAAGAAGATCATCTTATTAATCAGATAAAATATTTCTAGATTTCAGT

GCAATTTATCTCTTCAAATGTAGCACCTGAAGTCAGCCCCATACGATATAAGTTGTTACTAGTGCTTGGATTCTCACCAA

TAAAAAACGCCCGGCGGCAACCGAGCGTTCTGAACAAATCCAGATGGAGTTCTGAGGTCATTACTGGATCTATCAACAGG

AGTCCAAGCGAGCTCGTAAACTTGGTCTGACAGTTACCTCAGAAGAACTCGTCAAGAAGGCGATAGAAGGCGATGCGCTG

CGAATCGGGAGCGGCGATACCGTAAAGCACGAGGAAGCGGTCAGCCCATTCGCCGCCAAGCTCTTCAGCAATATCACGGG

TAGCCAACGCTATGTCCTGATAGCGGTCCGCCACACCCAGCCGGCCACAGTCGATGAATCCAGAAAAGCGGCCATTTTCC

ACCATGATATTCGGCAAGCAGGCATCGCCATGGGTCACGACGAGATCCTCGCCGTCGGGCATGCGCGCCTTGAGCCTGGC

GAACAGTTCGGCTGGCGCGAGCCCCTGATGCTCTTCGTCCAGATCATCCTGATCGACAAGACCGGCTTCCATCCGAGTAC

GTGCTCGCTCGATGCGATGTTTCGCTTGGTGGTCGAATGGGCAGGTAGCCGGATCAAGCGTATGCAGCCGCCGCATTGCA

TCAGCCATGATGGATACTTTCTCGGCAGGAGCAAGGTGAGATGACAGGAGATCCTGCCCCGGCACTTCGCCCAATAGCAG

CCAGTCCCTTCCCGCTTCAGTGACAACGTCGAGCACAGCTGCGCAAGGAACGCCCGTCGTGGCCAGCCACGATAGCCGCG

CTGCCTCGTCCTGCAGTTCATTCAGGGCACCGGACAGGTCGGTCTTGACAAAAAGAACCGGGCGCCCCTGCGCTGACAGC

CGGAACACGGCGGCATCAGAGCAGCCGATTGTCTGTTGTGCCCAGTCATAGCCGAATAGCCTCTCCACCCAAGCGGCCGG

AGAACCTGCGTGCAATCCATCTTGTTCAATCATGCGAAACGATCCTCATCCTGTCTCTTGATCAGATCATGATCCCCTGC

GCCATCAGATCCTTGGCGGCAAGAAAGCCATCCAGTTTACTTTGCAGGGCTTCCCAACCTTACCAGAGGGCGCCCCAGCT

GGCAATTCCGACGTCGA

EcAtoB SaHMGS SaHMGR ScMK ScPMK ScPMD Ecidi

**References**

1. Leferink, N.G., Jervis, A.J., Zebec, Z., Toogood, H.S., Hay, S., Takano, E., and Scrutton, N.S. (2016). A ‘Plug and Play’ platform for the production of diverse monoterpene hydrocarbon scaffolds in *Escherichia coli*. *ChemistrySelect,* 1, 1893-1896.

2. Jervis, A.J., Carbonell, P., Taylor, S., Sung, R., Dunstan, M.S., Robinson, C.J., Breitling, R., Takano, E., and Scrutton, N.S. (2019). SelProm: A queryable and predictive expression vector selection tool for *Escherichia coli*. *ACS Synth. Biol.,* 8, 1478-1483.

3. Kumano, T., Richard, S.B., Noel, J.P., Nishiyama, M., and Kuzuyama, T. (2008). Chemoenzymatic syntheses of prenylated aromatic small molecules using *Streptomyces* prenyltransferases with relaxed substrate specificities. *Bioorg. Med. Chem.,* 16, 8117-8126.

4. Valliere, M.A., Korman, T.P., Woodall, N.B., Khitrov, G.A., Taylor, R.E., Baker, D., and Bowie, J.U. (2019). A cell-free platform for the prenylation of natural products and application to cannabinoid production. *Nat. Commun.,* 10, 565.

5. Dong, H., Tao, W., Zhang, Y., and Li, Y. (2012). Development of an anhydrotetracycline-inducible gene expression system for solvent-producing *Clostridium acetobutylicum*: A useful tool for strain engineering. *Metab. Eng.,* 14, 59-67.

6. Blazeck, J., Garg, R., Reed, B., and Alper, H.S. (2012). Controlling promoter strength and regulation in *Saccharomyces cerevisiae* using synthetic hybrid promoters. *Biotechnol. Bioeng.,* 109, 2884-2895.

7. Anthony, J.R., Anthony, L.C., Nowroozi, F., Kwon, G., Newman, J.D., and Keasling, J.D. (2009). Optimization of the mevalonate-based isoprenoid biosynthetic pathway in *Escherichia coli* for production of the anti-malarial drug precursor amorpha-4,11-diene. *Metab. Eng.,* 11, 13-19.

8. Alonso-Gutierrez, J., Chan, R., Batth, T.S., Adams, P.D., Keasling, J.D., Petzold, C.J., and Lee, T.S. (2013). Metabolic engineering of *Escherichia coli* for limonene and perillyl alcohol production. *Metab. Eng.,* 19, 33-41.

9. Rinaldi, M.A., Tait, S., Toogood, H.S., and Scrutton, N.S. (2022). Bioproduction of linalool from paper mill waste. *Front. Bioeng. Biotechnol.,* 10, 892896.

10. Tan, Z., Clomburg, J.M., and Gonzalez, R. (2018). Synthetic pathway for the production of olivetolic acid in *Escherichia coli*. *ACS Synth. Biol.,* 7, 1886-1896.

11. Kearsey, L.J., Prandi, N., Karuppiah, V., Yan, C., Leys, D., Toogood, H., Takano, E., and Scrutton, N.S. (2020). Structure of the *Cannabis sativa* olivetol-producing enzyme reveals cyclization plasticity in type III polyketide synthases. *FEBS J.,* 287, 1511-1524.

12. Lim, C.G., Fowler, Z.L., Hueller, T., Schaffer, S., and Koffas, M.A. (2011). High-yield resveratrol production in engineered *Escherichia coli*. *Appl. Environ. Microbiol.,* 77, 3451-3460.

13. Milke, L., and Marienhagen, J. (2020). Engineering intracellular malonyl-CoA availability in microbial hosts and its impact on polyketide and fatty acid synthesis. *Appl. Microbiol. Biotechnol.,* 104, 6057-6065.

14. Pryce, G., Visintin, C., Ramagopalan, S.V., Al-Izki, S., De Faveri, L.E., Nuamah, R.A., Mein, C.A., Montpetit, A., Hardcastle, A.J., Kooij, G., de Vries, H.E., Amor, S., Thomas, S.A., Ledent, C., Marsicano, G., Lutz, B., Thompson, A.J., Selwood, D.L., Giovannoni, G., and Baker, D. (2014). Control of spasticity in a multiple sclerosis model using central nervous system-excluded CB1 cannabinoid receptor agonists. *FASEB J.,* 28, 117-130.

15. Carbonell, P., Jervis, A.J., Robinson, C.J., Yan, C., Dunstan, M., Swainston, N., Vinaixa, M., Hollywood, K.A., Currin, A., Rattray, N.J.W., Taylor, S., Spiess, R., Sung, R., Williams, A.R., Fellows, D., Stanford, N.J., Mulherin, P., Le Feuvre, R., Barran, P., Goodacre, R., Turner, N.J., Goble, C., Chen, G.G., Kell, D.B., Micklefield, J., Breitling, R., Takano, E., Faulon, J.L., and Scrutton, N.S. (2018). An automated Design-Build-Test-Learn pipeline for enhanced microbial production of fine chemicals. *Commun. Biol.,* 1, 66.

16. Wu, J., Du, G., Zhou, J., and Chen, J. (2013). Metabolic engineering of *Escherichia coli* for (2S)-pinocembrin production from glucose by a modular metabolic strategy. *Metab. Eng.,* 16, 48-55.

17. Huang, H., Levin, E.J., Liu, S., Bai, Y., Lockless, S.W., and Zhou, M. (2014). Structure of a membrane-embedded prenyltransferase homologous to UBIAD1. *PLoS Biol,* 12, e1001911.

18. Pettersen, E.F., Goddard, T.D., Huang, C.C., Couch, G.S., Greenblatt, D.M., Meng, E.C., and Ferrin, T.E. (2004). UCSF Chimera--a visualization system for exploratory research and analysis. *J. Comput. Chem.,* 25, 1605-1612.

19. Briggs, G.E., and Haldane, J.B. (1925). A note on the kinetics of enzyme action. *Biochem. J.,* 19, 338-339.

20. Lee, T.S., Krupa, R.A., Zhang, F., Hajimorad, M., Holtz, W.J., Prasad, N., Lee, S.K., and Keasling, J.D. (2011). BglBrick vectors and datasheets: A synthetic biology platform for gene expression. *J. Biol. Eng.,* 5, 12.
